# Supplementary material for: Quantitative mappings between symmetry and topology in solids
Source: Nat Commun. 2018 Aug 30;9:3530. doi: 10.1038/s41467-018-06010-w (PMC6117291; doi:10.1038/s41467-018-06010-w)
Supplement: Supplementary file 1 — Supplementary Information [file 41467_2018_6010_MOESM1_ESM.pdf]

Supplementary information —

Quantitative mappings between symmetry and topology in solids

Song et al.

Supplementary Note 1. Topology of layer construction

General consideration

A general layer consistent with a lattice is described by a set of Miller indices (mnl) and the distance to the origin point d. Formally, we denote such a layer as $(mnl;d)$

$$(mnl;d) = \Big\{ \mathbf{r} | \mathbf{r}\cdot(m\mathbf{b}_1 + n\mathbf{b}_2 + l\mathbf{b}_3) = 2\pi\,(d+q) \quad q\in\mathbb{Z},\ 0\le d<1 \Big\} \tag{1}$$

where $\mathbf{b}_{1,2,3}$ are the reciprocal lattices. Due to the translation symmetry, the layer consists of an infinite number of planes, each of which is represented by an integer q. For a layer, denoted as L, in a particular space group (SG) $\mathcal{G}$, its symmetry property is described by its little group, which is defined as the subgroup of $\mathcal{G}$ that leaves L invariant

$$\mathcal{S}(L) = \{ s\in\mathcal{G} | sL = L \} \tag{2}$$

$\mathcal{S}$ must also be a SG containing the full translation subgroup since by definition any lattice vector would translate the layer in Supplementary Eq. (1) to itself. Thus the SG can be decomposed as a finite number of cosets of $\mathcal{S}$

$$\mathcal{G} = g_0\mathcal{S} + g_1\mathcal{S} + \cdots \tag{3}$$

By applying all the coset representatives on L, we get a set of symmetric layers

$$\{ g_0 L, g_1 L, \cdots \} \tag{4}$$

An elementary layer construction (eLC) can be got by decorating the layers in $\{g_0L, g_1L,\cdots\}$ with a set of symmetric 2D topological states, i.e., 2D topological insulators protected by time-reversal symmetry (TIs) or 2D mirror topological crystalline insulators (TCIs) with mirror Chern number 1. We denote the eLC generated from L as eLC(L). A general layer construction (LC) can be got by stacking a finite number of eLCs together. Formally, a LC state can be expressed as the direct product of some eLC states

$$C = E_1^{\otimes c_1} \otimes E_2^{\otimes c_2} \otimes \cdots \tag{5}$$

Here C represent the LC, E_1, $E_2\cdots$ are the eLCs, and $c_1, c_2\cdots$ are integers representing the multiplicities of these eLCs.

In this work, the topology of a LC C is represented by a set of invariants, denoted as $\{\delta(C)\}$. The invariant set should be properly designed such that (i) it is complete for LC states, i.e., any two topologically different LCs have different invariants, and (ii) each invariant in it is additive, i.e.,

$$\delta(C\otimes C') = \delta(C) + \delta(C') \tag{6}$$

Here the strong TI invariant, denoted as δ_t, is not included because the strong TI can not be constructed from 2D topological states. From Supplementary Eq. (5) and (6) it is direct to show that the invariants of a LC are completely determined by the constituent eLCs and the corresponding expansion coefficients

$$\delta(C) = c_1\delta(E_1) + c_2\delta(E_2) + \cdots \mod N_\delta \tag{7}$$

Here N_δ is the order of the additive group formed by δ. For example, for mirror Chern number $N_\delta=\infty$ and for hourglass invariant $N_\delta = 2$. Hereafter, we will say that two LCs are *equivalent* with each other if their invariant sets are same. Consequently, although a LC with negative coefficients on eLCs seems not physical it can be equivalent with a physical one. In this sense, we generalize the coefficients in Supplementary Eq. (7) from non-negative integers to any integers. Therefore, all the possible topologies can be easily found once we got all the nonequivalent eLCs. In Supplementary Note 3 we will give the systematic method to generate all these eLCs. For now, let us introduce the invariant set, which consists of four kinds of known invariants and two newly found invariants. Since for any single spatial operation we have either found its corresponding TCI invariant or proved the nonexistence of TCI invariant, we *conjecture* that the six kinds of invariants are complete for LC states.

Four kinds of topological crystalline insulator invariants

The weak invariants $\delta_{\mathrm{w},i=1,2,3}$. These three $\mathbb{Z}_2$ numbers are associated with three primitive lattice bases $\mathbf{a}_{1,2,3}$, and, a nontrivial invariant implies that gapless modes must exist on the surfaces preserving the translation symmetry generated from the corresponding lattice base. Here we give the method to calculate the weak invariants of an eLC. As shown in Supplementary Figure 1a, for a layer given in Supplementary Eq. (1) we can easily calculate its intersections with $\mathbf{a}_{1,2,3}$ as

$$\frac{d+q}{m},\ \frac{d+q}{n},\ \frac{d+q}{l} \tag{8}$$

respectively. Thus, on an $\mathbf{a}_1$ ($\mathbf{a}_2$, $\mathbf{a}_3$) preserving surface, the layer cuts $\mathbf{a}_1$ ($\mathbf{a}_2$, $\mathbf{a}_3$) by m (n, l) times per surface unit cell and each cut contributes to a helical mode if the layer is filled with 2D TI. As each pair of helical modes can be trivialized without breaking the translation symmetry, the i-th weak invariant of an eLC should be nontrivial only if the layers in it cuts $\mathbf{a}_i$ odd times per unit cell. Therefore, we have

$$\delta_{\mathrm{w},1}(E) = \sum_{L\in E} m_L \mod 2 \tag{9}$$

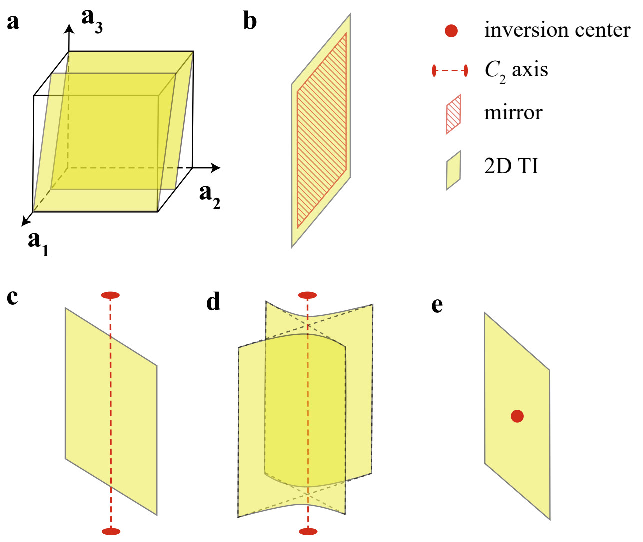

Supplementary Figure 1. In **a** we illustrate the calculation of weak invariants for a LC. The layer $(201;0)$ intersects with $\mathbf{a}_1$, $\mathbf{a}_2$, and $\mathbf{a}_3$ by 2, 0, and 1 times per unit cell, and so the weak invariants are $\delta_{\mathrm{w},1}=0$, $\delta_{\mathrm{w},2}=0$, and $\delta_{\mathrm{w},3}=1$. **b** is the LC of a mirror TCI. **c** is the LC with nontrivial C_2-rotation invariant. **e** is the LC with nontrivial inversion invariant. In **d** we show how a LC occupying the C_2 axis twice can be dimerized and moved away from the C_2 axis symmetrically, where the two 2D TIs making up the LC are represented by the blank dashed parallelograms and the dimerized configuration is colored in yellow.

$$\delta_{\mathrm{w},2}(E)=\sum_{L\in E} n_L \mod 2 \tag{10}$$

$$\delta_{\mathrm{w},3}(E)=\sum_{L\in E} l_L \mod 2 \tag{11}$$

Here E is the eLC, and L sums over all the layers in it. In the example shown in Supplementary Figure 1a, the layer's Miller indices are (201), thus the corresponding weak invariants are $\delta_{\mathrm{w},1}=0$, $\delta_{\mathrm{w},2}=0$, $\delta_{\mathrm{w},3}=1$.

The real space mirror Chern number C_{m}. In presence of mirror symmetries, 3D mirror TCIs can be got by decorating the mirror planes with 2D mirror TCIs. And, the corresponding mirror Chern numbers (in real space) is simply given by the number of times that the mirror planes are occupied by the 2D mirror TCIs. Therefore, at each mirror plane we assign a mirror Chern number, which can be calculated as

$$C_{\mathrm{m}}(E)=\sum_{L\in E} N_m^{\mathrm{o}}(L) \tag{12}$$

$$N_m^{\mathrm{o}}(L)=\begin{cases}1 & \text{if } m\in L\\ 0 & \text{otherwise}\end{cases} \tag{13}$$

for an eLC, where $m\in L$ means the mirror plane is occupied by L and $N_m^{\mathrm{o}}(L)$ is the number of times that the mirror plane is occupied by L. In the following, we refer such numbers as occupation numbers (ONs).

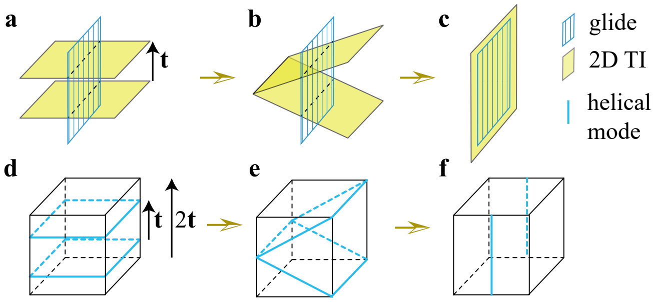

Supplementary Figure 2. In **a** and **c**, the two LCs having nontrivial hourglass invariant are plotted respectively, and in **b** the intermediate state between them is plotted. The corresponding boundary states on a square cylinder geometry are plotted in **d**-**f**. The translation symmetry on the surface is assumed to be broken.

There should be no worry about the signs of the mirror Chern numbers. For the 2D state on the mirror plane, the mirror Chern number can be defined as

$$C_{\mathrm{m}}=\frac{1}{2\pi}\int d^2\mathbf{k}\ \mathbf{n}\cdot\mathbf{\Omega}_{\mathbf{k}}^{(i)} \tag{14}$$

where $\mathbf{\Omega}_{\mathbf{k}}^{(i)}$ is the Berry curvature in the sector with mirror eigenvalue i, $\mathbf{n}$ is the normal vector of the mirror, and $\mathbf{k}$ is integrated in the 2D Brillouin zone on the mirror plane. Firstly, this definition does not depend on the choice of the normal vector. If we choose $\mathbf{n}'=-\mathbf{n}$ as the normal vector, then the $\pm i$ mirror eigenvalues interchange with each other and so the Berry curvature in i sector becomes $\mathbf{\Omega}^{(i)\prime}=\mathbf{\Omega}^{(-i)}=-\mathbf{\Omega}^{(i)}$, leading to the same C_{m}. Secondly, the mirror Chern numbers on all equivalent mirror planes in an eLC must equal to each other, since under any space group operation both $\mathbf{n}$ and $\mathbf{\Omega}^{(i)}$ transforms as pseudo vectors, i.e., vectors ignoring the inversion, and thus C_{m} transforms as a scalar. In an eLC there is at most one kind of equivalent mirror planes are occupied, therefore we can always choose proper 2D mirror TCIs such that all the corresponding mirror Chern numbers are 1.

The hourglass invariant δ_{h}. In presence of glide symmetry, a $\mathbb{Z}_2$ bulk invariant can be defined from the Wilson loop operator [1, 2]. And the nontrivial topology is manifested by the hourglass fermion on the surface preserving the glide symmetry. A fixed-point wavefunction of such a state is a simple stacking of 2D TIs along the glide vector [3], as shown in Supplementary Figure 2a. The two 2D TIs in one unit cell are connected by the glide vector, thus they can not be dimerized without breaking the glide symmetry. Correspondingly, on the surface preserving the glide symmetry the two helical modes contributed by the two 2D TIs are also connected by the glide vector and so also can not be dimerized.

Here we propose another glide-protected nontrivial LC configuration where only a single 2D TI occupies the glide

plane, as shown in Supplementary Figure 2c. This LC is nontrivial because the single 2D TI can not be trivialized symmetrically. The corresponding boundary state on a square cylinder geometry, which is infinitely long to keep the glide symmetry, hosts only two 1D helical modes (Supplementary Figure 2f) if the other translation symmetry on surface is broken. As illustrated in Supplementary Figure 2b, such a state can be symmetrically deformed to the above LC of the hourglass state where 2D TIs are stacked along the glide vector. Therefore, the three LCs plotted in Supplementary Figure 2a-c are all topologically equivalent.

With the above analysis, we conclude that each pair of layers connected by the glide vector (Supplementary Figure 2a,b) or each single layer occupying the glide plane (Supplementary Figure 2c) contributes to one hourglass mode. Due to the $\mathbb{Z}_2$ nature of the hourglass invariant, to calculate the $\delta_{\rm h}$ we should count the number of such configurations in an eLC and take the parity (even or odd) in the end. Since the minimal lattice translation along the glide vector $\mathbf{t}_\parallel$ is $2\mathbf{t}_\parallel$, the layer L in Supplementary Figure 2a,b contribute $\frac{1}{2\pi}2|\mathbf{t}_\parallel\cdot\mathbf{g}_L|$ helical modes per surface unit cell, or $\frac{1}{2\pi}|\mathbf{t}_\parallel\cdot\mathbf{g}_L|$ pairs of helical modes per surface unit cell, where $\mathbf{g}_L=m_L\mathbf{b}_1+n_L\mathbf{b}_2+l_L\mathbf{b}_3$ is the normal vector of L. Therefore, the corresponding invariant can be calculated as

$$\delta_{\rm h}(E)=\sum_{L\in E}N^{\rm o}_{m,\mathbf{t}_\parallel}(L)+N^{\rm s}_{m,\mathbf{t}_\parallel}(L)\mod 2 \tag{15}$$

$$N^{\rm o}_{m,\mathbf{t}_\parallel}(L)=\begin{cases}1 & \text{if } m\in L\\ 0 & \text{otherwise}\end{cases} \tag{16}$$

$$N^{\rm s}_{m,\mathbf{t}_\parallel}(L)=\frac{1}{2\pi}|\mathbf{t}_\parallel\cdot\mathbf{g}_L| \tag{17}$$

where m is the mirror plane, $\mathbf{t}_\parallel$ is glide vector, and $m\in L$ means that the glide plane is occupied by L. $N^{\rm o}_{m,\mathbf{t}_\parallel}(L)$ and $N^{\rm s}_{m,\mathbf{t}_\parallel}(L)$ are the glide-ON and the glide-stacking-number (glide-SN) contributed by the LC configurations in Supplementary Figure 2c and Supplementary Figure 2a,b respectively,

The rotation invariant $\delta_{\rm r}$. The TCIs protected by $C_{n=2,4,6}$-rotation are proposed very recently [4, 5]. For a given rotation axis, the corresponding $\mathbb{Z}_2$ invariant $\delta_{\rm r}$ can be defined as: 1 if the state can not be adiabatically deformed to an atomic insulator symmetrically with respect to the corresponding rotation axis, and 0 otherwise. As discussed in Supplementary Ref. [4], such states are easy to realize by layer construction. For example, as illustrated in Supplementary Figure 1c, if the C_2 axis is occupied by only one 2D TI, then this 2D TI can not be dimerized symmetrically since the minimal configuration that does not occupy the C_2 axis needs at least two 2D TIs (Supplementary Figure 1d. However, if the C_2 axis is occupied by two 2D TIs (dashed parallelograms in Supplementary Figure 1d), the 2D TIs can be dimerized symmetrically. Similarly, for the C_4 and C_6 axis, the minimal configuration occupying the axis needs only 2 and 3 2D TIs, respectively; while the minimal configuration that does not occupy the axis needs 4 and 6 2D TIs, respectively. Therefore the eLC consisting of 2 (3) 2D TIs occupying a C_4 (C_6) axis is also topologically nontrivial. (An exception is the C_3 axis, for which both the minimal occupying configuration and the minimal not-occupying configuration need 3 2D TIs.) To calculate the $\mathbb{Z}_2$ invariant of a C_n-rotation axis for a given eLC we need only to count the times that the axis is occupied

$$\delta_{\rm r}(E)=\sum_{L\in E}\frac{2}{n}N^{\rm o}_{C_n}(L)\mod 2 \tag{18}$$

$$N^{\rm o}_{C_n}(L)=\begin{cases}1 & \text{if } C_n\subset L\\ 0 & \text{otherwise}\end{cases} \tag{19}$$

where $C_n\in L$ means that the C_n-rotation axis is occupied by L and $N^{\rm o}_{C_n}(L)$ is the rotation-ON of L.

The inversion invariant $\delta_{\rm i}$. The inversion symmetry can also protect a new kind of TCI [4]. Such a state can be thought as a double of the centrosymmetric TI. Double of TI is usually considered as a trivial state since the surface Dirac nodes can be gapped, while it is proved that in presence of inversion symmetry even all the surfaces are gapped there must be a 1D helical mode on the inversion preserving boundary. Such a state can be realized by a LC occupying the inversion center (Supplementary Figure 1e). Similar with C_2-rotation-protected TCI, odd number of 2D TIs occupying the inversion center can not be trivialized without breaking the inversion while even number can be, leading to the $\mathbb{Z}_2$ classification. For each inversion center, we can assign an inversion invariant $\delta_{\rm i}$ defined as: 1 if the state can not be adiabatically deformed to an atomic limit centrosymmetrically with respect to the corresponding inversion center, and 0 otherwise. In an eLC state, the invariant $\delta_{\rm i}$ can be simply counted as the parity of the number of times that the inversion center being occupied by the layers.

$$\delta_{\rm i}(E)=\sum_{L\in E}N^{\rm o}_{i}(L)\mod 2 \tag{20}$$

$$N^{\rm o}_{i}(L)=\begin{cases}1 & \text{if } i\in L\\ 0 & \text{otherwise}\end{cases} \tag{21}$$

where $i\in L$ means that the inversion center i is occupied by L and $N^{\rm o}_i(L)$ is the inversion-ON of L.

Two new topological crystalline insulator invariants

The screw invariant $\delta_{\rm s}$. In the above we have discussed that the TCI invariant $\delta_{\rm r}$ can be defined in presence of $C_{n=2,4,6}$-rotation symmetry. A natural question is whether similar TCI invariant exits in presence of

$C_{n=2,4,6}$-screw symmetry. Suppose that the C_2-rotation axis in Supplementary Figure 1c is replaced by a C_2-screw axis, then without breaking the C_2-screw symmetry this 2D TI can not be trivialized, whereas double of the LC can be symmetrically trivialized. Similar statements also hold for C_4- and C_6-screw symmetries. Therefore a $\mathbb{Z}_2$ invariant, denoted as $\delta_{\rm s}$, can be defined when $C_{n=2,4,6}$-screw presents. On the other hand, similar with the hour-glass state, such a LC can be deformed symmetrically to another LC where 2D TIs are stacked along the screw vector (just like the LC in Supplementary Figure 2a. Similar deformations can also happen for $C_{n=4,6}$-screw-protected TCIs. The key character of the deformed LC is that there are odd number of 2D TIs stacked in each screw-vector section, i.e., $\sum_L \frac{1}{2\pi}|\mathbf{t}_\parallel \cdot \mathbf{g}_L| = 1 \mod 2$. Here L sums over the layers in the LC, $\mathbf{g}_L = m_L\mathbf{b}_1 + n_L\mathbf{b}_2 + l_L\mathbf{b}_3$ is the normal vector of L, and $\mathbf{t}_\parallel$ is the screw vector. Since the two possible LCs are topologically equivalent, to calculate $\delta_{\rm r}$ they should be equally treated. Therefore, the screw invariant can be calculated as

$$\delta_{\rm s}(E) = \sum_{L\in E} \frac{2}{n} N^{\rm o}_{C_n,\mathbf{t}_\parallel}(L) + N^{\rm s}_{C_n,\mathbf{t}_\parallel}(L) \mod 2 \quad (22)$$

$$N^{\rm o}_{C_n,\mathbf{t}_\parallel}(L) = \begin{cases} 1 & \text{if } C_n \subset L \\ 0 & \text{otherwise} \end{cases} \quad (23)$$

$$N^{\rm s}_{C_n,\mathbf{t}_\parallel}(L) = \frac{1}{2\pi}|\mathbf{t}_\parallel \cdot \mathbf{g}_L| \quad (24)$$

where $N^{\rm o}_{C_n,\mathbf{t}_\parallel}(L)$ and $N^{\rm s}_{C_n,\mathbf{t}_\parallel}(L)$ are the screw-ON and the screw-SN contributed by the two possible LC configurations, respectively.

The S_4 invariant δ_{S_4}. There are three types of symmetry elements, i.e., the point like, the line like, and the plane like elements. In the above section, all the topologies protected by line like and plane like symmetry elements have been discussed. However, the discussion about point like elements is incomplete—besides the inversion center there is another point like symmetry element, i.e., the S_4 center. It should be noticed that, other than S_6 which is equivalent with a mirror plus a C_3 rotation, S_4 center is a real point like element which can not be reduced to other type symmetry elements.

Now consider the symmetric configurations of 2D TIs occupying a S_4 center. There are two kinds of them are nontrivial. In the first configuration (Supplementary Figure 3a), only one 2D TI occupies the S_4 center and it is perpendicular to the C_2-rotation axis given by S_4^2. This configuration is nontrivial because we can not move or dimerize the only 2D TI there. In the second configuration (Supplementary Figure 3c), two 2D TIs occupy the S_4 center and they are parallel with the C_2-rotation axis given by S_4^2. As shown in Supplementary Figure 3b, there is a symmetric deformation connecting it to the first configuration, thus the second configuration is indeed equivalent with the first one. From the aspect of boundary state on a sphere geometry, the first configuration contributes one helical mode along the equator (Supplementary Figure 3d), while the second configuration contributes two helical modes along two orthogonal meridians (Supplementary Figure 3f). These two boundary states are also connected by a symmetric deformation, as shown in Supplementary Figure 3e.

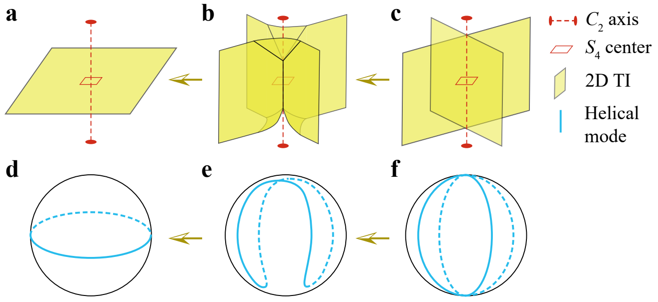

Supplementary Figure 3. S_4 invariant in LC. In **a** and **c**, we show two topologically equivalent LCs with nontrivial S_4 invariant, and in **b** we show the symmetric deformation from **c** to **a**. **d**, **f**, **e** are the corresponding boundary states on a sphere geometry.

Double of the nontrivial configuration must be trivial, because both the bulk 2D TIs and the corresponding boundary states can be dimerized without breaking the S_4 symmetry. Therefore, a $\mathbb{Z}_2$ invariant for this new TCI can be defined as: 1 if the state can not be deformed to an atomic insulator adiabatically and symmetrically with respect to the S_4 symmetry, and 0 otherwise. To calculate this invariant for an eLC, we need only to count the number of 2D TIs occupying the S_4 center

$$\delta_{S_4}(E) = \sum_{L\in E} N^{\rm o}_{S_4}(L) + \frac{1}{2}\sum_{L\in E} N^{\rm o}_{S_4^2}(L) \mod 2 \quad (25)$$

$$N^{\rm o}_{S_4}(L) = \begin{cases} 1 & \text{if } S_4 \in L \text{ and } L \perp S_4^2 \\ 0 & \text{otherwise} \end{cases} \quad (26)$$

where $N^{\rm o}_{S_4}(L)$ is the S_4-ON of L and $N^{\rm o}_{S_4^2}(L)$ is the C_2-rotation-ON of L. A trick used here is to count the contribution of each 2D TI in the second configuration as $\frac{1}{2}$ such that the two 2D TIs in total contribute 1.

Surface states of topological crystalline insulators

Here we present a short summary of the topological surface states corresponding to the TCI invariants introduced above (Supplementary Figure 4).

The TCI invariants can be roughly classified into two classes by whether the protecting symmetry is point-group-like or not. The point-group-like symmetries can be further classified into three cases by the dimensions of symmetry elements. (i) The point-like symmetries, i.e., the inversion and S_4. The corresponding invariants

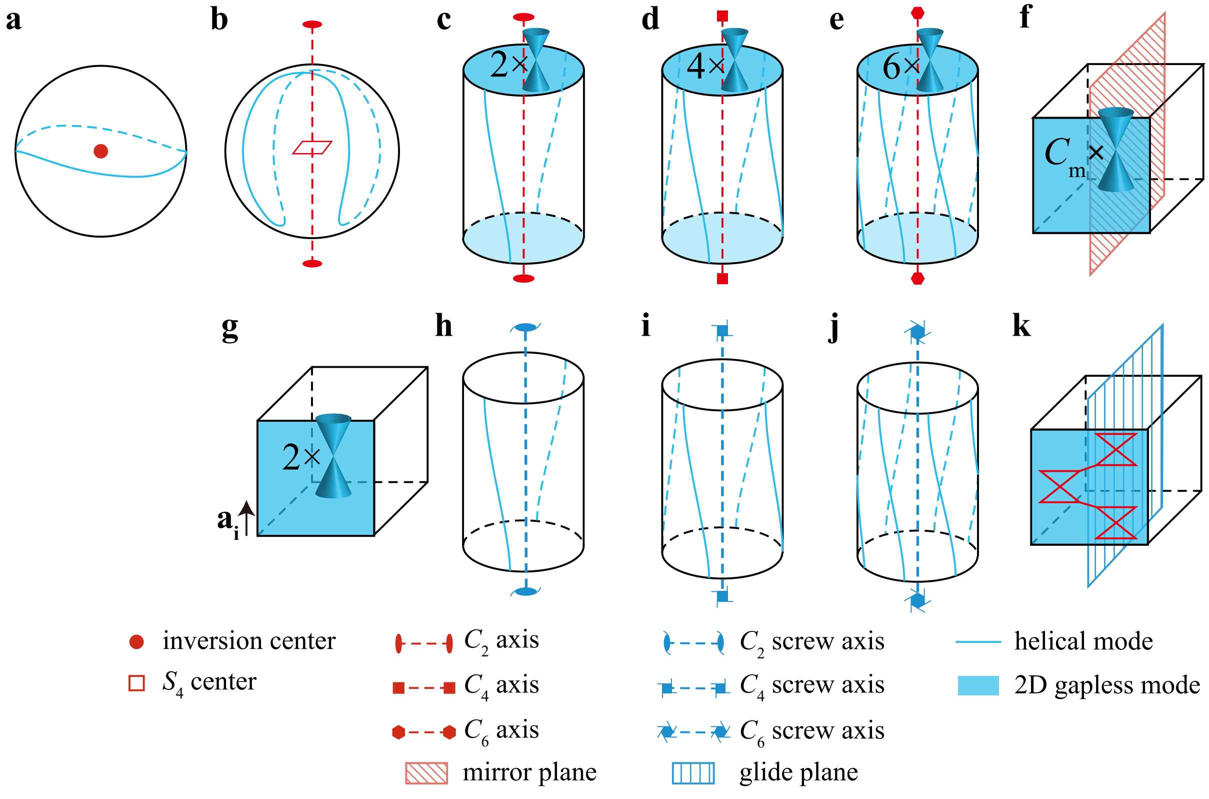

Supplementary Figure 4. Surface states of topological crystalline insulators. **a** The surface state of inversion-protected TCI. **b** The surface state of S_4-protected TCI. **c-e** The surface states of $C_{n=2,4,6}$-rotation-protected TCI. **f** The surface state of mirror TCI. **g** The surface state of weak TI, here $\mathbf{a}_i$ represents the translation symmetry protecting the weak index. **h-j** The surface states of $C_{n=2,4,6}$-screw-protected TCI. **k** The surface state of hourglass TCI.

are $\delta_{\rm i}$ and δ_{S_4}, and the correspinding anomalous surface states are 1D helical modes, which can be detected by putting the TCIs on finite geometries preserving the point-like symmetries, as shown in Supplementary Figure 4a,b. Under the point-like symmetries the 1D modes transform to themselves such that without breaking the point-like symmetries the 1D modes can not be removed. (See Supplementary Ref. [4] for more details of surface states of the inversion and S_4 invariants, respectively.) (ii) The line-like symmetries, i.e., the $C_{n=2,4,6}$-rotation. The correspinding invariant is $\delta_{\rm r}$. And the corresponding anomalous surface state consists of two parts: the n (modulo $2n$) 2D Dirac nodes on surface preserving the rotation symmetry and the n (modulo $2n$) 1D helical modes on finite geometry preserving the rotation symmetry. Both the two kinds of surface states can be detected by putting the TCI on a cylinder geometry preserving the rotation symmetry, where on the top surface are the 2D Dirac nodes and on the side surface are the 1D helical modes, as shown in Supplementary Figure 4c-e. (See Supplementary Ref. [4] and [5] for the 2D and 1D surface states, respectively.) (iii) The plane-like symmetry, i.e., the mirror. The correspinding invariant is the mirror Chern number $C_{\rm m}$, and the correspinding anomalous surface state is the 2D state with $C_{\rm m}$ Dirac nodes on surface preserving the mirror symmetry, as shown in Supplementary Figure 4f [6, 7].

The non-point-group symmetries that can protect TCI invariants can also be classified into three cases. (i) The translation symmetry. The correspinding invariant is the weak TI index, i.e., $\delta_{\rm w}$, and the corresponding anomalous surface state is the 2D state with even number of Dirac nodes on surface preserving the translation symmetry, as shown in Supplementary Figure 4g [8]. (ii) The $C_{n=2,4,6}$-screw symmetry. The corresponding invariant is $\delta_{\rm s}$ and the correspinding anomalous surface states are the 1D helical modes on cylinder geometry preserving the screw symmetry. As discussed above in this Supplementary Note, screw-protected TCI can have identical LCs with the rotation-protected TCI, where the $C_{n=2,4,6}$-screw axis is occupied by $n/2$ times. Putting such LCs on a cylinder geometry, we get the 1D helical modes shown in Supplementary Figure 4h-j. The only difference with rotation-protected TCI is the absence of 2D gapless surface state because the screw symmetry must be broken on any 2D surface. (iii) The glide symmetry. The corresponding invariant is $\delta_{\rm h}$, and the corresponding anomalous surface state is the 2D hourglass fermion on surface preserving the glide symmetry, as shown in Supplementary Figure 4k [1].

Generating all symmetry elements in a space group

In this section we give the general strategy to generate all the symmetry elements in a given space group. When we talk about the symmetry element associated with a symmetry operation, we mean (i) the center about which the operation takes place, which is given by the invariant geometry object under the operation, and (ii) the type of the corresponding operation, i.e., inversion, rotation, screw, etc. A general space group operation can be written as $\{p|\mathbf{t}+\mathbf{R}\}$ and can be interpreted as a point group operation centered at $\mathbf{x}$ followed by a translation $\mathbf{t}_{\|}$

$$p\mathbf{r}+\mathbf{t}+\mathbf{R}=p\left(\mathbf{r}-\mathbf{x}\right)+\mathbf{x}+\mathbf{t}_{\|} \tag{27}$$

where $\mathbf{x}$ and $\mathbf{t}_{\|}$ are determined by

$$\left(1-p\right)\mathbf{x}+\mathbf{t}_{\|}=\mathbf{t}+\mathbf{R} \tag{28}$$

$$p\mathbf{t}_{\|}=\mathbf{t}_{\|} \tag{29}$$

Beware that $\mathbf{x}$ can be a point, a line, or a plane. Symmetry element got from nonzero $\mathbf{R}$ is called "additional symmetry elements" [9], which in general has different location and even different type with the original symmetry element given by $\{p|\mathbf{t}\}$, for example, a rotation axis may change to a screw axis, and a mirror plane may change to a glide plane. Due to the translation symmetry, the TCI invariants defined on symmetry elements locating in different cells must be identical with each other. Therefore, a complete TCI invariant set should include only the TCI invariants defined in the home cell.

The momentum space mirror Chern numbers

As discussed above in this Supplementary Note, the real space mirror Chern number is easy to calculate for LCs. However, people usually understand the mirror Chern numbers in momentum space. Thus we devote this section to translate the real space mirror Chern numbers to momentum space Mirror Chern numbers.

Consider a mirror operation $\{m|\mathbf{t}\}$, where $\mathbf{t}$ is perpendicular with the mirror. By applying Supplementary Eq. (28) and (29), we get that the mirror or glide plane generated from $\{m|\mathbf{t}+\mathbf{R}\}$ satisfies

$$d=\frac{1}{2\pi}\mathbf{x}\cdot\mathbf{g}_m=\frac{1}{4\pi}\mathbf{t}\cdot\mathbf{g}_m+\frac{1}{4\pi}\mathbf{R}\cdot\mathbf{g}_m \tag{30}$$

$$\mathbf{t}_{\|}=\mathbf{R}-\left(\mathbf{R}\cdot\hat{\mathbf{g}}_m\right)\hat{\mathbf{g}}_m \tag{31}$$

Here $\mathbf{g}_m$ is the normal vector of the mirror, i.e., the minimal reciprocal vector perpendicular with the mirror, $\hat{\mathbf{g}}_m$ is the unit vector along $\mathbf{g}_m$, d is the position of the mirror or glide plane, and $\mathbf{t}_{\|}$ is the translation parallel with the glide plane (zero for mirror). Since $\frac{1}{2\pi}\mathbf{R}\cdot\mathbf{g}_m$ must be an integer, $\frac{1}{4\pi}\mathbf{R}\cdot\mathbf{g}_m$ is either an integer or a half-integer.

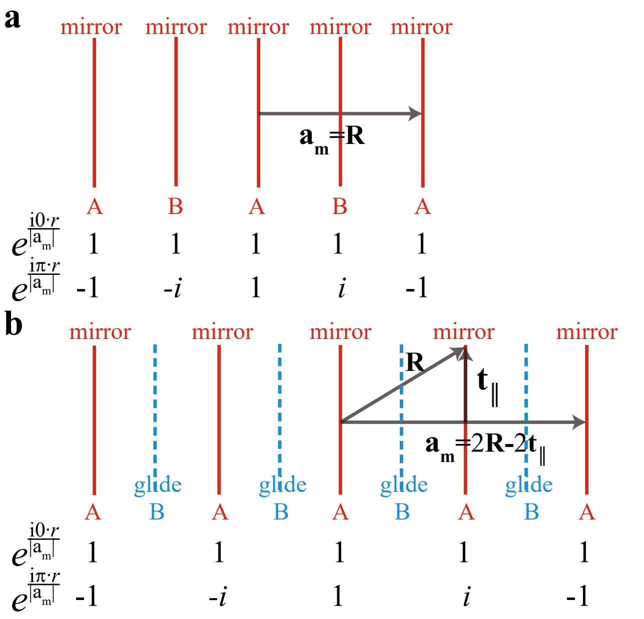

Supplementary Figure 5. The mirror plane generated from $\{m|\mathbf{t}\}$ is denoted as A, and the mirror or glide plane generated from $\{m|\mathbf{t}+\mathbf{R}\}$ with $\frac{1}{4\pi}\mathbf{R}\cdot\mathbf{g}_m=\frac{1}{2}$ (modulo 1) is denoted as B. The $\mathbf{R}$ plotted is the shortest lattice vector giving $\frac{1}{2\pi}\mathbf{R}\cdot\mathbf{g}_m=\frac{1}{2}$. In **a** B is a also a mirror plane, and the minimal lattice vector perpendicular with A is $\mathbf{a}_m=\mathbf{R}$. In **b** B is a glide plane, and the minimal lattice vector perpendicular with A is $\mathbf{a}_m=2\mathbf{R}-2\mathbf{t}_{\|}$, where $\mathbf{t}_{\|}$ is the glide vector of B. In the bottom of **a** and **b**, we list the phase factors in the Fourier transformations along $\mathbf{a}_m$ for $k=0$ and $k=\pi$.

Due to the translation symmetry, all $\mathbf{R}$ giving integer $\frac{1}{4\pi}\mathbf{R}\cdot\mathbf{g}_m$ correspond to the original mirror plane A, and all $\mathbf{R}$ giving half-integer $\frac{1}{4\pi}\mathbf{R}\cdot\mathbf{g}_m$ correspond to the new mirror or glide plane B. The two cases that B is a mirror plane or a glide plane, which depend on the value of $\mathbf{t}_{\|}$, are very different. For the first case, we have two real space mirror Chern numbers for A and B, respectively. While, for the second case, we have only one real space mirror Chern number for A. For the first case, as shown in Supplementary Figure 5a, the minimal lattice vector perpendicular to the mirror, denoted as $\mathbf{a}_m$, is just the shortest $\mathbf{R}$ giving $\frac{1}{4\pi}\mathbf{R}\cdot\mathbf{g}_m=\frac{1}{2}$, and in one period there are two distinct mirror planes. Here we choose the mirror operation on A to define the mirror eigenvalue. Applying the Fourier transformation along $\mathbf{a}_m$, we find that for the two mirror-invariant momenta $k=0,\pi$ A subsystem has the same mirror eigenvalues while B subsystem has two converse mirror eigenvalues. Therefore, the mirror Chern number at $k=0$ and $k=\pi$ is given by

$$C_{\mathrm{m},0}=C_{\mathrm{m,A}}+C_{\mathrm{m,B}} \tag{32}$$

$$C_{\mathrm{m},\pi}=C_{\mathrm{m,A}}-C_{\mathrm{m,B}} \tag{33}$$

respectively, where $C_{\mathrm{m,A}}$ and $C_{\mathrm{m,B}}$ are the two real space mirror Chern numbers. For the second case, as shown

in Supplementary Figure 5b, the minimal lattice vector perpendicular to the mirror is given by $2\mathbf{R} - 2\mathbf{t}_{\parallel}$, where $\mathbf{R}$ is the shortest lattice vector giving $\frac{1}{4\pi}\mathbf{R} \cdot \mathbf{g}_m = \frac{1}{2}$, and in one period there are two same mirror planes. Applying the Fourier transformation, we get

$$C_{\mathrm{m},0} = 2C_{\mathrm{m,A}} \tag{34}$$

$$C_{\mathrm{m},\pi} = C_{\mathrm{m,A}} - C_{\mathrm{m,A}} = 0 \tag{35}$$

where $C_{\mathrm{m},\pi}$ is always zero.

Supplementary Note 2. Convention dependence of topological invariants

There may be more than one symmetry elements that are identical with each other but locate at different positions. For example, for any centrosymmetric SG there are eight inversion centers in a unit cell. In the following we call such symmetry elements as noncoincident identical symmetry elements. In general case, noncoincident identical symmetry elements are given by $\{p|\mathbf{t}+\mathbf{R}\}$, where p and $\mathbf{t}$ are fixed and $\mathbf{R}$ goes over lattice vectors that merely change the position of the symmetry operation (Supplementary Note 1). On each of the noncoincident symmetry elements we should assign a TCI invariant, e.g., eight inversion invariants for the eight inversion centers. However, after an exhaustive enumeration over 230 SGs, we find that the TCI invariants defined on noncoincident identical symmetry elements are not independent with each other. For glide, rotation, inversion, screw, and S_4 symmetries, one of the invariants can be uniquely determined from another and the three weak invariants. Therefore, among each kind of noncoincide identical symmetry elements it is enough to choose one to define the invariant. The convention for this choice in given in Supplementary Note 7.

Here we take SG #2 ($P\bar{1}$) as an example to show this convention dependence. As shown in Supplementary Figure 8a, if we choose the inversion center at origin to define the inversion invariant, then E_1, E_2, E_3 have nontrivial invariants, and E_4 has trivial invariant. While, if we choose the inversion center at $(0,0,\frac{1}{2})$ to define the inversion invariant, then E_1, E_2, E_4 have nontrivial invariants, and E_3 has trivial invariant.

It should be emphasized that physically nontrivial topology should not depend on this convention. In other words, the topology is physical as long as the invariants defined on every of the noncoincident symmetry elements are all nontrivial. Taking SG $P\bar{1}$ as example, none of E_1, E_2, E_3, E_4 is physically nontrivial, because in any of them there are four empty inversion centers around which the 2D TIs can be dimerized symmetrically by breaking the inversion symmetry on the other four centers. On the other hand, a physical nontrivial state can be realized as $E_3 \otimes E_4$, where all the eight inversion centers are occupied and so $\delta_{\mathrm{i}} = 1$ does not depend on the choice of inversion center. In the ultimate presentation of our results (Supplementary Table 7), convention-independent $\mathbb{Z}_2$ invariants will be marked.

Supplementary Note 3. Generating all nonequivalent elementary layer constructions

Nonequivalent layers

The topology of an eLC is completely determined by the little group, the weak invariants, the ONs, and the SNs of *any* layer in it, because by applying the coset representatives of the little group, we can reproduce weak invariants, ONs, and SNs of all the other layers in the eLC and so reproduce the topology of the eLC. Let us denote a layer in an eLC as L, its little group as $\mathcal{S}(L)$, and the corresponding coset representatives as g_0, g_1, $\cdots$. The eLC consists of $\{g_0 L, g_1 L, \cdots\}$. Firstly, according to Supplementary Eq. (9) to (11), the weak invariants of $g_i L$ are just the parities of $g_i L$'s Miller indices and so are determined by the rotation matrix of g_i (an integer matrix in the basis of lattice vectors) and the parities of L's Miller indices (L's weak invariants). Secondly, the ONs (SNs) on any symmetry element e of $g_i L$ can be calculated as $N_e^{\rm o}(g_i L) = N^{\rm o}_{g_i^{-1} e g_i}(L)$ ($N_e^{\rm s}(g_i L) = N^{\rm s}_{g_i^{-1} e g_i}(L)$). In the following, we will say two layers are *equivalent* with each other if the eLCs generated from them are topologically equivalent.

Here we give a sufficient condition for equivalence: two layers L and L' must be equivalent with each other if (i) $\mathcal{S}(L) = \mathcal{S}(L')$, (ii) $\delta_{{\rm w},i=1,2,3}(L) = \delta_{{\rm w},i=1,2,3}(L')$, (iii) $N_e^{\rm o}(L) = N_e^{\rm o}(L')$ for e as mirror (glide) planes, C_2-rotation (screw) axes, inversion centers, and S_4 centers, and (iv) $n_{\mathbf{t}_\parallel} N_e^{\rm s}(L) = n_{\mathbf{t}_\parallel} N_e^{\rm s}(L') \mod 2$ for e as glide planes and $C_{n=2,4,6}$-screw axes, where $n_{\mathbf{t}_\parallel}$ is the minimal multiplier that makes $\mathbf{t}_\parallel$ a lattice vector, and $\mathbf{t}_\parallel$ is the glide or screw vector. For example, for glide and C_2-screw $n_{\mathbf{t}_\parallel} = 2$. Now let us prove the sufficient condition. Firstly, according to (i)-(iii) and the discussion in last paragraph, the mirror, C_2-rotation, inversion, S_4 invariants, and weak invariants of eLC(L) and eLC(L') must equal to each other. Secondly, since a single layer's ON on a C_4-rotation (C_6-rotation) axis equals to its ON on the C_2-rotation axis given by C_4^2 (C_6^3), according to (i) and (iii) the C_4-rotation (C_6-rotation) invariants of eLC(L) and eLC(L') must equal to each other. Thirdly, for e as glide planes two cases should be discussed separately and in both cases the hourglass invariants of eLC(L) and eLC(L') equal to each other. In the first case we assume $N_g^{\rm o}(L) = N_g^{\rm o}(L') \neq 0$, where g is the glide operation. As both L and L' occupy the glide plane, we have $N_g^{\rm s}(L) = N_g^{\rm s}(L') = 0$. According to (i) the hourglass invariants of eLC(L) and eLC(L') must equal to each other. In the second case we assume $N_g^{\rm o}(L) = N_g^{\rm o}(L') = 0$, which implies both L and L' are not invariant under the glide operation. However, we can introduce glide-invariant "composite layers" as $\tilde{L} = L \otimes gL$ and $\tilde{L}' = L' \otimes gL'$, whose little group is given by $\mathcal{S}(L) \oplus g\mathcal{S}(L) = \mathcal{S}(L') \oplus g\mathcal{S}(L')$. On one hand, the discussion in last paragraph also applies for these "composite layers" and so the topologies of the eLCs are uniquely determined by the little groups, weak invariants, ONs, and SNs of the "composite layers". On the other hand, we have

$$N_g^{\rm s}(\tilde{L}) = \frac{1}{2\pi}(|\mathbf{t}_\parallel \cdot \mathbf{g}_L| + |\mathbf{t}_\parallel \cdot g\mathbf{g}_L|) = 2\frac{1}{2\pi}|\mathbf{t}_\parallel \cdot \mathbf{g}_L| = 2N_g^{\rm s}(L) \tag{36}$$

and $N_g^{\rm s}(\tilde{L}') = 2N_g^{\rm s}(L')$ according to Supplementary Eq. (17). Therefore, the hourglass invariants of eLC(L) and eLC(L') on g should equal to each other as long as (i) and (iv) are satisfied. Fourthly, the proof for $C_{n=2,4,6}$-screw axes is parallel with the proof for glide plane, where a "composite layer" should be introduced if the layer does not occupy the screw axis.

To exhaust all nonequivalent eLCs, we need only to exhaust all nonequivalent layers, which is done in two steps—in the following two sections we first give the strategy to generate all nonequivalent layers with same Miller indices, and then the strategy to exhaust all Miller indices.

Nonequivalent layers with same Miller index

In this section, we will prove that for a layer with given Miller indices $(mnl;d)$ there are only finite cases of d to give nonequivalent layers, and give the algorithm to find these cases. Since the weak invariants and SNs do not depend on d, in the following two d's will be identified as equivalent if and only if the layer given by them have same little group and ONs on mirror (glide) planes, C_2-rotation (screw) axes, inversion centers, and S_4 centers.

Let us start with the discussion of little group. For convenience, we set $\mathbf{g} = m\mathbf{b}_1 + n\mathbf{b}_2 + l\mathbf{b}_3$. Then the little group $\mathcal{S}(mnl;d)$ consists of two kinds of operations, (i) the operations that leave $\mathbf{g}$ invariant

$$\mathcal{S}_{\rm I}(mnl) = \Big\{ \{p|\mathbf{t}+\mathbf{R}\} \in \mathcal{G} \big| p\mathbf{g} = \mathbf{g} \quad \frac{1}{2\pi}\mathbf{t}\cdot\mathbf{g} = 0 \bmod 1 \Big\} \tag{37}$$

and (ii) the operations that reverse the direction of $\mathbf{g}$

$$\mathcal{S}_{\rm II}(mnl;d) = \Big\{ \{p|\mathbf{t}+\mathbf{R}\} \in \mathcal{G} \big| p\mathbf{g} = -\mathbf{g} \quad \frac{1}{4\pi}(\mathbf{t}+\mathbf{R})\cdot\mathbf{g} = d \bmod \frac{1}{2} \Big\} \tag{38}$$

The additional conditions in the braces come from the simultaneous layer equations (Supplementary Eq. (1)) before and after the operation, i.e.,

$$\mathbf{r}\cdot\mathbf{g} = 2\pi d \mod 2\pi \tag{39}$$

$$(p\mathbf{r}+\mathbf{t}+\mathbf{R})\cdot\mathbf{g} = 2\pi d \mod 2\pi \tag{40}$$

Apparently, the dependence of $\mathcal{S}\,(mnl;d)$ on d comes from the conditions in $\mathcal{S}_{\rm II}$, which require the layer to locate at some special positions. To make these conditions more explicit, we simply list all the special positions as

$$\mathcal{D}'_{mnl} = \left\{ d \bmod 1 \middle| d = \frac{\mathbf{t}\cdot\mathbf{g}}{4\pi}, \left(\frac{\mathbf{t}\cdot\mathbf{g}}{4\pi}+\frac{1}{2}\right) \quad \forall\,\{p|\mathbf{t}\}\in\mathcal{G} \quad p\mathbf{g}=-\mathbf{g} \right\} \tag{41}$$

where we only take one of the points that coincide, and $\frac{1}{4\pi}\mathbf{R}\cdot\mathbf{g}$ is omitted because it is either an integer or a half-integer. Then, for any $d\in\mathcal{D}'$, $\mathcal{S}_{\rm II}(mnl;d)$ can be uniquely determined, and for any $d\notin\mathcal{D}'$ there must be $\mathcal{S}_{\rm II}\,(mnl;d)=\emptyset$. Use d_0 to represent the general point not in $\mathcal{D}'$, the full classification of d from 0 to 1 can be formally written as

$$\mathcal{D}_{mnl} = \{d_0\}\cup\mathcal{D}'_{m_L n_L l_L} \tag{42}$$

Here each entry of $\mathcal{D}_{mnl}$ can be thought as a Wyckoff position of the 1D SG projected from the 3D SG along the direction specified by the Miller indices, and, each entry of $\mathcal{D}_{mnl}$ is associated with a little group $\mathcal{S}(mnl;d)$.

Now we prove that, not only the little group but also the ONs for each $d\in\mathcal{D}_{mnl}$ can be determined. They include ONs for (i) inversion centers, (ii) C_2-rotation (screw) axes perpendicular with $\mathbf{g}$, (iii) mirror (glide) planes perpendicular with $\mathbf{g}$, and (iv) S_4 centers with S_4^2 parallel with $\mathbf{g}$. Notice that operations that contribute to Supplementary Eq. (41) can only be (i) inversion, (ii) C_2-rotation (screw) perpendicular with $\mathbf{g}_L$, (iii) mirror (glide) perpendicular with $\mathbf{g}_L$, (iv) S_4 with S_4^2 parallel with $\mathbf{g}_L$, corresponding to the the four kinds of ONs. According to Supplementary Eq. (28) and (29), d in Supplementary Eq. (41) is nothing but the possible positions of the corresponding symmetry elements. To be specific, for the symmetry element given by $\{p|\mathbf{t}+\mathbf{R}\}$, project both sides of Supplementary Eq. (28) on $\mathbf{g}$, we get its position as

$$\frac{1}{2\pi}\mathbf{x}\cdot\mathbf{g} = \frac{1}{4\pi}\left(\mathbf{t}+\mathbf{R}\right)\cdot\mathbf{g} \tag{43}$$

which equals to either $\frac{1}{4\pi}\mathbf{t}\cdot\mathbf{g}$ or $\frac{1}{4\pi}\mathbf{t}\cdot\mathbf{g}+\frac{1}{2}$ (modulo 1). Therefore, all the possible positions of these symmetry elements have been included in $\mathcal{D}_{mnl}$, and for each $d\in\mathcal{D}_{mnl}$, the four kinds of ONs can be calculated by checking whether the corresponding position (Supplementary Eq. (43)) coincides with d. The trivial case where all the ONs are zero is given by $d=d_0$.

Exhausting Miller indices

In this section, we will prove that to generate all nonequivalent eLCs only a finite number of Miller indices around $(0,0,0)$ need to be considered, and give the concrete algorithm to find these Miller indices.

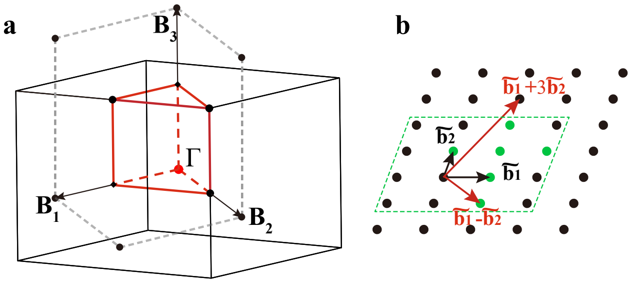

Supplementary Figure 6. In **a** is plotted the irreducible domain, which is spanned by the three reciprocal vectors $\mathbf{B}_1$, $\mathbf{B}_2$, and $\mathbf{B}_3$ and can be thought as the extension of the irreducible Brillouin zone (plotted by the red lines) to infinity. In general case, a reciprocal lattice in the irreducible domain can be expanded by $\mathbf{B}_{1/2/3}$ with *positive* integers or fractions. In **b** we show the 6 reciprocal lattices (green circles) that need to be considered on a high symmetry plane for the purpose of generating nonequivalent eLCs, where $\tilde{\mathbf{b}}_1$ and $\tilde{\mathbf{b}}_2$ are the primitive bases on this plane. For example, all the eLCs generated from $\tilde{\mathbf{b}}_1+3\tilde{\mathbf{b}}_2$ can also be generated from $\tilde{\mathbf{b}}_1-\tilde{\mathbf{b}}_2$.

Two sets of Miller indices (mnl) $(m'n'l')$ are called to be *equivalent* with each other if one of them can be transformed from another by a SG operation, i.e.,

$$\exists\,\{p|\mathbf{t}\}\in\mathcal{G}\quad s.t.\quad \mathbf{g}=\pm p\mathbf{g}' \tag{44}$$

where $\mathbf{g}=m\mathbf{b}_1+n\mathbf{b}_2+l\mathbf{b}_3$ and $\mathbf{g}'=m'\mathbf{b}_1+n'\mathbf{b}_2+l'\mathbf{b}_3$. Based on the equivalent relation we introduce the concept of irreducible domain of reciprocal lattice—the minimal subset of reciprocal lattice from which the full reciprocal lattice can be reproduced by equivalence relation (Supplementary Eq. (44)). The irreducible domain can be thought as the extension of the irreducible Brillouin zone to infinity, as shown in Supplementary Figure 6a. The irreducible domain consists of high symmetry directions (edges), high symmetry planes (surfaces), and general points (bulk). Similar with the irreducible Brillouin zone, any reciprocal vector outside the irreducible domain can be transformed from a vector in it by a SG operation. Thus to cover all the nonequivalent eLCs, we need only to enumerate all the acceptable Miller indices in the irreducible domain. Due to the coprime condition of Miller indices, along an edge only the shortest vector give acceptable Miller indices. While in a surface or the bulk of the irreducible domain there are infinite acceptable Miller indices. Here we will prove that even in the latter case only finite Miller indices around (000) need to be considered.

We firstly give the proof and algorithm for Miller indices in a surface of the irreducible domain. As shown in Supplementary Figure 6b, $\tilde{\mathbf{b}}_1$ and $\tilde{\mathbf{b}}_2$ are chosen as the primitive bases in the surface such that any reciprocal vector in the surface can be expanded on them with integer coefficients

$$\mathbf{g}=\tilde{m}\tilde{\mathbf{b}}_1+\tilde{n}\tilde{\mathbf{b}}_2 \tag{45}$$

A layer on the surface $(\tilde{m}\tilde{n};d)$ is given by a pair of coprime integers $\tilde{m},\tilde{n}$ and a position d. As discussed in the above two sections, the relevant properties of a layer includes (i) the little group, (ii) the weak invariants, (iii) the ONs on inversion centers, C_2-rotation (screw) axes, mirror (glide) planes, and S_4 centers, and (iv) the multiples of glide-SNs and screw-SNs, i.e,

$$n_{\mathbf{t}_\parallel}\frac{1}{2\pi}\mathbf{t}_\parallel\cdot\left(\tilde{m}\tilde{\mathbf{b}}_1+\tilde{n}\tilde{\mathbf{b}}_2\right)\quad\text{mod }2 \tag{46}$$

where $\mathbf{t}_\parallel$ is the glide or screw vector and $n_{\mathbf{t}_\parallel}$ is the minimal multiplier that makes $\mathbf{t}_\parallel$ a lattice vector. Now we prove that, for any integers r and s if $\tilde{m}-4r$ and $\tilde{n}-4s$ are coprime the four relevant properties of the layer $(\tilde{m}-4r,\tilde{n}-4s;d)$ are identical with the $(\tilde{m}\tilde{n};d)$'s. Firstly, as we count $\tilde{m}\tilde{\mathbf{b}}_1+\tilde{n}\tilde{\mathbf{b}}_2$ as a general vector in the Miller surface, the little group should only consist of operations that leave both $\tilde{\mathbf{b}}_1$ and $\tilde{\mathbf{b}}_2$ invariant or reverse, i.e.,

$$\mathcal{S}\left(\tilde{m}\tilde{n};d\right)=\mathcal{S}_{\mathrm{I}}\left(\tilde{m}\tilde{n}\right)\cup\mathcal{S}_{\mathrm{II}}\left(\tilde{m}\tilde{n};d\right) \tag{47}$$

$$\mathcal{S}_{\mathrm{I}}\left(\tilde{m}\tilde{n}\right)=\Big\{\{p|\mathbf{t}+\mathbf{R}\}\in\mathcal{G}\big|p\tilde{\mathbf{b}}_1=\tilde{\mathbf{b}}_1,\ p\tilde{\mathbf{b}}_2=\tilde{\mathbf{b}}_2 \\ \frac{1}{2\pi}\mathbf{t}\cdot\left(\tilde{m}\tilde{\mathbf{b}}_1+\tilde{n}\tilde{\mathbf{b}}_2\right)=0\text{ mod }1\Big\} \tag{48}$$

$$\mathcal{S}_{\mathrm{II}}\left(\tilde{m}\tilde{n};d\right)=\Big\{\{p|\mathbf{t}+\mathbf{R}\}\in\mathcal{G}\big|p\tilde{\mathbf{b}}_1=-\tilde{\mathbf{b}}_1,\ p\tilde{\mathbf{b}}_2=-\tilde{\mathbf{b}}_2 \\ \frac{1}{4\pi}\mathbf{t}\cdot\left(\tilde{m}\tilde{\mathbf{b}}_1+\tilde{n}\tilde{\mathbf{b}}_2\right)=d\text{ mod }\frac{1}{2}\Big\} \tag{49}$$

Operation in $\mathcal{S}_{\mathrm{I}}$ can only be mirror or glide, and operation in $\mathcal{S}_{\mathrm{II}}$ can only be inversion, C_2-rotation, or C_2-screw. Thus $\mathbf{t}$ in Supplementary Eq. (48) and (49) must be either a lattice vector or half of a lattice vector, which implies

$$\frac{1}{2\pi}\mathbf{t}\cdot\left(4r\tilde{\mathbf{b}}_1+4s\tilde{\mathbf{b}}_2\right)=0\quad\text{mod }1 \tag{50}$$

$$\frac{1}{4\pi}\mathbf{t}\cdot\left(4r\tilde{\mathbf{b}}_1+4s\tilde{\mathbf{b}}_2\right)=0\quad\text{mod }\frac{1}{2} \tag{51}$$

Then it follows that $\mathcal{S}\left(\tilde{m}\tilde{n};d\right)=\mathcal{S}\left(\tilde{m}-4r,\tilde{n}-4s;d\right)$. Secondly, as $4r$ and $4s$ does not change any parities of the Miller indices, there must be $\delta_{\mathrm{w},i=1,2,3}\left(\tilde{m}\tilde{n};d\right)=\delta_{\mathrm{w},i=1,2,3}\left(\tilde{m}-4r,\tilde{n}-4s;d\right)$. Thirdly, since $\tilde{m}\tilde{\mathbf{b}}_1+\tilde{n}\tilde{\mathbf{b}}_2$ is a general vector in the Miller surface, the symmetry elements occupied by $(\tilde{m}\tilde{n};d)$ can only be inversion centers, C_2-rotation axes, or C_2-screw axes, as given by

$$\mathcal{E}\left(\tilde{m}\tilde{n};d\right)=\Big\{\{p|\mathbf{t}+\mathbf{R}\}\in\mathcal{G}\big|p\tilde{\mathbf{b}}_1=-\tilde{\mathbf{b}}_1,\ p\tilde{\mathbf{b}}_2=-\tilde{\mathbf{b}}_2, \\ \frac{1}{4\pi}\left(\mathbf{t}+\mathbf{R}\right)\cdot\left(\tilde{m}\tilde{\mathbf{b}}_1+\tilde{n}\tilde{\mathbf{b}}_2\right)=d\text{ mod }1\Big\} \tag{52}$$

where $\mathbf{t}$ is either a lattice vector or half of a lattice vector. ONs on these occupied symmetry elements are 1, and the ONs on all other symmetry elements are 0. Then it follows that $\mathcal{E}\left(\tilde{m}\tilde{n};d\right)=\mathcal{E}\left(\tilde{m}-4r,\tilde{n}-4s;d\right)$ due to the relation

$$\frac{1}{4\pi}\left(\mathbf{t}+\mathbf{R}\right)\cdot\left(4r\tilde{\mathbf{b}}_1+4s\tilde{\mathbf{b}}_2\right)=0\quad\text{mod }1 \tag{53}$$

Fourthly, the multiples of glide-SNs or screw-SNs (Supplementary Eq. (46)) of $(\tilde{m}\tilde{n};d)$ and $(\tilde{m}-4r,\tilde{n}-4s;d)$ must also equal to each other for similar reason. In the end, we show that, for any coprime $\tilde{m},\tilde{n}$ their correspondences $\tilde{m}',\tilde{n}'$ in the interval $[-1,2]$ got by $\tilde{m}'=\tilde{m}-4r$ and $\tilde{n}'=\tilde{n}-4s$ must also be coprime. All the possible non-coprime pairs of $\tilde{m}',\tilde{n}'$ can be $(0,2)$, $(2,0)$, $(2,2)$, however, if $\tilde{m}',\tilde{n}'$ have the common divisor 2 then $\tilde{m},\tilde{n}$ must also have the common divisor 2, which contradicts with the presumption. Therefore, the conclusion is that to generate all nonequivalent eLCs from the Miller indices within a surface of the irreducible domain we need only to consider the reciprocal vectors $\tilde{m}\tilde{\mathbf{b}}_1+\tilde{n}\tilde{\mathbf{b}}_2$ with $\tilde{m},\tilde{n}=-1,0,1,2$, as shown by the dashed green box in Supplementary Figure 6b. After removing the parallel vectors, only the six vectors marked as green in Supplementary Figure 6b need to be considered.

The discussion for the general Miller indices in the bulk of the irreducible domain is much more simple. In this case the only possible operation in the little group is inversion and the only possible occupied symmetry element is inversion center. As we take the convention where the inversion center is put at the origin, the two layers $(mnl;d)$ $(m-2r,n-2s,l-2t;d)$ have same little group and same inversion-ONs. As $2r$, $2s$, $2t$ are even numbers, they also have same weak invariants, same glide-SNs, and same screw-SNs (Supplementary Eq. (46)). Therefore, in the bulk of the irreducible domain, we need only to consider $m\mathbf{b}_1+n\mathbf{b}_2+l\mathbf{b}_3$ with $m,n,l=0,1$.

Supplementary Note 4. Symmetry-based indicators

In Supplementary Ref. [10, 11], a complete theory of symmetry-based indicator (SI) has been developped, which in principle is all we can know about band topology from symmetry eigenvalues. However, the explicit expressions of these SI are absent in the original work. Here we give explicit formulae for *all* the SI. In the rest of this paper, these formulae are referred as Fu-Kane-like formulae.

Symmetry-based indicators

For a system with given SG, its band representation (BR) for a group of selected bands (the occupied bands for example) can be written as an integer vector

$$\mathrm{BR} = \left[n\left(\xi_1^{K_1}\right), n\left(\xi_2^{K_1}\right)\cdots, n\left(\xi_1^{K_2}\right)\cdots\right]^T \tag{54}$$

where each entry gives the number of a irreducible representation (irrep) ξ at a momentum K. On one hand, all the compatibility relation allowed BRs form a linear space, whose bases can be written as

$$\{B_1, B_2\cdots B_D\} \tag{55}$$

On the other hand, all the BRs that can be generated from symmetric Wannier functions, i.e., the BRs of atomic insulators, also form a linear space, with bases

$$\{A_1, A_2\cdots A_{D'}\} \tag{56}$$

By an exhaustive enumeration over 230 SGs, the author of Supplementary Ref. [10] find that the dimensions of these two linear spaces are always same ($D = D'$) and all B_i can be expanded by $\{A_i\}$ with integral or fractional coefficients. B_i with fractional coefficients on $\{A_i\}$ must be topologically nontrivial because it mismatches with all possible atomic BRs. Then the SI of a given BR are defined as its expansion coefficients on the nontrivial B_i's. In this work we call each coefficient as a indicator or a SI, and the coefficient set as a SI set.

An observation follows, if $\mathcal{G}$, $\mathcal{G}'$ are two SGs and $\mathcal{G}' \subset \mathcal{G}$, then we can build a many-to-one mapping from the SI sets in $\mathcal{G}$ to the SI sets in $\mathcal{G}'$ by breaking the additional symmetries. If this mapping is one to one, i.e., any two different SI sets in $\mathcal{G}$ map to two different SI sets in $\mathcal{G}'$, we say that the SI sets in $\mathcal{G}$ are *induced* from the SI sets in $\mathcal{G}'$. After an exhaustive study, we find that the SI in *all* SGs can be induced from the following six SGs

$$\#2\ (P\bar{1}),\ \#81\ (P\bar{4}),\ \#83\ (P4/m),\ \#174\ (P\bar{6})$$

$$\#175\ (P6/m),\ \#176\ \ (P6_3/m)$$

Therefore, in order for the complete Fu-Kane-like formulae, we need only to derive the Fu-Kane-like formulae for this six SGs.

Five kinds of Fu-Kane-like formulae

$P\bar{1}$ formulae. SI of SG #2 ($P\bar{1}$) form the group $\mathbb{Z}_2\times\mathbb{Z}_2\times\mathbb{Z}_2\times\mathbb{Z}_4$. As discussed in Supplementary Ref. [10], the $\mathbb{Z}_4$ and $\mathbb{Z}_2$ generators are the strong and weak TIs, respectively. The $\mathbb{Z}_2$ indicators, denoted as $z_{2\mathrm{w},i=1,2,3}$ in the following, can be calculated by the Fu-Kane formula. And, by a detailed analysis on the BR, we find that the $\mathbb{Z}_4$ indicator can be calculated as

$$z_4 = \sum_{\mathbf{K}\in\mathrm{TRIM}} \frac{n_{\mathbf{K}}^- - n_{\mathbf{K}}^+}{2} \mod 4 \tag{57}$$

Here $n_{\mathbf{K}}^-$ is the number of occupied odd-parity Kramer pairs at $\mathbf{K}$, $n_{\mathbf{K}}^+$ is the number of occupied even-parity Kramer pairs at $\mathbf{K}$, and $\mathbf{K}$ is summed over all the eight time-reversal invariant momenta (TRIMs). According to the Fu-Kane criterion, $z_4 = 1, 3$ corresponds to strong TI. And, according to the discussion in Supplementary Note 5, $z_4 = 2$ corresponds to the inversion-protected TCI.

$P4/m$ formulae. SI of SG #83 ($P4/m$) form the group $\mathbb{Z}_2\times\mathbb{Z}_4\times\mathbb{Z}_8$, whose complete Fu-Kane-like formulae have been derived in Supplementary Ref. [5]. Here we only summarize the main results. The $\mathbb{Z}_2$ indicator is the weak TI indicator $z_{2\mathrm{w},1}$, which equals to $z_{2\mathrm{w},2}$ according to the C_4 symmetry. The $\mathbb{Z}_4$ indicator is the mirror Chern number (modulo 4) of the $k_z = \pi$ plane and will be denoted as $z_{4\mathrm{m},\pi}$ in the following. And the $\mathbb{Z}_8$ indicator is given by

$$z_8 = \left[3n_{\frac{3}{2}}^+ - 3n_{\frac{3}{2}}^- - n_{\frac{1}{2}}^+ + n_{\frac{1}{2}}^-\right]/2 \mod 8 \tag{58}$$

where the definitions for $n_{\frac{3}{2}}^+$, $n_{\frac{3}{2}}^-$, $n_{\frac{1}{2}}^+$, $n_{\frac{1}{2}}^-$ are given in Supplementary Table 1. According to Supplementary Ref. [5], if $z_{2\mathrm{w},1} = 0$ and $z_{4\mathrm{m},\pi} = 0$, odd z_8 corresponds to strong TI, $z_8 = 2, 6$ corresponds to mirror TCI with mirror Chern number 2 (modulo 4) in the $k_z = 0$ plane, and $z_8 = 4$ corresponds to either mirror TCI with mirror Chern number 4 (modulo 8) in the $k_z = 0$ plane or TCI with nontrivial C_4-rotation invariant.

$P\bar{6}$ formulae. SI of SG #174 ($P\bar{6}$) form the group $\mathbb{Z}_3\times\mathbb{Z}_3$. This SG has a horizontal mirror and a vertical C_3-rotation. The $\mathbb{Z}_3$ indicators are the mirror Chern numbers (modulo 3) of the $k_z = 0$ and the $k_z = \pi$ planes. This two indicators, denoted as $z_{3\mathrm{m},0}$ and $z_{3\mathrm{m},\pi}$, can be derived by applying the Chern number formula for C_3-invariant insulator [14] in each mirror eigenvalue sector. In Supplementary Table 2 we give the concrete expressions and their generalizations in other SGs with C_3 and mirror symmetries.

$P6/m$ formulae. SI of SG #175 ($P6/m$) form the group $\mathbb{Z}_6\times\mathbb{Z}_{12}$. As this SG has a vertical C_6-rotation symmetry, a horizontal mirror symmetry, and an inversion symmetry, we can at least calculate two mirror Chern numbers (modulo 6) in the $k_z = 0$ and $k_z = \pi$

Lattice	SGs	n	Definitions for $n_{\frac{3}{2}}^{+}$, $n_{\frac{3}{2}}^{-}$, $n_{\frac{1}{2}}^{+}$, $n_{\frac{1}{2}}^{-}$
Tetragonal primitive	83 (123, 124, 127, 128)[a]	$n_{\frac{1}{2}}^{+}$	$n(E^{\Gamma}_{\frac{1}{2}g}) + n(E^{M}_{\frac{1}{2}g}) + n(E^{Z}_{\frac{1}{2}g}) + n(E^{A}_{\frac{1}{2}g}) + n(E^{X}_{\frac{1}{2}g}) + n(E^{R}_{\frac{1}{2}g})$
		$n_{\frac{1}{2}}^{-}$	$n(E^{\Gamma}_{\frac{1}{2}u}) + n(E^{M}_{\frac{1}{2}u}) + n(E^{Z}_{\frac{1}{2}u}) + n(E^{A}_{\frac{1}{2}u}) + n(E^{X}_{\frac{1}{2}u}) + n(E^{R}_{\frac{1}{2}u})$
		$n_{\frac{3}{2}}^{+}$	$n(E^{\Gamma}_{\frac{3}{2}g}) + n(E^{M}_{\frac{3}{2}g}) + n(E^{Z}_{\frac{3}{2}g}) + n(E^{A}_{\frac{3}{2}g}) + n(E^{X}_{\frac{1}{2}g}) + n(E^{R}_{\frac{1}{2}g})$
		$n_{\frac{3}{2}}^{-}$	$n(E^{\Gamma}_{\frac{3}{2}u}) + n(E^{M}_{\frac{3}{2}u}) + n(E^{Z}_{\frac{3}{2}u}) + n(E^{A}_{\frac{3}{2}u}) + n(E^{X}_{\frac{1}{2}u}) + n(E^{R}_{\frac{1}{2}u})$
Tetragonal body-centred	87 (139, 140)[a]	$n_{\frac{1}{2}}^{+}$	$n(E^{\Gamma}_{\frac{1}{2}g}) + n(E^{M}_{\frac{1}{2}g}) + n(E^{X}_{\frac{1}{2}g}) + 2n(E^{N}_{\frac{1}{2}g}) + n(E^{P}_{\frac{1}{2}})$[b]
		$n_{\frac{1}{2}}^{-}$	$n(E^{\Gamma}_{\frac{1}{2}u}) + n(E^{M}_{\frac{1}{2}u}) + n(E^{X}_{\frac{1}{2}u}) + 2n(E^{N}_{\frac{1}{2}u}) + n(E^{P}_{\frac{3}{2}})$
		$n_{\frac{3}{2}}^{+}$	$n(E^{\Gamma}_{\frac{3}{2}g}) + n(E^{M}_{\frac{3}{2}g}) + n(E^{X}_{\frac{1}{2}g}) + 2n(E^{N}_{\frac{1}{2}g}) + n(E^{P}_{\frac{3}{2}})$
		$n_{\frac{3}{2}}^{-}$	$n(E^{\Gamma}_{\frac{3}{2}u}) + n(E^{M}_{\frac{3}{2}u}) + n(E^{X}_{\frac{1}{2}u}) + 2n(E^{N}_{\frac{1}{2}u}) + n(E^{P}_{\frac{1}{2}})$
Cubic primitive	221	$n_{\frac{1}{2}}^{+}$	$n(E^{\Gamma}_{\frac{1}{2}g}) + n(F^{\Gamma}_{\frac{3}{2}g}) + n(E^{R}_{\frac{1}{2}g}) + n(F^{R}_{\frac{3}{2}g}) + 2n(E^{M}_{\frac{1}{2}g}) + n(E^{M}_{\frac{3}{2}g}) + 2n(E^{X}_{\frac{1}{2}g}) + n(E^{X}_{\frac{3}{2}g})$
		$n_{\frac{1}{2}}^{-}$	$n(E^{\Gamma}_{\frac{1}{2}u}) + n(F^{\Gamma}_{\frac{3}{2}u}) + n(E^{R}_{\frac{1}{2}u}) + n(F^{R}_{\frac{3}{2}u}) + 2n(E^{M}_{\frac{1}{2}u}) + n(E^{M}_{\frac{3}{2}u}) + 2n(E^{X}_{\frac{1}{2}u}) + n(E^{X}_{\frac{3}{2}u})$
		$n_{\frac{3}{2}}^{+}$	$n(F^{\Gamma}_{\frac{3}{2}g}) + n(E^{\Gamma}_{\frac{5}{2}g}) + n(F^{R}_{\frac{3}{2}g}) + n(E^{R}_{\frac{5}{2}g}) + 2n(E^{M}_{\frac{3}{2}g}) + n(E^{M}_{\frac{1}{2}g}) + 2n(E^{X}_{\frac{3}{2}g}) + n(E^{X}_{\frac{1}{2}g})$
		$n_{\frac{3}{2}}^{-}$	$n(F^{\Gamma}_{\frac{3}{2}u}) + n(E^{\Gamma}_{\frac{5}{2}u}) + n(F^{R}_{\frac{3}{2}u}) + n(E^{R}_{\frac{5}{2}u}) + 2n(E^{M}_{\frac{3}{2}u}) + n(E^{M}_{\frac{1}{2}u}) + 2n(E^{X}_{\frac{3}{2}u}) + n(E^{X}_{\frac{1}{2}u})$
Cubic face-centred	225	$n_{\frac{1}{2}}^{+}$	$n(E^{\Gamma}_{\frac{1}{2}g}) + n(F^{\Gamma}_{\frac{3}{2}g}) + 2n(E^{X}_{\frac{1}{2}g}) + n(E^{X}_{\frac{3}{2}g}) + 2n(E^{L}_{\frac{1}{2}g}) + 2n(E^{L}_{\frac{3}{2}g}) + n(E^{W}_{\frac{1}{2}})$
		$n_{\frac{1}{2}}^{-}$	$n(E^{\Gamma}_{\frac{1}{2}u}) + n(F^{\Gamma}_{\frac{3}{2}u}) + 2n(E^{X}_{\frac{1}{2}u}) + n(E^{X}_{\frac{3}{2}u}) + 2n(E^{L}_{\frac{1}{2}u}) + 2n(E^{L}_{\frac{3}{2}u}) + n(E^{W}_{\frac{3}{2}})$
		$n_{\frac{3}{2}}^{+}$	$n(F^{\Gamma}_{\frac{3}{2}g}) + n(E^{\Gamma}_{\frac{5}{2}g}) + 2n(E^{X}_{\frac{3}{2}g}) + n(E^{X}_{\frac{1}{2}g}) + 2n(E^{L}_{\frac{1}{2}g}) + 2n(E^{L}_{\frac{3}{2}g}) + n(E^{W}_{\frac{3}{2}})$
		$n_{\frac{3}{2}}^{-}$	$n(F^{\Gamma}_{\frac{3}{2}u}) + n(E^{\Gamma}_{\frac{5}{2}u}) + 2n(E^{X}_{\frac{3}{2}u}) + n(E^{X}_{\frac{1}{2}u}) + 2n(E^{L}_{\frac{1}{2}u}) + 2n(E^{L}_{\frac{3}{2}u}) + n(E^{W}_{\frac{1}{2}})$
	226	$n_{\frac{1}{2}}^{+}$	$n(E^{\Gamma}_{\frac{1}{2}g}) + n(F^{\Gamma}_{\frac{3}{2}g}) + n(E^{X}_{\frac{3}{2}g}) + n(E^{X}_{\frac{1}{2}u}) + n(E^{X}_{\frac{3}{2}u})$
		$n_{\frac{1}{2}}^{-}$	$n(E^{\Gamma}_{\frac{1}{2}u}) + n(F^{\Gamma}_{\frac{3}{2}u}) + n(E^{X}_{\frac{3}{2}u}) + n(E^{X}_{\frac{1}{2}g}) + n(E^{X}_{\frac{3}{2}g})$
		$n_{\frac{3}{2}}^{+}$	$n(F^{\Gamma}_{\frac{3}{2}g}) + n(E^{\Gamma}_{\frac{5}{2}g}) + n(E^{X}_{\frac{1}{2}g}) + n(E^{X}_{\frac{1}{2}u}) + n(E^{X}_{\frac{3}{2}u})$
		$n_{\frac{3}{2}}^{-}$	$n(F^{\Gamma}_{\frac{3}{2}u}) + n(E^{\Gamma}_{\frac{5}{2}u}) + n(E^{X}_{\frac{1}{2}u}) + n(E^{X}_{\frac{1}{2}g}) + n(E^{X}_{\frac{3}{2}g})$
Cubic body-centred	229	$n_{\frac{1}{2}}^{+}$	$n(E^{\Gamma}_{\frac{1}{2}g}) + n(F^{\Gamma}_{\frac{3}{2}g}) + n(E^{H}_{\frac{1}{2}g}) + n(F^{H}_{\frac{3}{2}g}) + 3n(E^{N}_{\frac{1}{2}g}) + n(E^{P}_{\frac{1}{2}}) + n(F^{P}_{\frac{3}{2}})$
		$n_{\frac{1}{2}}^{-}$	$n(E^{\Gamma}_{\frac{1}{2}u}) + n(F^{\Gamma}_{\frac{3}{2}u}) + n(E^{H}_{\frac{1}{2}u}) + n(F^{H}_{\frac{3}{2}u}) + 3n(E^{N}_{\frac{1}{2}u}) + n(F^{P}_{\frac{3}{2}}) + n(E^{P}_{\frac{5}{2}})$
		$n_{\frac{3}{2}}^{+}$	$n(F^{\Gamma}_{\frac{3}{2}g}) + n(E^{\Gamma}_{\frac{5}{2}g}) + n(F^{H}_{\frac{3}{2}g}) + n(E^{H}_{\frac{5}{2}g}) + 3n(E^{N}_{\frac{1}{2}g}) + n(F^{P}_{\frac{3}{2}}) + n(E^{P}_{\frac{5}{2}})$
		$n_{\frac{3}{2}}^{-}$	$n(F^{\Gamma}_{\frac{3}{2}u}) + n(E^{\Gamma}_{\frac{5}{2}u}) + n(F^{H}_{\frac{3}{2}u}) + n(E^{H}_{\frac{5}{2}u}) + 3n(E^{N}_{\frac{1}{2}u}) + n(E^{P}_{\frac{1}{2}}) + n(F^{P}_{\frac{3}{2}})$

[a] The equations here are derived for SG #83 (#87) but also applicable to the SGs in the bracket, which are supergroups of SG #83 (#87). To apply these equations for these supergroups, one should omit the additional symmetries and count them as SG #83 (#87).

[b] In SG #87, the little group at N is C_i and the irrep notations in Supplementary Ref. [12] is $A_{\frac{1}{2}g}$ and $A_{\frac{1}{2}u}$, both of which are one dimensional. However, due to the Kramer's theorem, the irreps at N should be double degenerate, thus we adopt the two dimensional notations $E_{\frac{1}{2}g}$ and $E_{\frac{1}{2}u}$.

Supplementary Table 1. The concrete expressions for $n_{\frac{3}{2}}^{+}$, $n_{\frac{3}{2}}^{-}$, $n_{\frac{1}{2}}^{+}$, $n_{\frac{1}{2}}^{-}$ in the z_8 Fu-Kane-like formulae in all applicable SGs. The notations of high symmetry momenta follow the standard convention [13], and the notations of point group irreps follow Supplementary Ref. [12].

planes, denoted as $z_{6\text{m},0}$ and $z_{6\text{m},\pi}$, by applying the Chern number formula [14] in each mirror eigenvalue sector, and a z_4 indicator, as tabulated in Supplementary Table 2. It seems that the SI group should be $\mathbb{Z}_6 \times \mathbb{Z}_6 \times \mathbb{Z}_4$. However, the three indicators are not independent—$z_{6\text{m},0} + z_{6\text{m},\pi}$ and z_4 must have same parity because odd values of both indicate a strong TI, leading to the $\mathbb{Z}_6 \times \mathbb{Z}_{12}$ group. Choosing proper BR bases, we find that the $\mathbb{Z}_6$ generator corresponds to $z_{6\text{m},0} = 5$, $z_{6\text{m},\pi} = 1$, $z_4 = 0$, and the $\mathbb{Z}_{12}$ generator corresponds to $z_{6\text{m},0} = 1$, $z_{6\text{m},\pi} = 0$, $z_4 = 1$. In this convention, the relations between the $\mathbb{Z}_6$, $\mathbb{Z}_{12}$ indicators, denoted as z_6 and z_{12} respectively, and the $z_{6\text{m},0}$, $z_{6\text{m},\pi}$, z_4 indicators can be derived as

$$z_{6\text{m},0} = -z_6 + z_{12} \mod 6 \tag{59}$$

$$z_{6\text{m},\pi} = z_6 \mod 6 \tag{60}$$

$$z_4 = z_{12} \mod 4 \tag{61}$$

Therefore, the z_6 indicator is directly given by $z_{6\mathrm{m},\pi}$, and the z_{12} indicator is determined by the following two equations

$$z_{12} \mod 6 = z_{6\mathrm{m},0} + z_{6\mathrm{m},\pi} \tag{62}$$

$$z_{12} \mod 4 = z_4 \tag{63}$$

which is equivalent to

$$z_{12} = \left\{ \bar{z}_{6\mathrm{m}} + 3\left[(\bar{z}_{6\mathrm{m}} - z_4) \mod 4 \right] \right\} \mod 12 \tag{64}$$

where

$$\bar{z}_{6\mathrm{m}} = z_{6\mathrm{m},0} + z_{6\mathrm{m},\pi} \mod 6 \tag{65}$$

$P6_3/m$ formulae. SI of SG #176 ($P6_3/m$) form the group $\mathbb{Z}_{12}$. Due to the horizontal mirror symmetry a mirror Chern number for the $k_z = 0$ plane can be defined, and, its value (modulo 6) can be calculated from symmetry eigenvalues at high symmetry momenta in the $k_z = 0$ plane, where the C_6-screw is equivalent with C_6-rotation, by applying the Chern number formula for C_6-invariant insulator [14] (The mirror Chern number for $k_z = \pi$ plane always, however, equals to zero due to the C_6-screw symmetry.) Here we denote this the mirror Chern number (modulo 6) as indicator $z_{6\mathrm{m},0}$. On the other hand, due to the inversion symmetry the z_4 indicator can be also defined. Similar with indicators in SG $P6/m$, $z_{6\mathrm{m},0}$ and z_4 have same parity, leading to the SI group $\mathbb{Z}_{12}$. Therefore, the $\mathbb{Z}_{12}$ indicator can be calculated as

$$z'_{12} = \{z_{6\mathrm{m},0} + 3\left[(z_{6\mathrm{m},0} - z_4) \mod 4\right]\} \mod 12 \tag{66}$$

Here we use the prime notation to distinguish it from the $\mathbb{Z}_{12}$ indicator in SG $P6/m$ (Supplementary Eq. (64)).

The S_4 Fu-Kane-like formula

The SI of SG #81 ($P\bar{4}$) form the group $\mathbb{Z}_2$, and we find that the $\mathbb{Z}_2$ indicator can be calculated as

$$z_2 = \sum_{\mathbf{K}} \frac{n_{\mathbf{K}}^{\frac{3}{2}} - n_{\mathbf{K}}^{\frac{1}{2}}}{2} \mod 2 \tag{67}$$

where $\mathbf{K}$ is summed over all the four S_4-invariant TRIMs, $n_{\mathbf{K}}^{\frac{3}{2}}$ is the number of Kramer pairs at $\mathbf{K}$ with $\mathrm{tr}\left[D\left(S_4\right)\right] = -\sqrt{2}$, $n_{\mathbf{K}}^{\frac{1}{2}}$ is the number of Kramer pairs at $\mathbf{K}$ with $\mathrm{tr}\left[D\left(S_4\right)\right] = \sqrt{2}$, and $D\left(S_4\right)$ is the S_4 representation matrix on the corresponding Kramer pair.

The S_4 indicator z_2 has nothing to do with the S_4-invariant δ_{S_4} defined in Supplementary Note 1. By the models following, we find that $z_2 = 1$ corresponds to a strong TI or a Weyl semimetal. Since for $z_2 = 1$ there are two topologically distinct phases, i.e., $\delta_{S_4} = 0$ and $\delta_{S_4} = 1$ phases, we conjecture that the Weyl semimetal phase is the intermediate state between them. Correspondingly, $z_2 = 0$ corresponds to an insulator with $\delta_t = 0$ and $\delta_{S_4} = 0$ or 1, or a Weyl semimetal.

Strong TI. Consider the model

$$\hat{H}(\mathbf{k}) = \left[\Delta - \sum_i \cos k_i\right] \tau_z\sigma_0 + \sum_i \sin k_i \tau_x \sigma_i \tag{68}$$

where $\tau_{x,y,z}$ and $\sigma_{x,y,z}$ are Pauli matrices, and σ_0 is two by two identity matrix, the bases is set as $|\frac{1}{2}\rangle$, $|\bar{\frac{1}{2}}\rangle$, $|\frac{3}{2}\rangle$, $|\bar{\frac{3}{2}}\rangle$, i.e., the bases of $E_{\frac{1}{2}}$ and $E_{\frac{3}{2}}$ irreps. This model has rotoreflection symmetry $\hat{S}_4 = \tau_z e^{i\frac{\pi}{4}\sigma_z}$ and time-reversal symmetry $\hat{T} = -i\sigma_y K$. On one hand, the z_2 indicator can be calculated as: 1 for $1 < |\Delta| < 3$ and 0 for $|\Delta| < 1$ or $|\Delta| > 3$. On the other hand, according to Supplementary Ref. [15], this model is indeed a strong TI when $1 < |\Delta| < 3$. Therefore, in this model $z_2 = 1$ corresponds to strong TI.

Now, by the k·p analysis below, we argue that this correspondence is universal. We assume $\Delta = 3 + m$ and expand the model around (000) to linear terms in k. Then a Dirac Hamiltonian with mass m can be got as

$$H(\mathbf{k}) \approx m\tau_z\sigma_0 + \sum_i k_i \tau_x \sigma_i \tag{69}$$

It should be noticed that, although this k·p model is derived from the above tight binding model, it is the universal Hamiltonian around the phase transition point from strong TI to trivial insulator [16]. On one hand, the topological phase transition is given by the change of $\mathrm{sgn}(m)$. On the other hand, the change of $\mathrm{sgn}(m)$ must lead to a change of z_2 due to the following reason. If the model is S_4 invariant, the S_4 operator has to be $\hat{S}_4 = \tau_z \sigma^{i\frac{\pi}{4}\sigma_z}$ (up to a ± 1 sign), thus the change of $\mathrm{sgn}(m)$ inverts the $E_{\frac{1}{2}}$ and $E_{\frac{3}{2}}$ irreps at (000), leading to a change of z_2. Therefore, the topological phase transition is always accompanied by the change of z_2 indicator, and the correspondence between $z_2 = 1$ and strong TI should be universal.

Weyl semimetal with $z_2 = 1$. Consider the model

$$\hat{H}\left(\mathbf{k}\right) = \left(2 - \sum_i \cos k_i\right) \tau_z\sigma_0 + m \sin k_z \tau_x \sigma_z + \left(\cos k_x - \cos k_y\right) \tau_x \sigma_0 + \sin k_x \sin k_y \tau_y \sigma_z \tag{70}$$

This Hamiltonian has time-reversal symmetry $\hat{T} = -i\sigma_y$ and rotoreflection symmetry $\hat{S}_4 = \tau_z e^{i\frac{\pi}{4}\sigma_z}$, and the occupied states at the four S_4-invariant TRIMs give $z_2 = 1$ (at half-filling). Expand this model around $\left(00\frac{\pi}{2}\right)$ for the spin up ($\sigma_z = 1$) and spin down ($\sigma_z = -1$) components respectively, we get

$$\hat{H}^{\uparrow}(\mathbf{k}) \approx \delta k_z \tau_z + \left(k_y^2 - k_x^2 + m\right) \tau_x + k_x k_y \tau_y \tag{71}$$

and

$$\hat{H}^{\downarrow}(\mathbf{k}) \approx \delta k_z \tau_z + \left(k_y^2 - k_x^2 - m\right) \tau_x - k_x k_y \tau_y \tag{72}$$

Indicator	SGs	Formula
$z_{2\mathrm{w},j=1,2,3}$	All SGs with inversion	$\sum'_{\mathbf{K}} n^-_{\mathbf{K}} \mod 2$ [a]
z_4	All SGs with inversion	$\sum_{\mathbf{K}\in\mathrm{TRIM}} \frac{1}{2} n^-_{\mathbf{K}} - \frac{1}{2} n^+_{\mathbf{K}} \mod 4$
z_2	All SGs with S_4	$\sum_{\mathbf{K}} \frac{1}{2} n^{\frac{3}{2}}_{\mathbf{K}} - \frac{1}{2} n^{\frac{1}{2}}_{\mathbf{K}} \mod 2$ [b]
$z_{4\mathrm{m},\pi}$	83 (123, 127)[c]	$\frac{3}{2}n(E^{\mathrm{Z}}_{\frac{3}{2}\mathrm{g}}) - \frac{3}{2}n(E^{\mathrm{Z}}_{\frac{3}{2}\mathrm{u}}) - \frac{1}{2}n(E^{\mathrm{Z}}_{\frac{1}{2}\mathrm{g}}) + \frac{1}{2}n(E^{\mathrm{Z}}_{\frac{1}{2}\mathrm{u}}) + \frac{3}{2}n(E^{\mathrm{A}}_{\frac{3}{2}\mathrm{g}}) - \frac{3}{2}n(E^{\mathrm{A}}_{\frac{3}{2}\mathrm{u}})$ $-\frac{1}{2}n(E^{\mathrm{A}}_{\frac{1}{2}\mathrm{g}}) + \frac{1}{2}n(E^{\mathrm{A}}_{\frac{1}{2}\mathrm{u}}) + n(E^{\mathrm{R}}_{\frac{1}{2}\mathrm{g}}) - n(E^{\mathrm{R}}_{\frac{1}{2}\mathrm{u}}) \mod 4$
	221	$\frac{3}{2}n(E^{\mathrm{X}}_{\frac{3}{2}\mathrm{g}}) - \frac{3}{2}n(E^{\mathrm{X}}_{\frac{3}{2}\mathrm{u}}) - \frac{1}{2}n(E^{\mathrm{X}}_{\frac{1}{2}\mathrm{g}}) + \frac{1}{2}n(E^{\mathrm{X}}_{\frac{1}{2}\mathrm{u}})$ $+\frac{3}{2}n(E^{\mathrm{R}}_{\frac{5}{2}\mathrm{g}}) - \frac{3}{2}n(E^{\mathrm{R}}_{\frac{5}{2}\mathrm{u}}) + n(F^{\mathrm{R}}_{\frac{3}{2}\mathrm{g}}) - n(F^{\mathrm{R}}_{\frac{3}{2}\mathrm{u}}) - \frac{1}{2}n(E^{\mathrm{R}}_{\frac{1}{2}\mathrm{g}}) + \frac{1}{2}n(E^{\mathrm{R}}_{\frac{1}{2}\mathrm{u}})$ $+n(E^{\mathrm{M}}_{\frac{1}{2}\mathrm{g}}) + n(E^{\mathrm{M}}_{\frac{3}{2}\mathrm{g}}) - n(E^{\mathrm{M}}_{\frac{1}{2}\mathrm{u}}) - n(E^{\mathrm{M}}_{\frac{3}{2}\mathrm{u}}) \mod 4$
z_8	83, 87, 123, 124, 127, 128, 139, 140, 221, 225, 226, 229	$\frac{3}{2}n^+_{\frac{3}{2}} - \frac{3}{2}n^-_{\frac{3}{2}} - \frac{1}{2}n^+_{\frac{1}{2}} + \frac{1}{2}n^-_{\frac{1}{2}} \mod 8$ [d]
$z_{3\mathrm{m},0}$	174 (187, 188, 189, 190)[c]	$-\frac{1}{2}n(^1E^K_{\frac{1}{2}}) + \frac{3}{2}n(^1E^K_{\frac{3}{2}}) + \frac{1}{2}n(^1E^K_{\frac{5}{2}}) - \frac{1}{2}n(^2E^K_{\frac{1}{2}}) + \frac{3}{2}n(^2E^K_{\frac{3}{2}}) + \frac{1}{2}n(^2E^K_{\frac{5}{2}})$ $+n(E^{\Gamma}_{\frac{1}{2}}) - n(E^{\Gamma}_{\frac{5}{2}}) \mod 3$
$z_{3\mathrm{m},\pi}$	174 (187, 189) [c]	$-\frac{1}{2}n(^1E^{\mathrm{H}}_{\frac{1}{2}}) + \frac{3}{2}n(^1E^{\mathrm{H}}_{\frac{3}{2}}) + \frac{1}{2}n(^1E^{\mathrm{H}}_{\frac{5}{2}}) - \frac{1}{2}n(^2E^{\mathrm{H}}_{\frac{1}{2}}) + \frac{3}{2}n(^2E^{\mathrm{H}}_{\frac{3}{2}}) + \frac{1}{2}n(^2E^{\mathrm{H}}_{\frac{5}{2}})$ $+n(E^{\mathrm{A}}_{\frac{1}{2}}) - n(E^{\mathrm{A}}_{\frac{5}{2}}) \mod 3$
$z_{6\mathrm{m},0}$	175 (191, 192), 176 (193, 194)[c]	$\frac{3}{2}n(E^{\Gamma}_{\frac{3}{2}\mathrm{g}}) - \frac{5}{2}n(E^{\Gamma}_{\frac{5}{2}\mathrm{g}}) - \frac{1}{2}n(E^{\Gamma}_{\frac{1}{2}\mathrm{g}}) - \frac{3}{2}n(E^{\Gamma}_{\frac{3}{2}\mathrm{u}}) + \frac{5}{2}n(E^{\Gamma}_{\frac{5}{2}\mathrm{u}}) + \frac{1}{2}n(E^{\Gamma}_{\frac{1}{2}\mathrm{u}})$ $+3n(E^K_{\frac{3}{2}}) - 5n(E^K_{\frac{5}{2}}) - n(E^K_{\frac{1}{2}}) + \frac{3}{2}n(E^{\mathrm{M}}_{\frac{1}{2}\mathrm{g}}) - \frac{3}{2}n(E^{\mathrm{M}}_{\frac{1}{2}\mathrm{u}}) \mod 6$
$z_{6\mathrm{m},\pi}$	175 (191, 192) [c]	$\frac{3}{2}n(E^{\mathrm{A}}_{\frac{3}{2}\mathrm{g}}) - \frac{5}{2}n(E^{\mathrm{A}}_{\frac{5}{2}\mathrm{g}}) - \frac{1}{2}n(E^{\mathrm{A}}_{\frac{1}{2}\mathrm{g}}) - \frac{3}{2}n(E^{\mathrm{A}}_{\frac{3}{2}\mathrm{u}}) + \frac{5}{2}n(E^{\mathrm{A}}_{\frac{5}{2}\mathrm{u}}) + \frac{1}{2}n(E^{\mathrm{A}}_{\frac{1}{2}\mathrm{u}})$ $+3n(E^{\mathrm{H}}_{\frac{3}{2}}) - 5n(E^{\mathrm{H}}_{\frac{5}{2}}) - n(E^{\mathrm{H}}_{\frac{1}{2}}) + \frac{3}{2}n(E^{\mathrm{L}}_{\frac{1}{2}\mathrm{g}}) - \frac{3}{2}n(E^{\mathrm{L}}_{\frac{1}{2}\mathrm{u}}) \mod 6$
z_{12}	175, 191, 192	$\left\{\bar{z}_{6\mathrm{m}} + 3\left[(\bar{z}_{6\mathrm{m}} - z_4) \mod 4\right]\right\} \mod 12$ [e]
z'_{12}	176, 193, 194	$\left\{z_{6\mathrm{m},0} + 3\left[(z_{6\mathrm{m},0} - z_4) \mod 4\right]\right\} \mod 12$

[a] $\mathbf{K}$ is summed over the four TRIMs with $k_j = \pi$.

[b] $\mathbf{K}$ is summed over the four S_4 invariant TRIMs, $n^{\frac{1}{2}}_{\mathbf{K}}$ is the number of Kramer pairs at $\mathbf{K}$ with $\mathrm{tr}\left[D\left(S_4\right)\right] = \sqrt{2}$, $n^{\frac{3}{2}}_{\mathbf{K}}$ is the number of Kramer pairs at $\mathbf{K}$ with $\mathrm{tr}\left[D\left(S_4\right)\right] = -\sqrt{2}$, and $D(S_4)$ is the representation matrix on the corresponding Kramer pair.

[c] The equation is derived for the SG in front of the bracket but also applicable to the SGs in the bracket, which are supergroups of the SG in front of the bracket. To apply the equation for these supergroups, one should omit the additional symmetries and count them as the corresponding subgroup.

[d] The concrete definitions for $n^+_{\frac{3}{2}}$, $n^-_{\frac{3}{2}}$, $n^+_{\frac{1}{2}}$, and $n^-_{\frac{1}{2}}$ is given in Supplementary Table 1.

[e] Here $\bar{z}_{6\mathrm{m}} = z_{6\mathrm{m},0} + z_{6\mathrm{m},\pi} \mod 6$

Supplementary Table 2. Fu-Kane-like formulae for all SI. The indicators whose odd values correspond to strong TI are printed in red. The notations of high symmetry momenta follow the standard convention [13], and the notations of point group irreps follow Supplementary Ref. [12].

where $\delta k_z = k_z - \frac{\pi}{2}$. Two +1-change Weyl points and two -1-change Weyl points are found to locate at $\left(\pm\sqrt{m}, 0, \frac{\pi}{2}\right)$ and $\left(0, \pm\sqrt{m}, \frac{\pi}{2}\right)$, respectively. (Here we assume m is a positive small quantity.) Similarly, by expanding this model around $\left(0, 0, -\frac{\pi}{2}\right)$, we get two other +1-change Weyl points at $\left(\pm\sqrt{m}, 0, -\frac{\pi}{2}\right)$ and two other -1-change Weyl points at $\left(0, \pm\sqrt{m}, -\frac{\pi}{2}\right)$. The eight Weyl points at generic momenta form the minimal configuration satisfying both time-reversal and S_4 symmetries, as shown in Supplementary Figure 7a.

Without a band inversion at S_4-invariant TRIMs, there is no way to annihilate these Weyl points. For example, as shown in Supplementary Figure 7b, by tuning m to zero the two +1-charge Weyl points and the two -1-charge Weyl points at the $k_z = \frac{\pi}{2}$ plane will merge at $\left(00\frac{\pi}{2}\right)$ but can not annihilate each other. Instead, they form a double Dirac point, which is protected by the S_4 and time-reversal symmetries. (The terms anti-commuting with the $\tau_z\sigma_0$, $\tau_x\sigma_0$, and $\sigma_y\sigma_z$ terms in Supplementary Eq. (70) can only be $\tau_y\sigma_x$ and $\tau_y\sigma_y$. The corresponding coefficients, denoted as $I(\mathbf{k})$ and $J(\mathbf{k})$ respectively, should satisfy $I(\mathbf{k}) = I(-\mathbf{k})$, $J(\mathbf{k}) = J(-\mathbf{k})$, $I(-k_y, k_x, -k_z) = -J(\mathbf{k})$, $J(S_4\mathbf{k}) = I(\mathbf{k})$ and vanish at the Dirac points.) To annihilate the two double Dirac points at $(0, 0, \pm\pi/2)$, one need to move them to Γ, which causes a band inversion. In this particular model the

SI set	SI group	SGs
$z_{2\mathrm{w},1}$ $z_{2\mathrm{w},2}$ $z_{2\mathrm{w},3}$ z_4	$\mathbb{Z}_2 \times \mathbb{Z}_2 \times \mathbb{Z}_2 \times \mathbb{Z}_4$	2, 10, 47
	$\mathbb{Z}_2 \times \mathbb{Z}_2 \times \mathbb{Z}_4$	11, 12, 13, 49, 51, 65, 67, 69
	$\mathbb{Z}_2 \times \mathbb{Z}_4$	14, 15, 48, 50, 53, 54, 55, 57, 59, 63, 64, 66, 68, 71, 72, 73, 74, 84, 85, 86, 125, 129, 131, 132, 134, 147, 148, 162, 164, 166, 200, 201, 204, 206, 224
	$\mathbb{Z}_4$	52, 56, 58, 60, 61, 62, 70, 88, 126, 130, 133, 135, 136, 137, 138, 141, 142, 163, 165, 167, 202, 203, 205, 222, 223, 227, 228, 230
z_2	$\mathbb{Z}_2$	81, 82, 111, 112, 113, 114, 115, 116, 117, 118, 119, 120, 121, 122, 215, 216, 217, 218, 219, 220
$z_{2\mathrm{w},1}$ $z_{4\mathrm{m},\pi}$ z_8	$\mathbb{Z}_2 \times \mathbb{Z}_4 \times \mathbb{Z}_8$	83, 123
$z_{2\mathrm{w},1}$ z_8	$\mathbb{Z}_2 \times \mathbb{Z}_8$	87, 124, 139, 140, 229
$z_{4\mathrm{m},\pi}$ z_8	$\mathbb{Z}_4 \times \mathbb{Z}_8$	127, 221
z_8	$\mathbb{Z}_8$	128, 225, 226
$z_{3\mathrm{m},0}$ $z_{3\mathrm{m},\pi}$	$\mathbb{Z}_3 \times \mathbb{Z}_3$	174, 187, 189
$z_{3\mathrm{m},0}$	$\mathbb{Z}_3$	188, 190
$z_{6\mathrm{m},\pi}$ z_{12}	$\mathbb{Z}_6 \times \mathbb{Z}_{12}$	175, 191
z_{12}	$\mathbb{Z}_{12}$	192
z'_{12}	$\mathbb{Z}_{12}$	176, 193, 194

Supplementary Table 3. SI in all SGs. The indicators whose odd values correspond to strong TI are printed in red.

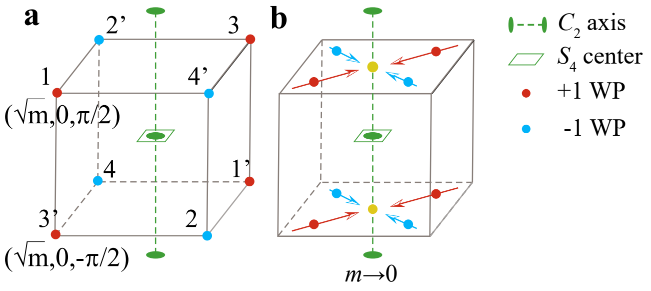

Supplementary Figure 7. The S_4 Weyl semimetal. In **a** we plot the eight Weyl points in model Supplementary Eq. (70), where the red circles represent +1-charge Weyl points and the blue circles represent -1-charge Weyl points. The Weyl points labeled with 1, 2, 3, 4 transform to each other in turn under the S_4 operation, and transform to the Weyl points labeled with 1', 2', 3', 4' respectively under the time reversal. In **b**, we plot the motion of these Weyl points as m approaches zero. When $m = 0$ four Weyl points merge to a double Dirac point.

band inversion causes a change of z_2, however, in general case it does not. For instance, here we consider a product state consisting of a Weyl semimetal model with $z_2 = 1$ and two strong TI models. The z_2 indicator of this product state is given by $z_2 = 1 + 1 + 1 \bmod 2 = 1$. Then the process annihilating the Weyl points without changing z_2 can be constructed as: (i) move the eight Weyl points to (000) to cause a band inversion, (ii) at the same time close and reopen the gap of one of the two TI models. After this process, all the Weyl points are gapped and the z_2 indicator remains unchanged, i.e. $z_2 = 0 + 0 + 1 = 1$.

It should be emphasized that in all the other non-centrosymmetric SGs with nontrivial SI groups, i.e., #174 and #187-190, Weyl points can be annihilated without band inversion at high symmetry momentum. In all these five SGs $k_z = 0$-plane is mirror-invariant, and Weyl points at generic momenta should constitute symmetric pairs about the mirror, where each pair consists of two Weyl points with opposite charges. Therefore, each pair of Weyl points can move toward each other until they annihilate each other at the $k_z = 0$-plane. To be specific, let us assume the k·p model of a pair of Weyl points moved to the $k_z = 0$-plane as

$$\hat{H}(\delta\mathbf{k}) = \sum_i \delta k_i \tau_z \sigma_i \tag{73}$$

where $\delta\mathbf{k} = \mathbf{k} - \mathbf{k}_c$ and $\mathbf{k}_c$ is the position of the two Weyl points. The mirror operator can be chosen as $\hat{m}_z = i\tau_x\sigma_z$. Apparently, the mass term $m\tau_x$ is symmetry-allowed. Thus the Weyl points can pairwise annihilate at generic point in the $k_z = 0$ plane without any level crossing at high symmetry momentum.

Convention dependence of z_4, z_8, z_{12}, z'_{12} indicator

In the text we have discussed that by redefining $\hat{P} \to -\hat{P}$ $z_4 = 1,3$ turn into each other, and without external reference $z_4 = 1,3$ should be physically identical. Such a convention dependence also exists for the z_8, z_{12}, and z'_{12} indicator (Supplementary Table 2).

For the z_8 indicator, according to Supplementary Table 1, redefining $\hat{P} \to -\hat{P}$ interchanges $n^{+}_{\frac{3}{2}/\frac{1}{2}}$ and $n^{-}_{\frac{3}{2}/\frac{1}{2}}$, and redefining $\hat{C}_4 \to -\hat{C}_4$ interchanges $n^{+/-}_{\frac{3}{2}}$ and $n^{+/-}_{\frac{1}{2}}$. Substitute this relation into Supplementary Eq. (58), we find that there are only four kinds of physically distinct cases: (i) $z_8 = 0$, (ii) $z_8 = 1,3,5,7$ corresponding to the strong TI, (iii) $z_8 = 2,6$, and (iv) $z_8 = 4$.

The z_{12} indicator is completely determined by the mirror Chern number indicator $z_{6\mathrm{m},0}$, $z_{6\mathrm{m},\pi}$ and the z_4 indicator, wherein $z_4 = 1, 3$ turn into each other when redefining $\hat{P} \to -\hat{P}$, and $z_{6\mathrm{m},0/\pi} = 1, 5$ turn into each other when redefining $\hat{C}_6 \to -\hat{C}_6$. Substitute this into Supplementary Eq. (64) we conclude that there are only six kinds of physically distinct cases: (i) $z_{12} = 0$, (ii) $z_{12} = 1, 5, 7, 11$, (iii) $z_{12} = 2, 10$, (iv) $z_{12} = 3, 9$, (v) $z_{12} = 4, 8$, and (vi) $z_{12} = 6$, wherein (ii) and (iv) correspond to strong TI. The difference between (ii) and (iv) is the magnitude of mirror Chern numbers: in (ii) $C_{\mathrm{m},0}+C_{\mathrm{m},\pi} = \pm 1$ (modulo 6), while in (iv) $C_{\mathrm{m},0} + C_{\mathrm{m},\pi} = 3$ (modulo 6).

The discussion for the z'_{12} indicator is similar with the discussion for the z_{12} indicator. And the conclusion is same—there are only six kinds of physically distinct cases, i.e., (i) $z'_{12} = 0$, (ii) $z'_{12} = 1, 5, 7, 11$, (iii) $z'_{12} = 2, 10$, (iv) $z'_{12} = 3, 9$, (v) $z'_{12} = 4, 8$, and (vi) $z'_{12} = 6$, wherein (ii) and (iv) corresponds to strong TI. And, the difference between (ii) and (iv) is also the magnitude of mirror Chern numbers: in (ii) $C_{\mathrm{m},0} = \pm 1$ (modulo 6), while in (iv) $C_{\mathrm{m},0} = 3$ (modulo 6).

Fu-Kane-like formulae for all symmetry-based indicators

To summarize, we tabulate all the Fu-Kane-like formulae in Supplementary Table 2 and their relation with SI groups in Supplementary Table 3. Two comments are made here. The first is that, in the second to fourth rows of Supplementary Table 3, the Fu-Kane-like formulae seem to "mismatch" with the SI groups. This is simply because some weak indicators are trivialized by some SG operations. For example, the SI group of SG #11 ($P2_1/m$) is $\mathbb{Z}_2 \times \mathbb{Z}_2 \times \mathbb{Z}_4$ instead of $\mathbb{Z}_2 \times \mathbb{Z}_2 \times \mathbb{Z}_2 \times \mathbb{Z}_4$ because the C_2-screw axis (along $\mathbf{a}_2$) enforce the four TRIMs at $k_2 = \pi$ to have same parities and so enforce $z_{2\mathrm{w},2} = 0$. Thus, there should be no worry about this "mismatch". The second is that, the SI itself can not tell us whether a state is fully gapped. Therefore, to apply the Fu-Kane-like formulae one should firstly check whether the band is fully gapped.

Supplementary Note 5. Symmetry-based indicator of layer construction

In Supplementary Notes 1 to 3, we developped systematic methods to (i) diagnose the TCI invariants of an eLC according to its geometry configuration and (ii) enumerate all nonequivalent eLCs. With all the nonequivalent eLCs at hand it is direct to enumerate all the TCI states consistent with LCs by stacking the eLCs together. On the other hand, if the SI of eLCs can be calculated then the SI of all these TCI states can also be calculated due to the additive property of SI. In other words, we can use eLC as intermedia to complete the mapping from SI to TCI invariants. However, the inputs of Fu-Kane-like formulae derived in Supplementary Note 4 are the numbers of irreps at high symmetry momenta, which are not explicit for eLC. Therefore, to finish the mapping in this Supplementary Note we give the method to calculate the SI of eLCs.

Firstly, one should notice that the SI can be uniquely determined from a complete set of topological invariants, because by definition two different SI corresponds to two different topologies. Here we take the *assumption* that the the seven kinds of invariants introduced in Supplementary Note 1 are complete for LC states. Therefore the SI of a LC is uniquely determined by these invariants. Secondly, as shown in Supplementary Note 4 SI in all SGs can be induced from only six SGs, thus to calculate the SI of an eLC E in any SG $\mathcal{G}$ we can (i) reduce $\mathcal{G}$ to one of the six SGs, say $\mathcal{G}'$, (ii) calculate the topological invariants of E in $\mathcal{G}'$, and (iii) calculate the SI in $\mathcal{G}'$ due to the invariants. Therefore, we need only to derive the SI of eLCs in these six SGs.

$P\bar{1}$

According to the discussion in Supplementary Note 1, the TCI invariants of SG #2 ($P\bar{1}$) should include three weak invariants $\delta_{\mathrm{w},i=1,2,3}$ and an inversion invariant δ_{i}, which is defined on the inversion center at the origin (see Supplementary Note 7 for the convention). There are only four independent eLCs—$E_1 = \mathrm{eLC}\,(100;0)$, $E_2 = \mathrm{eLC}\,(010;0)$, $E_3 = \mathrm{eLC}\,(001;0)$, $E_4 = \mathrm{eLC}\left(001;\frac{1}{2}\right)$, whose invariants are

$$\{\delta_{\mathrm{w},i=1,2,3},\delta_{\mathrm{i}}\}\,(E_1) = \{100,1\} \tag{74}$$

$$\{\delta_{\mathrm{w},i=1,2,3},\delta_{\mathrm{i}}\}\,(E_2) = \{010,1\} \tag{75}$$

$$\{\delta_{\mathrm{w},i=1,2,3},\delta_{\mathrm{i}}\}\,(E_3) = \{001,1\} \tag{76}$$

$$\{\delta_{\mathrm{w},i=1,2,3},\delta_{\mathrm{i}}\}\,(E_4) = \{001,0\} \tag{77}$$

as shown in Supplementary Figure 8a and tabulated in Supplementary Table 5. The SI set includes three weak TI indicators $z_{2\mathrm{w},i=1,2,3}$ and one $\mathbb{Z}_4$ indicator z_4, where $z_{2\mathrm{w},i=1,2,3}$ equals to $\delta_{\mathrm{w},i=1,2,3}$ according to the Fu-Kane criterion. To calculate the z_4 indicator, let us assume that the 2D TI in E_3 (Supplementary Figure 8a) gives the BR

$$E^{\Gamma}_{\frac{1}{2}\mathrm{u}}\ E^{\mathrm{X}}_{\frac{1}{2}\mathrm{g}}\ E^{\mathrm{Y}}_{\frac{1}{2}\mathrm{g}}\ E^{\mathrm{V}}_{\frac{1}{2}\mathrm{g}} \tag{78}$$

in the $k_z = 0$ plane. Taking this 2D TI as building block, we get the 3D BRs for the four eLCs, as shown in Supplementary Figure 8a. It should be noticed that, for E_4, the $k_3 = \pi$ states have opposite parities with $k_3 = 0$ states; while for $E_{1,2,3}$, the $k_{1,2,3} = \pi$ states have same parities with $k_{1,2,3} = 0$ states. (Analysis on parities resembles the analysis on mirror eigenvalues, as discussed in Supplementary Note 1 and sketched in Supplementary Figure 5a.) Substitute these BRs into Supplementary Table 2, we get

$$\{z_{2\mathrm{w},i=1,2,3},z_4\}\,(E_1) = \{100,2\} \tag{79}$$

$$\{z_{2\mathrm{w},i=1,2,3},z_4\}\,(E_2) = \{010,2\} \tag{80}$$

$$\{z_{2\mathrm{w},i=1,2,3},z_4\}\,(E_3) = \{001,2\} \tag{81}$$

$$\{z_{2\mathrm{w},i=1,2,3},z_4\}\,(E_4) = \{001,0\} \tag{82}$$

$P4/m$

The invariants in SG #83 ($P4/m$) include three weak invariants $\delta_{\mathrm{w},i=1,2,3}$, two mirror Chern numbers $C_{\mathrm{m},0/\pi}$, a C_4-rotation invariant δ_{r}, an inversion invariant δ_{i}, and a S_4 invariant δ_{S_4}. The independent eLCs are $E_1 = \mathrm{eLC}\,(001;0)$, $E_2 = \mathrm{eLC}\left(001;\frac{1}{2}\right)$, $E_3 = (100;0)$, $E_4 = \left(100;\frac{1}{2}\right)$, and their invariants are

$$\{\delta_{\mathrm{w},i=1,2,3},C_{\mathrm{m},k=0,\pi},\delta_{\mathrm{r}},\delta_{\mathrm{i}},\delta_{S_4}\}\,(E_1) \\ =\{001,11,0,1,1\} \tag{83}$$

$$\{\delta_{\mathrm{w},i=1,2,3},C_{\mathrm{m},k=0,\pi},\delta_{\mathrm{r}},\delta_{\mathrm{i}},\delta_{S_4}\}\,(E_2) \\ =\{001,1\bar{1},0,0,0\} \tag{84}$$

$$\{\delta_{\mathrm{w},i=1,2,3},C_{\mathrm{m},k=0,\pi},\delta_{\mathrm{r}},\delta_{\mathrm{i}},\delta_{S_4}\}\,(E_3) \\ =\{110,00,1,0,1\} \tag{85}$$

$$\{\delta_{\mathrm{w},i=1,2,3},C_{\mathrm{m},k=0,\pi},\delta_{\mathrm{r}},\delta_{\mathrm{i}},\delta_{S_4}\}\,(E_4) \\ =\{110,00,0,0,0\} \tag{86}$$

, as shown in Supplementary Figure 8b. We assume the 2D TI in E_1 has the BR

$$E^{\Gamma}_{\frac{1}{2}\mathrm{u}}\ E^{\mathrm{X}}_{\frac{1}{2}\mathrm{g}}\ E^{\mathrm{M}}_{\frac{1}{2}\mathrm{g}} \tag{87}$$

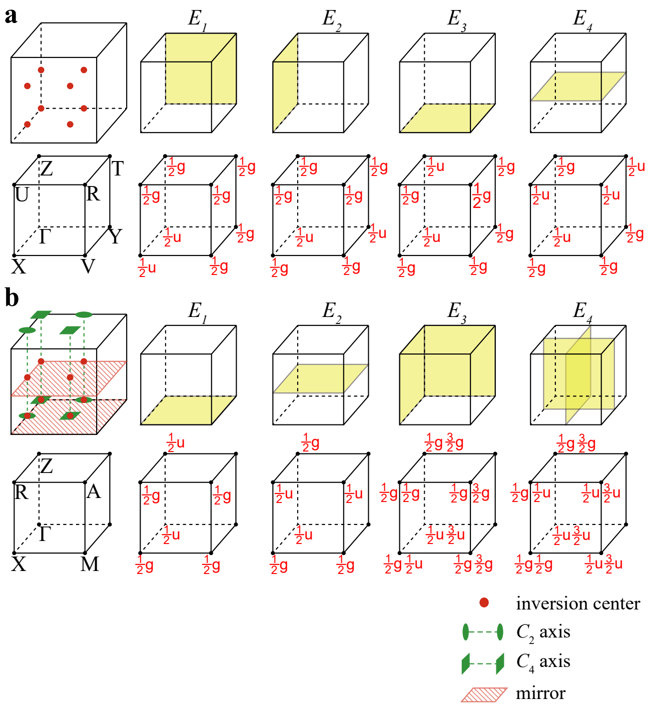

Supplementary Figure 8. **a** are the band representations of the four independent eLCs in SG $P\bar{1}$, and **b** are the band representations of the four independent eLCs in SG $P4/m$. In the top panel of each, we plot the symmetry elements and the real space eLCs, in the bottom panel of each, we plot the irreducible Brillouin zone and the irreps of the corresponding eLCs.

in the $k_3 = 0$ plane, using which as building Blocks we can derive the BRs of E_2, E_3, E_4. In E_1 the $k_3 = \pi$ states have same parities with $k_3 = 0$ states; while in E_2 the $k_3 = \pi$ states have opposite parities with the $k_3 = 0$ states. In E_3 and E_4, the parities at TRIMs can be derived in a similar way, and the irreps at C_4-invariant TRIMs must consist of a pair of $E_{\frac{1}{2},g/u}$ and $E_{\frac{3}{2},g/u}$ because the C_4 operation transforms a layer to another such that the C_4 representation matrix must be traceless. The BRs of the four eLCs are summarized in Supplementary Figure 8b, substitute which into equations in Supplementary Table 2 we get

$$\{z_{2\mathrm{w},1}, z_{4\mathrm{m},\pi}, z_8\}(E_1) = \{0, 1, 2\} \tag{88}$$

$$\{z_{2\mathrm{w},1}, z_{4\mathrm{m},\pi}, z_8\}(E_2) = \{0, 3, 0\} \tag{89}$$

$$\{z_{2\mathrm{w},1}, z_{4\mathrm{m},\pi}, z_8\}(E_3) = \{1, 0, 4\} \tag{90}$$

$$\{z_{2\mathrm{w},1}, z_{4\mathrm{m},\pi}, z_8\}(E_4) = \{1, 0, 0\} \tag{91}$$

Supplementary Eq. (83) to Supplementary Eq. (91) are consistent with the results in Supplementary Ref. [5].

$P\bar{6}$

SG #174 ($P\bar{6}$) has five invariants, i.e., three weak invariants $\delta_{\mathrm{w},i=1,2,3}$ and two mirror Chern numbers $C_{\mathrm{m},k_z=0,\pi}$. And, the two independent eLCs $E_1 = \mathrm{eLC}\,(001;0)$, $E_2 = \mathrm{eLC}\,(001;\frac{1}{2})$ correspond to

$$\{\delta_{\mathrm{w},i=1,2,3}, C_{\mathrm{m},k=0,\pi}\}(E_1) = \{001, 11\} \tag{92}$$

$$\{\delta_{\mathrm{w},i=1,2,3}, C_{\mathrm{m},k=0,\pi}\}(E_2) = \{001, 1\bar{1}\} \tag{93}$$

Correspondingly, the SI (Chern number modulo 3) can be directly got as

$$\{z_{3\mathrm{m},k=0,\pi}\}(E_1) = \{11\} \tag{94}$$

$$\{z_{3\mathrm{m},k=0,\pi}\}(E_2) = \{12\} \tag{95}$$

$P6/m$

SG #175 ($P6/m$) has eight TCI invariants, i.e., three weak invariants $\delta_{\mathrm{w},i=1,2,3}$, two mirror Chern numbers $C_{\mathrm{m},k=0,\pi}$, a C_6-rotation invariant δ_{r}, and an inversion invariant δ_{i} And, the three independent eLCs $E_1 = \mathrm{eLC}\,(001;0)$, $E_2 = \mathrm{eLC}\,(001;\frac{1}{2})$, $E_3 = \mathrm{eLC}\,(100;0)$ correspond to

$$\{\delta_{\mathrm{w},i=1,2,3}, C_{\mathrm{m},k=0,\pi}, \delta_{\mathrm{r}}, \delta_{\mathrm{i}}\}(E_1) = \{001, 11, 0, 1\} \tag{96}$$

$$\{\delta_{\mathrm{w},i=1,2,3}, C_{\mathrm{m},k=0,\pi}, \delta_{\mathrm{r}}, \delta_{\mathrm{i}}\}(E_2) = \{001, 1\bar{1}, 0, 0\} \tag{97}$$

$$\{\delta_{\mathrm{w},i=1,2,3}, C_{\mathrm{m},k=0,\pi}, \delta_{\mathrm{r}}, \delta_{\mathrm{i}}\}(E_3) = \{000, 00, 1, 1\} \tag{98}$$

According to Supplementary Note 4, SG $P6/m$ has the SI set $\{z_{6\mathrm{m},\pi}, z_{12}\}$. The $z_{6\mathrm{m},\pi}$ indicator can be got as $C_{\mathrm{m},k=\pi}$ mod 6, and the z_{12} indicator can be determined from $z_{6\mathrm{m},k=0} + z_{6\mathrm{m},k=\pi}$ and z_4 (Supplementary Table 2), wherein $z_{6\mathrm{m},k=0} + z_{6\mathrm{m},k=\pi}$ can be calculated as $C_{\mathrm{m},k=0} + C_{\mathrm{m},k=\pi}$ mod 6 and the z_4 indicator can be calculated by group reduction to $P\bar{1}$. We get

$$\{z_{6\mathrm{m},\pi}, z_{12}\}(E_1) = \{1, 2\} \tag{99}$$

$$\{z_{6\mathrm{m},\pi}, z_{12}\}(E_2) = \{5, 0\} \tag{100}$$

$$\{z_{6\mathrm{m},\pi}, z_{12}\}(E_3) = \{0, 6\} \tag{101}$$

$P6_3/m$

The invariants in SG #176 ($P6_3/m$) include three weak invariants $\delta_{\mathrm{w},i=1,2,3}$, two mirror Chern numbers $C_{\mathrm{m},k=0,\pi}$, an inversion invariant δ_{i}, and a C_6-screw invariant δ_{s}. There are two independent eLCs, $E_1 = \mathrm{eLC}\,(001;0)$, $E_2 = \mathrm{eLC}\,\left(001;\frac{1}{4}\right)$, whose invariants are

$$\begin{aligned} &\{\delta_{\mathrm{w},i=1,2,3}, C_{\mathrm{m},k=0,\pi}, \delta_{\mathrm{i}}, \delta_{\mathrm{s}}\}\,(E_1) \\ =&\{000, 00, 1, 1\} \end{aligned} \tag{102}$$

$$\begin{aligned} &\{\delta_{\mathrm{w},i=1,2,3}, C_{\mathrm{m},k=0,\pi}, \delta_{\mathrm{i}}, \delta_{\mathrm{s}}\}\,(E_2) \\ =&\{000, 20, 0, 1\} \end{aligned} \tag{103}$$

Similar with SG #175 ($P6/m$), their SI can be calculated as

$$z'_{12}(E_1) = 6, \qquad z'_{12}(E_2) = 8 \tag{104}$$

$P\bar{4}$

$z_2 = 1$ corresponds to strong TI or Weyl semimetal, neither of which can be realized by layer construction. Thus all the eLCs in SG $P\bar{4}$ have a trivial indicator.

Supplementary Note 6. Weak topological insulators beyond layer constructions

Using the layer construction method introduced above we success to obtain all the SI in all SGs except five (and only five) corner cases, where we find that the compatibility-relation allowed weak TIs can not be realized in any LC. These corner cases are SGs #48 ($Pnnn$), #86 ($P4_2/n$), #134 ($P4_2/nnm$), #201 ($Pn\bar{3}$), and #224 ($Pn\bar{3}m$), all of which are centrosymmetric and have the SI group $\mathbb{Z}_2 \times \mathbb{Z}_4$. The $\mathbb{Z}_4$ indicator is z_4 and the $\mathbb{Z}_2$ indicator is the weak TI indicator $z_{2\mathrm{w},1}$, which equals to the other two weak TI indicators, i.e., $z_{2\mathrm{w},1} = z_{2\mathrm{w},2} = z_{2\mathrm{w},3}$, due to the compatibility relation.

In this Supplementary Note, we will construct a tight-binding model for the weak TI in SG $Pn\bar{3}m$. Since all the other four SGs are subgroups of $Pn\bar{3}m$ and the corresponding symmetry breaking from $Pn\bar{3}m$ does not expand the cells, the weak TI models in the other four SGs can be got from this model by a slight breaking of additional crystalline symmetries. Therefore, by this model we show that (i) compatibility-relation allowed weak TIs in these SGs can indeed be realized in tight-binding models and (ii) these weak TIs are beyond the scope of layer construction.

Now let us construct the model. $Pn\bar{3}m$ has a primitive cubic lattice, and its SG generators include inversion $P = \{-1|000\}$, glide $n = \{m_{001}|\frac{1}{2}\frac{1}{2}0\}$, rotation $C_3 = \{3_{111}|000\}$, and mirror $m = \{m_{1\bar{1}0}|000\}$ [17]. Here we consider the Wyckoff position $2a$, which include two positions $(\frac{1}{4}\frac{1}{4}\frac{1}{4})$ and $(\frac{\bar{1}}{4}\frac{\bar{1}}{4}\frac{\bar{1}}{4})$ and has the site-symmetry group T_d. We choose the bases of $E_{\frac{1}{2}}$ and $E_{\frac{5}{2}}$ irreps on $2a$ as the tight-binding model bases. Then the SG operator on Bloch bases can be derived as

$$\hat{P} = \mu_0\tau_x\sigma_0 \tag{105}$$

$$\hat{n} = -ie^{-i\frac{k_x+k_y}{2}}\mu_0\left[\cos\frac{k_x+k_y}{2} - \sin\frac{k_x+k_y}{2}\right]\sigma_z \tag{106}$$

$$\hat{C}_3 = \frac{1}{2}\mu_0\tau_0[\sigma_0 - i\sigma_x - i\sigma_y - i\sigma_z] \tag{107}$$

$$\hat{m} = \frac{1}{\sqrt{2}}\mu_z\tau_0[-i\sigma_x + i\sigma_y] \tag{108}$$

where μ_i, τ_i, σ_i are pauli matrices representing the $E_{\frac{1}{2}}$ and $E_{\frac{5}{2}}$ irreps, the $(\frac{1}{4}\frac{1}{4}\frac{1}{4})$ and $(\frac{\bar{1}}{4}\frac{\bar{1}}{4}\frac{\bar{1}}{4})$ positions, and the two bases in each irrep, respectively. The Bloch bases are defined as a Fourier transformation of the orbitals on $2a$

$$|\phi_{\alpha\mathbf{k}}\rangle = \frac{1}{\sqrt{N}}\sum_R e^{i\mathbf{k}\cdot\mathbf{R}}|a_{\alpha\mathbf{R}}\rangle \tag{109}$$

where $|a_{\alpha\mathbf{R}}\rangle$ is the α-th orbital in the lattice $\mathbf{R}$, and α is a composite index consisting of irreps, sites, and bases of irreps. The equation for tight-binding model is given by

$$\hat{g}\hat{H}(\mathbf{k})\hat{g}^{-1} = \hat{H}(g\mathbf{k}) \tag{110}$$

Substitute Supplementary Eq. (105) to (108) to it, we get a solution as

$$\begin{aligned} H(\mathbf{k}) = {} & \Delta\mu_z\tau_0\sigma_0 + f_x(\mathbf{k})\mu_0\tau_x\sigma_0 + f_y(\mathbf{k})\mu_0\tau_y\sigma_0 + f_x^z(\mathbf{k})\mu_y\tau_x\sigma_z + f_y^z(\mathbf{k})\mu_y\tau_y\sigma_z + f_x^x(\mathbf{k})\mu_y\tau_x\sigma_x + f_y^x(\mathbf{k})\mu_y\tau_y\sigma_x \\ & + f_x^y(\mathbf{k})\mu_y\tau_x\sigma_y + f_y^y(\mathbf{k})\mu_y\tau_y\sigma_y \end{aligned} \tag{111}$$

where

$$f_x(\mathbf{k}) = t_1\cos\left(\frac{k_x+k_y+k_z}{2}\right)\cos\frac{k_x}{2}\cos\frac{k_y}{2}\cos\frac{k_z}{2} + t_2\sin\left(\frac{k_x+k_y+k_z}{2}\right)\sin\frac{k_x}{2}\sin\frac{k_y}{2}\sin\frac{k_z}{2} \tag{112}$$

$$f_y(\mathbf{k}) = -t_1\sin\left(\frac{k_x+k_y+k_z}{2}\right)\cos\frac{k_x}{2}\cos\frac{k_y}{2}\cos\frac{k_z}{2} + t_2\cos\left(\frac{k_x+k_y+k_z}{2}\right)\sin\frac{k_x}{2}\sin\frac{k_y}{2}\sin\frac{k_z}{2} \tag{113}$$

$$f_x^z(\mathbf{k}) = \lambda_1\cos\left(\frac{k_x+k_y+k_z}{2}\right)\sin\frac{k_x}{2}\sin\frac{k_y}{2}\cos\frac{k_z}{2} + \lambda_2\sin\left(\frac{k_x+k_y+k_z}{2}\right)\cos\frac{k_x}{2}\cos\frac{k_y}{2}\sin\frac{k_z}{2} \tag{114}$$

$$f_y^z(\mathbf{k}) = -\lambda_1\sin\left(\frac{k_x+k_y+k_z}{2}\right)\sin\frac{k_x}{2}\sin\frac{k_y}{2}\cos\frac{k_z}{2} + \lambda_2\cos\left(\frac{k_x+k_y+k_z}{2}\right)\cos\frac{k_x}{2}\cos\frac{k_y}{2}\sin\frac{k_z}{2} \tag{115}$$

and

$$f_{x/y}^x(k_xk_yk_z) = f_{x/y}^z(k_yk_zk_x) \qquad f_{x/y}^y(k_xk_yk_z) = f_{x/y}^z(k_zk_xk_y) \tag{116}$$

Here Δ, t_1, and t_2 are the non-spin-orbital-coupling parameters, and λ_1, λ_2 are the spin-orbital-coupling pa-

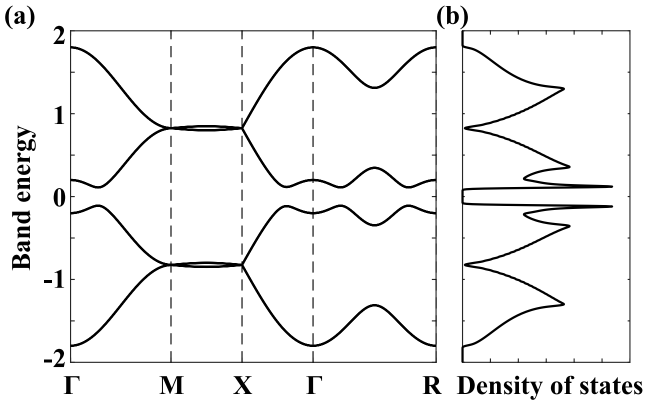

Supplementary Figure 9. In **a** and **b** the band structure and density of states of the weak TI model for SG $Pn\bar{3}m$ are ploted, respectively. The parameters are set as $t_1 = -t_2 = 1$, $\Delta = 0.8$, $\lambda_1 = \lambda_2 = 0.2$.

rameters. If we set $t_1 = -t_2 = 1$ and $\Delta = 1 - \delta$ $(\delta > 0)$ two band inversions are created at (000) and $(\pi\pi\pi)$. The band inversions are stable under spin-orbital-coupling because the spin-orbital-coupling terms vanish at (000) and $(\pi\pi\pi)$. We plot the band structure and density of states with $t_1 = -t_2 = 1$, $\Delta = 0.8$, $\lambda_1 = \lambda_2 = 0.2$ in Supplementary Figure 9. On one hand, the density of states in Supplementary Figure 9b shows that this model is fully gapped at half filling. On the other hand, from the Fu-Kane criterion we get $z_{2\mathrm{w},1} = z_{2\mathrm{w},2} = z_{2\mathrm{w},3} = 1$, $z_4 = 0$. Therefore, this model corresponds to a weak TI with proper parameters.

Supplementary Note 7. A guide for the tables

In this Supplementary Note, we present a users' guide for our main results, i.e., Supplementary Tables 5 and 7, where the independent eLCs and the mapping from SI to TCI invariants in all SGs with nontrivial SI groups are tabulated, respectively. With these data, the diagnosis of topology for a given material reduces to three steps of searching in the "dictionary" (i) applying standard first principle calculations to obtain the occupied BR at high symmetry momenta, (ii) checking whether the band structure is fully gapped, which can be done by either calculating density of states or checking the compatibility relation [11, 18, 19], and (iii) using Supplementary Tables 2 and 3 to calculate the indicators and then looking for the corresponding TCI invariants in Supplementary Table 7. A concrete example of using these tables for SnTe is given in the main text.

As byproducts of our work, the independent eLCs and the possible TCI invariant combinations for SGs with trivial SI groups are tabulated in Supplementary Tables 6 and 8, respectively. Although these states have no indicators and so can not be diagnosed from symmetry data, we think they can yet be regarded as an useful reference for future study in the sense that they are all the possible TCIs that can be realized by layer construction.

In the following we explain the notations in these tables.

eLC. Each eLC is represented by a layer generating it, and the layer notation $(hkl; d)$ consists of Miller indices in *conventional* lattice and its position. General position is represented by the symbol "d_0". Due to the mismatch between conventional and primitive cells, the vector $\mathbf{g} = h\mathbf{G}_1 + k\mathbf{G}_2 + l\mathbf{G}_3$, where $\mathbf{G}_{i=1,2,3}$ are the bases of the conventional reciprocal lattice, may not be the minimal reciprocal lattice in its direction, and the position d is defined with respect to the minimal vector. To be specific, the layer represented by $(hkl; d)$ is given by

$$(hkl; d) = \{\mathbf{r}|\mathbf{r} \cdot \mathbf{g}' = 2\pi(d + q), \quad q \in \mathbb{Z},\ 0 \le d < 1\} \tag{117}$$

where $\mathbf{g}'$ is the minimal reciprocal vector in $\mathbf{g}$'s direction.

SI group. For brevity, the SI group $\mathbb{Z}_p \times \mathbb{Z}_q \times \cdots$ will be written as $\mathbb{Z}_{p,q,\cdots}$.

TCI invariant. In the first row of each SG section we list all the possible TCI invariants, wherein the weak invariants are denoted as "weak", while all the other TCI invariants are represented by the correspinding symmetry elements. It should be noticed that the weak invariants are defined with respect to the primitive cell.

Convention-independent invariant. The convention-independent invariants (see Supplementary Note 2 for detail) for glide, rotation, inversion, screw, and S_4 symmetries are printed in double-stroke font and colored with blue.

Lattice. The lattice setting follows the international table [9, 17], and the convention for primitive cell is tabulated in Supplementary Table 4.

Symmetry element. Mirror plane is denoted as m^{hkl}, glide plane is denoted as $g^{hkl}_{t_1t_2t_3}$, $C_{n=2,4,6}$-rotation axis is denoted as n^{uvw}, inversion center is denoted as i, $C_{n=2,4,6}$-screw axis with a q/n translation along the axis is denoted as n^{uvw}_q, and S_4 center is denoted as $\bar{4}^{uvw}$, Here uvw are the *conventional* indices of the corresponding axis direction, hkl are the *conventional* Miller indices of the corresponding mirror or glide plane, and $t_1t_2t_3$ are the *conventional* coordinates of the corresponding glide vector. In order to keep the table compact, negative indice $-u$ is printed as $\bar{u}$. Analysis in Supplementary Note 2 shows that for identical symmetry elements locating at different positions it is enough to keep only one to define the corresponding TCI invariant. In this work, we take the one with minimal x_1, and if two have same x_1 we take the one with minimal x_2, and then if two have same x_2 we take the one with minimal x_3, where $0 \le x_{i=1,2,3} < 1$ are in *primitive* cell.

Additional symmetry element. Due to the discussion in Supplementary Note 1, screw or glide elements can exist even in symmorphic SGs, which is referred as "additional symmetry elements" in Supplementary Ref. [9]. For example, in SG #12 ($C2/m$), screw axis 2^{010}_1 can be got by the rotation 2^{010} followed by a translation $\mathbf{a}_1$.

Mirror Chern number. As mirror Chern number C_m can be any integer, here we set a cutoff p for each mirror and list only the TCIs with $-p < C_\mathrm{m} \le p$. p is properly chosen such that TCIs with higher C_m can be constructed from the listed TCIs. This cutoff is printed in the subscript of the corresponding mirror element, such as $m^{010}_{(p)}$. There are two kinds of mirror planes as shown in Supplementary Figure 5a,b. For the former we print both $C_{\mathrm{m},0}$ and $C_{\mathrm{m},\pi}$ while for the latter we print only $C_{\mathrm{m},0}$ since $C_{\mathrm{m},\pi}$ always equals to zero. In order to make the table more compact, negative mirror Chern number $-n$ is printed as $\bar{n}$.

Lattice	SGs	$\mathbf{a}_1$, $\mathbf{a}_2$, $\mathbf{a}_3$
Triclinic primitive	1, 2	(100), (010), (001)
Monoclinic primitive	3, 4, 6, 7, 10, 11, 13, 14	(100), (010), (001)
Monoclinic base-centred	5, 8, 9, 12, 15	$(\frac{1}{2}\frac{1}{2}0)$, $(\frac{\bar{1}}{2}\frac{1}{2}0)$, (001)
Orthorhombic primitive	16, 17, 18, 19, 25, 26, 27, 28, 29, 30, 31, 32, 33, 34, 47, 48, 49, 50, 51, 52, 53, 54, 55, 56, 57, 58, 59, 60, 61, 62	(100), (010), (001)
Orthorhombic base-centered	20, 21, 35, 36, 37, 63, 64, 65, 66, 67, 68	$(\frac{1}{2}\frac{1}{2}0)$, $(\frac{\bar{1}}{2}\frac{1}{2}0)$, (001)
	38, 39, 40, 41	(100), $(0\frac{1}{2}\frac{1}{2})$, $(0\frac{\bar{1}}{2}\frac{1}{2})$
Orthorhombic body-centred	23, 24, 44, 45, 46, 71, 72, 73, 74	$(\frac{\bar{1}}{2}\frac{1}{2}\frac{1}{2})$, $(\frac{1}{2}\frac{\bar{1}}{2}\frac{1}{2})$, $(\frac{1}{2}\frac{1}{2}\frac{\bar{1}}{2})$
Orthorhombic face-centred	22, 42, 43, 69, 70	$(0\frac{1}{2}\frac{1}{2})$, $(\frac{1}{2}0\frac{1}{2})$, $(\frac{1}{2}\frac{1}{2}0)$
Tetragonal primitive	75, 76, 77, 78, 81, 83, 84, 85, 86, 89, 90, 91, 92, 93, 94, 95, 96, 99, 100, 101, 102, 103, 104, 105, 106, 111, 112, 113, 114, 115, 116, 117, 118, 123, 124, 125, 126, 127, 128, 129, 130, 131, 132, 133, 134, 135, 136, 137, 138	(100), (010), (001)
Tetragonal body-centred	79, 80, 82, 87, 88, 97, 98, 107, 108, 109, 110, 119, 120, 121, 122, 139, 140, 141, 142	$(\frac{\bar{1}}{2}\frac{1}{2}\frac{1}{2})$, $(\frac{1}{2}\frac{\bar{1}}{2}\frac{1}{2})$, $(\frac{1}{2}\frac{1}{2}\frac{\bar{1}}{2})$
Trigonal primitive	146, 148, 155, 160, 161, 166, 167	$(\frac{2}{3}\frac{1}{3}\frac{1}{3})$, $(\frac{\bar{1}}{3}\frac{1}{3}\frac{1}{3})$, $(\frac{\bar{1}}{3}\frac{\bar{2}}{3}\frac{1}{3})$
Hexagonal primitive	143, 144, 145, 147, 149, 150, 151, 152, 153, 154, 156, 157, 158, 159, 162, 163, 164, 165, 168, 169, 170, 171, 172, 173, 174, 175, 176, 177, 178, 179, 180, 181, 182, 183, 184, 185, 186, 187, 188, 189, 190, 191, 192, 193, 194	(100), (010), (001)
Cubic primitive	195, 198, 200, 201, 205, 207, 208, 212, 213, 215, 218, 221, 222, 223, 224	(100), (010), (001)
Cubic face-centred	196, 202, 203, 209, 210, 216, 219, 225, 226, 227, 228	$(0\frac{1}{2}\frac{1}{2})$, $(\frac{1}{2}0\frac{1}{2})$, $(\frac{1}{2}\frac{1}{2}0)$
Cubic body-centred	197, 199, 204, 206, 211, 214, 217, 220, 229, 230	$(\frac{\bar{1}}{2}\frac{1}{2}\frac{1}{2})$, $(\frac{1}{2}\frac{\bar{1}}{2}\frac{1}{2})$, $(\frac{1}{2}\frac{1}{2}\frac{\bar{1}}{2})$

Supplementary Table 4. The primitive cell settings in SGs with nontrivial TCI SI groups. Settings for centered lattices (base-centered, body-centered, face-centered) follow Supplementary Ref. [9, 17], and the primitive lattice bases are written in the conventional coordinate.

Supplementary Table 5: eLCs in all SGs with nontrivial SI groups.

**eLC**	**SI**	**Invariants**		
		Space group #2 : $P\bar{1}$		
$(hkl;d)$	$\mathbb{Z}_{2,2,2,4}$	weak	i	
$001;0$	0012	001	1	
$001;\frac{1}{2}$	0010	001	0	
$010;0$	0102	010	1	
$100;0$	1002	100	1	

Space group #10 : $P2/m$

$(hkl;d)$	$\mathbb{Z}_{2,2,2,4}$	weak	$m^{010}_{(2)}$	2^{010}	i
$001;0$	0012	001	00	1	1
$001;\frac{1}{2}$	0010	001	00	0	0
$010;0$	0102	010	11	0	1
$010;\frac{1}{2}$	0100	010	$1\bar{1}$	0	0
$100;0$	1002	100	00	1	1

Space group #11 : $P2_1/m$

$(hkl;d)$	$\mathbb{Z}_{2,2,2,4}$	weak	$m^{010}_{(2)}$	i	2_1^{010}
$001;0$	0012	001	00	1	1
$001;\frac{1}{2}$	0010	001	00	0	0
$010;\frac{1}{4}$	0000	000	20	0	1
$100;0$	1002	100	00	1	1

Space group #12 : $C2/m$

$(hkl;d)$	$\mathbb{Z}_{2,2,2,4}$	weak	$m^{010}_{(2)}$	$g^{010}_{\frac{1}{2}00}$	2^{010}	i	2_1^{010}
$001;0$	0012	001	0	0	1	1	1
$001;\frac{1}{2}$	0010	001	0	0	0	0	0
$\bar{1}10;0$	1100	110	0	1	0	0	1
$010;0$	1102	110	2	0	0	1	1

Space group #13 : $P2/c$

$(hkl;d)$	$\mathbb{Z}_{2,2,2,4}$	weak	$g^{010}_{00\frac{1}{2}}$	2^{010}	i
$001;\frac{1}{4}$	0000	000	1	1	0
$001;0$	0002	000	1	0	1
$010;0$	0102	010	1	0	1
$100;0$	1002	100	0	1	1

Space group #14 : $P2_1/c$

$(hkl;d)$	$\mathbb{Z}_{2,2,2,4}$	weak	$g^{010}_{00\frac{1}{2}}$	i	2_1^{010}
$001;\frac{1}{4}$	0000	000	1	0	1
$001;0$	0002	000	1	1	0
$100;0$	1002	100	0	1	1

Space group #15 : $C2/c$

$(hkl;d)$	$\mathbb{Z}_{2,2,2,4}$	weak	$g^{010}_{00\frac{1}{2}}$	$g^{010}_{\frac{1}{2}0\frac{1}{2}}$	2^{010}	i	2_1^{010}
$001;\frac{1}{4}$	0000	000	1	1	1	0	1
$001;0$	0002	000	1	1	0	1	0
$\bar{1}10;0$	1100	110	0	1	0	0	1

Space group #47 : $Pmmm$

$(hkl;d)$	$\mathbb{Z}_{2,2,2,4}$	weak	$m^{001}_{(2)}$	$m^{010}_{(2)}$	$m^{100}_{(2)}$	2^{001}	2^{010}	2^{100}	i
$001;0$	0012	001	11	00	00	0	1	1	1
$001;\frac{1}{2}$	0010	001	$1\bar{1}$	00	00	0	0	0	0
$010;0$	0102	010	00	11	00	1	0	1	1
$010;\frac{1}{2}$	0100	010	00	$1\bar{1}$	00	0	0	0	0
$100;0$	1002	100	00	00	11	1	1	0	1
$100;\frac{1}{2}$	1000	100	00	00	$1\bar{1}$	0	0	0	0

Space group #48 : $Pnnn$

$(hkl;d)$	$\mathbb{Z}_{2,2,2,4}$	weak	$g^{001}_{\frac{1}{2}\frac{1}{2}0}$	$g^{010}_{\frac{1}{2}0\frac{1}{2}}$	$g^{100}_{0\frac{1}{2}\frac{1}{2}}$	2^{001}	2^{010}	2^{100}	i
$001;\frac{1}{4}$	0000	000	0	1	1	0	1	1	0
$001;0$	0002	000	1	1	1	0	0	0	1
$010;\frac{1}{4}$	0000	000	1	0	1	1	0	1	0

Space group #49 : $Pccm$

Continued on next page

Supplementary Table 5 – continued

eLC	SI	Invariants							
$(hkl;d)$	$\mathbb{Z}_{2,2,2,4}$	weak	$m^{001}_{(2)}$	$g^{010}_{00\frac{1}{2}}$	$g^{100}_{00\frac{1}{2}}$	2^{001}	2^{010}	2^{100}	i
$001;\frac{1}{4}$	0000	000	00	1	1	0	1	1	0
$001;0$	0002	000	20	1	1	0	0	0	1
$010;0$	0102	010	00	1	0	1	0	1	1
$010;\frac{1}{2}$	0100	010	00	0	0	0	0	0	0
$100;0$	1002	100	00	0	1	1	1	0	1
Space group #50 : *Pban*									
$(hkl;d)$	$\mathbb{Z}_{2,2,2,4}$	weak	$g^{001}_{\frac{1}{2}\frac{1}{2}0}$	$g^{010}_{\frac{1}{2}00}$	$g^{100}_{0\frac{1}{2}0}$	2^{001}	2^{010}	2^{100}	i
$001;0$	0012	001	1	0	0	0	1	1	1
$001;\frac{1}{2}$	0010	001	0	0	0	0	0	0	0
$010;\frac{1}{4}$	0000	000	1	0	1	1	0	1	0
$010;0$	0002	000	1	1	1	0	0	0	1
Space group #51 : *Pmma*									
$(hkl;d)$	$\mathbb{Z}_{2,2,2,4}$	weak	$m^{010}_{(2)}$	$m^{100}_{(2)}$	$g^{001}_{\frac{1}{2}00}$	2^{001}	2^{010}	i	2^{100}_{1}
$001;0$	0012	001	00	00	1	0	1	1	1
$001;\frac{1}{2}$	0010	001	00	00	0	0	0	0	0
$010;0$	0102	010	11	00	0	1	0	1	1
$010;\frac{1}{2}$	0100	010	$1\bar{1}$	00	0	0	0	0	0
$100;\frac{1}{4}$	0000	000	00	20	1	1	0	0	1
Space group #52 : *Pnna*									
$(hkl;d)$	$\mathbb{Z}_{2,2,2,4}$	weak	$g^{001}_{\frac{1}{2}00}$	$g^{010}_{\frac{1}{2}0\frac{1}{2}}$	$g^{100}_{0\frac{1}{2}\frac{1}{2}}$	2^{001}	2^{100}	i	2^{010}_{1}
$001;\frac{1}{4}$	0000	000	0	1	1	0	1	0	1
$001;0$	0002	000	1	1	1	0	0	1	0
$010;0$	0002	000	0	0	1	1	0	1	1
Space group #53 : *Pmna*									
$(hkl;d)$	$\mathbb{Z}_{2,2,2,4}$	weak	$m^{100}_{(2)}$	$g^{001}_{\frac{1}{2}00}$	$g^{010}_{\frac{1}{2}0\frac{1}{2}}$	2^{010}	2^{100}	i	2^{001}_{1}
$001;\frac{1}{4}$	0000	000	00	1	1	1	0	0	1
$001;0$	0002	000	00	0	1	0	1	1	1
$010;0$	0102	010	00	0	1	0	1	1	1
$100;0$	0002	000	20	1	1	0	0	1	0
Space group #54 : *Pcca*									
$(hkl;d)$	$\mathbb{Z}_{2,2,2,4}$	weak	$g^{001}_{\frac{1}{2}00}$	$g^{010}_{00\frac{1}{2}}$	$g^{100}_{00\frac{1}{2}}$	2^{001}	2^{010}	i	2^{100}_{1}
$001;\frac{1}{4}$	0000	000	0	1	1	0	1	0	1
$001;0$	0002	000	1	1	1	0	0	1	0
$010;0$	0102	010	0	1	0	1	0	1	1
$010;\frac{1}{2}$	0100	010	0	0	0	0	0	0	0
Space group #55 : *Pbam*									
$(hkl;d)$	$\mathbb{Z}_{2,2,2,4}$	weak	$m^{001}_{(2)}$	$g^{010}_{\frac{1}{2}00}$	$g^{100}_{0\frac{1}{2}0}$	2^{001}	i	2^{010}_{1}	2^{100}_{1}
$001;0$	0012	001	11	0	0	0	1	1	1
$001;\frac{1}{2}$	0010	001	$1\bar{1}$	0	0	0	0	0	0
$010;0$	0002	000	00	0	1	1	1	1	0
$010;\frac{1}{4}$	0000	000	00	1	1	0	0	1	1
Space group #56 : *Pccn*									
$(hkl;d)$	$\mathbb{Z}_{2,2,2,4}$	weak	$g^{001}_{\frac{1}{2}\frac{1}{2}0}$	$g^{010}_{00\frac{1}{2}}$	$g^{100}_{00\frac{1}{2}}$	2^{001}	i	2^{010}_{1}	2^{100}_{1}
$001;\frac{1}{4}$	0000	000	0	1	1	0	0	1	1
$001;0$	0002	000	1	1	1	0	1	0	0
$010;\frac{1}{4}$	0000	000	1	1	0	1	0	1	0
Space group #57 : *Pbcm*									
$(hkl;d)$	$\mathbb{Z}_{2,2,2,4}$	weak	$m^{001}_{(2)}$	$g^{010}_{00\frac{1}{2}}$	$g^{100}_{0\frac{1}{2}0}$	2^{100}	i	2^{001}_{1}	2^{010}_{1}
$001;\frac{1}{4}$	0000	000	20	1	0	0	0	1	1
$001;0$	0002	000	00	1	0	1	1	1	0
$010;0$	0002	000	00	0	1	0	1	1	1
$100;0$	1002	100	00	0	1	0	1	1	1
Space group #58 : *Pnnm*									

Continued on next page

Supplementary Table 5 – continued

eLC	SI	Invariants											
$(hkl;d)$	$\mathbb{Z}_{2,2,2,4}$	weak	$m^{001}_{(2)}$	$g^{010}_{\frac{1}{2}0\frac{1}{2}}$	$g^{100}_{0\frac{1}{2}\frac{1}{2}}$	2^{001}	i	2^{010}_1	2^{100}_1				
$001;\frac{1}{4}$	0000	000	00	1	1	0	0	1	1				
$001;0$	0002	000	20	1	1	0	1	0	0				
$010;0$	0002	000	00	0	1	1	1	1	0				
Space group #59 : *Pmmn*													
$(hkl;d)$	$\mathbb{Z}_{2,2,2,4}$	weak	$m^{010}_{(2)}$	$m^{100}_{(2)}$	$g^{001}_{\frac{1}{2}\frac{1}{2}0}$	2^{001}	i	2^{010}_1	2^{100}_1				
$001;0$	0012	001	00	00	1	0	1	1	1				
$001;\frac{1}{2}$	0010	001	00	00	0	0	0	0	0				
$010;\frac{1}{4}$	0000	000	20	00	1	1	0	1	0				
$100;\frac{1}{4}$	0000	000	00	20	1	1	0	0	1				
Space group #60 : *Pbcn*													
$(hkl;d)$	$\mathbb{Z}_{2,2,2,4}$	weak	$g^{001}_{\frac{1}{2}\frac{1}{2}0}$	$g^{010}_{00\frac{1}{2}}$	$g^{100}_{0\frac{1}{2}0}$	2^{010}	i	2^{001}_1	2^{100}_1				
$001;\frac{1}{4}$	0000	000	1	1	0	1	0	1	0				
$001;0$	0002	000	0	1	0	0	1	1	1				
$010;\frac{1}{4}$	0000	000	1	0	1	0	0	1	1				
Space group #61 : *Pbca*													
$(hkl;d)$	$\mathbb{Z}_{2,2,2,4}$	weak	$g^{001}_{\frac{1}{2}00}$	$g^{010}_{00\frac{1}{2}}$	$g^{100}_{0\frac{1}{2}0}$	i	2^{001}_1	2^{010}_1	2^{100}_1				
$001;\frac{1}{4}$	0000	000	1	1	0	0	1	1	0				
$001;0$	0002	000	0	1	0	1	1	0	1				
$010;0$	0002	000	0	0	1	1	1	1	0				
Space group #62 : *Pnma*													
$(hkl;d)$	$\mathbb{Z}_{2,2,2,4}$	weak	$m^{010}_{(2)}$	$g^{001}_{\frac{1}{2}00}$	$g^{100}_{0\frac{1}{2}\frac{1}{2}}$	i	2^{001}_1	2^{010}_1	2^{100}_1				
$001;0$	0002	000	00	0	1	1	1	1	0				
$001;\frac{1}{4}$	0000	000	00	1	1	0	1	0	1				
$010;\frac{1}{4}$	0000	000	20	0	1	0	0	1	1				
Space group #63 : *Cmcm*													
$(hkl;d)$	$\mathbb{Z}_{2,2,2,4}$	weak	$m^{001}_{(2)}$	$m^{100}_{(2)}$	$g^{010}_{00\frac{1}{2}}$	$g^{010}_{\frac{\bar{1}}{2}0\frac{1}{2}}$	$g^{100}_{0\frac{1}{2}0}$	2^{010}	2^{100}	i	2^{001}_1	2^{010}_1	2^{100}_1
$001;\frac{1}{4}$	0000	000	20	0	1	1	0	1	0	0	1	1	0
$001;0$	0002	000	00	0	1	1	0	0	1	1	1	0	1
$\bar{1}10;0$	1100	110	00	0	0	1	1	0	0	0	0	1	1
$100;0$	1102	110	00	2	0	1	0	1	0	1	1	0	1
Space group #64 : *Cmce*													
$(hkl;d)$	$\mathbb{Z}_{2,2,2,4}$	weak	$m^{100}_{(2)}$	$g^{001}_{0\frac{1}{2}0}$	$g^{010}_{00\frac{1}{2}}$	$g^{010}_{\frac{\bar{1}}{2}0\frac{1}{2}}$	$g^{100}_{0\frac{1}{2}0}$	2^{010}	2^{100}	i	2^{001}_1	2^{010}_1	2^{100}_1
$001;\frac{1}{4}$	0000	000	0	1	1	1	0	1	0	0	1	1	0
$001;0$	0002	000	0	0	1	1	0	0	1	1	1	0	1
$010;0$	1102	110	0	1	0	1	1	0	1	1	0	1	0
$100;0$	1102	110	2	0	0	1	0	0	0	1	1	1	1
Space group #65 : *Cmmm*													
$(hkl;d)$	$\mathbb{Z}_{2,2,2,4}$	weak	$m^{001}_{(2)}$	$m^{010}_{(2)}$	$m^{100}_{(2)}$	$g^{010}_{\frac{\bar{1}}{2}00}$	$g^{100}_{0\frac{1}{2}0}$	2^{001}	2^{010}	2^{100}	i	2^{010}_1	2^{100}_1
$001;0$	0012	001	11	0	0	0	0	0	1	1	1	1	1
$001;\frac{1}{2}$	0010	001	$1\bar{1}$	0	0	0	0	0	0	0	0	0	0
$\bar{1}10;0$	1100	110	00	0	0	1	1	0	0	0	0	1	1
$010;0$	1102	110	00	2	0	0	1	1	0	1	1	1	0
$100;0$	1102	110	00	0	2	1	0	1	1	0	1	0	1
Space group #66 : *Cccm*													
$(hkl;d)$	$\mathbb{Z}_{2,2,2,4}$	weak	$m^{001}_{(2)}$	$g^{010}_{00\frac{1}{2}}$	$g^{010}_{\frac{\bar{1}}{2}0\frac{1}{2}}$	$g^{100}_{00\frac{1}{2}}$	$g^{100}_{0\frac{1}{2}\frac{1}{2}}$	2^{001}	2^{010}	2^{100}	i	2^{010}_1	2^{100}_1
$001;\frac{1}{4}$	0000	000	00	1	1	1	1	0	1	1	0	1	1
$001;0$	0002	000	20	1	1	1	1	0	0	0	1	0	0
$\bar{1}10;0$	1100	110	00	0	1	0	1	0	0	0	0	1	1
$010;0$	1102	110	00	1	0	0	1	1	0	1	1	1	0
Space group #67 : *Cmme*													
$(hkl;d)$	$\mathbb{Z}_{2,2,2,4}$	weak	$m^{010}_{(2)}$	$m^{100}_{(2)}$	$g^{001}_{0\frac{1}{2}0}$	$g^{010}_{\frac{\bar{1}}{2}00}$	$g^{100}_{0\frac{1}{2}0}$	2^{001}	2^{010}	2^{100}	i	2^{010}_1	2^{100}_1
$001;0$	0012	001	0	0	1	0	0	0	1	1	1	1	1
$001;\frac{1}{2}$	0010	001	0	0	0	0	0	0	0	0	0	0	0

Continued on next page

Supplementary Table 5 – continued

eLC	SI	Invariants										
010; 0	1102	110	0	0	1	1	1	0	0	1	1	1	0
010; $\frac{1}{2}$	1100	110	2	0	1	0	1	1	0	0	0	1	1
100; 0	1102	110	0	2	0	1	0	1	0	0	1	1	1

Space group #68 : $Ccce$

$(hkl;d)$	$\mathbb{Z}_{2,2,2,4}$	weak	$g^{001}_{0\frac{1}{2}0}$	$g^{010}_{00\frac{1}{2}}$	$g^{010}_{\frac{1}{2}0\frac{1}{2}}$	$g^{100}_{00\frac{1}{2}}$	$g^{100}_{0\frac{1}{2}\frac{1}{2}}$	2^{001}	2^{010}	2^{100}	i	2^{010}_1	2^{100}_1
001; $\frac{1}{4}$	0000	000	0	1	1	1	1	0	1	1	0	1	1
001; 0	0002	000	1	1	1	1	1	0	0	0	1	0	0
010; 0	1102	110	1	1	0	0	1	0	0	0	1	1	1
010; $\frac{1}{2}$	1100	110	1	0	1	0	1	1	0	1	0	1	0

Space group #69 : $Fmmm$

$(hkl;d)$	$\mathbb{Z}_{2,2,2,4}$	weak	$m^{001}_{(2)}$	$m^{010}_{(2)}$	$m^{100}_{(2)}$	$g^{001}_{\frac{1}{2}00}$	$g^{010}_{\frac{1}{2}00}$	$g^{100}_{0\frac{1}{2}0}$	2^{001}	2^{010}	2^{100}	i	2^{001}_1	2^{010}_1	2^{100}_1
100; 0	0112	011	0	0	2	1	1	0	1	1	0	1	1	1	1
100; $\frac{1}{2}$	0110	011	0	0	0	1	1	1	0	0	0	0	0	0	1
010; 0	1012	101	0	2	0	0	0	1	1	0	1	1	0	1	1
010; $\frac{1}{2}$	1010	101	0	0	0	0	1	1	0	0	0	0	1	1	0
001; 0	1102	110	2	0	0	0	0	0	0	1	1	1	1	0	0

Space group #70 : $Fddd$

$(hkl;d)$	$\mathbb{Z}_{2,2,2,4}$	weak	$g^{001}_{\frac{1}{4}\frac{1}{4}0}$	$g^{001}_{\frac{1}{4}\frac{\bar{1}}{4}0}$	$g^{010}_{\frac{1}{4}0\frac{\bar{1}}{4}}$	$g^{010}_{\frac{1}{4}0\frac{1}{4}}$	$g^{100}_{0\frac{1}{4}\frac{1}{4}}$	$g^{100}_{0\frac{1}{4}\frac{\bar{1}}{4}}$	2^{001}	2^{010}	2^{100}	i	2^{001}_1	2^{010}_1	2^{100}_1
$11\bar{1}$; 0	0002	000	1	1	1	1	1	1	0	0	0	1	0	0	0
100; $\frac{1}{4}$	0000	000	1	1	1	1	0	0	1	1	0	0	1	1	0
010; $\frac{1}{4}$	0000	000	1	1	0	0	1	1	1	0	1	0	1	0	1

Space group #71 : $Immm$

$(hkl;d)$	$\mathbb{Z}_{2,2,2,4}$	weak	$m^{001}_{(2)}$	$m^{010}_{(2)}$	$m^{100}_{(2)}$	$g^{001}_{\frac{1}{2}\frac{1}{2}0}$	$g^{010}_{\frac{1}{2}0\frac{1}{2}}$	$g^{100}_{0\frac{1}{2}\frac{1}{2}}$	2^{001}	2^{010}	2^{100}	i	2^{001}_1	2^{010}_1	2^{100}_1
110; 0	1110	111	0	0	0	1	1	1	0	0	0	0	1	1	1
001; 0	1112	111	2	0	0	0	1	1	0	1	1	1	1	0	0
010; 0	1112	111	0	2	0	1	0	1	1	0	1	1	0	1	0
$\bar{1}00$; 0	1112	111	0	0	2	1	1	0	1	1	0	1	0	0	1

Space group #72 : $Ibam$

$(hkl;d)$	$\mathbb{Z}_{2,2,2,4}$	weak	$m^{001}_{(2)}$	$g^{001}_{\frac{1}{2}\frac{1}{2}0}$	$g^{010}_{00\frac{1}{2}}$	$g^{010}_{\frac{1}{2}00}$	$g^{100}_{00\frac{1}{2}}$	$g^{100}_{0\frac{1}{2}0}$	2^{001}	2^{010}	2^{100}	i	2^{001}_1	2^{010}_1	2^{100}_1
110; 0	1110	111	0	1	0	1	0	1	0	0	0	0	1	1	1
001; 0	1112	111	2	0	1	0	1	0	0	0	0	1	1	1	1
001; $\frac{1}{2}$	1110	111	0	1	1	0	1	0	0	1	1	0	1	0	0
010; 0	1112	111	0	1	1	0	0	1	1	0	1	1	0	1	0

Space group #73 : $Ibca$

$(hkl;d)$	$\mathbb{Z}_{2,2,2,4}$	weak	$g^{001}_{0\frac{1}{2}0}$	$g^{001}_{\frac{1}{2}00}$	$g^{010}_{00\frac{1}{2}}$	$g^{010}_{\frac{1}{2}00}$	$g^{100}_{00\frac{1}{2}}$	$g^{100}_{0\frac{1}{2}0}$	2^{001}	2^{010}	2^{100}	i	2^{001}_1	2^{010}_1	2^{100}_1
001; 0	1112	111	1	0	1	0	1	0	0	1	0	1	1	0	1
001; $\frac{1}{2}$	1110	111	0	1	1	0	1	0	0	0	1	0	1	1	0
010; 0	1112	111	1	0	0	1	0	1	0	0	1	1	1	1	0
010; $\frac{1}{2}$	1110	111	1	0	1	0	0	1	1	0	0	0	0	1	1

Space group #74 : $Imma$

$(hkl;d)$	$\mathbb{Z}_{2,2,2,4}$	weak	$m^{010}_{(2)}$	$m^{100}_{(2)}$	$g^{001}_{0\frac{1}{2}0}$	$g^{001}_{\frac{1}{2}00}$	$g^{010}_{\frac{1}{2}0\frac{1}{2}}$	$g^{100}_{0\frac{1}{2}\frac{1}{2}}$	2^{001}	2^{010}	2^{100}	i	2^{001}_1	2^{010}_1	2^{100}_1
101; 0	1110	111	0	0	0	1	1	1	0	1	0	0	1	0	1
$0\bar{1}1$; 0	1112	111	0	0	1	0	1	1	0	0	1	1	1	1	0
010; $\frac{1}{2}$	1110	111	2	0	1	0	0	1	1	0	0	0	0	1	1
$\bar{1}00$; 0	1112	111	0	2	0	1	1	0	1	0	0	1	0	1	1

Space group #83 : $P4/m$

$(hkl;d)$	$\mathbb{Z}_{2,4,8}$	weak	$m^{001}_{(4)}$	2^{001}	4^{001}	i	$\bar{4}^{001}$
001; 0	012	001	11	0	0	1	1
001; $\frac{1}{2}$	030	001	$1\bar{1}$	0	0	0	0
010; 0	104	110	00	0	1	0	1
010; $\frac{1}{2}$	100	110	00	0	0	0	0

Space group #84 : $P4_2/m$

$(hkl;d)$	$\mathbb{Z}_{2,2,2,4}$	weak	$m^{001}_{(4)}$	2^{001}	i	4^{001}_2	$\bar{4}^{001}$
001; $\frac{1}{4}$	0000	000	00	0	0	1	1
001; 0	0002	000	20	0	1	1	0

Continued on next page

Supplementary Table 5 – continued

**eLC**	**SI**	**Invariants**					
010; 0	1100	110	00	0	0	1	1

Space group #85 : $P4/n$

$(hkl;d)$	$\mathbb{Z}_{2,2,2,4}$	weak	$g^{001}_{\frac{1}{2}\frac{1}{2}0}$	2^{001}	4^{001}	i	$\bar{4}^{001}$
001; 0	0012	001	1	0	0	1	1
001; $\frac{1}{2}$	0010	001	0	0	0	0	0
010; $\frac{1}{4}$	0000	000	0	0	1	0	1

Space group #86 : $P4_2/n$

$(hkl;d)$	$\mathbb{Z}_{2,2,2,4}$	weak	$g^{001}_{\frac{1}{2}\frac{1}{2}0}$	2^{001}	i	4^{001}_2	$\bar{4}^{001}$
001; $\frac{1}{4}$	0000	000	0	0	0	1	1
001; 0	0002	000	1	0	1	1	0

Space group #87 : $I4/m$

$(hkl;d)$	$\mathbb{Z}_{2,8}$	weak	$m^{001}_{(4)}$	$g^{001}_{\frac{1}{2}\frac{1}{2}0}$	2^{001}	4^{001}	i	2^{001}_1	4^{001}_2	$\bar{4}^{001}$
110; 0	14	111	0	1	0	1	0	1	0	1
110; $\frac{1}{2}$	10	111	0	1	0	0	0	1	1	0
001; 0	12	111	2	0	0	0	1	1	1	1

Space group #88 : $I4_1/a$

$(hkl;d)$	$\mathbb{Z}_{2,2,2,4}$	weak	$g^{001}_{0\frac{1}{2}0}$	$g^{001}_{\frac{1}{2}00}$	2^{001}	i	2^{001}_1	4^{001}_1	4^{001}_3	$\bar{4}^{001}$
110; $\frac{1}{4}$	0000	000	0	0	0	0	0	1	1	1
101; 0	0002	000	1	1	0	1	0	1	1	0

Space group #123 : $P4/mmm$

$(hkl;d)$	$\mathbb{Z}_{2,4,8}$	weak	$m^{001}_{(4)}$	$m^{1\bar{1}0}_{(2)}$	$m^{100}_{(2)}$	$g^{1\bar{1}0}_{\frac{1}{2}\frac{1}{2}0}$	2^{001}	2^{100}	2^{110}	4^{001}	i	2^{110}_1	$\bar{4}^{001}$
001; 0	012	001	11	0	00	0	0	1	1	0	1	0	1
001; $\frac{1}{2}$	030	001	$1\bar{1}$	0	00	0	0	0	0	0	0	1	0
010; 0	104	110	00	0	11	1	0	1	0	1	0	1	1
010; $\frac{1}{2}$	100	110	00	0	$1\bar{1}$	1	0	0	0	0	0	1	0
110; 0	004	000	00	2	00	1	0	0	1	1	0	1	1

Space group #124 : $P4/mcc$

$(hkl;d)$	$\mathbb{Z}_{2,8}$	weak	$m^{001}_{(4)}$	$g^{1\bar{1}0}_{00\frac{1}{2}}$	$g^{1\bar{1}0}_{\frac{1}{2}\frac{1}{2}\frac{1}{2}}$	$g^{100}_{00\frac{1}{2}}$	2^{001}	2^{100}	2^{110}	4^{001}	i	2^{110}_1	$\bar{4}^{001}$
001; $\frac{1}{4}$	00	000	00	1	1	1	0	1	1	0	0	1	0
001; 0	02	000	20	1	1	1	0	0	0	0	1	0	1
010; 0	14	110	00	0	1	1	0	1	0	1	0	1	1
010; $\frac{1}{2}$	10	110	00	0	1	0	0	0	0	0	0	1	0

Space group #125 : $P4/nbm$

$(hkl;d)$	$\mathbb{Z}_{2,2,2,4}$	weak	$m^{1\bar{1}0}_{(2)}$	$g^{001}_{\frac{1}{2}\frac{1}{2}0}$	$g^{1\bar{1}0}_{\frac{1}{2}\frac{1}{2}0}$	$g^{100}_{0\frac{1}{2}0}$	2^{001}	2^{100}	2^{110}	4^{001}	i	2^{110}_1	$\bar{4}^{001}$
001; 0	0012	001	0	1	0	0	0	1	1	0	1	0	1
001; $\frac{1}{2}$	0010	001	0	0	0	0	0	0	0	0	0	1	0
010; $\frac{1}{4}$	0000	000	0	0	0	1	0	1	0	1	0	0	1
110; 0	0002	000	2	1	1	1	0	0	0	0	1	0	1

Space group #126 : $P4/nnc$

$(hkl;d)$	$\mathbb{Z}_{2,2,2,4}$	weak	$g^{001}_{\frac{1}{2}\frac{1}{2}0}$	$g^{1\bar{1}0}_{00\frac{1}{2}}$	$g^{1\bar{1}0}_{\frac{1}{2}\frac{1}{2}\frac{1}{2}}$	$g^{100}_{0\frac{1}{2}\frac{1}{2}}$	2^{001}	2^{100}	2^{110}	4^{001}	i	2^{110}_1	$\bar{4}^{001}$
001; $\frac{1}{4}$	0000	000	0	1	1	1	0	1	1	0	0	1	0
001; 0	0002	000	1	1	1	1	0	0	0	0	1	0	1
010; $\frac{1}{4}$	0000	000	0	0	0	1	0	1	0	1	0	0	1

Space group #127 : $P4/mbm$

$(hkl;d)$	$\mathbb{Z}_{4,8}$	weak	$m^{001}_{(4)}$	$m^{1\bar{1}0}_{(2)}$	$g^{1\bar{1}0}_{\frac{1}{2}\frac{1}{2}0}$	$g^{100}_{0\frac{1}{2}0}$	2^{001}	2^{110}	4^{001}	i	2^{100}_1	2^{110}_1	$\bar{4}^{001}$
001; 0	12	001	11	0	0	0	0	0	0	1	1	1	1
001; $\frac{1}{2}$	30	001	$1\bar{1}$	0	0	0	0	1	0	0	0	0	0
010; 0	04	000	00	0	0	1	0	0	1	0	1	0	1
110; $\frac{1}{2}$	00	000	00	2	1	1	0	1	0	0	1	1	0

Space group #128 : $P4/mnc$

$(hkl;d)$	$\mathbb{Z}_{8}$	weak	$m^{001}_{(4)}$	$g^{1\bar{1}0}_{00\frac{1}{2}}$	$g^{1\bar{1}0}_{\frac{1}{2}\frac{1}{2}\frac{1}{2}}$	$g^{100}_{0\frac{1}{2}\frac{1}{2}}$	2^{001}	2^{110}	4^{001}	i	2^{100}_1	2^{110}_1	$\bar{4}^{001}$
001; $\frac{1}{4}$	0	000	00	1	1	1	0	1	0	0	1	1	0
001; 0	2	000	20	1	1	1	0	0	0	1	0	0	1

Continued on next page

Supplementary Table 5 – continued

**eLC**	**SI**	**Invariants**											
$010;0$	4	000	00	0	0	1	0	0	1	0	1	0	1
		Space group #129 : $P4/nmm$											
$(hkl;d)$	$\mathbb{Z}_{2,2,2,4}$	weak	$m_{(2)}^{1\bar{1}0}$	$m_{(2)}^{100}$	$g_{\frac{1}{2}\frac{1}{2}0}^{001}$	$g_{\frac{1}{2}\frac{1}{2}0}^{1\bar{1}0}$	2^{001}	2^{110}	4^{001}	i	2_1^{100}	2_1^{110}	$\bar{4}^{001}$
$001;0$	0012	001	0	00	1	0	0	0	0	1	1	1	1
$001;\frac{1}{2}$	0010	001	0	00	0	0	0	1	0	0	0	0	0
$010;\frac{1}{4}$	0000	000	0	20	0	0	0	0	1	0	1	0	1
$110;\frac{1}{2}$	0002	000	2	00	1	1	0	0	1	1	1	0	0
		Space group #130 : $P4/ncc$											
$(hkl;d)$	$\mathbb{Z}_{2,2,2,4}$	weak	$g_{\frac{1}{2}\frac{1}{2}0}^{001}$	$g_{00\frac{1}{2}}^{1\bar{1}0}$	$g_{\frac{1}{2}\frac{1}{2}\frac{1}{2}}^{1\bar{1}0}$	$g_{00\frac{1}{2}}^{100}$	2^{001}	2^{110}	4^{001}	i	2_1^{100}	2_1^{110}	$\bar{4}^{001}$
$001;\frac{1}{4}$	0000	000	0	1	1	1	0	1	0	0	1	1	0
$001;0$	0002	000	1	1	1	1	0	0	0	1	0	0	1
$010;\frac{1}{4}$	0000	000	0	0	0	1	0	0	1	0	1	0	1
		Space group #131 : $P4_2/mmc$											
$(hkl;d)$	$\mathbb{Z}_{2,2,2,4}$	weak	$m_{(4)}^{001}$	$m_{(2)}^{100}$	$g_{00\frac{1}{2}}^{1\bar{1}0}$	$g_{\frac{1}{2}\frac{1}{2}\frac{1}{2}}^{1\bar{1}0}$	2^{001}	2^{100}	2^{110}	i	2_1^{110}	4_2^{001}	$\bar{4}^{001}$
$001;0$	0002	000	20	00	1	1	0	1	0	1	0	1	0
$001;\frac{1}{4}$	0000	000	00	00	1	1	0	0	1	0	1	1	1
$010;0$	1100	110	00	11	0	1	0	1	0	0	1	1	1
$010;\frac{1}{2}$	1100	110	00	$1\bar{1}$	0	1	0	0	0	0	1	0	0
		Space group #132 : $P4_2/mcm$											
$(hkl;d)$	$\mathbb{Z}_{2,2,2,4}$	weak	$m_{(4)}^{001}$	$m_{(2)}^{1\bar{1}0}$	$g_{\frac{1}{2}\frac{1}{2}0}^{1\bar{1}0}$	$g_{00\frac{1}{2}}^{100}$	2^{001}	2^{100}	2^{110}	i	2_1^{110}	4_2^{001}	$\bar{4}^{001}$
$001;\frac{1}{4}$	0000	000	00	0	0	1	0	1	0	0	0	1	1
$001;0$	0002	000	20	0	0	1	0	0	1	1	1	1	0
$010;0$	1100	110	00	0	1	1	0	1	0	0	1	1	1
$110;0$	0000	000	00	2	1	0	0	0	1	0	1	1	1
		Space group #133 : $P4_2/nbc$											
$(hkl;d)$	$\mathbb{Z}_{2,2,2,4}$	weak	$g_{\frac{1}{2}\frac{1}{2}0}^{001}$	$g_{00\frac{1}{2}}^{1\bar{1}0}$	$g_{\frac{1}{2}\frac{1}{2}\frac{1}{2}}^{1\bar{1}0}$	$g_{0\frac{1}{2}0}^{100}$	2^{001}	2^{100}	2^{110}	i	2_1^{110}	4_2^{001}	$\bar{4}^{001}$
$001;0$	0002	000	1	1	1	0	0	1	0	1	0	1	0
$001;\frac{1}{4}$	0000	000	0	1	1	0	0	0	1	0	1	1	1
$010;\frac{1}{4}$	0000	000	0	0	0	1	0	1	0	0	0	1	1
		Space group #134 : $P4_2/nnm$											
$(hkl;d)$	$\mathbb{Z}_{2,2,2,4}$	weak	$m_{(2)}^{1\bar{1}0}$	$g_{\frac{1}{2}\frac{1}{2}0}^{001}$	$g_{\frac{1}{2}\frac{1}{2}0}^{1\bar{1}0}$	$g_{0\frac{1}{2}\frac{1}{2}}^{100}$	2^{001}	2^{100}	2^{110}	i	2_1^{110}	4_2^{001}	$\bar{4}^{001}$
$001;\frac{1}{4}$	0000	000	0	0	0	1	0	1	0	0	0	1	1
$001;0$	0002	000	0	1	0	1	0	0	1	1	1	1	0
$110;0$	0002	000	2	1	1	1	0	0	0	1	0	0	1
		Space group #135 : $P4_2/mbc$											
$(hkl;d)$	$\mathbb{Z}_{2,2,2,4}$	weak	$m_{(4)}^{001}$	$g_{00\frac{1}{2}}^{1\bar{1}0}$	$g_{\frac{1}{2}\frac{1}{2}\frac{1}{2}}^{1\bar{1}0}$	$g_{0\frac{1}{2}0}^{100}$	2^{001}	2^{110}	i	2_1^{100}	2_1^{110}	4_2^{001}	$\bar{4}^{001}$
$001;0$	0002	000	20	1	1	0	0	0	1	1	0	1	0
$001;\frac{1}{4}$	0000	000	00	1	1	0	0	1	0	0	1	1	1
$010;0$	0000	000	00	0	0	1	0	0	0	1	0	1	1
		Space group #136 : $P4_2/mnm$											
$(hkl;d)$	$\mathbb{Z}_{2,2,2,4}$	weak	$m_{(4)}^{001}$	$m_{(2)}^{1\bar{1}0}$	$g_{\frac{1}{2}\frac{1}{2}0}^{1\bar{1}0}$	$g_{0\frac{1}{2}\frac{1}{2}}^{100}$	2^{001}	2^{110}	i	2_1^{100}	2_1^{110}	4_2^{001}	$\bar{4}^{001}$
$001;\frac{1}{4}$	0000	000	00	0	0	1	0	0	0	1	0	1	1
$001;0$	0002	000	20	0	0	1	0	1	1	0	1	1	0
$110;0$	0000	000	00	2	1	1	0	1	0	1	1	0	0
		Space group #137 : $P4_2/nmc$											
$(hkl;d)$	$\mathbb{Z}_{2,2,2,4}$	weak	$m_{(2)}^{100}$	$g_{\frac{1}{2}\frac{1}{2}0}^{001}$	$g_{00\frac{1}{2}}^{1\bar{1}0}$	$g_{\frac{1}{2}\frac{1}{2}\frac{1}{2}}^{1\bar{1}0}$	2^{001}	2^{110}	i	2_1^{100}	2_1^{110}	4_2^{001}	$\bar{4}^{001}$
$001;0$	0002	000	00	1	1	1	0	0	1	1	0	1	0
$001;\frac{1}{4}$	0000	000	00	0	1	1	0	1	0	0	1	1	1
$010;\frac{1}{4}$	0000	000	20	0	0	0	0	0	0	1	0	1	1
		Space group #138 : $P4_2/ncm$											
$(hkl;d)$	$\mathbb{Z}_{2,2,2,4}$	weak	$m_{(2)}^{1\bar{1}0}$	$g_{\frac{1}{2}\frac{1}{2}0}^{001}$	$g_{\frac{1}{2}\frac{1}{2}0}^{1\bar{1}0}$	$g_{00\frac{1}{2}}^{100}$	2^{001}	2^{110}	i	2_1^{100}	2_1^{110}	4_2^{001}	$\bar{4}^{001}$
$001;\frac{1}{4}$	0000	000	0	0	0	1	0	0	0	1	0	1	1
$001;0$	0002	000	0	1	0	1	0	1	1	0	1	1	0

Continued on next page

Supplementary Table 5 – continued

eLC	SI	Invariants																
110; $\frac{1}{2}$	0002	000	2	1	1	0	0	0	1	1	0	1	0					
Space group #139 : $I4/mmm$																		
$(hkl;d)$	$\mathbb{Z}_{2,8}$	weak	$m^{001}_{(4)}$	$m^{1\bar{1}0}_{(2)}$	$m^{100}_{(2)}$	$g^{001}_{\frac{1}{2}\frac{1}{2}0}$	$g^{1\bar{1}0}_{\frac{1}{2}\frac{1}{2}0}$	$g^{100}_{0\frac{1}{2}\frac{1}{2}}$	2^{001}	2^{100}	2^{110}	4^{001}	i	2^{001}_1	2^{100}_1	2^{110}_1	4^{001}_2	$\bar{4}^{001}$
110; 0	14	111	0	2	0	1	1	1	0	0	1	1	0	1	1	0	0	1
110; $\frac{1}{2}$	10	111	0	0	0	1	0	1	0	0	0	0	0	1	1	1	1	0
001; 0	12	111	2	0	0	0	0	1	0	1	1	0	1	1	0	0	1	1
010; 0	04	000	0	0	2	0	0	1	0	1	0	1	0	0	1	0	1	1
Space group #140 : $I4/mcm$																		
$(hkl;d)$	$\mathbb{Z}_{2,8}$	weak	$m^{001}_{(4)}$	$m^{1\bar{1}0}_{(2)}$	$g^{001}_{\frac{1}{2}\frac{1}{2}0}$	$g^{1\bar{1}0}_{\frac{1}{2}\frac{1}{2}0}$	$g^{100}_{00\frac{1}{2}}$	$g^{100}_{0\frac{1}{2}0}$	2^{001}	2^{100}	2^{110}	4^{001}	i	2^{001}_1	2^{100}_1	2^{110}_1	4^{001}_2	$\bar{4}^{001}$
110; 0	14	111	0	0	1	0	0	1	0	0	1	1	0	1	1	0	0	1
110; $\frac{1}{2}$	10	111	0	2	1	1	0	1	0	0	0	0	0	1	1	1	1	0
001; 0	12	111	2	0	0	0	1	0	0	0	0	0	1	1	1	1	1	1
001; $\frac{1}{2}$	10	111	0	0	1	0	1	0	0	1	1	0	0	1	0	0	1	0
Space group #141 : $I4_1/amd$																		
$(hkl;d)$	$\mathbb{Z}_{2,2,2,4}$	weak	$m^{100}_{(2)}$	$g^{001}_{0\frac{1}{2}0}$	$g^{001}_{\frac{1}{2}00}$	$g^{1\bar{1}0}_{\frac{1}{4}\frac{1}{4}\frac{\bar{1}}{4}}$	$g^{1\bar{1}0}_{\frac{1}{4}\frac{1}{4}\frac{1}{4}}$	$g^{100}_{0\frac{1}{2}\frac{1}{2}}$	2^{001}	2^{100}	2^{110}	i	2^{001}_1	2^{100}_1	2^{110}_1	4^{001}_1	4^{001}_3	$\bar{4}^{001}$
110; $\frac{1}{4}$	0000	000	0	0	0	1	1	0	0	0	1	0	0	0	1	1	1	1
101; 0	0002	000	0	1	1	1	1	0	0	1	0	1	0	1	0	1	1	0
010; $\frac{1}{2}$	0002	000	2	1	1	1	1	1	0	0	0	1	0	0	0	0	0	1
Space group #142 : $I4_1/acd$																		
$(hkl;d)$	$\mathbb{Z}_{2,2,2,4}$	weak	$g^{001}_{0\frac{1}{2}0}$	$g^{001}_{\frac{1}{2}00}$	$g^{1\bar{1}0}_{\frac{1}{4}\frac{1}{4}\frac{\bar{1}}{4}}$	$g^{1\bar{1}0}_{\frac{1}{4}\frac{1}{4}\frac{1}{4}}$	$g^{100}_{00\frac{1}{2}}$	$g^{100}_{0\frac{1}{2}0}$	2^{001}	2^{100}	2^{110}	i	2^{001}_1	2^{100}_1	2^{110}_1	4^{001}_1	4^{001}_3	$\bar{4}^{001}$
110; $\frac{1}{4}$	0000	000	0	0	1	1	0	0	0	0	1	0	0	0	1	1	1	1
001; 0	0002	000	1	1	1	1	0	0	0	1	0	1	0	1	0	1	1	0
010; $\frac{1}{2}$	0002	000	1	1	1	1	1	1	0	0	0	1	0	0	0	0	0	1
Space group #147 : $P\bar{3}$																		
$(hkl;d)$	$\mathbb{Z}_{2,2,2,4}$	weak	i															
001; 0	0012	001	1															
001; $\frac{1}{2}$	0010	001	0															
Space group #148 : $R\bar{3}$																		
$(hkl;d)$	$\mathbb{Z}_{2,2,2,4}$	weak	i															
$0\bar{1}1$; 0	1112	111	1															
$0\bar{1}1$; $\frac{1}{2}$	1110	111	0															
Space group #162 : $P\bar{3}1m$																		
$(hkl;d)$	$\mathbb{Z}_{2,2,2,4}$	weak	$m^{010}_{(2)}$	$g^{010}_{\frac{1}{2}00}$	2^{120}	i	2^{120}_1											
001; 0	0012	001	0	0	1	1	1											
001; $\frac{1}{2}$	0010	001	0	0	0	0	0											
010; 0	0002	000	2	1	0	1	0											
Space group #163 : $P\bar{3}1c$																		
$(hkl;d)$	$\mathbb{Z}_{2,2,2,4}$	weak	$g^{010}_{00\frac{1}{2}}$	$g^{010}_{\frac{1}{2}0\frac{1}{2}}$	2^{120}	i	2^{120}_1											
001; 0	0002	000	1	1	0	1	0											
001; $\frac{1}{4}$	0000	000	1	1	1	0	1											
Space group #164 : $P\bar{3}m1$																		
$(hkl;d)$	$\mathbb{Z}_{2,2,2,4}$	weak	$m^{\bar{2}10}_{(2)}$	$g^{\bar{2}10}_{\frac{1}{2}10}$	2^{100}	i	2^{100}_1											
001; 0	0012	001	0	0	1	1	1											
001; $\frac{1}{2}$	0010	001	0	0	0	0	0											
110; 0	0002	000	2	1	0	1	0											
Space group #165 : $P\bar{3}c1$																		
$(hkl;d)$	$\mathbb{Z}_{2,2,2,4}$	weak	$g^{\bar{2}10}_{00\frac{1}{2}}$	$g^{\bar{2}10}_{\frac{1}{2}1\frac{1}{2}}$	2^{100}	i	2^{100}_1											
001; 0	0002	000	1	1	0	1	0											
001; $\frac{1}{4}$	0000	000	1	1	1	0	1											
Space group #166 : $R\bar{3}m$																		
$(hkl;d)$	$\mathbb{Z}_{2,2,2,4}$	weak	$m^{\bar{2}10}_{(2)}$	$g^{\bar{2}10}_{\frac{1}{6}\frac{1}{3}\frac{1}{3}}$	2^{100}	i	2^{100}_1											
$0\bar{1}1$; 0	1112	111	0	1	1	1	1											
$0\bar{1}1$; $\frac{1}{2}$	1110	111	0	1	0	0	0											

Continued on next page

Supplementary Table 5 – continued

**eLC**	**SI**	**Invariants**													
$\bar{1}20;0$	0002	000	2	1	0	1	0								
		Space group #167 : $R\bar{3}c$													
$(hkl;d)$	$\mathbb{Z}_{2,2,2,4}$	weak	$g^{\bar{2}10}_{\frac{\bar{1}}{6}\frac{\bar{1}}{3}\frac{1}{6}}$	$g^{\bar{2}10}_{00\frac{1}{2}}$	2^{100}	i	2^{100}_{1}								
$0\bar{1}1;0$	0002	000	1	1	0	1	0								
$0\bar{1}1;\frac{1}{4}$	0000	000	1	1	1	0	1								
		Space group #174 : $P\bar{6}$													
$(hkl;d)$	$\mathbb{Z}_{3,3}$	weak	$m^{001}_{(3)}$												
$001;0$	11	001	11												
$001;\frac{1}{2}$	12	001	$1\bar{1}$												
		Space group #175 : $P6/m$													
$(hkl;d)$	$\mathbb{Z}_{6,12}$	weak	$m^{001}_{(6)}$	2^{001}	6^{001}	i									
$001;0$	1, 2	001	11	0	0	1									
$001;\frac{1}{2}$	5, 0	001	$1\bar{1}$	0	0	0									
$010;0$	0, 6	000	00	1	1	1									
		Space group #176 : $P6_3/m$													
$(hkl;d)$	$\mathbb{Z}_{12}$	weak	$m^{001}_{(6)}$	i	2^{001}_{1}	6^{001}_{3}									
$001;\frac{1}{4}$	8	000	20	0	1	1									
$001;0$	6	000	00	1	1	1									
		Space group #187 : $P\bar{6}m2$													
$(hkl;d)$	$\mathbb{Z}_{3,3}$	weak	$m^{\bar{1}20}_{(2)}$	$m^{001}_{(3)}$	$g^{\bar{1}20}_{1\frac{1}{2}0}$	$2^{1\bar{1}0}$	$2^{1\bar{1}0}_{1}$								
$001;0$	11	001	0	11	0	1	0								
$001;\frac{1}{2}$	12	001	0	$1\bar{1}$	0	0	1								
$110;0$	00	000	2	00	1	1	1								
		Space group #188 : $P\bar{6}c2$													
$(hkl;d)$	$\mathbb{Z}_{3}$	weak	$m^{001}_{(3)}$	$g^{\bar{1}20}_{00\frac{1}{2}}$	$g^{\bar{1}20}_{1\frac{1}{2}\frac{1}{2}}$	$2^{1\bar{1}0}$	$2^{1\bar{1}0}_{1}$								
$001;\frac{1}{4}$	2	000	20	1	1	0	0								
$001;0$	0	000	00	1	1	1	1								
		Space group #189 : $P\bar{6}2m$													
$(hkl;d)$	$\mathbb{Z}_{3,3}$	weak	$m^{001}_{(3)}$	$m^{1\bar{1}0}_{(2)}$	$g^{1\bar{1}0}_{\frac{1}{2}\frac{1}{2}0}$	2^{010}	2^{010}_{1}								
$001;0$	11	001	11	0	0	1	0								
$001;\frac{1}{2}$	12	001	$1\bar{1}$	0	0	0	1								
$010;0$	00	000	00	2	1	1	1								
		Space group #190 : $P\bar{6}2c$													
$(hkl;d)$	$\mathbb{Z}_{3}$	weak	$m^{001}_{(3)}$	$g^{1\bar{1}0}_{00\frac{1}{2}}$	$g^{1\bar{1}0}_{\frac{1}{2}\frac{1}{2}\frac{1}{2}}$	2^{010}	2^{010}_{1}								
$001;\frac{1}{4}$	2	000	20	1	1	0	0								
$001;0$	0	000	00	1	1	1	1								
		Space group #191 : $P6/mmm$													
$(hkl;d)$	$\mathbb{Z}_{6,12}$	weak	$m^{\bar{1}20}_{(2)}$	$m^{001}_{(6)}$	$m^{010}_{(2)}$	$g^{\bar{1}20}_{1\frac{1}{2}0}$	$g^{010}_{\frac{1}{2}00}$	2^{001}	2^{010}	$2^{1\bar{1}0}$	6^{001}	i	2^{010}_{1}	$2^{1\bar{1}0}_{1}$	
$001;0$	1, 2	001	0	11	0	0	0	0	1	1	0	1	0	0	
$001;\frac{1}{2}$	5, 0	001	0	$1\bar{1}$	0	0	0	0	0	0	0	0	1	1	
$010;0$	0, 6	000	0	00	2	0	1	1	1	0	1	1	1	0	
$110;0$	0, 6	000	2	00	0	1	0	1	0	1	1	1	0	1	
		Space group #192 : $P6/mcc$													
$(hkl;d)$	$\mathbb{Z}_{12}$	weak	$m^{001}_{(6)}$	$g^{\bar{1}20}_{00\frac{1}{2}}$	$g^{\bar{1}20}_{1\frac{1}{2}\frac{1}{2}}$	$g^{010}_{00\frac{1}{2}}$	$g^{010}_{\frac{1}{2}0\frac{1}{2}}$	2^{001}	2^{010}	$2^{1\bar{1}0}$	6^{001}	i	2^{010}_{1}	$2^{1\bar{1}0}_{1}$	
$001;\frac{1}{4}$	0	000	00	1	1	1	1	0	1	1	0	0	1	1	
$001;0$	2	000	20	1	1	1	1	0	0	0	0	1	0	0	
$010;0$	6	000	00	0	0	1	1	1	1	0	1	1	1	0	
		Space group #193 : $P6_3/mcm$													
$(hkl;d)$	$\mathbb{Z}_{12}$	weak	$m^{001}_{(6)}$	$m^{010}_{(2)}$	$g^{\bar{1}20}_{00\frac{1}{2}}$	$g^{\bar{1}20}_{1\frac{1}{2}\frac{1}{2}}$	$g^{010}_{\frac{1}{2}00}$	2^{010}	$2^{1\bar{1}0}$	i	2^{001}_{1}	2^{010}_{1}	$2^{1\bar{1}0}_{1}$	6^{001}_{3}	
$001;\frac{1}{4}$	8	000	20	0	1	1	0	1	0	0	1	1	0	1	
$001;0$	6	000	00	0	1	1	0	0	1	1	1	0	1	1	
$010;0$	6	000	00	2	0	0	1	1	0	1	1	1	0	1	
		Space group #194 : $P6_3/mmc$													

Continued on next page

Supplementary Table 5 – continued

eLC	SI	Invariants												
$(hkl;d)$	$\mathbb{Z}_{12}$	weak	$m^{\bar{1}20}_{(2)}$	$m^{001}_{(6)}$	$g^{\bar{1}20}_{1\frac{1}{2}0}$	$g^{010}_{00\frac{1}{2}}$	$g^{010}_{\frac{1}{2}0\frac{1}{2}}$	2^{010}	$2^{1\bar{1}0}$	i	2^{001}_{1}	2^{010}_{1}	$2^{1\bar{1}0}_{1}$	6^{001}_{3}
$001;0$	6	000	0	00	0	1	1	1	0	1	1	1	0	1
$001;\frac{1}{4}$	8	000	0	20	0	1	1	0	1	0	1	0	1	1
$110;0$	6	000	2	00	1	0	0	0	1	1	1	0	1	1

Space group #200 : $Pm\bar{3}$

$(hkl;d)$	$\mathbb{Z}_{2,2,2,4}$	weak	$m^{001}_{(2)}$	2^{001}	i
$001;0$	1112	111	11	0	1
$001;\frac{1}{2}$	1110	111	$1\bar{1}$	0	0

Space group #201 : $Pn\bar{3}$

$(hkl;d)$	$\mathbb{Z}_{2,2,2,4}$	weak	$g^{001}_{\frac{1}{2}\frac{1}{2}0}$	2^{001}	i
$001;0$	0002	000	1	0	1

Space group #202 : $Fm\bar{3}$

$(hkl;d)$	$\mathbb{Z}_{2,2,2,4}$	weak	$m^{001}_{(2)}$	$g^{001}_{\frac{1}{2}00}$	2^{001}	i	2^{001}_{1}
$100;0$	0002	000	2	1	0	1	0

Space group #203 : $Fd\bar{3}$

$(hkl;d)$	$\mathbb{Z}_{2,2,2,4}$	weak	$g^{001}_{\frac{1}{4}\frac{1}{4}0}$	$g^{001}_{\frac{1}{4}\frac{\bar{1}}{4}0}$	2^{001}	i	2^{001}_{1}
$11\bar{1};0$	0002	000	1	1	0	1	0

Space group #204 : $Im\bar{3}$

$(hkl;d)$	$\mathbb{Z}_{2,2,2,4}$	weak	$m^{001}_{(2)}$	$g^{001}_{\frac{1}{2}\frac{1}{2}0}$	2^{001}	i	2^{001}_{1}
$110;0$	1110	111	0	1	0	0	1
$001;0$	1112	111	2	0	0	1	1

Space group #205 : $Pa\bar{3}$

$(hkl;d)$	$\mathbb{Z}_{2,2,2,4}$	weak	$g^{001}_{\frac{1}{2}00}$	i	2^{001}_{1}
$001;0$	0002	000	1	1	0

Space group #206 : $Ia\bar{3}$

$(hkl;d)$	$\mathbb{Z}_{2,2,2,4}$	weak	$g^{001}_{0\frac{1}{2}0}$	$g^{001}_{\frac{1}{2}00}$	2^{001}	i	2^{001}_{1}
$001;0$	1112	111	0	1	1	1	0
$001;\frac{1}{2}$	1110	111	1	0	1	0	0

Space group #221 : $Pm\bar{3}m$

$(hkl;d)$	$\mathbb{Z}_{4,8}$	weak	$m^{001}_{(4)}$	$m^{101}_{(2)}$	$g^{101}_{\frac{\bar{1}}{2}0\frac{1}{2}}$	2^{001}	2^{011}	4^{001}	i	2^{011}_{1}	$\bar{4}^{001}$
$001;0$	16	111	11	0	1	0	1	1	1	0	0
$001;\frac{1}{2}$	30	111	$1\bar{1}$	0	1	0	0	0	0	1	0
$011;0$	04	000	00	2	1	0	1	1	0	1	1

Space group #222 : $Pn\bar{3}n$

$(hkl;d)$	$\mathbb{Z}_{2,2,2,4}$	weak	$g^{001}_{\frac{1}{2}\frac{1}{2}0}$	$g^{101}_{0\frac{1}{2}0}$	$g^{101}_{\frac{\bar{1}}{2}\frac{1}{2}\frac{1}{2}}$	2^{001}	2^{011}	4^{001}	i	2^{011}_{1}	$\bar{4}^{001}$
$001;0$	0002	000	1	1	1	0	0	0	1	0	1
$001;\frac{1}{4}$	0000	000	0	1	1	0	1	1	0	1	1

Space group #223 : $Pm\bar{3}n$

$(hkl;d)$	$\mathbb{Z}_{2,2,2,4}$	weak	$m^{001}_{(4)}$	$g^{101}_{0\frac{1}{2}0}$	$g^{101}_{\frac{\bar{1}}{2}\frac{1}{2}\frac{1}{2}}$	2^{001}	2^{011}	i	2^{011}_{1}	4^{001}_{2}	$\bar{4}^{001}$
$001;0$	0002	000	20	1	1	0	0	1	0	0	1
$001;\frac{1}{4}$	0000	000	00	1	1	0	1	0	1	1	1

Space group #224 : $Pn\bar{3}m$

$(hkl;d)$	$\mathbb{Z}_{2,2,2,4}$	weak	$m^{101}_{(2)}$	$g^{001}_{\frac{1}{2}\frac{1}{2}0}$	$g^{101}_{\frac{\bar{1}}{2}0\frac{1}{2}}$	2^{001}	2^{011}	i	2^{011}_{1}	4^{001}_{2}	$\bar{4}^{001}$
$001;0$	0002	000	0	1	0	0	1	1	1	1	0
$011;\frac{1}{2}$	0002	000	2	1	1	0	0	1	0	0	1

Space group #225 : $Fm\bar{3}m$

$(hkl;d)$	$\mathbb{Z}_{8}$	weak	$m^{001}_{(4)}$	$m^{101}_{(2)}$	$g^{001}_{\frac{1}{2}00}$	$g^{101}_{\frac{1}{4}\frac{1}{2}\frac{\bar{1}}{4}}$	2^{001}	2^{011}	4^{001}	i	2^{001}_{1}	2^{011}_{1}	4^{001}_{2}	$\bar{4}^{001}$
$100;0$	6	000	2	0	1	0	0	1	1	1	0	1	1	0
$0\bar{1}1;0$	4	000	0	2	0	1	0	1	1	0	0	1	1	1

Space group #226 : $Fm\bar{3}c$

$(hkl;d)$	$\mathbb{Z}_{8}$	weak	$m^{001}_{(4)}$	$g^{001}_{\frac{1}{2}00}$	$g^{101}_{0\frac{1}{2}0}$	$g^{101}_{\frac{1}{4}0\frac{\bar{1}}{4}}$	2^{001}	2^{011}	4^{001}	i	2^{001}_{1}	2^{011}_{1}	4^{001}_{2}	$\bar{4}^{001}$

Continued on next page

Supplementary Table 5 – continued

**eLC**	**SI**	**Invariants**												
$100;0$	2	000	2	1	1	1	0	0	0	1	0	0	0	1
$100;\frac{1}{2}$	4	000	0	0	1	1	0	1	1	0	0	1	1	1
Space group #227 : $Fd\bar{3}m$														
$(hkl;d)$	$\mathbb{Z}_{2,2,2,4}$	weak	$m^{101}_{(2)}$	$g^{001}_{\frac{1}{4}\frac{1}{4}0}$	$g^{001}_{\frac{1}{4}\frac{\bar{1}}{4}0}$	$g^{101}_{\frac{1}{4}\frac{1}{2}\frac{\bar{1}}{4}}$	2^{001}	2^{011}	i	2^{001}_1	2^{011}_1	4^{001}_1	4^{001}_3	$\bar{4}^{001}$
$11\bar{1};0$	0002	000	0	1	1	0	0	1	1	0	1	1	1	0
$0\bar{1}1;0$	0002	000	2	1	1	1	0	0	1	0	0	0	0	1
Space group #228 : $Fd\bar{3}c$														
$(hkl;d)$	$\mathbb{Z}_{2,2,2,4}$	weak	$g^{001}_{\frac{1}{4}\frac{1}{4}0}$	$g^{001}_{\frac{1}{4}\frac{\bar{1}}{4}0}$	$g^{101}_{0\frac{1}{2}0}$	$g^{101}_{\frac{1}{4}0\frac{\bar{1}}{4}}$	2^{001}	2^{011}	i	2^{001}_1	2^{011}_1	4^{001}_1	4^{001}_3	$\bar{4}^{001}$
$100;0$	0002	000	1	1	0	0	0	1	1	0	1	1	1	0
$0\bar{1}1;0$	0002	000	1	1	1	1	0	0	1	0	0	0	0	1
Space group #229 : $Im\bar{3}m$														
$(hkl;d)$	$\mathbb{Z}_{2,8}$	weak	$m^{001}_{(4)}$	$m^{101}_{(2)}$	$g^{001}_{\frac{1}{2}\frac{1}{2}0}$	$g^{101}_{0\frac{1}{2}0}$	2^{001}	2^{011}	4^{001}	i	2^{001}_1	2^{011}_1	4^{001}_2	$\bar{4}^{001}$
$110;0$	14	111	0	2	1	0	0	1	1	0	1	0	0	1
$110;\frac{1}{2}$	10	111	0	0	1	1	0	0	0	0	1	1	1	0
$001;0$	16	111	2	0	0	1	0	1	1	1	1	0	0	0
Space group #230 : $Ia\bar{3}d$														
$(hkl;d)$	$\mathbb{Z}_{2,2,2,4}$	weak	$g^{001}_{0\frac{1}{2}0}$	$g^{001}_{\frac{1}{2}00}$	$g^{101}_{\frac{\bar{1}}{4}\frac{1}{4}\frac{1}{4}}$	$g^{101}_{\frac{1}{4}\frac{1}{4}\frac{\bar{1}}{4}}$	2^{001}	2^{011}	i	2^{001}_1	2^{011}_1	4^{001}_1	4^{001}_3	$\bar{4}^{001}$
$110;\frac{1}{4}$	0000	000	0	0	1	1	0	1	0	0	1	1	1	1
$001;0$	0002	000	1	1	1	1	0	0	1	0	0	0	0	1

Supplementary Table 6: eLCs in all SGs with trivial SI groups.

**eLC**	**Invariants**			
Space group #1 : $P1$				
$(hkl;d)$	weak			
$001;d_0$	001			
$010;d_0$	010			
$100;d_0$	100			
Space group #3 : $P2$				
$(hkl;d)$	weak	2^{010}		
$001;0$	001	1		
$001;\frac{1}{2}$	001	0		
$010;d_0$	010	0		
$100;0$	100	1		
Space group #4 : $P2_1$				
$(hkl;d)$	weak	2^{010}_1		
$001;0$	001	1		
$001;\frac{1}{2}$	001	0		
$100;0$	100	1		
Space group #5 : $C2$				
$(hkl;d)$	weak	2^{010}	2^{010}_1	
$001;0$	001	1	1	
$001;\frac{1}{2}$	001	0	0	
$\bar{1}10;d_0$	110	0	1	
Space group #6 : Pm				
$(hkl;d)$	weak	$m^{010}_{(2)}$		
$001;d_0$	001	00		
$010;0$	010	11		
$010;\frac{1}{2}$	010	$1\bar{1}$		
$100;d_0$	100	00		
Space group #7 : Pc				
$(hkl;d)$	weak	$g^{010}_{00\frac{1}{2}}$		
$001;d_0$	000	1		
$010;0$	010	1		

Continued on next column

Supplementary Table 6 – continued

**eLC**	**Invariants**			
$100;d_0$	100	0		
Space group #8 : Cm				
$(hkl;d)$	weak	$m^{010}_{(2)}$	$g^{010}_{\frac{\bar{1}}{2}00}$	
$001;d_0$	001	0	0	
$\bar{1}10;d_0$	110	0	1	
$010;0$	110	2	0	
Space group #9 : Cc				
$(hkl;d)$	weak	$g^{010}_{00\frac{1}{2}}$	$g^{010}_{\frac{\bar{1}}{2}0\frac{1}{2}}$	
$001;d_0$	000	1	1	
$\bar{1}10;d_0$	110	0	1	
Space group #16 : $P222$				
$(hkl;d)$	weak	2^{001}	2^{010}	2^{100}
$001;0$	001	0	1	1
$001;\frac{1}{2}$	001	0	0	0
$010;0$	010	1	0	1
$010;\frac{1}{2}$	010	0	0	0
$100;0$	100	1	1	0
Space group #17 : $P222_1$				
$(hkl;d)$	weak	2^{010}	2^{100}	2^{001}_1
$001;\frac{1}{4}$	000	1	0	1
$001;0$	000	0	1	1
$010;0$	010	0	1	1
$100;0$	100	1	0	1
Space group #18 : $P2_12_12$				
$(hkl;d)$	weak	2^{001}	2^{010}_1	2^{100}_1
$001;0$	001	0	1	1
$001;\frac{1}{2}$	001	0	0	0
$010;0$	000	1	1	0
Space group #19 : $P2_12_12_1$				
$(hkl;d)$	weak	2^{001}_1	2^{010}_1	2^{100}_1
$001;\frac{1}{4}$	000	1	1	0
$001;0$	000	1	0	1

Continued on next column

Supplementary Table 6 – continued

eLC	Invariants

Space group #20 : $C222_1$

$(hkl;d)$	weak	2^{010}	2^{100}	2_1^{001}	2_1^{010}	2_1^{100}
$001;\frac{1}{4}$	000	1	0	1	1	0
$001;0$	000	0	1	1	0	1
$\bar{1}10;0$	110	0	0	0	1	1

Space group #21 : $C222$

$(hkl;d)$	weak	2^{001}	2^{010}	2^{100}	2_1^{010}	2_1^{100}
$001;0$	001	0	1	1	1	1
$001;\frac{1}{2}$	001	0	0	0	0	0
$\bar{1}10;0$	110	0	0	0	1	1
$010;0$	110	1	0	1	1	0

Space group #22 : $F222$

$(hkl;d)$	weak	2^{001}	2^{010}	2^{100}	2_1^{001}	2_1^{010}	2_1^{100}
$100;0$	011	1	1	0	1	1	1
$100;\frac{1}{2}$	011	0	0	0	0	0	1
$010;0$	101	1	0	1	0	1	1
$010;\frac{1}{2}$	101	0	0	0	1	1	0

Space group #23 : $I222$

$(hkl;d)$	weak	2^{001}	2^{010}	2^{100}	2_1^{001}	2_1^{010}	2_1^{100}
$110;0$	111	0	0	0	1	1	1
$001;0$	111	0	1	1	1	0	0
$010;0$	111	1	0	1	0	1	0

Space group #24 : $I2_12_12_1$

$(hkl;d)$	weak	2^{001}	2^{010}	2^{100}	2_1^{001}	2_1^{010}	2_1^{100}
$110;\frac{1}{4}$	111	1	0	0	0	1	1
$101;\frac{1}{4}$	111	0	1	0	1	0	1
$0\bar{1}1;\frac{1}{4}$	111	0	0	1	1	1	0

Space group #25 : $Pmm2$

$(hkl;d)$	weak	$m_{(2)}^{010}$	$m_{(2)}^{100}$	2^{001}
$001;d_0$	001	00	00	0
$010;0$	010	11	00	1
$010;\frac{1}{2}$	010	$1\bar{1}$	00	0
$100;0$	100	00	11	1
$100;\frac{1}{2}$	100	00	$1\bar{1}$	0

Space group #26 : $Pmc2_1$

$(hkl;d)$	weak	$m_{(2)}^{100}$	$g_{00\frac{1}{2}}^{010}$	2_1^{001}
$001;d_0$	000	00	1	1
$010;0$	010	00	1	1
$100;0$	100	11	0	1
$100;\frac{1}{2}$	100	$1\bar{1}$	0	0

Space group #27 : $Pcc2$

$(hkl;d)$	weak	$g_{00\frac{1}{2}}^{010}$	$g_{00\frac{1}{2}}^{100}$	2^{001}
$001;d_0$	000	1	1	0
$010;0$	010	1	0	1
$010;\frac{1}{2}$	010	0	0	0
$100;0$	100	0	1	1

Space group #28 : $Pma2$

$(hkl;d)$	weak	$m_{(2)}^{100}$	$g_{\frac{1}{2}00}^{010}$	2^{001}
$001;d_0$	001	00	0	0
$010;0$	010	00	1	1
$010;\frac{1}{2}$	010	00	0	0
$100;\frac{1}{4}$	000	20	1	0

Space group #29 : $Pca2_1$

$(hkl;d)$	weak	$g_{\frac{1}{2}00}^{010}$	$g_{00\frac{1}{2}}^{100}$	2_1^{001}
$001;d_0$	000	0	1	1
$010;0$	010	1	0	1
$010;\frac{1}{2}$	010	0	0	0

Continued on next column

Supplementary Table 6 – continued

eLC	Invariants

Space group #30 : $Pnc2$

$(hkl;d)$	weak	$g_{00\frac{1}{2}}^{010}$	$g_{0\frac{1}{2}\frac{1}{2}}^{100}$	2^{001}
$001;d_0$	000	1	1	0
$010;0$	000	0	1	1
$100;0$	100	0	1	1

Space group #31 : $Pmn2_1$

$(hkl;d)$	weak	$m_{(2)}^{100}$	$g_{\frac{1}{2}0\frac{1}{2}}^{010}$	2_1^{001}
$001;d_0$	000	00	1	1
$010;0$	010	00	1	1
$100;0$	000	20	1	0

Space group #32 : $Pba2$

$(hkl;d)$	weak	$g_{\frac{1}{2}00}^{010}$	$g_{0\frac{1}{2}0}^{100}$	2^{001}
$001;d_0$	001	0	0	0
$010;0$	000	0	1	1
$010;\frac{1}{4}$	000	1	1	0

Space group #33 : $Pna2_1$

$(hkl;d)$	weak	$g_{\frac{1}{2}00}^{010}$	$g_{0\frac{1}{2}\frac{1}{2}}^{100}$	2_1^{001}
$001;d_0$	000	0	1	1
$010;\frac{1}{4}$	000	1	1	0

Space group #34 : $Pnn2$

$(hkl;d)$	weak	$g_{\frac{1}{2}0\frac{1}{2}}^{010}$	$g_{0\frac{1}{2}\frac{1}{2}}^{100}$	2^{001}
$001;d_0$	000	1	1	0
$010;0$	000	0	1	1

Space group #35 : $Cmm2$

$(hkl;d)$	weak	$m_{(2)}^{010}$	$m_{(2)}^{100}$	$g_{\frac{1}{2}00}^{010}$	$g_{0\frac{1}{2}0}^{100}$	2^{001}
$001;d_0$	001	0	0	0	0	0
$\bar{1}10;0$	110	0	0	1	1	0
$010;0$	110	2	0	0	1	1
$100;0$	110	0	2	1	0	1

Space group #36 : $Cmc2_1$

$(hkl;d)$	weak	$m_{(2)}^{100}$	$g_{00\frac{1}{2}}^{010}$	$g_{\bar{\frac{1}{2}}0\frac{1}{2}}^{010}$	$g_{0\frac{1}{2}0}^{100}$	2_1^{001}
$001;d_0$	000	0	1	1	0	1
$\bar{1}10;0$	110	0	0	1	1	0
$100;0$	110	2	0	1	0	1

Space group #37 : $Ccc2$

$(hkl;d)$	weak	$g_{00\frac{1}{2}}^{010}$	$g_{\bar{\frac{1}{2}}0\frac{1}{2}}^{010}$	$g_{00\frac{1}{2}}^{100}$	$g_{0\frac{1}{2}\frac{1}{2}}^{100}$	2^{001}
$001;d_0$	000	1	1	1	1	0
$\bar{1}10;0$	110	0	1	0	1	0
$010;0$	110	1	0	0	1	1

Space group #38 : $Amm2$

$(hkl;d)$	weak	$m_{(2)}^{010}$	$m_{(2)}^{100}$	$g_{00\frac{1}{2}}^{010}$	2^{001}	2_1^{001}
$0\bar{1}1;d_0$	011	0	00	1	0	1
$010;0$	011	2	00	0	1	0
$100;0$	100	0	11	0	1	1
$100;\frac{1}{2}$	100	0	$1\bar{1}$	0	0	0

Space group #39 : $Aem2$

$(hkl;d)$	weak	$m_{(2)}^{010}$	$g_{00\frac{1}{2}}^{010}$	$g_{00\frac{1}{2}}^{100}$	2^{001}	2_1^{001}
$001;d_0$	011	0	1	1	0	1
$010;0$	011	0	1	0	1	0
$010;\frac{1}{2}$	011	2	0	0	0	1
$100;0$	100	0	0	1	1	1

Space group #40 : $Ama2$

Continued on next column

Supplementary Table 6 – continued

eLC $(hkl;d)$	weak	$m^{100}_{(2)}$	$g^{010}_{\frac{1}{2}00}$	$g^{010}_{\frac{1}{2}0\frac{1}{2}}$	2^{001}	2^{001}_1
$0\bar{1}1;d_0$	011	00	0	1	0	1
$010;0$	011	00	1	0	1	0
$100;\frac{1}{4}$	000	20	1	1	0	0

Space group #41 : $Aea2$

$(hkl;d)$	weak	$g^{010}_{\frac{1}{2}00}$	$g^{010}_{\frac{1}{2}0\frac{1}{2}}$	$g^{100}_{00\frac{1}{2}}$	2^{001}	2^{001}_1
$001;d_0$	011	0	1	1	0	1
$010;0$	011	0	1	0	1	0
$010;\frac{1}{2}$	011	1	0	0	0	1

Space group #42 : $Fmm2$

$(hkl;d)$	weak	$m^{010}_{(2)}$	$m^{100}_{(2)}$	$g^{010}_{\frac{1}{2}00}$	$g^{100}_{0\frac{1}{2}0}$	2^{001}	2^{001}_1
$100;0$	011	0	2	1	0	1	1
$100;\frac{1}{2}$	011	0	0	1	1	0	0
$010;0$	101	2	0	0	1	1	0
$010;\frac{1}{2}$	101	0	0	1	1	0	1

Space group #43 : $Fdd2$

$(hkl;d)$	weak	$g^{010}_{\frac{1}{4}0\bar{\frac{1}{4}}}$	$g^{010}_{\frac{1}{4}0\frac{1}{4}}$	$g^{100}_{0\frac{1}{4}\frac{1}{4}}$	$g^{100}_{0\frac{1}{4}\bar{\frac{1}{4}}}$	2^{001}	2^{001}_1
$11\bar{1};d_0$	000	1	1	1	1	0	0
$100;0$	000	1	1	0	0	1	1

Space group #44 : $Imm2$

$(hkl;d)$	weak	$m^{010}_{(2)}$	$m^{100}_{(2)}$	$g^{010}_{\frac{1}{2}0\frac{1}{2}}$	$g^{100}_{0\frac{1}{2}\frac{1}{2}}$	2^{001}	2^{001}_1
$110;0$	111	0	0	1	1	0	1
$010;0$	111	2	0	0	1	1	0
$\bar{1}00;0$	111	0	2	1	0	1	0

Space group #45 : $Iba2$

$(hkl;d)$	weak	$g^{010}_{00\frac{1}{2}}$	$g^{010}_{\frac{1}{2}00}$	$g^{100}_{00\frac{1}{2}}$	$g^{100}_{0\frac{1}{2}0}$	2^{001}	2^{001}_1
$110;0$	111	0	1	0	1	0	1
$001;d_0$	111	1	0	1	0	0	1
$010;0$	111	1	0	0	1	1	0

Space group #46 : $Ima2$

$(hkl;d)$	weak	$m^{100}_{(2)}$	$g^{010}_{00\frac{1}{2}}$	$g^{010}_{\frac{1}{2}00}$	$g^{100}_{0\frac{1}{2}\frac{1}{2}}$	2^{001}	2^{001}_1
$110;0$	111	0	0	1	1	1	0
$0\bar{1}1;d_0$	111	0	1	0	1	0	1
$\bar{1}00;\frac{1}{2}$	111	2	0	1	0	0	1

Space group #75 : $P4$

$(hkl;d)$	weak	2^{001}	4^{001}
$001;d_0$	001	0	0
$010;0$	110	0	1
$010;\frac{1}{2}$	110	0	0

Space group #76 : $P4_1$

$(hkl;d)$	weak	2^{001}_1	4^{001}_1
$001;d_0$	000	0	1
$010;0$	110	0	1

Space group #77 : $P4_2$

$(hkl;d)$	weak	2^{001}	4^{001}_2
$001;d_0$	000	0	1
$010;0$	110	0	1

Space group #78 : $P4_3$

$(hkl;d)$	weak	2^{001}_1	4^{001}_3
$001;d_0$	000	0	1
$010;0$	110	0	1

Space group #79 : $I4$

$(hkl;d)$	weak	2^{001}	4^{001}	2^{001}_1	4^{001}_2
$110;0$	111	0	1	1	0
$110;\frac{1}{2}$	111	0	0	1	1

Space group #80 : $I4_1$

$(hkl;d)$	weak	2^{001}	2^{001}_1	4^{001}_1	4^{001}_3
$110;0$	111	1	0	1	0
$110;\frac{1}{2}$	111	1	0	0	1

Space group #81 : $P\bar{4}$

$(hkl;d)$	weak	2^{001}	$\bar{4}^{001}$
$001;0$	001	0	1
$001;\frac{1}{2}$	001	0	0
$010;0$	110	0	1

Space group #82 : $I\bar{4}$

$(hkl;d)$	weak	2^{001}	2^{001}_1	$\bar{4}^{001}$
$110;0$	111	0	1	1
$110;\frac{1}{2}$	111	0	1	0

Space group #89 : $P422$

$(hkl;d)$	weak	2^{001}	2^{100}	2^{110}	4^{001}	2^{110}_1
$001;0$	001	0	1	1	0	0
$001;\frac{1}{2}$	001	0	0	0	0	1
$010;0$	110	0	1	0	1	1
$010;\frac{1}{2}$	110	0	0	0	0	1

Space group #90 : $P42_12$

$(hkl;d)$	weak	2^{001}	2^{110}	4^{001}	2^{100}_1	2^{110}_1
$001;0$	001	0	1	0	1	0
$001;\frac{1}{2}$	001	0	0	0	0	1
$010;0$	000	0	0	1	1	0

Space group #91 : $P4_122$

$(hkl;d)$	weak	2^{100}	2^{110}	2^{001}_1	2^{110}_1	4^{001}_1
$001;0$	000	1	0	0	0	1
$001;\frac{1}{8}$	000	0	1	0	1	1
$010;0$	110	1	0	0	1	1

Space group #92 : $P4_12_12$

$(hkl;d)$	weak	2^{110}	2^{001}_1	2^{100}_1	2^{110}_1	4^{001}_1
$001;\frac{1}{8}$	000	0	0	1	0	1
$001;\frac{1}{4}$	000	1	0	0	1	1

Space group #93 : $P4_222$

$(hkl;d)$	weak	2^{001}	2^{100}	2^{110}	2^{110}_1	4^{001}_2
$001;0$	000	0	1	0	0	1
$001;\frac{1}{4}$	000	0	0	1	1	1
$010;0$	110	0	1	0	1	1

Space group #94 : $P4_22_12$

$(hkl;d)$	weak	2^{001}	2^{110}	2^{100}_1	2^{110}_1	4^{001}_2
$001;\frac{1}{4}$	000	0	0	1	0	1
$001;0$	000	0	1	0	1	1

Space group #95 : $P4_322$

$(hkl;d)$	weak	2^{100}	2^{110}	2^{001}_1	2^{110}_1	4^{001}_3
$001;0$	000	1	0	0	0	1
$001;\frac{3}{8}$	000	0	1	0	1	1
$010;0$	110	1	0	0	1	1

Space group #96 : $P4_32_12$

$(hkl;d)$	weak	2^{110}	2^{001}_1	2^{100}_1	2^{110}_1	4^{001}_3
$001;\frac{3}{8}$	000	0	0	1	0	1
$001;\frac{1}{4}$	000	1	0	0	1	1

Space group #97 : $I422$

$(hkl;d)$	weak	2^{001}	2^{100}	2^{110}	4^{001}	2^{001}_1	2^{100}_1	2^{110}_1	4^{001}_2
$110;0$	111	0	0	1	1	1	1	0	0
$110;\frac{1}{2}$	111	0	0	0	0	1	1	1	1

Continued on next column

Supplementary Table 6 – continued

eLC	Invariants								
$001;0$	111	0	1	1	0	1	0	0	1

Space group #98 : $I4_122$

$(hkl;d)$	weak	2^{001}	2^{100}	2^{110}	2_1^{001}	2_1^{100}	2_1^{110}	4_1^{001}	4_3^{001}
$110;0$	111	1	0	0	0	1	1	1	0
$110;\frac{1}{2}$	111	1	0	1	0	1	0	0	1
$101;\frac{1}{8}$	000	0	1	0	0	1	0	1	1

Space group #99 : $P4mm$

$(hkl;d)$	weak	$m_{(2)}^{100}$	$m_{(2)}^{110}$	$g^{110}_{\frac{\bar{1}}{2}\frac{1}{2}0}$	2^{001}	4^{001}
$001;d_0$	001	00	0	0	0	0
$010;0$	110	11	0	1	0	1
$010;\frac{1}{2}$	110	$1\bar{1}$	0	1	0	0
$110;0$	000	00	2	1	0	1

Space group #100 : $P4bm$

$(hkl;d)$	weak	$m_{(2)}^{110}$	$g^{100}_{0\frac{1}{2}0}$	$g^{110}_{\frac{\bar{1}}{2}\frac{1}{2}0}$	2^{001}	4^{001}
$001;d_0$	001	0	0	0	0	0
$010;0$	000	0	1	0	0	1
$110;\frac{1}{2}$	000	2	1	1	0	0

Space group #101 : $P4_2cm$

$(hkl;d)$	weak	$m_{(2)}^{110}$	$g^{100}_{00\frac{1}{2}}$	$g^{110}_{\frac{\bar{1}}{2}\frac{1}{2}0}$	2^{001}	4_2^{001}
$001;d_0$	000	0	1	0	0	1
$010;0$	110	0	1	1	0	1
$110;0$	000	2	0	1	0	1

Space group #102 : $P4_2cm$

$(hkl;d)$	weak	$m_{(2)}^{110}$	$g^{100}_{0\frac{1}{2}\frac{1}{2}}$	$g^{110}_{\frac{\bar{1}}{2}\frac{1}{2}0}$	2^{001}	4_2^{001}
$001;d_0$	000	0	1	0	0	1
$110;0$	000	2	1	1	0	0

Space group #103 : $P4cc$

$(hkl;d)$	weak	$g^{100}_{00\frac{1}{2}}$	$g^{110}_{00\frac{1}{2}}$	$g^{110}_{\frac{\bar{1}}{2}\frac{1}{2}\frac{1}{2}}$	2^{001}	4^{001}
$001;d_0$	000	1	1	1	0	0
$010;0$	110	1	0	1	0	1
$010;\frac{1}{2}$	110	0	0	1	0	0

Space group #104 : $P4nc$

$(hkl;d)$	weak	$g^{100}_{0\frac{1}{2}\frac{1}{2}}$	$g^{110}_{00\frac{1}{2}}$	$g^{110}_{\frac{\bar{1}}{2}\frac{1}{2}\frac{1}{2}}$	2^{001}	4^{001}
$001;d_0$	000	1	1	1	0	0
$010;0$	000	1	0	0	0	1

Space group #105 : $P4_2mc$

$(hkl;d)$	weak	$m_{(2)}^{100}$	$g^{110}_{00\frac{1}{2}}$	$g^{110}_{\frac{\bar{1}}{2}\frac{1}{2}\frac{1}{2}}$	2^{001}	4_2^{001}
$001;d_0$	000	00	1	1	0	1
$010;0$	110	11	0	1	0	1
$010;\frac{1}{2}$	110	$1\bar{1}$	0	1	0	0

Space group #106 : $P4_2bc$

$(hkl;d)$	weak	$g^{100}_{0\frac{1}{2}0}$	$g^{110}_{00\frac{1}{2}}$	$g^{110}_{\frac{\bar{1}}{2}\frac{1}{2}\frac{1}{2}}$	2^{001}	4_2^{001}
$001;d_0$	000	0	1	1	0	1
$010;0$	000	1	0	0	0	1

Space group #107 : $I4mm$

$(hkl;d)$	weak	$m_{(2)}^{100}$	$m_{(2)}^{110}$	$g^{100}_{0\frac{1}{2}\frac{1}{2}}$	$g^{110}_{00\frac{1}{2}}$	2^{001}	4^{001}	2_1^{001}	4_2^{001}
$110;0$	111	0	2	1	0	0	1	1	0
$110;\frac{1}{2}$	111	0	0	1	1	0	0	1	1
$010;0$	000	2	0	1	0	0	1	0	1

Space group #108 : $I4cm$

$(hkl;d)$	weak	$m_{(2)}^{110}$	$g^{100}_{00\frac{1}{2}}$	$g^{100}_{0\frac{1}{2}0}$	$g^{110}_{00\frac{1}{2}}$	2^{001}	4^{001}	2_1^{001}	4_2^{001}
$110;0$	111	0	0	1	1	0	1	1	0
$110;\frac{1}{2}$	111	2	0	1	0	0	0	1	1

Continued on next column

Supplementary Table 6 – continued

eLC	Invariants								
$001;d_0$	111	0	1	0	1	0	0	1	1

Space group #109 : $I4_1md$

$(hkl;d)$	weak	$m_{(2)}^{100}$	$g^{100}_{0\frac{1}{2}\frac{1}{2}}$	$g^{110}_{\frac{1}{4}\frac{\bar{1}}{4}\frac{1}{4}}$	$g^{110}_{\frac{\bar{1}}{4}\frac{1}{4}\frac{1}{4}}$	2^{001}	2_1^{001}	4_1^{001}	4_3^{001}
$110;0$	000	0	0	1	1	0	0	1	1
$010;0$	000	2	1	1	1	0	0	0	0

Space group #110 : $I4_1cd$

$(hkl;d)$	weak	$g^{100}_{00\frac{1}{2}}$	$g^{100}_{0\frac{1}{2}0}$	$g^{110}_{\frac{1}{4}\frac{\bar{1}}{4}\frac{1}{4}}$	$g^{110}_{\frac{\bar{1}}{4}\frac{1}{4}\frac{1}{4}}$	2^{001}	2_1^{001}	4_1^{001}	4_3^{001}
$110;0$	000	0	0	1	1	0	0	1	1
$010;0$	000	1	1	1	1	0	0	0	0

Space group #111 : $P\bar{4}2m$

$(hkl;d)$	weak	$m_{(2)}^{110}$	$g^{110}_{\frac{\bar{1}}{2}\frac{1}{2}0}$	2^{001}	2^{100}	$\bar{4}^{001}$
$001;0$	001	0	0	0	1	1
$001;\frac{1}{2}$	001	0	0	0	0	0
$010;0$	110	0	1	0	1	1
$110;0$	000	2	1	0	0	1

Space group #112 : $P\bar{4}2c$

$(hkl;d)$	weak	$g^{110}_{00\frac{1}{2}}$	$g^{110}_{\frac{\bar{1}}{2}\frac{1}{2}\frac{1}{2}}$	2^{001}	2^{100}	$\bar{4}^{001}$
$001;0$	000	1	1	0	0	1
$001;\frac{1}{4}$	000	1	1	0	1	0
$010;0$	110	0	1	0	1	1

Space group #113 : $P\bar{4}2_1m$

$(hkl;d)$	weak	$m_{(2)}^{110}$	$g^{110}_{\frac{\bar{1}}{2}\frac{1}{2}0}$	2^{001}	2_1^{100}	$\bar{4}^{001}$
$001;0$	001	0	0	0	1	1
$001;\frac{1}{2}$	001	0	0	0	0	0
$110;\frac{1}{2}$	000	2	1	0	1	0

Space group #114 : $P\bar{4}2_1c$

$(hkl;d)$	weak	$g^{110}_{00\frac{1}{2}}$	$g^{110}_{\frac{\bar{1}}{2}\frac{1}{2}\frac{1}{2}}$	2^{001}	2_1^{100}	$\bar{4}^{001}$
$001;0$	000	1	1	0	0	1
$001;\frac{1}{4}$	000	1	1	0	1	0

Space group #115 : $P\bar{4}m2$

$(hkl;d)$	weak	$m_{(2)}^{100}$	2^{001}	2^{110}	2_1^{110}	$\bar{4}^{001}$
$001;0$	001	00	0	1	0	1
$001;\frac{1}{2}$	001	00	0	0	1	0
$010;0$	110	11	0	0	1	1
$010;\frac{1}{2}$	110	$1\bar{1}$	0	0	1	0

Space group #116 : $P\bar{4}c2$

$(hkl;d)$	weak	$g^{100}_{00\frac{1}{2}}$	2^{001}	2^{110}	2_1^{110}	$\bar{4}^{001}$
$001;0$	000	1	0	0	0	1
$001;\frac{1}{4}$	000	1	0	1	1	0
$010;0$	110	1	0	0	1	1

Space group #117 : $P\bar{4}b2$

$(hkl;d)$	weak	$g^{100}_{0\frac{1}{2}0}$	2^{001}	2^{110}	2_1^{110}	$\bar{4}^{001}$
$001;0$	001	0	0	0	1	1
$001;\frac{1}{2}$	001	0	0	1	0	0
$010;0$	000	1	0	0	0	1

Space group #118 : $P\bar{4}n2$

$(hkl;d)$	weak	$g^{100}_{0\frac{1}{2}\frac{1}{2}}$	2^{001}	2^{110}	2_1^{110}	$\bar{4}^{001}$
$001;0$	000	1	0	0	0	1
$001;\frac{1}{4}$	000	1	0	1	1	0

Space group #119 : $I\bar{4}m2$

$(hkl;d)$	weak	$m_{(2)}^{100}$	$g^{100}_{0\frac{1}{2}\frac{1}{2}}$	2^{001}	2^{110}	2_1^{001}	2_1^{110}	$\bar{4}^{001}$
$110;0$	111	0	1	0	1	1	0	1
$110;\frac{1}{2}$	111	0	1	0	0	1	1	0

Continued on next column

Supplementary Table 6 – continued

**eLC**	**Invariants**							
010; 0	000	2	1	0	0	0	0	1

Space group #120 : $I\bar{4}c2$

$(hkl;d)$	weak	$g^{100}_{00\frac{1}{2}}$	$g^{100}_{0\frac{1}{2}0}$	2^{001}	2^{110}	2_1^{001}	2_1^{110}	$\bar{4}^{001}$
110; 0	111	0	1	0	1	1	0	1
110; $\frac{1}{2}$	111	0	1	0	0	1	1	0
001; 0	111	1	0	0	0	1	1	1

Space group #121 : $I\bar{4}2m$

$(hkl;d)$	weak	$m^{110}_{(2)}$	$g^{110}_{00\frac{1}{2}}$	2^{001}	2^{100}	2_1^{001}	2_1^{100}	$\bar{4}^{001}$
110; 0	111	2	0	0	0	1	1	1
110; $\frac{1}{2}$	111	0	1	0	0	1	1	0
001; 0	111	0	1	0	1	1	0	1

Space group #122 : $I\bar{4}2d$

$(hkl;d)$	weak	$g^{110}_{\frac{1}{4}\frac{\bar{1}}{4}\frac{1}{4}}$	$g^{110}_{\frac{\bar{1}}{4}\frac{1}{4}\frac{1}{4}}$	2^{001}	2^{100}	2_1^{001}	2_1^{100}	$\bar{4}^{001}$
110; 0	000	1	1	0	0	0	0	1
101; $\frac{1}{8}$	000	1	1	0	1	0	1	0

Space group #143 : $P3$

$(hkl;d)$	weak
001; d_0	001

Space group #144 : $P3_1$

$(hkl;d)$	weak
001; d_0	001

Space group #145 : $P3_2$

$(hkl;d)$	weak
001; d_0	001

Space group #146 : $R3$

$(hkl;d)$	weak
$0\bar{1}1$; d_0	111

Space group #149 : $P312$

$(hkl;d)$	weak	2^{120}	2_1^{120}
001; 0	001	1	1
001; $\frac{1}{2}$	001	0	0

Space group #150 : $P321$

$(hkl;d)$	weak	2^{100}	2_1^{100}
001; 0	001	1	1
001; $\frac{1}{2}$	001	0	0

Space group #151 : $P3_112$

$(hkl;d)$	weak	2^{120}	2_1^{120}
001; $\frac{1}{3}$	001	0	0
001; $\frac{5}{6}$	001	1	1

Space group #152 : $P3_121$

$(hkl;d)$	weak	2^{100}	2_1^{100}
001; 0	001	1	1
001; $\frac{1}{2}$	001	0	0

Space group #153 : $P3_212$

$(hkl;d)$	weak	2^{120}	2_1^{120}
001; $\frac{1}{6}$	001	0	0
001; $\frac{2}{3}$	001	1	1

Space group #154 : $P3_221$

$(hkl;d)$	weak	2^{100}	2_1^{100}
001; 0	001	0	1
001; $\frac{1}{2}$	001	1	0

Space group #155 : $R32$

$(hkl;d)$	weak	2^{100}	2_1^{100}
$0\bar{1}1$; 0	111	1	1

Continued on next column

Supplementary Table 6 – continued

**eLC**	**Invariants**		
$0\bar{1}1$; $\frac{1}{2}$	111	0	0

Space group #156 : $P3m1$

$(hkl;d)$	weak	$m^{\bar{2}10}_{(2)}$	$g^{\bar{2}10}_{\frac{1}{2}10}$
001; d_0	001	0	0
110; 0	000	2	1

Space group #157 : $P31m$

$(hkl;d)$	weak	$m^{010}_{(2)}$	$g^{010}_{\frac{1}{2}00}$
001; d_0	001	0	0
010; 0	000	2	1

Space group #158 : $P3c1$

$(hkl;d)$	weak	$g^{\bar{2}10}_{00\frac{1}{2}}$	$g^{\bar{2}10}_{\frac{1}{2}1\frac{1}{2}}$
001; d_0	000	1	1

Space group #159 : $P31c$

$(hkl;d)$	weak	$g^{010}_{00\frac{1}{2}}$	$g^{010}_{\frac{1}{2}0\frac{1}{2}}$
001; d_0	000	1	1

Space group #160 : $R3m$

$(hkl;d)$	weak	$m^{\bar{2}10}_{(2)}$	$g^{\bar{2}10}_{\frac{1}{6}\frac{1}{3}\frac{1}{3}}$
$0\bar{1}1$; d_0	111	0	1
$\bar{1}20$; 0	000	2	1

Space group #161 : $R3c$

$(hkl;d)$	weak	$g^{\bar{2}10}_{\frac{\bar{1}}{6}\frac{\bar{1}}{3}\frac{1}{6}}$	$g^{\bar{2}10}_{00\frac{1}{2}}$
$0\bar{1}1$; d_0	000	1	1

Space group #168 : $P6$

$(hkl;d)$	weak	2^{001}	6^{001}
001; d_0	001	0	0
010; 0	000	1	1

Space group #169 : $P6_1$

$(hkl;d)$	weak	2_1^{001}	6_1^{001}
001; d_0	000	1	1

Space group #170 : $P6_5$

$(hkl;d)$	weak	2_1^{001}	6_5^{001}
001; d_0	000	1	1

Space group #171 : $P6_2$

$(hkl;d)$	weak	2^{001}	6_2^{001}
001; d_0	001	0	1
010; 0	000	1	1

Space group #172 : $P6_4$

$(hkl;d)$	weak	2^{001}	6_4^{001}
001; d_0	001	0	0
010; 0	000	1	1

Space group #173 : $P6_3$

$(hkl;d)$	weak	2_1^{001}	6_3^{001}
001; d_0	000	1	1

Space group #177 : $P622$

$(hkl;d)$	weak	2^{001}	2^{010}	$2^{1\bar{1}0}$	6^{001}	2_1^{010}	$2_1^{1\bar{1}0}$
001; 0	001	0	1	1	0	0	0
001; $\frac{1}{2}$	001	0	0	0	0	1	1
010; 0	000	1	1	0	1	1	0

Space group #178 : $P6_122$

$(hkl;d)$	weak	2^{010}	$2^{1\bar{1}0}$	2_1^{001}	2_1^{010}	$2_1^{1\bar{1}0}$	6_1^{001}
001; $\frac{1}{6}$	000	1	0	1	1	0	1
001; $\frac{1}{4}$	000	0	1	1	0	1	1

Space group #179 : $P6_522$

Continued on next column

Supplementary Table 6 – continued

eLC	Invariants						
$(hkl;d)$	weak	2^{010}	$2^{1\bar{1}0}$	2_1^{001}	2_1^{010}	$2_1^{1\bar{1}0}$	6_5^{001}
$001;\frac{1}{3}$	000	1	0	1	1	0	1
$001;\frac{1}{4}$	000	0	1	1	0	1	1

Space group #180 : $P6_222$

$(hkl;d)$	weak	2^{001}	2^{010}	$2^{1\bar{1}0}$	2_1^{010}	$2_1^{1\bar{1}0}$	6_2^{001}
$001;\frac{1}{3}$	001	0	0	1	1	0	1
$001;\frac{5}{6}$	001	0	1	0	0	1	1
$010;0$	000	1	1	0	1	0	1

Space group #181 : $P6_422$

$(hkl;d)$	weak	2^{001}	2^{010}	$2^{1\bar{1}0}$	2_1^{010}	$2_1^{1\bar{1}0}$	6_4^{001}
$001;\frac{1}{6}$	001	0	0	1	1	0	0
$001;\frac{2}{3}$	001	0	1	0	0	1	0
$010;0$	000	1	1	0	1	0	1

Space group #182 : $P6_322$

$(hkl;d)$	weak	2^{010}	$2^{1\bar{1}0}$	2_1^{001}	2_1^{010}	$2_1^{1\bar{1}0}$	6_3^{001}
$001;0$	000	1	0	1	1	0	1
$001;\frac{1}{4}$	000	0	1	1	0	1	1

Space group #183 : $P6mm$

$(hkl;d)$	weak	$m_{(2)}^{\bar{1}20}$	$m_{(2)}^{1\bar{1}0}$	$g_{1\frac{1}{2}0}^{\bar{1}20}$	$g_{\frac{1}{2}\frac{1}{2}0}^{1\bar{1}0}$	2^{001}	6^{001}
$001;d_0$	001	0	0	0	0	0	0
$010;0$	000	0	2	0	1	1	1
$110;0$	000	2	0	1	0	1	1

Space group #184 : $P6cc$

$(hkl;d)$	weak	$g_{00\frac{1}{2}}^{\bar{1}20}$	$g_{1\frac{1}{2}\frac{1}{2}}^{\bar{1}20}$	$g_{00\frac{1}{2}}^{1\bar{1}0}$	$g_{\frac{1}{2}\frac{1}{2}\frac{1}{2}}^{1\bar{1}0}$	2^{001}	6^{001}
$001;d_0$	000	1	1	1	1	0	0
$010;0$	000	0	0	1	1	1	1

Space group #185 : $P6_3cm$

$(hkl;d)$	weak	$m_{(2)}^{1\bar{1}0}$	$g_{00\frac{1}{2}}^{\bar{1}20}$	$g_{1\frac{1}{2}\frac{1}{2}}^{\bar{1}20}$	$g_{\frac{1}{2}\frac{1}{2}0}^{1\bar{1}0}$	2_1^{001}	6_3^{001}
$001;d_0$	000	0	1	1	0	1	1
$010;0$	000	2	0	0	1	1	1

Space group #186 : $P6_3mc$

$(hkl;d)$	weak	$m_{(2)}^{\bar{1}20}$	$g_{1\frac{1}{2}0}^{\bar{1}20}$	$g_{00\frac{1}{2}}^{1\bar{1}0}$	$g_{\frac{1}{2}\frac{1}{2}\frac{1}{2}}^{1\bar{1}0}$	2_1^{001}	6_3^{001}
$001;d_0$	000	0	0	1	1	1	1
$110;0$	000	2	1	0	0	1	1

Space group #195 : $P23$

$(hkl;d)$	weak	2^{001}
$001;0$	111	0

Space group #196 : $F23$

$(hkl;d)$	weak	2^{001}	2_1^{001}

Space group #197 : $I23$

$(hkl;d)$	weak	2^{001}	2_1^{001}
$110;0$	111	0	1

Space group #198 : $P2_13$

$(hkl;d)$	weak	2_1^{001}

Space group #199 : $I2_13$

$(hkl;d)$	weak	2^{001}	2_1^{001}
$110;\frac{1}{4}$	111	1	0

Space group #207 : $P432$

$(hkl;d)$	weak	2^{001}	2^{011}	4^{001}	2_1^{011}
$001;0$	111	0	1	1	0
$001;\frac{1}{2}$	111	0	0	0	1

Space group #208 : $P4_232$

$(hkl;d)$	weak	2^{001}	2^{011}	2_1^{011}	4_2^{001}
$001;\frac{1}{4}$	000	0	1	1	1

Continued on next column

Supplementary Table 6 – continued

Space group #209 : $F432$

$(hkl;d)$	weak	2^{001}	2^{011}	4^{001}	2_1^{001}	2_1^{011}	4_2^{001}
$100;0$	000	0	1	1	0	1	1

Space group #210 : $F4_132$

$(hkl;d)$	weak	2^{001}	2^{011}	2_1^{001}	2_1^{011}	4_1^{001}	4_3^{001}
$11\bar{1};\frac{1}{8}$	000	0	1	0	1	1	1

Space group #211 : $I432$

$(hkl;d)$	weak	2^{001}	2^{011}	4^{001}	2_1^{001}	2_1^{011}	4_2^{001}
$110;0$	111	0	1	1	1	0	0
$110;\frac{1}{2}$	111	0	0	0	1	1	1

Space group #212 : $P4_332$

$(hkl;d)$	weak	2^{011}	2_1^{001}	2_1^{011}	4_3^{001}
$001;\frac{3}{8}$	000	1	0	1	1

Space group #213 : $P4_132$

$(hkl;d)$	weak	2^{011}	2_1^{001}	2_1^{011}	4_1^{001}
$001;\frac{1}{8}$	000	1	0	1	1

Space group #214 : $I4_132$

$(hkl;d)$	weak	2^{001}	2^{011}	2_1^{001}	2_1^{011}	4_1^{001}	4_3^{001}
$110;\frac{1}{4}$	111	1	0	0	1	0	1
$110;\frac{3}{4}$	111	1	1	0	0	1	0

Space group #215 : $P\bar{4}3m$

$(hkl;d)$	weak	$m_{(2)}^{011}$	$g_{0\frac{\bar{1}}{2}\frac{1}{2}}^{011}$	2^{001}	$\bar{4}^{001}$
$001;0$	111	0	1	0	0
$011;0$	000	2	1	0	1

Space group #216 : $F\bar{4}3m$

$(hkl;d)$	weak	$m_{(2)}^{011}$	$g_{\frac{1}{2}\frac{\bar{1}}{4}\frac{1}{4}}^{011}$	2^{001}	2_1^{001}	$\bar{4}^{001}$
$0\bar{1}1;0$	000	2	1	0	0	1

Space group #217 : $I\bar{4}3m$

$(hkl;d)$	weak	$m_{(2)}^{011}$	$g_{\frac{1}{2}00}^{011}$	2^{001}	2_1^{001}	$\bar{4}^{001}$
$110;0$	111	2	0	0	1	1
$110;\frac{1}{2}$	111	0	1	0	1	0

Space group #218 : $P\bar{4}3n$

$(hkl;d)$	weak	$g_{\frac{1}{2}00}^{011}$	$g_{\frac{1}{2}\frac{\bar{1}}{2}\frac{1}{2}}^{011}$	2^{001}	$\bar{4}^{001}$
$001;\frac{1}{4}$	000	1	1	0	1

Space group #219 : $F\bar{4}3c$

$(hkl;d)$	weak	$g_{\frac{1}{2}00}^{011}$	$g_{0\frac{\bar{1}}{4}\frac{1}{4}}^{011}$	2^{001}	2_1^{001}	$\bar{4}^{001}$
$100;0$	000	1	1	0	0	1

Space group #220 : $I\bar{4}3d$

$(hkl;d)$	weak	$g_{\frac{1}{4}\frac{1}{4}\frac{\bar{1}}{4}}^{011}$	$g_{\frac{1}{4}\frac{\bar{1}}{4}\frac{1}{4}}^{011}$	2^{001}	2_1^{001}	$\bar{4}^{001}$
$110;\frac{1}{4}$	000	1	1	0	0	1

Supplementary Table 7: Mapping from SI to TCI invariants in all SGs with nontrivial SI groups.

**SI**	**Invariants**

Space group #2 : $P\bar{1}$

$\mathbb{Z}_{2,2,2,4}$	weak	i
0000	000	0
0002	000	1
0010	001	0
0012	001	1
0100	010	0
0102	010	1
0110	011	0
0112	011	1
1000	100	0
1002	100	1
1010	101	0
1012	101	1
1100	110	0
1102	110	1
1110	111	0
1112	111	1

Space group #10 : $P2/m$

$\mathbb{Z}_{2,2,2,4}$	weak	$m_{(2)}^{010}$	2^{010}	i
0000	000	00	0	0
0000	000	02	1	0
0000	000	20	1	0
0000	000	22	0	0
0002	000	00	1	1
0002	000	02	0	1
0002	000	20	0	1
0002	000	22	1	1
0010	001	00	0	0
0010	001	02	1	0
0010	001	20	1	0
0010	001	22	0	0
0012	001	00	1	1
0012	001	02	0	1
0012	001	20	0	1
0012	001	22	1	1
0100	010	11	1	0
0100	010	$1\bar{1}$	0	0
0100	010	$\bar{1}1$	0	0
0100	010	$\bar{1}\bar{1}$	1	0
0102	010	11	0	1
0102	010	$1\bar{1}$	1	1
0102	010	$\bar{1}1$	1	1
0102	010	$\bar{1}\bar{1}$	0	1
0110	011	11	1	0
0110	011	$1\bar{1}$	0	0
0110	011	$\bar{1}1$	0	0
0110	011	$\bar{1}\bar{1}$	1	0
0112	011	11	0	1
0112	011	$1\bar{1}$	1	1
0112	011	$\bar{1}1$	1	1
0112	011	$\bar{1}\bar{1}$	0	1
1000	100	00	0	0
1000	100	02	1	0
1000	100	20	1	0
1000	100	22	0	0
1002	100	00	1	1
1002	100	02	0	1
1002	100	20	0	1

Continued on next page

Supplementary Table 7 – continued

**SI**	**Invariants**			
1002	100	22	1	1
1010	101	00	0	0
1010	101	02	1	0
1010	101	20	1	0
1010	101	22	0	0
1012	101	00	1	1
1012	101	02	0	1
1012	101	20	0	1
1012	101	22	1	1
1100	110	11	1	0
1100	110	$1\bar{1}$	0	0
1100	110	$\bar{1}1$	0	0
1100	110	$\bar{1}\bar{1}$	1	0
1102	110	11	0	1
1102	110	$1\bar{1}$	1	1
1102	110	$\bar{1}1$	1	1
1102	110	$\bar{1}\bar{1}$	0	1
1110	111	11	1	0
1110	111	$1\bar{1}$	0	0
1110	111	$\bar{1}1$	0	0
1110	111	$\bar{1}\bar{1}$	1	0
1112	111	11	0	1
1112	111	$1\bar{1}$	1	1
1112	111	$\bar{1}1$	1	1
1112	111	$\bar{1}\bar{1}$	0	1

Space group #11 : $P2_1/m$

$\mathbb{Z}_{2,2,2,4}$	weak	$m_{(2)}^{010}$	i	2_1^{010}
0000	000	00	0	0
0000	000	20	0	$\mathbb{1}$
0002	000	00	$\mathbb{1}$	$\mathbb{1}$
0002	000	20	$\mathbb{1}$	0
0010	001	00	0	0
0010	001	20	0	1
0012	001	00	1	1
0012	001	20	1	0
1000	100	00	0	0
1000	100	20	0	1
1002	100	00	1	1
1002	100	20	1	0
1010	101	00	0	0
1010	101	20	0	1
1012	101	00	1	1
1012	101	20	1	0

Space group #12 : $C2/m$

$\mathbb{Z}_{2,2,2,4}$	weak	$m_{(2)}^{010}$	$g_{\frac{1}{2}00}^{010}$	2^{010}	i	2_1^{010}
0000	000	0	0	0	0	0
0000	000	2	$\mathbb{1}$	$\mathbb{1}$	0	$\mathbb{1}$
0002	000	0	0	$\mathbb{1}$	$\mathbb{1}$	$\mathbb{1}$
0002	000	2	$\mathbb{1}$	0	$\mathbb{1}$	0
0010	001	0	0	0	0	0
0010	001	2	$\mathbb{1}$	1	0	1
0012	001	0	0	1	1	1
0012	001	2	$\mathbb{1}$	0	1	0
1100	110	0	$\mathbb{1}$	0	0	$\mathbb{1}$
1100	110	2	0	$\mathbb{1}$	0	0
1102	110	0	$\mathbb{1}$	$\mathbb{1}$	1	0
1102	110	2	0	0	1	$\mathbb{1}$
1110	111	0	$\mathbb{1}$	0	0	1
1110	111	2	0	1	0	0
1112	111	0	$\mathbb{1}$	1	1	0

Continued on next page

Supplementary Table 7 – continued

**SI**	**Invariants**					
1112	111	2	0	0	1	1

Space group #13 : $P2/c$

$\mathbb{Z}_{2,2,2,4}$	weak	$g^{010}_{00\frac{1}{2}}$	2^{010}	i
0000	000	0	0	0
0000	000	$\mathbb{1}$	$\mathbb{1}$	0
0002	000	0	$\mathbb{1}$	$\mathbb{1}$
0002	000	$\mathbb{1}$	0	$\mathbb{1}$
0100	010	0	0	0
0100	010	1	$\mathbb{1}$	0
0102	010	0	$\mathbb{1}$	1
0102	010	1	0	1
1000	100	0	0	0
1000	100	$\mathbb{1}$	1	0
1002	100	0	1	1
1002	100	$\mathbb{1}$	0	1
1100	110	0	0	0
1100	110	1	1	0
1102	110	0	1	1
1102	110	1	0	1

Space group #14 : $P2_1/c$

$\mathbb{Z}_{2,2,2,4}$	weak	$g^{010}_{00\frac{1}{2}}$	i	2^{010}_1
0000	000	0	0	0
0000	000	$\mathbb{1}$	0	$\mathbb{1}$
0002	000	0	$\mathbb{1}$	$\mathbb{1}$
0002	000	$\mathbb{1}$	$\mathbb{1}$	0
1000	100	0	0	0
1000	100	$\mathbb{1}$	0	1
1002	100	0	1	1
1002	100	$\mathbb{1}$	1	0

Space group #15 : $C2/c$

$\mathbb{Z}_{2,2,2,4}$	weak	$g^{010}_{00\frac{1}{2}}$	$g^{010}_{\frac{\bar{1}}{2}0\frac{1}{2}}$	2^{010}	i	2^{010}_1
0000	000	0	0	0	0	0
0000	000	$\mathbb{1}$	$\mathbb{1}$	$\mathbb{1}$	0	$\mathbb{1}$
0002	000	0	0	$\mathbb{1}$	$\mathbb{1}$	$\mathbb{1}$
0002	000	$\mathbb{1}$	$\mathbb{1}$	0	$\mathbb{1}$	0
1100	110	0	$\mathbb{1}$	0	0	$\mathbb{1}$
1100	110	$\mathbb{1}$	0	$\mathbb{1}$	0	0
1102	110	0	$\mathbb{1}$	$\mathbb{1}$	1	0
1102	110	$\mathbb{1}$	0	0	1	$\mathbb{1}$

Space group #47 : $Pmmm$

$\mathbb{Z}_{2,2,2,4}$	weak	$m^{001}_{(2)}$	$m^{010}_{(2)}$	$m^{100}_{(2)}$	2^{001}	2^{010}	2^{100}	i
0000	000	00	00	00	0	0	0	0
0000	000	00	00	22	0	0	0	0
0000	000	00	02	02	0	$\mathbb{1}$	$\mathbb{1}$	0
0000	000	00	02	20	0	$\mathbb{1}$	$\mathbb{1}$	0
0000	000	00	20	02	0	$\mathbb{1}$	$\mathbb{1}$	0
0000	000	00	20	20	0	$\mathbb{1}$	$\mathbb{1}$	0
0000	000	00	22	00	0	0	0	0
0000	000	00	22	22	0	0	0	0
0000	000	02	00	02	$\mathbb{1}$	0	$\mathbb{1}$	0
0000	000	02	00	20	$\mathbb{1}$	0	$\mathbb{1}$	0
0000	000	02	02	00	$\mathbb{1}$	$\mathbb{1}$	0	0
0000	000	02	02	22	$\mathbb{1}$	$\mathbb{1}$	0	0
0000	000	02	20	00	$\mathbb{1}$	$\mathbb{1}$	0	0
0000	000	02	20	22	$\mathbb{1}$	$\mathbb{1}$	0	0
0000	000	02	22	02	$\mathbb{1}$	0	$\mathbb{1}$	0
0000	000	02	22	20	$\mathbb{1}$	0	$\mathbb{1}$	0
0000	000	20	00	02	$\mathbb{1}$	0	$\mathbb{1}$	0

Continued on next page

Supplementary Table 7 – continued

SI	Invariants							
0000	000	20	00	20	$\mathbb{1}$	0	$\mathbb{1}$	0
0000	000	20	02	00	$\mathbb{1}$	$\mathbb{1}$	0	0
0000	000	20	02	22	$\mathbb{1}$	$\mathbb{1}$	0	0
0000	000	20	20	00	$\mathbb{1}$	$\mathbb{1}$	0	0
0000	000	20	20	22	$\mathbb{1}$	$\mathbb{1}$	0	0
0000	000	20	22	02	$\mathbb{1}$	0	$\mathbb{1}$	0
0000	000	20	22	20	$\mathbb{1}$	0	$\mathbb{1}$	0
0000	000	22	00	00	0	0	0	0
0000	000	22	00	22	0	0	0	0
0000	000	22	02	02	0	$\mathbb{1}$	$\mathbb{1}$	0
0000	000	22	02	20	0	$\mathbb{1}$	$\mathbb{1}$	0
0000	000	22	20	02	0	$\mathbb{1}$	$\mathbb{1}$	0
0000	000	22	20	20	0	$\mathbb{1}$	$\mathbb{1}$	0
0000	000	22	22	00	0	0	0	0
0000	000	22	22	22	0	0	0	0
0002	000	00	00	02	$\mathbb{1}$	$\mathbb{1}$	0	$\mathbb{1}$
0002	000	00	00	20	$\mathbb{1}$	$\mathbb{1}$	0	$\mathbb{1}$
0002	000	00	02	00	$\mathbb{1}$	0	$\mathbb{1}$	$\mathbb{1}$
0002	000	00	02	22	$\mathbb{1}$	0	$\mathbb{1}$	$\mathbb{1}$
0002	000	00	20	00	$\mathbb{1}$	0	$\mathbb{1}$	$\mathbb{1}$
0002	000	00	20	22	$\mathbb{1}$	0	$\mathbb{1}$	$\mathbb{1}$
0002	000	00	22	02	$\mathbb{1}$	$\mathbb{1}$	0	$\mathbb{1}$
0002	000	00	22	20	$\mathbb{1}$	$\mathbb{1}$	0	$\mathbb{1}$
0002	000	02	00	00	0	$\mathbb{1}$	$\mathbb{1}$	$\mathbb{1}$
0002	000	02	00	22	0	$\mathbb{1}$	$\mathbb{1}$	$\mathbb{1}$
0002	000	02	02	02	0	0	0	$\mathbb{1}$
0002	000	02	02	20	0	0	0	$\mathbb{1}$
0002	000	02	20	02	0	0	0	$\mathbb{1}$
0002	000	02	20	20	0	0	0	$\mathbb{1}$
0002	000	02	22	00	0	$\mathbb{1}$	$\mathbb{1}$	$\mathbb{1}$
0002	000	02	22	22	0	$\mathbb{1}$	$\mathbb{1}$	$\mathbb{1}$
0002	000	20	00	00	0	$\mathbb{1}$	$\mathbb{1}$	$\mathbb{1}$
0002	000	20	00	22	0	$\mathbb{1}$	$\mathbb{1}$	$\mathbb{1}$
0002	000	20	02	02	0	0	0	$\mathbb{1}$
0002	000	20	02	20	0	0	0	$\mathbb{1}$
0002	000	20	20	02	0	0	0	$\mathbb{1}$
0002	000	20	20	20	0	0	0	$\mathbb{1}$
0002	000	20	22	00	0	$\mathbb{1}$	$\mathbb{1}$	$\mathbb{1}$
0002	000	20	22	22	0	$\mathbb{1}$	$\mathbb{1}$	$\mathbb{1}$
0002	000	22	00	02	$\mathbb{1}$	$\mathbb{1}$	0	$\mathbb{1}$
0002	000	22	00	20	$\mathbb{1}$	$\mathbb{1}$	0	$\mathbb{1}$
0002	000	22	02	00	$\mathbb{1}$	0	$\mathbb{1}$	$\mathbb{1}$
0002	000	22	02	22	$\mathbb{1}$	0	$\mathbb{1}$	$\mathbb{1}$
0002	000	22	20	00	$\mathbb{1}$	0	$\mathbb{1}$	$\mathbb{1}$
0002	000	22	20	22	$\mathbb{1}$	0	$\mathbb{1}$	$\mathbb{1}$
0002	000	22	22	02	$\mathbb{1}$	$\mathbb{1}$	0	$\mathbb{1}$
0002	000	22	22	20	$\mathbb{1}$	$\mathbb{1}$	0	$\mathbb{1}$
0010	001	11	00	02	$\mathbb{1}$	0	1	0
0010	001	11	00	20	$\mathbb{1}$	0	1	0
0010	001	11	02	00	$\mathbb{1}$	1	0	0
0010	001	11	02	22	$\mathbb{1}$	1	0	0
0010	001	11	20	00	$\mathbb{1}$	1	0	0
0010	001	11	20	22	$\mathbb{1}$	1	0	0
0010	001	11	22	02	$\mathbb{1}$	0	1	0
0010	001	11	22	20	$\mathbb{1}$	0	1	0
0010	001	$1\bar{1}$	00	00	0	0	0	0
0010	001	$1\bar{1}$	00	22	0	0	0	0
0010	001	$1\bar{1}$	02	02	0	1	1	0
0010	001	$1\bar{1}$	02	20	0	1	1	0
0010	001	$1\bar{1}$	20	02	0	1	1	0
0010	001	$1\bar{1}$	20	20	0	1	1	0

Continued on next page

Supplementary Table 7 – continued

SI	Invariants							
0010	001	$1\bar{1}$	22	00	0	0	0	0
0010	001	$1\bar{1}$	22	22	0	0	0	0
0010	001	$\bar{1}1$	00	00	0	0	0	0
0010	001	$\bar{1}1$	00	22	0	0	0	0
0010	001	$\bar{1}1$	02	02	0	1	1	0
0010	001	$\bar{1}1$	02	20	0	1	1	0
0010	001	$\bar{1}1$	20	02	0	1	1	0
0010	001	$\bar{1}1$	20	20	0	1	1	0
0010	001	$\bar{1}1$	22	00	0	0	0	0
0010	001	$\bar{1}1$	22	22	0	0	0	0
0010	001	$\bar{1}\bar{1}$	00	02	$\mathbb{1}$	0	1	0
0010	001	$\bar{1}\bar{1}$	00	20	$\mathbb{1}$	0	1	0
0010	001	$\bar{1}\bar{1}$	02	00	$\mathbb{1}$	1	0	0
0010	001	$\bar{1}\bar{1}$	02	22	$\mathbb{1}$	1	0	0
0010	001	$\bar{1}\bar{1}$	20	00	$\mathbb{1}$	1	0	0
0010	001	$\bar{1}\bar{1}$	20	22	$\mathbb{1}$	1	0	0
0010	001	$\bar{1}\bar{1}$	22	02	$\mathbb{1}$	0	1	0
0010	001	$\bar{1}\bar{1}$	22	20	$\mathbb{1}$	0	1	0
0012	001	11	00	00	0	1	1	1
0012	001	11	00	22	0	1	1	1
0012	001	11	02	02	0	0	0	1
0012	001	11	02	20	0	0	0	1
0012	001	11	20	02	0	0	0	1
0012	001	11	20	20	0	0	0	1
0012	001	11	22	00	0	1	1	1
0012	001	11	22	22	0	1	1	1
0012	001	$1\bar{1}$	00	02	$\mathbb{1}$	1	0	1
0012	001	$1\bar{1}$	00	20	$\mathbb{1}$	1	0	1
0012	001	$1\bar{1}$	02	00	$\mathbb{1}$	0	1	1
0012	001	$1\bar{1}$	02	22	$\mathbb{1}$	0	1	1
0012	001	$1\bar{1}$	20	00	$\mathbb{1}$	0	1	1
0012	001	$1\bar{1}$	20	22	$\mathbb{1}$	0	1	1
0012	001	$1\bar{1}$	22	02	$\mathbb{1}$	1	0	1
0012	001	$1\bar{1}$	22	20	$\mathbb{1}$	1	0	1
0012	001	$\bar{1}1$	00	02	$\mathbb{1}$	1	0	1
0012	001	$\bar{1}1$	00	20	$\mathbb{1}$	1	0	1
0012	001	$\bar{1}1$	02	00	$\mathbb{1}$	0	1	1
0012	001	$\bar{1}1$	02	22	$\mathbb{1}$	0	1	1
0012	001	$\bar{1}1$	20	00	$\mathbb{1}$	0	1	1
0012	001	$\bar{1}1$	20	22	$\mathbb{1}$	0	1	1
0012	001	$\bar{1}1$	22	02	$\mathbb{1}$	1	0	1
0012	001	$\bar{1}1$	22	20	$\mathbb{1}$	1	0	1
0012	001	$\bar{1}\bar{1}$	00	00	0	1	1	1
0012	001	$\bar{1}\bar{1}$	00	22	0	1	1	1
0012	001	$\bar{1}\bar{1}$	02	02	0	0	0	1
0012	001	$\bar{1}\bar{1}$	02	20	0	0	0	1
0012	001	$\bar{1}\bar{1}$	20	02	0	0	0	1
0012	001	$\bar{1}\bar{1}$	20	20	0	0	0	1
0012	001	$\bar{1}\bar{1}$	22	00	0	1	1	1
0012	001	$\bar{1}\bar{1}$	22	22	0	1	1	1
0100	010	00	11	02	0	$\mathbb{1}$	1	0
0100	010	00	11	20	0	$\mathbb{1}$	1	0
0100	010	00	$1\bar{1}$	00	0	0	0	0
0100	010	00	$1\bar{1}$	22	0	0	0	0
0100	010	00	$\bar{1}1$	00	0	0	0	0
0100	010	00	$\bar{1}1$	22	0	0	0	0
0100	010	00	$\bar{1}\bar{1}$	02	0	$\mathbb{1}$	1	0
0100	010	00	$\bar{1}\bar{1}$	20	0	$\mathbb{1}$	1	0
0100	010	02	11	00	1	$\mathbb{1}$	0	0
0100	010	02	11	22	1	$\mathbb{1}$	0	0
0100	010	02	$1\bar{1}$	02	1	0	1	0

Continued on next page

Supplementary Table 7 – continued

SI	Invariants							
0100	010	02	$1\bar{1}$	20	1	0	1	0
0100	010	02	$\bar{1}1$	02	1	0	1	0
0100	010	02	$\bar{1}1$	20	1	0	1	0
0100	010	02	$\bar{1}\bar{1}$	00	1	1	0	0
0100	010	02	$\bar{1}\bar{1}$	22	1	1	0	0
0100	010	20	11	00	1	1	0	0
0100	010	20	11	22	1	1	0	0
0100	010	20	$1\bar{1}$	02	1	0	1	0
0100	010	20	$1\bar{1}$	20	1	0	1	0
0100	010	20	$\bar{1}1$	02	1	0	1	0
0100	010	20	$\bar{1}1$	20	1	0	1	0
0100	010	20	$\bar{1}\bar{1}$	00	1	1	0	0
0100	010	20	$\bar{1}\bar{1}$	22	1	1	0	0
0100	010	22	11	02	0	1	1	0
0100	010	22	11	20	0	1	1	0
0100	010	22	$1\bar{1}$	00	0	0	0	0
0100	010	22	$1\bar{1}$	22	0	0	0	0
0100	010	22	$\bar{1}1$	00	0	0	0	0
0100	010	22	$\bar{1}1$	22	0	0	0	0
0100	010	22	$\bar{1}\bar{1}$	02	0	1	1	0
0100	010	22	$\bar{1}\bar{1}$	20	0	1	1	0
0102	010	00	11	00	1	0	1	1
0102	010	00	11	22	1	0	1	1
0102	010	00	$1\bar{1}$	02	1	1	0	1
0102	010	00	$1\bar{1}$	20	1	1	0	1
0102	010	00	$\bar{1}1$	02	1	1	0	1
0102	010	00	$\bar{1}1$	20	1	1	0	1
0102	010	00	$\bar{1}\bar{1}$	00	1	0	1	1
0102	010	00	$\bar{1}\bar{1}$	22	1	0	1	1
0102	010	02	11	02	0	0	0	1
0102	010	02	11	20	0	0	0	1
0102	010	02	$1\bar{1}$	00	0	1	1	1
0102	010	02	$1\bar{1}$	22	0	1	1	1
0102	010	02	$\bar{1}1$	00	0	1	1	1
0102	010	02	$\bar{1}1$	22	0	1	1	1
0102	010	02	$\bar{1}\bar{1}$	02	0	0	0	1
0102	010	02	$\bar{1}\bar{1}$	20	0	0	0	1
0102	010	20	11	02	0	0	0	1
0102	010	20	11	20	0	0	0	1
0102	010	20	$1\bar{1}$	00	0	1	1	1
0102	010	20	$1\bar{1}$	22	0	1	1	1
0102	010	20	$\bar{1}1$	00	0	1	1	1
0102	010	20	$\bar{1}1$	22	0	1	1	1
0102	010	20	$\bar{1}\bar{1}$	02	0	0	0	1
0102	010	20	$\bar{1}\bar{1}$	20	0	0	0	1
0102	010	22	11	00	1	0	1	1
0102	010	22	11	22	1	0	1	1
0102	010	22	$1\bar{1}$	02	1	1	0	1
0102	010	22	$1\bar{1}$	20	1	1	0	1
0102	010	22	$\bar{1}1$	02	1	1	0	1
0102	010	22	$\bar{1}1$	20	1	1	0	1
0102	010	22	$\bar{1}\bar{1}$	00	1	0	1	1
0102	010	22	$\bar{1}\bar{1}$	22	1	0	1	1
0110	011	11	11	00	1	1	0	0
0110	011	11	11	22	1	1	0	0
0110	011	11	$1\bar{1}$	02	1	0	1	0
0110	011	11	$1\bar{1}$	20	1	0	1	0
0110	011	11	$\bar{1}1$	02	1	0	1	0
0110	011	11	$\bar{1}1$	20	1	0	1	0
0110	011	11	$\bar{1}\bar{1}$	00	1	1	0	0
0110	011	11	$\bar{1}\bar{1}$	22	1	1	0	0

Continued on next page

Supplementary Table 7 – continued

SI	Invariants							
0110	011	$1\bar{1}$	11	02	0	1	1	0
0110	011	$1\bar{1}$	11	20	0	1	1	0
0110	011	$1\bar{1}$	$1\bar{1}$	00	0	0	0	0
0110	011	$1\bar{1}$	$1\bar{1}$	22	0	0	0	0
0110	011	$1\bar{1}$	$\bar{1}1$	00	0	0	0	0
0110	011	$1\bar{1}$	$\bar{1}1$	22	0	0	0	0
0110	011	$1\bar{1}$	$\bar{1}\bar{1}$	02	0	1	1	0
0110	011	$1\bar{1}$	$\bar{1}\bar{1}$	20	0	1	1	0
0110	011	$\bar{1}1$	11	02	0	1	1	0
0110	011	$\bar{1}1$	11	20	0	1	1	0
0110	011	$\bar{1}1$	$1\bar{1}$	00	0	0	0	0
0110	011	$\bar{1}1$	$1\bar{1}$	22	0	0	0	0
0110	011	$\bar{1}1$	$\bar{1}1$	00	0	0	0	0
0110	011	$\bar{1}1$	$\bar{1}1$	22	0	0	0	0
0110	011	$\bar{1}1$	$\bar{1}\bar{1}$	02	0	1	1	0
0110	011	$\bar{1}1$	$\bar{1}\bar{1}$	20	0	1	1	0
0110	011	$\bar{1}\bar{1}$	11	00	1	1	0	0
0110	011	$\bar{1}\bar{1}$	11	22	1	1	0	0
0110	011	$\bar{1}\bar{1}$	$1\bar{1}$	02	1	0	1	0
0110	011	$\bar{1}\bar{1}$	$1\bar{1}$	20	1	0	1	0
0110	011	$\bar{1}\bar{1}$	$\bar{1}1$	02	1	0	1	0
0110	011	$\bar{1}\bar{1}$	$\bar{1}1$	20	1	0	1	0
0110	011	$\bar{1}\bar{1}$	$\bar{1}\bar{1}$	00	1	1	0	0
0110	011	$\bar{1}\bar{1}$	$\bar{1}\bar{1}$	22	1	1	0	0
0112	011	11	11	02	0	0	0	1
0112	011	11	11	20	0	0	0	1
0112	011	11	$1\bar{1}$	00	0	1	1	1
0112	011	11	$1\bar{1}$	22	0	1	1	1
0112	011	11	$\bar{1}1$	00	0	1	1	1
0112	011	11	$\bar{1}1$	22	0	1	1	1
0112	011	11	$\bar{1}\bar{1}$	02	0	0	0	1
0112	011	11	$\bar{1}\bar{1}$	20	0	0	0	1
0112	011	$1\bar{1}$	11	00	1	0	1	1
0112	011	$1\bar{1}$	11	22	1	0	1	1
0112	011	$1\bar{1}$	$1\bar{1}$	02	1	1	0	1
0112	011	$1\bar{1}$	$1\bar{1}$	20	1	1	0	1
0112	011	$1\bar{1}$	$\bar{1}1$	02	1	1	0	1
0112	011	$1\bar{1}$	$\bar{1}1$	20	1	1	0	1
0112	011	$1\bar{1}$	$\bar{1}\bar{1}$	00	1	0	1	1
0112	011	$1\bar{1}$	$\bar{1}\bar{1}$	22	1	0	1	1
0112	011	$\bar{1}1$	11	00	1	0	1	1
0112	011	$\bar{1}1$	11	22	1	0	1	1
0112	011	$\bar{1}1$	$1\bar{1}$	02	1	1	0	1
0112	011	$\bar{1}1$	$1\bar{1}$	20	1	1	0	1
0112	011	$\bar{1}1$	$\bar{1}1$	02	1	1	0	1
0112	011	$\bar{1}1$	$\bar{1}1$	20	1	1	0	1
0112	011	$\bar{1}1$	$\bar{1}\bar{1}$	00	1	0	1	1
0112	011	$\bar{1}1$	$\bar{1}\bar{1}$	22	1	0	1	1
0112	011	$\bar{1}\bar{1}$	11	02	0	0	0	1
0112	011	$\bar{1}\bar{1}$	11	20	0	0	0	1
0112	011	$\bar{1}\bar{1}$	$1\bar{1}$	00	0	1	1	1
0112	011	$\bar{1}\bar{1}$	$1\bar{1}$	22	0	1	1	1
0112	011	$\bar{1}\bar{1}$	$\bar{1}1$	00	0	1	1	1
0112	011	$\bar{1}\bar{1}$	$\bar{1}1$	22	0	1	1	1
0112	011	$\bar{1}\bar{1}$	$\bar{1}\bar{1}$	02	0	0	0	1
0112	011	$\bar{1}\bar{1}$	$\bar{1}\bar{1}$	20	0	0	0	1
1000	100	00	00	$1\bar{1}$	0	0	0	0
1000	100	00	00	$\bar{1}1$	0	0	0	0
1000	100	00	02	11	0	1	1	0
1000	100	00	02	$\bar{1}\bar{1}$	0	1	1	0
1000	100	00	20	11	0	1	1	0

Continued on next page

Supplementary Table 7 – continued

SI	Invariants							
1000	100	00	20	$\bar{1}\bar{1}$	0	1	$\mathbb{1}$	0
1000	100	00	22	$1\bar{1}$	0	0	0	0
1000	100	00	22	$\bar{1}1$	0	0	0	0
1000	100	02	00	11	1	0	$\mathbb{1}$	0
1000	100	02	00	$\bar{1}\bar{1}$	1	0	$\mathbb{1}$	0
1000	100	02	02	$1\bar{1}$	1	1	0	0
1000	100	02	02	$\bar{1}1$	1	1	0	0
1000	100	02	20	$1\bar{1}$	1	1	0	0
1000	100	02	20	$\bar{1}1$	1	1	0	0
1000	100	02	22	11	1	0	$\mathbb{1}$	0
1000	100	02	22	$\bar{1}\bar{1}$	1	0	$\mathbb{1}$	0
1000	100	20	00	11	1	0	$\mathbb{1}$	0
1000	100	20	00	$\bar{1}\bar{1}$	1	0	$\mathbb{1}$	0
1000	100	20	02	$1\bar{1}$	1	1	0	0
1000	100	20	02	$\bar{1}1$	1	1	0	0
1000	100	20	20	$1\bar{1}$	1	1	0	0
1000	100	20	20	$\bar{1}1$	1	1	0	0
1000	100	20	22	11	1	0	$\mathbb{1}$	0
1000	100	20	22	$\bar{1}\bar{1}$	1	0	$\mathbb{1}$	0
1000	100	22	00	$1\bar{1}$	0	0	0	0
1000	100	22	00	$\bar{1}1$	0	0	0	0
1000	100	22	02	11	0	1	$\mathbb{1}$	0
1000	100	22	02	$\bar{1}\bar{1}$	0	1	$\mathbb{1}$	0
1000	100	22	20	11	0	1	$\mathbb{1}$	0
1000	100	22	20	$\bar{1}\bar{1}$	0	1	$\mathbb{1}$	0
1000	100	22	22	$1\bar{1}$	0	0	0	0
1000	100	22	22	$\bar{1}1$	0	0	0	0
1002	100	00	00	11	1	1	0	1
1002	100	00	00	$\bar{1}\bar{1}$	1	1	0	1
1002	100	00	02	$1\bar{1}$	1	0	$\mathbb{1}$	1
1002	100	00	02	$\bar{1}1$	1	0	$\mathbb{1}$	1
1002	100	00	20	$1\bar{1}$	1	0	$\mathbb{1}$	1
1002	100	00	20	$\bar{1}1$	1	0	$\mathbb{1}$	1
1002	100	00	22	11	1	1	0	1
1002	100	00	22	$\bar{1}\bar{1}$	1	1	0	1
1002	100	02	00	$1\bar{1}$	0	1	$\mathbb{1}$	1
1002	100	02	00	$\bar{1}1$	0	1	$\mathbb{1}$	1
1002	100	02	02	11	0	0	0	1
1002	100	02	02	$\bar{1}\bar{1}$	0	0	0	1
1002	100	02	20	11	0	0	0	1
1002	100	02	20	$\bar{1}\bar{1}$	0	0	0	1
1002	100	02	22	$1\bar{1}$	0	1	$\mathbb{1}$	1
1002	100	02	22	$\bar{1}1$	0	1	$\mathbb{1}$	1
1002	100	20	00	$1\bar{1}$	0	1	$\mathbb{1}$	1
1002	100	20	00	$\bar{1}1$	0	1	$\mathbb{1}$	1
1002	100	20	02	11	0	0	0	1
1002	100	20	02	$\bar{1}\bar{1}$	0	0	0	1
1002	100	20	20	11	0	0	0	1
1002	100	20	20	$\bar{1}\bar{1}$	0	0	0	1
1002	100	20	22	$1\bar{1}$	0	1	$\mathbb{1}$	1
1002	100	20	22	$\bar{1}1$	0	1	$\mathbb{1}$	1
1002	100	22	00	11	1	1	0	1
1002	100	22	00	$\bar{1}\bar{1}$	1	1	0	1
1002	100	22	02	$1\bar{1}$	1	0	$\mathbb{1}$	1
1002	100	22	02	$\bar{1}1$	1	0	$\mathbb{1}$	1
1002	100	22	20	$1\bar{1}$	1	0	$\mathbb{1}$	1
1002	100	22	20	$\bar{1}1$	1	0	$\mathbb{1}$	1
1002	100	22	22	11	1	1	0	1
1002	100	22	22	$\bar{1}\bar{1}$	1	1	0	1
1010	101	11	00	11	1	0	1	0
1010	101	11	00	$\bar{1}\bar{1}$	1	0	1	0

Continued on next page

Supplementary Table 7 – continued

SI	Invariants							
1010	101	11	02	$1\bar{1}$	1	1	0	0
1010	101	11	02	$\bar{1}1$	1	1	0	0
1010	101	11	20	$1\bar{1}$	1	1	0	0
1010	101	11	20	$\bar{1}1$	1	1	0	0
1010	101	11	22	11	1	0	1	0
1010	101	11	22	$\bar{1}\bar{1}$	1	0	1	0
1010	101	$1\bar{1}$	00	$1\bar{1}$	0	0	0	0
1010	101	$1\bar{1}$	00	$\bar{1}1$	0	0	0	0
1010	101	$1\bar{1}$	02	11	0	1	1	0
1010	101	$1\bar{1}$	02	$\bar{1}\bar{1}$	0	1	1	0
1010	101	$1\bar{1}$	20	11	0	1	1	0
1010	101	$1\bar{1}$	20	$\bar{1}\bar{1}$	0	1	1	0
1010	101	$1\bar{1}$	22	$1\bar{1}$	0	0	0	0
1010	101	$1\bar{1}$	22	$\bar{1}1$	0	0	0	0
1010	101	$\bar{1}1$	00	$1\bar{1}$	0	0	0	0
1010	101	$\bar{1}1$	00	$\bar{1}1$	0	0	0	0
1010	101	$\bar{1}1$	02	11	0	1	1	0
1010	101	$\bar{1}1$	02	$\bar{1}\bar{1}$	0	1	1	0
1010	101	$\bar{1}1$	20	11	0	1	1	0
1010	101	$\bar{1}1$	20	$\bar{1}\bar{1}$	0	1	1	0
1010	101	$\bar{1}1$	22	$1\bar{1}$	0	0	0	0
1010	101	$\bar{1}1$	22	$\bar{1}1$	0	0	0	0
1010	101	$\bar{1}\bar{1}$	00	11	1	0	1	0
1010	101	$\bar{1}\bar{1}$	00	$\bar{1}\bar{1}$	1	0	1	0
1010	101	$\bar{1}\bar{1}$	02	$1\bar{1}$	1	1	0	0
1010	101	$\bar{1}\bar{1}$	02	$\bar{1}1$	1	1	0	0
1010	101	$\bar{1}\bar{1}$	20	$1\bar{1}$	1	1	0	0
1010	101	$\bar{1}\bar{1}$	20	$\bar{1}1$	1	1	0	0
1010	101	$\bar{1}\bar{1}$	22	11	1	0	1	0
1010	101	$\bar{1}\bar{1}$	22	$\bar{1}\bar{1}$	1	0	1	0
1012	101	11	00	$1\bar{1}$	0	1	1	1
1012	101	11	00	$\bar{1}1$	0	1	1	1
1012	101	11	02	11	0	0	0	1
1012	101	11	02	$\bar{1}\bar{1}$	0	0	0	1
1012	101	11	20	11	0	0	0	1
1012	101	11	20	$\bar{1}\bar{1}$	0	0	0	1
1012	101	11	22	$1\bar{1}$	0	1	1	1
1012	101	11	22	$\bar{1}1$	0	1	1	1
1012	101	$1\bar{1}$	00	11	1	1	0	1
1012	101	$1\bar{1}$	00	$\bar{1}\bar{1}$	1	1	0	1
1012	101	$1\bar{1}$	02	$1\bar{1}$	1	0	1	1
1012	101	$1\bar{1}$	02	$\bar{1}1$	1	0	1	1
1012	101	$1\bar{1}$	20	$1\bar{1}$	1	0	1	1
1012	101	$1\bar{1}$	20	$\bar{1}1$	1	0	1	1
1012	101	$1\bar{1}$	22	11	1	1	0	1
1012	101	$1\bar{1}$	22	$\bar{1}\bar{1}$	1	1	0	1
1012	101	$\bar{1}1$	00	11	1	1	0	1
1012	101	$\bar{1}1$	00	$\bar{1}\bar{1}$	1	1	0	1
1012	101	$\bar{1}1$	02	$1\bar{1}$	1	0	1	1
1012	101	$\bar{1}1$	02	$\bar{1}1$	1	0	1	1
1012	101	$\bar{1}1$	20	$1\bar{1}$	1	0	1	1
1012	101	$\bar{1}1$	20	$\bar{1}1$	1	0	1	1
1012	101	$\bar{1}1$	22	11	1	1	0	1
1012	101	$\bar{1}1$	22	$\bar{1}\bar{1}$	1	1	0	1
1012	101	$\bar{1}\bar{1}$	00	$1\bar{1}$	0	1	1	1
1012	101	$\bar{1}\bar{1}$	00	$\bar{1}1$	0	1	1	1
1012	101	$\bar{1}\bar{1}$	02	11	0	0	0	1
1012	101	$\bar{1}\bar{1}$	02	$\bar{1}\bar{1}$	0	0	0	1
1012	101	$\bar{1}\bar{1}$	20	11	0	0	0	1
1012	101	$\bar{1}\bar{1}$	20	$\bar{1}\bar{1}$	0	0	0	1
1012	101	$\bar{1}\bar{1}$	22	$1\bar{1}$	0	1	1	1

Continued on next page

Supplementary Table 7 – continued

SI	Invariants							
1012	101	$\bar{1}\bar{1}$	22	$\bar{1}1$	0	1	1	1
1100	110	00	11	11	0	1	1	0
1100	110	00	11	$\bar{1}\bar{1}$	0	1	1	0
1100	110	00	$1\bar{1}$	$1\bar{1}$	0	0	0	0
1100	110	00	$1\bar{1}$	$\bar{1}1$	0	0	0	0
1100	110	00	$\bar{1}1$	$1\bar{1}$	0	0	0	0
1100	110	00	$\bar{1}1$	$\bar{1}1$	0	0	0	0
1100	110	00	$\bar{1}\bar{1}$	11	0	1	1	0
1100	110	00	$\bar{1}\bar{1}$	$\bar{1}\bar{1}$	0	1	1	0
1100	110	02	11	$1\bar{1}$	1	1	0	0
1100	110	02	11	$\bar{1}1$	1	1	0	0
1100	110	02	$1\bar{1}$	11	1	0	1	0
1100	110	02	$1\bar{1}$	$\bar{1}\bar{1}$	1	0	1	0
1100	110	02	$\bar{1}1$	11	1	0	1	0
1100	110	02	$\bar{1}1$	$\bar{1}\bar{1}$	1	0	1	0
1100	110	02	$\bar{1}\bar{1}$	$1\bar{1}$	1	1	0	0
1100	110	02	$\bar{1}\bar{1}$	$\bar{1}1$	1	1	0	0
1100	110	20	11	$1\bar{1}$	1	1	0	0
1100	110	20	11	$\bar{1}1$	1	1	0	0
1100	110	20	$1\bar{1}$	11	1	0	1	0
1100	110	20	$1\bar{1}$	$\bar{1}\bar{1}$	1	0	1	0
1100	110	20	$\bar{1}1$	11	1	0	1	0
1100	110	20	$\bar{1}1$	$\bar{1}\bar{1}$	1	0	1	0
1100	110	20	$\bar{1}\bar{1}$	$1\bar{1}$	1	1	0	0
1100	110	20	$\bar{1}\bar{1}$	$\bar{1}1$	1	1	0	0
1100	110	22	11	11	0	1	1	0
1100	110	22	11	$\bar{1}\bar{1}$	0	1	1	0
1100	110	22	$1\bar{1}$	$1\bar{1}$	0	0	0	0
1100	110	22	$1\bar{1}$	$\bar{1}1$	0	0	0	0
1100	110	22	$\bar{1}1$	$1\bar{1}$	0	0	0	0
1100	110	22	$\bar{1}1$	$\bar{1}1$	0	0	0	0
1100	110	22	$\bar{1}\bar{1}$	11	0	1	1	0
1100	110	22	$\bar{1}\bar{1}$	$\bar{1}\bar{1}$	0	1	1	0
1102	110	00	11	$1\bar{1}$	1	0	1	1
1102	110	00	11	$\bar{1}1$	1	0	1	1
1102	110	00	$1\bar{1}$	11	1	1	0	1
1102	110	00	$1\bar{1}$	$\bar{1}\bar{1}$	1	1	0	1
1102	110	00	$\bar{1}1$	11	1	1	0	1
1102	110	00	$\bar{1}1$	$\bar{1}\bar{1}$	1	1	0	1
1102	110	00	$\bar{1}\bar{1}$	$1\bar{1}$	1	0	1	1
1102	110	00	$\bar{1}\bar{1}$	$\bar{1}1$	1	0	1	1
1102	110	02	11	11	0	0	0	1
1102	110	02	11	$\bar{1}\bar{1}$	0	0	0	1
1102	110	02	$1\bar{1}$	$1\bar{1}$	0	1	1	1
1102	110	02	$1\bar{1}$	$\bar{1}1$	0	1	1	1
1102	110	02	$\bar{1}1$	$1\bar{1}$	0	1	1	1
1102	110	02	$\bar{1}1$	$\bar{1}1$	0	1	1	1
1102	110	02	$\bar{1}\bar{1}$	11	0	0	0	1
1102	110	02	$\bar{1}\bar{1}$	$\bar{1}\bar{1}$	0	0	0	1
1102	110	20	11	11	0	0	0	1
1102	110	20	11	$\bar{1}\bar{1}$	0	0	0	1
1102	110	20	$1\bar{1}$	$1\bar{1}$	0	1	1	1
1102	110	20	$1\bar{1}$	$\bar{1}1$	0	1	1	1
1102	110	20	$\bar{1}1$	$1\bar{1}$	0	1	1	1
1102	110	20	$\bar{1}1$	$\bar{1}1$	0	1	1	1
1102	110	20	$\bar{1}\bar{1}$	11	0	0	0	1
1102	110	20	$\bar{1}\bar{1}$	$\bar{1}\bar{1}$	0	0	0	1
1102	110	22	11	$1\bar{1}$	1	0	1	1
1102	110	22	11	$\bar{1}1$	1	0	1	1
1102	110	22	$1\bar{1}$	11	1	1	0	1
1102	110	22	$1\bar{1}$	$\bar{1}\bar{1}$	1	1	0	1

Continued on next page

Supplementary Table 7 – continued

SI	Invariants							
1102	110	22	$\bar{1}1$	11	1	1	0	1
1102	110	22	$\bar{1}1$	$\bar{1}\bar{1}$	1	1	0	1
1102	110	22	$\bar{1}\bar{1}$	$1\bar{1}$	1	0	1	1
1102	110	22	$\bar{1}\bar{1}$	$\bar{1}1$	1	0	1	1
1110	111	11	11	$1\bar{1}$	1	1	0	0
1110	111	11	11	$\bar{1}1$	1	1	0	0
1110	111	11	$1\bar{1}$	11	1	0	1	0
1110	111	11	$1\bar{1}$	$\bar{1}\bar{1}$	1	0	1	0
1110	111	11	$\bar{1}1$	11	1	0	1	0
1110	111	11	$\bar{1}1$	$\bar{1}\bar{1}$	1	0	1	0
1110	111	11	$\bar{1}\bar{1}$	$1\bar{1}$	1	1	0	0
1110	111	11	$\bar{1}\bar{1}$	$\bar{1}1$	1	1	0	0
1110	111	$1\bar{1}$	11	11	0	1	1	0
1110	111	$1\bar{1}$	11	$\bar{1}\bar{1}$	0	1	1	0
1110	111	$1\bar{1}$	$1\bar{1}$	$1\bar{1}$	0	0	0	0
1110	111	$1\bar{1}$	$1\bar{1}$	$\bar{1}1$	0	0	0	0
1110	111	$1\bar{1}$	$\bar{1}1$	$1\bar{1}$	0	0	0	0
1110	111	$1\bar{1}$	$\bar{1}1$	$\bar{1}1$	0	0	0	0
1110	111	$1\bar{1}$	$\bar{1}\bar{1}$	11	0	1	1	0
1110	111	$1\bar{1}$	$\bar{1}\bar{1}$	$\bar{1}\bar{1}$	0	1	1	0
1110	111	$\bar{1}1$	11	11	0	1	1	0
1110	111	$\bar{1}1$	11	$\bar{1}\bar{1}$	0	1	1	0
1110	111	$\bar{1}1$	$1\bar{1}$	$1\bar{1}$	0	0	0	0
1110	111	$\bar{1}1$	$1\bar{1}$	$\bar{1}1$	0	0	0	0
1110	111	$\bar{1}1$	$\bar{1}1$	$1\bar{1}$	0	0	0	0
1110	111	$\bar{1}1$	$\bar{1}1$	$\bar{1}1$	0	0	0	0
1110	111	$\bar{1}1$	$\bar{1}\bar{1}$	11	0	1	1	0
1110	111	$\bar{1}1$	$\bar{1}\bar{1}$	$\bar{1}\bar{1}$	0	1	1	0
1110	111	$\bar{1}\bar{1}$	11	$1\bar{1}$	1	1	0	0
1110	111	$\bar{1}\bar{1}$	11	$\bar{1}1$	1	1	0	0
1110	111	$\bar{1}\bar{1}$	$1\bar{1}$	11	1	0	1	0
1110	111	$\bar{1}\bar{1}$	$1\bar{1}$	$\bar{1}\bar{1}$	1	0	1	0
1110	111	$\bar{1}\bar{1}$	$\bar{1}1$	11	1	0	1	0
1110	111	$\bar{1}\bar{1}$	$\bar{1}1$	$\bar{1}\bar{1}$	1	0	1	0
1110	111	$\bar{1}\bar{1}$	$\bar{1}\bar{1}$	$1\bar{1}$	1	1	0	0
1110	111	$\bar{1}\bar{1}$	$\bar{1}\bar{1}$	$\bar{1}1$	1	1	0	0
1112	111	11	11	11	0	0	0	1
1112	111	11	11	$\bar{1}\bar{1}$	0	0	0	1
1112	111	11	$1\bar{1}$	$1\bar{1}$	0	1	1	1
1112	111	11	$1\bar{1}$	$\bar{1}1$	0	1	1	1
1112	111	11	$\bar{1}1$	$1\bar{1}$	0	1	1	1
1112	111	11	$\bar{1}1$	$\bar{1}1$	0	1	1	1
1112	111	11	$\bar{1}\bar{1}$	11	0	0	0	1
1112	111	11	$\bar{1}\bar{1}$	$\bar{1}\bar{1}$	0	0	0	1
1112	111	$1\bar{1}$	11	$1\bar{1}$	1	0	1	1
1112	111	$1\bar{1}$	11	$\bar{1}1$	1	0	1	1
1112	111	$1\bar{1}$	$1\bar{1}$	11	1	1	0	1
1112	111	$1\bar{1}$	$1\bar{1}$	$\bar{1}\bar{1}$	1	1	0	1
1112	111	$1\bar{1}$	$\bar{1}1$	11	1	1	0	1
1112	111	$1\bar{1}$	$\bar{1}1$	$\bar{1}\bar{1}$	1	1	0	1
1112	111	$1\bar{1}$	$\bar{1}\bar{1}$	$1\bar{1}$	1	0	1	1
1112	111	$1\bar{1}$	$\bar{1}\bar{1}$	$\bar{1}1$	1	0	1	1
1112	111	$\bar{1}1$	11	$1\bar{1}$	1	0	1	1
1112	111	$\bar{1}1$	11	$\bar{1}1$	1	0	1	1
1112	111	$\bar{1}1$	$1\bar{1}$	11	1	1	0	1
1112	111	$\bar{1}1$	$1\bar{1}$	$\bar{1}\bar{1}$	1	1	0	1
1112	111	$\bar{1}1$	$\bar{1}1$	11	1	1	0	1
1112	111	$\bar{1}1$	$\bar{1}1$	$\bar{1}\bar{1}$	1	1	0	1
1112	111	$\bar{1}1$	$\bar{1}\bar{1}$	$1\bar{1}$	1	0	1	1
1112	111	$\bar{1}1$	$\bar{1}\bar{1}$	$\bar{1}1$	1	0	1	1
1112	111	$\bar{1}\bar{1}$	11	11	0	0	0	1

Continued on next page

Supplementary Table 7 – continued

**SI**	**Invariants**							
1112	111	$\bar{1}\bar{1}$	11	$\bar{1}\bar{1}$	0	0	0	1
1112	111	$\bar{1}\bar{1}$	$1\bar{1}$	$1\bar{1}$	0	1	1	1
1112	111	$\bar{1}\bar{1}$	$1\bar{1}$	$\bar{1}1$	0	1	1	1
1112	111	$\bar{1}\bar{1}$	$\bar{1}1$	$1\bar{1}$	0	1	1	1
1112	111	$\bar{1}\bar{1}$	$\bar{1}1$	$\bar{1}1$	0	1	1	1
1112	111	$\bar{1}\bar{1}$	$\bar{1}\bar{1}$	11	0	0	0	1
1112	111	$\bar{1}\bar{1}$	$\bar{1}\bar{1}$	$\bar{1}\bar{1}$	0	0	0	1

Space group #48 : $Pnnn$

$\mathbb{Z}_{2,2,2,4}$	weak	$g^{001}_{\frac{1}{2}\frac{1}{2}0}$	$g^{010}_{\frac{1}{2}0\frac{1}{2}}$	$g^{100}_{0\frac{1}{2}\frac{1}{2}}$	2^{001}	2^{010}	2^{100}	i
0000	000	0	0	0	0	0	0	0
0000	000	0	𝟙	𝟙	0	𝟙	𝟙	0
0000	000	𝟙	0	𝟙	𝟙	0	𝟙	0
0000	000	𝟙	𝟙	0	𝟙	𝟙	0	0
0002	000	0	0	𝟙	𝟙	𝟙	0	𝟙
0002	000	0	𝟙	0	𝟙	0	𝟙	𝟙
0002	000	𝟙	0	0	0	𝟙	𝟙	𝟙
0002	000	𝟙	𝟙	𝟙	0	0	0	𝟙

Space group #49 : $Pccm$

$\mathbb{Z}_{2,2,2,4}$	weak	$m^{001}_{(2)}$	$g^{010}_{00\frac{1}{2}}$	$g^{100}_{00\frac{1}{2}}$	2^{001}	2^{010}	2^{100}	i
0000	000	00	0	0	0	0	0	0
0000	000	00	𝟙	𝟙	0	𝟙	𝟙	0
0000	000	20	0	𝟙	𝟙	0	𝟙	0
0000	000	20	𝟙	0	𝟙	𝟙	0	0
0002	000	00	0	𝟙	𝟙	𝟙	0	𝟙
0002	000	00	𝟙	0	𝟙	0	𝟙	𝟙
0002	000	20	0	0	0	𝟙	𝟙	𝟙
0002	000	20	𝟙	𝟙	0	0	0	𝟙
0100	010	00	0	0	0	0	0	0
0100	010	00	1	𝟙	0	𝟙	1	0
0100	010	20	0	𝟙	1	0	1	0
0100	010	20	1	0	1	𝟙	0	0
0102	010	00	0	𝟙	1	𝟙	0	1
0102	010	00	1	0	1	0	1	1
0102	010	20	0	0	0	𝟙	1	1
0102	010	20	1	𝟙	0	0	0	1
1000	100	00	0	0	0	0	0	0
1000	100	00	𝟙	1	0	1	𝟙	0
1000	100	20	0	1	1	0	𝟙	0
1000	100	20	𝟙	0	1	1	0	0
1002	100	00	0	1	1	1	0	1
1002	100	00	𝟙	0	1	0	𝟙	1
1002	100	20	0	0	0	1	𝟙	1
1002	100	20	𝟙	1	0	0	0	1
1100	110	00	0	0	0	0	0	0
1100	110	00	1	1	0	1	1	0
1100	110	20	0	1	1	0	1	0
1100	110	20	1	0	1	1	0	0
1102	110	00	0	1	1	1	0	1
1102	110	00	1	0	1	0	1	1
1102	110	20	0	0	0	1	1	1
1102	110	20	1	1	0	0	0	1

Space group #50 : $Pban$

$\mathbb{Z}_{2,2,2,4}$	weak	$g^{001}_{\frac{1}{2}\frac{1}{2}0}$	$g^{010}_{\frac{1}{2}00}$	$g^{100}_{0\frac{1}{2}0}$	2^{001}	2^{010}	2^{100}	i
0000	000	0	0	0	0	0	0	0
0000	000	0	𝟙	𝟙	0	𝟙	𝟙	0
0000	000	𝟙	0	𝟙	𝟙	0	𝟙	0
0000	000	𝟙	𝟙	0	𝟙	𝟙	0	0
0002	000	0	0	𝟙	𝟙	𝟙	0	𝟙
0002	000	0	𝟙	0	𝟙	0	𝟙	𝟙

Continued on next page

Supplementary Table 7 – continued

**SI**	**Invariants**							
0002	000	$\mathbb{1}$	0	0	0	$\mathbb{1}$	$\mathbb{1}$	$\mathbb{1}$
0002	000	$\mathbb{1}$	$\mathbb{1}$	$\mathbb{1}$	0	0	0	$\mathbb{1}$
0010	001	0	0	0	0	0	0	0
0010	001	0	$\mathbb{1}$	$\mathbb{1}$	0	1	1	0
0010	001	1	0	$\mathbb{1}$	$\mathbb{1}$	0	1	0
0010	001	1	$\mathbb{1}$	0	$\mathbb{1}$	1	0	0
0012	001	0	0	$\mathbb{1}$	$\mathbb{1}$	1	0	1
0012	001	0	$\mathbb{1}$	0	$\mathbb{1}$	0	1	1
0012	001	1	0	0	0	1	1	1
0012	001	1	$\mathbb{1}$	$\mathbb{1}$	0	0	0	1
Space group #51 : $Pmma$								
$\mathbb{Z}_{2,2,2,4}$	weak	$m^{010}_{(2)}$	$m^{100}_{(2)}$	$g^{001}_{\frac{1}{2}00}$	2^{001}	2^{010}	i	2^{100}_{1}
0000	000	00	00	0	0	0	0	0
0000	000	00	20	$\mathbb{1}$	$\mathbb{1}$	0	0	$\mathbb{1}$
0000	000	02	00	$\mathbb{1}$	$\mathbb{1}$	$\mathbb{1}$	0	0
0000	000	02	20	0	0	$\mathbb{1}$	0	$\mathbb{1}$
0000	000	20	00	$\mathbb{1}$	$\mathbb{1}$	$\mathbb{1}$	0	0
0000	000	20	20	0	0	$\mathbb{1}$	0	$\mathbb{1}$
0000	000	22	00	0	0	0	0	0
0000	000	22	20	$\mathbb{1}$	$\mathbb{1}$	0	0	$\mathbb{1}$
0002	000	00	00	$\mathbb{1}$	0	$\mathbb{1}$	$\mathbb{1}$	$\mathbb{1}$
0002	000	00	20	0	$\mathbb{1}$	$\mathbb{1}$	$\mathbb{1}$	0
0002	000	02	00	0	$\mathbb{1}$	0	$\mathbb{1}$	$\mathbb{1}$
0002	000	02	20	$\mathbb{1}$	0	0	$\mathbb{1}$	0
0002	000	20	00	0	$\mathbb{1}$	0	$\mathbb{1}$	$\mathbb{1}$
0002	000	20	20	$\mathbb{1}$	0	0	$\mathbb{1}$	0
0002	000	22	00	$\mathbb{1}$	0	$\mathbb{1}$	$\mathbb{1}$	$\mathbb{1}$
0002	000	22	20	0	$\mathbb{1}$	$\mathbb{1}$	$\mathbb{1}$	0
0010	001	00	00	0	0	0	0	0
0010	001	00	20	1	$\mathbb{1}$	0	0	1
0010	001	02	00	1	$\mathbb{1}$	1	0	0
0010	001	02	20	0	0	1	0	1
0010	001	20	00	1	$\mathbb{1}$	1	0	0
0010	001	20	20	0	0	1	0	1
0010	001	22	00	0	0	0	0	0
0010	001	22	20	1	$\mathbb{1}$	0	0	1
0012	001	00	00	1	0	1	1	1
0012	001	00	20	0	$\mathbb{1}$	1	1	0
0012	001	02	00	0	$\mathbb{1}$	0	1	1
0012	001	02	20	1	0	0	1	0
0012	001	20	00	0	$\mathbb{1}$	0	1	1
0012	001	20	20	1	0	0	1	0
0012	001	22	00	1	0	1	1	1
0012	001	22	20	0	$\mathbb{1}$	1	1	0
0100	010	11	00	$\mathbb{1}$	1	$\mathbb{1}$	0	0
0100	010	11	20	0	0	$\mathbb{1}$	0	1
0100	010	$1\bar{1}$	00	0	0	0	0	0
0100	010	$1\bar{1}$	20	$\mathbb{1}$	1	0	0	1
0100	010	$\bar{1}1$	00	0	0	0	0	0
0100	010	$\bar{1}1$	20	$\mathbb{1}$	1	0	0	1
0100	010	$\bar{1}\bar{1}$	00	$\mathbb{1}$	1	$\mathbb{1}$	0	0
0100	010	$\bar{1}\bar{1}$	20	0	0	$\mathbb{1}$	0	1
0102	010	11	00	0	1	0	1	1
0102	010	11	20	$\mathbb{1}$	0	0	1	0
0102	010	$1\bar{1}$	00	$\mathbb{1}$	0	$\mathbb{1}$	1	1
0102	010	$1\bar{1}$	20	0	1	$\mathbb{1}$	1	0
0102	010	$\bar{1}1$	00	$\mathbb{1}$	0	$\mathbb{1}$	1	1
0102	010	$\bar{1}1$	20	0	1	$\mathbb{1}$	1	0
0102	010	$\bar{1}\bar{1}$	00	0	1	0	1	1
0102	010	$\bar{1}\bar{1}$	20	$\mathbb{1}$	0	0	1	0

Continued on next page

Supplementary Table 7 – continued

**SI**	**Invariants**							
0110	011	11	00	1	1	1	0	0
0110	011	11	20	0	0	1	0	1
0110	011	$1\bar{1}$	00	0	0	0	0	0
0110	011	$1\bar{1}$	20	1	1	0	0	1
0110	011	$\bar{1}1$	00	0	0	0	0	0
0110	011	$\bar{1}1$	20	1	1	0	0	1
0110	011	$\bar{1}\bar{1}$	00	1	1	1	0	0
0110	011	$\bar{1}\bar{1}$	20	0	0	1	0	1
0112	011	11	00	0	1	0	1	1
0112	011	11	20	1	0	0	1	0
0112	011	$1\bar{1}$	00	1	0	1	1	1
0112	011	$1\bar{1}$	20	0	1	1	1	0
0112	011	$\bar{1}1$	00	1	0	1	1	1
0112	011	$\bar{1}1$	20	0	1	1	1	0
0112	011	$\bar{1}\bar{1}$	00	0	1	0	1	1
0112	011	$\bar{1}\bar{1}$	20	1	0	0	1	0
Space group #52 : *Pnna*								
$\mathbb{Z}_{2,2,2,4}$	weak	$g^{001}_{\frac{1}{2}00}$	$g^{010}_{\frac{1}{2}0\frac{1}{2}}$	$g^{100}_{0\frac{1}{2}\frac{1}{2}}$	2^{001}	2^{100}	i	2^{010}_1
0000	000	0	0	0	0	0	0	0
0000	000	0	$\mathbb{1}$	$\mathbb{1}$	0	$\mathbb{1}$	0	$\mathbb{1}$
0000	000	$\mathbb{1}$	0	$\mathbb{1}$	$\mathbb{1}$	$\mathbb{1}$	0	0
0000	000	$\mathbb{1}$	$\mathbb{1}$	0	$\mathbb{1}$	0	0	$\mathbb{1}$
0002	000	0	0	$\mathbb{1}$	$\mathbb{1}$	0	$\mathbb{1}$	$\mathbb{1}$
0002	000	0	$\mathbb{1}$	0	$\mathbb{1}$	$\mathbb{1}$	$\mathbb{1}$	0
0002	000	$\mathbb{1}$	0	0	0	$\mathbb{1}$	$\mathbb{1}$	$\mathbb{1}$
0002	000	$\mathbb{1}$	$\mathbb{1}$	$\mathbb{1}$	0	0	$\mathbb{1}$	0
Space group #53 : *Pmna*								
$\mathbb{Z}_{2,2,2,4}$	weak	$m^{100}_{(2)}$	$g^{001}_{\frac{1}{2}00}$	$g^{010}_{\frac{1}{2}0\frac{1}{2}}$	2^{010}	2^{100}	i	2^{001}_1
0000	000	00	0	0	0	0	0	0
0000	000	00	$\mathbb{1}$	$\mathbb{1}$	$\mathbb{1}$	0	0	$\mathbb{1}$
0000	000	20	0	$\mathbb{1}$	$\mathbb{1}$	$\mathbb{1}$	0	0
0000	000	20	$\mathbb{1}$	0	0	$\mathbb{1}$	0	$\mathbb{1}$
0002	000	00	0	$\mathbb{1}$	0	$\mathbb{1}$	$\mathbb{1}$	$\mathbb{1}$
0002	000	00	$\mathbb{1}$	0	$\mathbb{1}$	$\mathbb{1}$	$\mathbb{1}$	0
0002	000	20	0	0	$\mathbb{1}$	0	$\mathbb{1}$	$\mathbb{1}$
0002	000	20	$\mathbb{1}$	$\mathbb{1}$	0	0	$\mathbb{1}$	0
0100	010	00	0	0	0	0	0	0
0100	010	00	$\mathbb{1}$	1	$\mathbb{1}$	0	0	1
0100	010	20	0	1	$\mathbb{1}$	1	0	0
0100	010	20	$\mathbb{1}$	0	0	1	0	1
0102	010	00	0	1	0	1	1	1
0102	010	00	$\mathbb{1}$	0	$\mathbb{1}$	1	1	0
0102	010	20	0	0	$\mathbb{1}$	0	1	1
0102	010	20	$\mathbb{1}$	1	0	0	1	0
Space group #54 : *Pcca*								
$\mathbb{Z}_{2,2,2,4}$	weak	$g^{001}_{\frac{1}{2}00}$	$g^{010}_{00\frac{1}{2}}$	$g^{100}_{00\frac{1}{2}}$	2^{001}	2^{010}	i	2^{100}_1
0000	000	0	0	0	0	0	0	0
0000	000	0	$\mathbb{1}$	$\mathbb{1}$	0	$\mathbb{1}$	0	$\mathbb{1}$
0000	000	$\mathbb{1}$	0	$\mathbb{1}$	$\mathbb{1}$	0	0	$\mathbb{1}$
0000	000	$\mathbb{1}$	$\mathbb{1}$	0	$\mathbb{1}$	$\mathbb{1}$	0	0
0002	000	0	0	$\mathbb{1}$	$\mathbb{1}$	$\mathbb{1}$	$\mathbb{1}$	0
0002	000	0	$\mathbb{1}$	0	$\mathbb{1}$	0	$\mathbb{1}$	$\mathbb{1}$
0002	000	$\mathbb{1}$	0	0	0	$\mathbb{1}$	$\mathbb{1}$	$\mathbb{1}$
0002	000	$\mathbb{1}$	$\mathbb{1}$	$\mathbb{1}$	0	0	$\mathbb{1}$	0
0100	010	0	0	0	0	0	0	0
0100	010	0	1	$\mathbb{1}$	0	$\mathbb{1}$	0	1
0100	010	$\mathbb{1}$	0	$\mathbb{1}$	1	0	0	1
0100	010	$\mathbb{1}$	1	0	1	$\mathbb{1}$	0	0
0102	010	0	0	$\mathbb{1}$	1	$\mathbb{1}$	1	0

Continued on next page

Supplementary Table 7 – continued

SI	Invariants							
0102	010	0	1	0	1	0	1	1
0102	010	$\mathbb{1}$	0	0	0	$\mathbb{1}$	1	1
0102	010	$\mathbb{1}$	1	$\mathbb{1}$	0	0	1	0

Space group #55 : *Pbam*

$\mathbb{Z}_{2,2,2,4}$	weak	$m^{001}_{(2)}$	$g^{010}_{\frac{1}{2}00}$	$g^{100}_{0\frac{1}{2}0}$	2^{001}	i	2^{010}_1	2^{100}_1
0000	000	00	0	0	0	0	0	0
0000	000	00	$\mathbb{1}$	$\mathbb{1}$	0	0	$\mathbb{1}$	$\mathbb{1}$
0000	000	02	0	$\mathbb{1}$	$\mathbb{1}$	0	0	$\mathbb{1}$
0000	000	02	$\mathbb{1}$	0	$\mathbb{1}$	0	$\mathbb{1}$	0
0000	000	20	0	$\mathbb{1}$	$\mathbb{1}$	0	0	$\mathbb{1}$
0000	000	20	$\mathbb{1}$	0	$\mathbb{1}$	0	$\mathbb{1}$	0
0000	000	22	0	0	0	0	0	0
0000	000	22	$\mathbb{1}$	$\mathbb{1}$	0	0	$\mathbb{1}$	$\mathbb{1}$
0002	000	00	0	$\mathbb{1}$	$\mathbb{1}$	$\mathbb{1}$	$\mathbb{1}$	0
0002	000	00	$\mathbb{1}$	0	$\mathbb{1}$	$\mathbb{1}$	0	$\mathbb{1}$
0002	000	02	0	0	0	$\mathbb{1}$	$\mathbb{1}$	$\mathbb{1}$
0002	000	02	$\mathbb{1}$	$\mathbb{1}$	0	$\mathbb{1}$	0	0
0002	000	20	0	0	0	$\mathbb{1}$	$\mathbb{1}$	$\mathbb{1}$
0002	000	20	$\mathbb{1}$	$\mathbb{1}$	0	$\mathbb{1}$	0	0
0002	000	22	0	$\mathbb{1}$	$\mathbb{1}$	$\mathbb{1}$	$\mathbb{1}$	0
0002	000	22	$\mathbb{1}$	0	$\mathbb{1}$	$\mathbb{1}$	0	$\mathbb{1}$
0010	001	11	0	$\mathbb{1}$	$\mathbb{1}$	0	0	1
0010	001	11	$\mathbb{1}$	0	$\mathbb{1}$	0	1	0
0010	001	$1\bar{1}$	0	0	0	0	0	0
0010	001	$1\bar{1}$	$\mathbb{1}$	$\mathbb{1}$	0	0	1	1
0010	001	$\bar{1}1$	0	0	0	0	0	0
0010	001	$\bar{1}1$	$\mathbb{1}$	$\mathbb{1}$	0	0	1	1
0010	001	$\bar{1}\bar{1}$	0	$\mathbb{1}$	$\mathbb{1}$	0	0	1
0010	001	$\bar{1}\bar{1}$	$\mathbb{1}$	0	$\mathbb{1}$	0	1	0
0012	001	11	0	0	0	1	1	1
0012	001	11	$\mathbb{1}$	$\mathbb{1}$	0	1	0	0
0012	001	$1\bar{1}$	0	$\mathbb{1}$	$\mathbb{1}$	1	1	0
0012	001	$1\bar{1}$	$\mathbb{1}$	0	$\mathbb{1}$	1	0	1
0012	001	$\bar{1}1$	0	$\mathbb{1}$	$\mathbb{1}$	1	1	0
0012	001	$\bar{1}1$	$\mathbb{1}$	0	$\mathbb{1}$	1	0	1
0012	001	$\bar{1}\bar{1}$	0	0	0	1	1	1
0012	001	$\bar{1}\bar{1}$	$\mathbb{1}$	$\mathbb{1}$	0	1	0	0

Space group #56 : *Pccn*

$\mathbb{Z}_{2,2,2,4}$	weak	$g^{001}_{\frac{1}{2}\frac{1}{2}0}$	$g^{010}_{00\frac{1}{2}}$	$g^{100}_{00\frac{1}{2}}$	2^{001}	i	2^{010}_1	2^{100}_1
0000	000	0	0	0	0	0	0	0
0000	000	0	$\mathbb{1}$	$\mathbb{1}$	0	0	$\mathbb{1}$	$\mathbb{1}$
0000	000	$\mathbb{1}$	0	$\mathbb{1}$	$\mathbb{1}$	0	0	$\mathbb{1}$
0000	000	$\mathbb{1}$	$\mathbb{1}$	0	$\mathbb{1}$	0	$\mathbb{1}$	0
0002	000	0	0	$\mathbb{1}$	$\mathbb{1}$	$\mathbb{1}$	$\mathbb{1}$	0
0002	000	0	$\mathbb{1}$	0	$\mathbb{1}$	$\mathbb{1}$	0	$\mathbb{1}$
0002	000	$\mathbb{1}$	0	0	0	$\mathbb{1}$	$\mathbb{1}$	$\mathbb{1}$
0002	000	$\mathbb{1}$	$\mathbb{1}$	$\mathbb{1}$	0	$\mathbb{1}$	0	0

Space group #57 : *Pbcm*

$\mathbb{Z}_{2,2,2,4}$	weak	$m^{001}_{(2)}$	$g^{010}_{00\frac{1}{2}}$	$g^{100}_{0\frac{1}{2}0}$	2^{100}	i	2^{001}_1	2^{010}_1
0000	000	00	0	0	0	0	0	0
0000	000	00	$\mathbb{1}$	$\mathbb{1}$	$\mathbb{1}$	0	0	$\mathbb{1}$
0000	000	20	0	$\mathbb{1}$	$\mathbb{1}$	0	$\mathbb{1}$	0
0000	000	20	$\mathbb{1}$	0	0	0	$\mathbb{1}$	$\mathbb{1}$
0002	000	00	0	$\mathbb{1}$	0	$\mathbb{1}$	$\mathbb{1}$	$\mathbb{1}$
0002	000	00	$\mathbb{1}$	0	$\mathbb{1}$	$\mathbb{1}$	$\mathbb{1}$	0
0002	000	20	0	0	$\mathbb{1}$	$\mathbb{1}$	0	$\mathbb{1}$
0002	000	20	$\mathbb{1}$	$\mathbb{1}$	0	$\mathbb{1}$	0	0
1000	100	00	0	0	0	0	0	0
1000	100	00	$\mathbb{1}$	1	$\mathbb{1}$	0	0	1

Continued on next page

Supplementary Table 7 – continued

SI	Invariants							
1000	100	20	0	1	$\mathbb{1}$	0	1	0
1000	100	20	$\mathbb{1}$	0	0	0	1	1
1002	100	00	0	1	0	1	1	1
1002	100	00	$\mathbb{1}$	0	$\mathbb{1}$	1	1	0
1002	100	20	0	0	$\mathbb{1}$	1	0	1
1002	100	20	$\mathbb{1}$	1	0	1	0	0
Space group #58 : *Pnnm*								
$\mathbb{Z}_{2,2,2,4}$	weak	$m^{001}_{(2)}$	$g^{010}_{\frac{1}{2}0\frac{1}{2}}$	$g^{100}_{0\frac{1}{2}\frac{1}{2}}$	2^{001}	i	2^{010}_1	2^{100}_1
0000	000	00	0	0	0	0	0	0
0000	000	00	$\mathbb{1}$	$\mathbb{1}$	0	0	$\mathbb{1}$	$\mathbb{1}$
0000	000	20	0	$\mathbb{1}$	$\mathbb{1}$	0	0	$\mathbb{1}$
0000	000	20	$\mathbb{1}$	0	$\mathbb{1}$	0	$\mathbb{1}$	0
0002	000	00	0	$\mathbb{1}$	$\mathbb{1}$	$\mathbb{1}$	$\mathbb{1}$	0
0002	000	00	$\mathbb{1}$	0	$\mathbb{1}$	$\mathbb{1}$	0	$\mathbb{1}$
0002	000	20	0	0	0	$\mathbb{1}$	$\mathbb{1}$	$\mathbb{1}$
0002	000	20	$\mathbb{1}$	$\mathbb{1}$	0	$\mathbb{1}$	0	0
Space group #59 : *Pmmn*								
$\mathbb{Z}_{2,2,2,4}$	weak	$m^{010}_{(2)}$	$m^{100}_{(2)}$	$g^{001}_{\frac{1}{2}\frac{1}{2}0}$	2^{001}	i	2^{010}_1	2^{100}_1
0000	000	00	00	0	0	0	0	0
0000	000	00	20	$\mathbb{1}$	$\mathbb{1}$	0	0	$\mathbb{1}$
0000	000	20	00	$\mathbb{1}$	$\mathbb{1}$	0	$\mathbb{1}$	0
0000	000	20	20	0	0	0	$\mathbb{1}$	$\mathbb{1}$
0002	000	00	00	$\mathbb{1}$	0	$\mathbb{1}$	$\mathbb{1}$	$\mathbb{1}$
0002	000	00	20	0	$\mathbb{1}$	$\mathbb{1}$	$\mathbb{1}$	0
0002	000	20	00	0	$\mathbb{1}$	$\mathbb{1}$	0	$\mathbb{1}$
0002	000	20	20	$\mathbb{1}$	0	$\mathbb{1}$	0	0
0010	001	00	00	0	0	0	0	0
0010	001	00	20	1	$\mathbb{1}$	0	0	1
0010	001	20	00	1	$\mathbb{1}$	0	1	0
0010	001	20	20	0	0	0	1	1
0012	001	00	00	1	0	1	1	1
0012	001	00	20	0	$\mathbb{1}$	1	1	0
0012	001	20	00	0	$\mathbb{1}$	1	0	1
0012	001	20	20	1	0	1	0	0
Space group #60 : *Pbcn*								
$\mathbb{Z}_{2,2,2,4}$	weak	$g^{001}_{\frac{1}{2}\frac{1}{2}0}$	$g^{010}_{00\frac{1}{2}}$	$g^{100}_{0\frac{1}{2}0}$	2^{010}	i	2^{001}_1	2^{100}_1
0000	000	0	0	0	0	0	0	0
0000	000	0	$\mathbb{1}$	$\mathbb{1}$	$\mathbb{1}$	0	0	$\mathbb{1}$
0000	000	$\mathbb{1}$	0	$\mathbb{1}$	0	0	$\mathbb{1}$	$\mathbb{1}$
0000	000	$\mathbb{1}$	$\mathbb{1}$	0	$\mathbb{1}$	0	$\mathbb{1}$	0
0002	000	0	0	$\mathbb{1}$	$\mathbb{1}$	$\mathbb{1}$	$\mathbb{1}$	0
0002	000	0	$\mathbb{1}$	0	0	$\mathbb{1}$	$\mathbb{1}$	$\mathbb{1}$
0002	000	$\mathbb{1}$	0	0	$\mathbb{1}$	$\mathbb{1}$	0	$\mathbb{1}$
0002	000	$\mathbb{1}$	$\mathbb{1}$	$\mathbb{1}$	0	$\mathbb{1}$	0	0
Space group #61 : *Pbca*								
$\mathbb{Z}_{2,2,2,4}$	weak	$g^{001}_{\frac{1}{2}00}$	$g^{010}_{00\frac{1}{2}}$	$g^{100}_{0\frac{1}{2}0}$	i	2^{001}_1	2^{010}_1	2^{100}_1
0000	000	0	0	0	0	0	0	0
0000	000	0	$\mathbb{1}$	$\mathbb{1}$	0	0	$\mathbb{1}$	$\mathbb{1}$
0000	000	$\mathbb{1}$	0	$\mathbb{1}$	0	$\mathbb{1}$	0	$\mathbb{1}$
0000	000	$\mathbb{1}$	$\mathbb{1}$	0	0	$\mathbb{1}$	$\mathbb{1}$	0
0002	000	0	0	$\mathbb{1}$	$\mathbb{1}$	$\mathbb{1}$	$\mathbb{1}$	0
0002	000	0	$\mathbb{1}$	0	$\mathbb{1}$	$\mathbb{1}$	0	$\mathbb{1}$
0002	000	$\mathbb{1}$	0	0	$\mathbb{1}$	0	$\mathbb{1}$	$\mathbb{1}$
0002	000	$\mathbb{1}$	$\mathbb{1}$	$\mathbb{1}$	$\mathbb{1}$	0	0	0
Space group #62 : *Pnma*								
$\mathbb{Z}_{2,2,2,4}$	weak	$m^{010}_{(2)}$	$g^{001}_{\frac{1}{2}00}$	$g^{100}_{0\frac{1}{2}\frac{1}{2}}$	i	2^{001}_1	2^{010}_1	2^{100}_1
0000	000	00	0	0	0	0	0	0

Continued on next page

Supplementary Table 7 – continued

SI	Invariants							
0000	000	00	$\mathbb{1}$	$\mathbb{1}$	0	$\mathbb{1}$	0	$\mathbb{1}$
0000	000	20	0	$\mathbb{1}$	0	0	$\mathbb{1}$	$\mathbb{1}$
0000	000	20	$\mathbb{1}$	0	0	$\mathbb{1}$	$\mathbb{1}$	0
0002	000	00	0	$\mathbb{1}$	$\mathbb{1}$	$\mathbb{1}$	$\mathbb{1}$	0
0002	000	00	$\mathbb{1}$	0	$\mathbb{1}$	0	$\mathbb{1}$	$\mathbb{1}$
0002	000	20	0	0	$\mathbb{1}$	$\mathbb{1}$	0	$\mathbb{1}$
0002	000	20	$\mathbb{1}$	$\mathbb{1}$	$\mathbb{1}$	0	0	0

Space group #63 : *Cmcm*

$\mathbb{Z}_{2,2,2,4}$	weak	$m^{001}_{(2)}$	$m^{100}_{(2)}$	$g^{010}_{00\frac{1}{2}}$	$g^{010}_{\frac{1}{2}0\frac{1}{2}}$	$g^{100}_{0\frac{1}{2}0}$	2^{010}	2^{100}	i	2^{001}_1	2^{010}_1	2^{100}_1
0000	000	00	0	0	0	0	0	0	0	0	0	0
0000	000	00	2	$\mathbb{1}$	$\mathbb{1}$	$\mathbb{1}$	$\mathbb{1}$	$\mathbb{1}$	0	0	$\mathbb{1}$	$\mathbb{1}$
0000	000	20	0	$\mathbb{1}$	$\mathbb{1}$	0	$\mathbb{1}$	0	0	$\mathbb{1}$	$\mathbb{1}$	0
0000	000	20	2	0	0	$\mathbb{1}$	0	$\mathbb{1}$	0	$\mathbb{1}$	0	$\mathbb{1}$
0002	000	00	0	$\mathbb{1}$	$\mathbb{1}$	0	0	$\mathbb{1}$	$\mathbb{1}$	$\mathbb{1}$	0	$\mathbb{1}$
0002	000	00	2	0	0	$\mathbb{1}$	$\mathbb{1}$	0	$\mathbb{1}$	$\mathbb{1}$	$\mathbb{1}$	0
0002	000	20	0	0	0	0	$\mathbb{1}$	$\mathbb{1}$	$\mathbb{1}$	0	$\mathbb{1}$	$\mathbb{1}$
0002	000	20	2	$\mathbb{1}$	$\mathbb{1}$	$\mathbb{1}$	0	0	$\mathbb{1}$	0	0	0
1100	110	00	0	0	$\mathbb{1}$	$\mathbb{1}$	0	0	0	0	$\mathbb{1}$	$\mathbb{1}$
1100	110	00	2	$\mathbb{1}$	0	0	$\mathbb{1}$	$\mathbb{1}$	0	0	0	0
1100	110	20	0	$\mathbb{1}$	0	$\mathbb{1}$	$\mathbb{1}$	0	0	1	0	$\mathbb{1}$
1100	110	20	2	0	$\mathbb{1}$	0	0	$\mathbb{1}$	0	1	$\mathbb{1}$	0
1102	110	00	0	$\mathbb{1}$	0	$\mathbb{1}$	0	$\mathbb{1}$	1	1	$\mathbb{1}$	0
1102	110	00	2	0	$\mathbb{1}$	0	$\mathbb{1}$	0	1	1	0	$\mathbb{1}$
1102	110	20	0	0	$\mathbb{1}$	$\mathbb{1}$	$\mathbb{1}$	$\mathbb{1}$	1	0	0	0
1102	110	20	2	$\mathbb{1}$	0	0	0	0	1	0	$\mathbb{1}$	$\mathbb{1}$

Space group #64 : *Cmce*

$\mathbb{Z}_{2,2,2,4}$	weak	$m^{100}_{(2)}$	$g^{001}_{0\frac{1}{2}0}$	$g^{010}_{00\frac{1}{2}}$	$g^{010}_{\frac{1}{2}0\frac{1}{2}}$	$g^{100}_{0\frac{1}{2}0}$	2^{010}	2^{100}	i	2^{001}_1	2^{010}_1	2^{100}_1
0000	000	0	0	0	0	0	0	0	0	0	0	0
0000	000	0	$\mathbb{1}$	$\mathbb{1}$	$\mathbb{1}$	0	$\mathbb{1}$	0	0	$\mathbb{1}$	$\mathbb{1}$	0
0000	000	2	0	$\mathbb{1}$	$\mathbb{1}$	$\mathbb{1}$	$\mathbb{1}$	$\mathbb{1}$	0	0	$\mathbb{1}$	$\mathbb{1}$
0000	000	2	$\mathbb{1}$	0	0	$\mathbb{1}$	0	$\mathbb{1}$	0	$\mathbb{1}$	0	$\mathbb{1}$
0002	000	0	0	$\mathbb{1}$	$\mathbb{1}$	0	0	$\mathbb{1}$	$\mathbb{1}$	$\mathbb{1}$	0	$\mathbb{1}$
0002	000	0	$\mathbb{1}$	0	0	0	$\mathbb{1}$	$\mathbb{1}$	$\mathbb{1}$	0	$\mathbb{1}$	$\mathbb{1}$
0002	000	2	0	0	0	$\mathbb{1}$	$\mathbb{1}$	0	$\mathbb{1}$	$\mathbb{1}$	$\mathbb{1}$	0
0002	000	2	$\mathbb{1}$	$\mathbb{1}$	$\mathbb{1}$	$\mathbb{1}$	0	0	$\mathbb{1}$	0	0	0
1100	110	0	0	0	$\mathbb{1}$	$\mathbb{1}$	$\mathbb{1}$	0	0	0	0	$\mathbb{1}$
1100	110	0	$\mathbb{1}$	$\mathbb{1}$	0	$\mathbb{1}$	0	0	0	1	$\mathbb{1}$	$\mathbb{1}$
1100	110	2	0	$\mathbb{1}$	0	0	0	$\mathbb{1}$	0	0	$\mathbb{1}$	0
1100	110	2	$\mathbb{1}$	0	$\mathbb{1}$	0	$\mathbb{1}$	$\mathbb{1}$	0	1	0	0
1102	110	0	0	$\mathbb{1}$	0	$\mathbb{1}$	$\mathbb{1}$	$\mathbb{1}$	1	1	0	0
1102	110	0	$\mathbb{1}$	0	$\mathbb{1}$	$\mathbb{1}$	0	$\mathbb{1}$	1	0	$\mathbb{1}$	0
1102	110	2	0	0	$\mathbb{1}$	0	0	0	1	1	$\mathbb{1}$	$\mathbb{1}$
1102	110	2	$\mathbb{1}$	$\mathbb{1}$	0	0	$\mathbb{1}$	0	1	0	0	$\mathbb{1}$

Space group #65 : *Cmmm*

$\mathbb{Z}_{2,2,2,4}$	weak	$m^{001}_{(2)}$	$m^{010}_{(2)}$	$m^{100}_{(2)}$	$g^{010}_{\frac{1}{2}00}$	$g^{100}_{0\frac{1}{2}0}$	2^{001}	2^{010}	2^{100}	i	2^{010}_1	2^{100}_1
0000	000	00	0	0	0	0	0	0	0	0	0	0
0000	000	00	2	2	$\mathbb{1}$	$\mathbb{1}$	0	$\mathbb{1}$	$\mathbb{1}$	0	$\mathbb{1}$	$\mathbb{1}$
0000	000	02	0	2	0	$\mathbb{1}$	$\mathbb{1}$	0	$\mathbb{1}$	0	0	$\mathbb{1}$
0000	000	02	2	0	$\mathbb{1}$	0	$\mathbb{1}$	$\mathbb{1}$	0	0	$\mathbb{1}$	0
0000	000	20	0	2	0	$\mathbb{1}$	$\mathbb{1}$	0	$\mathbb{1}$	0	0	$\mathbb{1}$
0000	000	20	2	0	$\mathbb{1}$	0	$\mathbb{1}$	$\mathbb{1}$	0	0	$\mathbb{1}$	0
0000	000	22	0	0	0	0	0	0	0	0	0	0
0000	000	22	2	2	$\mathbb{1}$	$\mathbb{1}$	0	$\mathbb{1}$	$\mathbb{1}$	0	$\mathbb{1}$	$\mathbb{1}$
0002	000	00	0	2	0	$\mathbb{1}$	$\mathbb{1}$	$\mathbb{1}$	0	$\mathbb{1}$	$\mathbb{1}$	0
0002	000	00	2	0	$\mathbb{1}$	0	$\mathbb{1}$	0	$\mathbb{1}$	$\mathbb{1}$	0	$\mathbb{1}$
0002	000	02	0	0	0	0	0	$\mathbb{1}$	$\mathbb{1}$	$\mathbb{1}$	$\mathbb{1}$	$\mathbb{1}$
0002	000	02	2	2	$\mathbb{1}$	$\mathbb{1}$	0	0	0	$\mathbb{1}$	0	0
0002	000	20	0	0	0	0	0	$\mathbb{1}$	$\mathbb{1}$	$\mathbb{1}$	$\mathbb{1}$	$\mathbb{1}$
0002	000	20	2	2	$\mathbb{1}$	$\mathbb{1}$	0	0	0	$\mathbb{1}$	0	0

Continued on next page

Supplementary Table 7 – continued

SI	Invariants											
0002	000	22	0	2	0	$\mathbb{1}$	$\mathbb{1}$	$\mathbb{1}$	0	$\mathbb{1}$	$\mathbb{1}$	0
0002	000	22	2	0	$\mathbb{1}$	0	$\mathbb{1}$	0	$\mathbb{1}$	$\mathbb{1}$	0	$\mathbb{1}$
0010	001	11	0	2	0	$\mathbb{1}$	$\mathbb{1}$	0	1	0	0	1
0010	001	11	2	0	$\mathbb{1}$	0	$\mathbb{1}$	1	0	0	1	0
0010	001	$1\bar{1}$	0	0	0	0	0	0	0	0	0	0
0010	001	$1\bar{1}$	2	2	$\mathbb{1}$	$\mathbb{1}$	0	1	1	0	1	1
0010	001	$\bar{1}1$	0	0	0	0	0	0	0	0	0	0
0010	001	$\bar{1}1$	2	2	$\mathbb{1}$	$\mathbb{1}$	0	1	1	0	1	1
0010	001	$\bar{1}\bar{1}$	0	2	0	$\mathbb{1}$	$\mathbb{1}$	0	1	0	0	1
0010	001	$\bar{1}\bar{1}$	2	0	$\mathbb{1}$	0	$\mathbb{1}$	1	0	0	1	0
0012	001	11	0	0	0	0	0	1	1	1	1	1
0012	001	11	2	2	$\mathbb{1}$	$\mathbb{1}$	0	0	0	1	0	0
0012	001	$1\bar{1}$	0	2	0	$\mathbb{1}$	$\mathbb{1}$	1	0	1	1	0
0012	001	$1\bar{1}$	2	0	$\mathbb{1}$	0	$\mathbb{1}$	0	1	1	0	1
0012	001	$\bar{1}1$	0	2	0	$\mathbb{1}$	$\mathbb{1}$	1	0	1	1	0
0012	001	$\bar{1}1$	2	0	$\mathbb{1}$	0	$\mathbb{1}$	0	1	1	0	1
0012	001	$\bar{1}\bar{1}$	0	0	0	0	0	1	1	1	1	1
0012	001	$\bar{1}\bar{1}$	2	2	$\mathbb{1}$	$\mathbb{1}$	0	0	0	1	0	0
1100	110	00	0	0	$\mathbb{1}$	$\mathbb{1}$	0	0	0	0	$\mathbb{1}$	$\mathbb{1}$
1100	110	00	2	2	0	0	0	$\mathbb{1}$	$\mathbb{1}$	0	0	0
1100	110	02	0	2	$\mathbb{1}$	0	1	0	$\mathbb{1}$	0	$\mathbb{1}$	0
1100	110	02	2	0	0	$\mathbb{1}$	1	$\mathbb{1}$	0	0	0	$\mathbb{1}$
1100	110	20	0	2	$\mathbb{1}$	0	1	0	$\mathbb{1}$	0	$\mathbb{1}$	0
1100	110	20	2	0	0	$\mathbb{1}$	1	$\mathbb{1}$	0	0	0	$\mathbb{1}$
1100	110	22	0	0	$\mathbb{1}$	$\mathbb{1}$	0	0	0	0	$\mathbb{1}$	$\mathbb{1}$
1100	110	22	2	2	0	0	0	$\mathbb{1}$	$\mathbb{1}$	0	0	0
1102	110	00	0	2	$\mathbb{1}$	0	1	$\mathbb{1}$	0	1	0	$\mathbb{1}$
1102	110	00	2	0	0	$\mathbb{1}$	1	0	$\mathbb{1}$	1	$\mathbb{1}$	0
1102	110	02	0	0	$\mathbb{1}$	$\mathbb{1}$	0	$\mathbb{1}$	$\mathbb{1}$	1	0	0
1102	110	02	2	2	0	0	0	0	0	1	$\mathbb{1}$	$\mathbb{1}$
1102	110	20	0	0	$\mathbb{1}$	$\mathbb{1}$	0	$\mathbb{1}$	$\mathbb{1}$	1	0	0
1102	110	20	2	2	0	0	0	0	0	1	$\mathbb{1}$	$\mathbb{1}$
1102	110	22	0	2	$\mathbb{1}$	0	1	$\mathbb{1}$	0	1	0	$\mathbb{1}$
1102	110	22	2	0	0	$\mathbb{1}$	1	0	$\mathbb{1}$	1	$\mathbb{1}$	0
1110	111	11	0	2	$\mathbb{1}$	0	1	0	1	0	1	0
1110	111	11	2	0	0	$\mathbb{1}$	1	1	0	0	0	1
1110	111	$1\bar{1}$	0	0	$\mathbb{1}$	$\mathbb{1}$	0	0	0	0	1	1
1110	111	$1\bar{1}$	2	2	0	0	0	1	1	0	0	0
1110	111	$\bar{1}1$	0	0	$\mathbb{1}$	$\mathbb{1}$	0	0	0	0	1	1
1110	111	$\bar{1}1$	2	2	0	0	0	1	1	0	0	0
1110	111	$\bar{1}\bar{1}$	0	2	$\mathbb{1}$	0	1	0	1	0	1	0
1110	111	$\bar{1}\bar{1}$	2	0	0	$\mathbb{1}$	1	1	0	0	0	1
1112	111	11	0	0	$\mathbb{1}$	$\mathbb{1}$	0	1	1	1	0	0
1112	111	11	2	2	0	0	0	0	0	1	1	1
1112	111	$1\bar{1}$	0	2	$\mathbb{1}$	0	1	1	0	1	0	1
1112	111	$1\bar{1}$	2	0	0	$\mathbb{1}$	1	0	1	1	1	0
1112	111	$\bar{1}1$	0	2	$\mathbb{1}$	0	1	1	0	1	0	1
1112	111	$\bar{1}1$	2	0	0	$\mathbb{1}$	1	0	1	1	1	0
1112	111	$\bar{1}\bar{1}$	0	0	$\mathbb{1}$	$\mathbb{1}$	0	1	1	1	0	0
1112	111	$\bar{1}\bar{1}$	2	2	0	0	0	0	0	1	1	1

Space group #66 : *Cccm*

$\mathbb{Z}_{2,2,2,4}$	weak	$m^{001}_{(2)}$	$g^{010}_{00\frac{1}{2}}$	$g^{010}_{\frac{1}{2}0\frac{1}{2}}$	$g^{100}_{00\frac{1}{2}}$	$g^{100}_{0\frac{1}{2}\frac{1}{2}}$	2^{001}	2^{010}	2^{100}	i	2^{010}_{1}	2^{100}_{1}
0000	000	00	0	0	0	0	0	0	0	0	0	0
0000	000	00	$\mathbb{1}$	$\mathbb{1}$	$\mathbb{1}$	$\mathbb{1}$	0	$\mathbb{1}$	$\mathbb{1}$	0	$\mathbb{1}$	$\mathbb{1}$
0000	000	20	0	0	$\mathbb{1}$	$\mathbb{1}$	$\mathbb{1}$	0	$\mathbb{1}$	0	0	$\mathbb{1}$
0000	000	20	$\mathbb{1}$	$\mathbb{1}$	0	0	$\mathbb{1}$	$\mathbb{1}$	0	0	$\mathbb{1}$	0
0002	000	00	0	0	$\mathbb{1}$	$\mathbb{1}$	$\mathbb{1}$	$\mathbb{1}$	0	$\mathbb{1}$	$\mathbb{1}$	0
0002	000	00	$\mathbb{1}$	$\mathbb{1}$	0	0	$\mathbb{1}$	0	$\mathbb{1}$	$\mathbb{1}$	0	$\mathbb{1}$
0002	000	20	0	0	0	0	0	$\mathbb{1}$	$\mathbb{1}$	$\mathbb{1}$	$\mathbb{1}$	$\mathbb{1}$
0002	000	20	$\mathbb{1}$	$\mathbb{1}$	$\mathbb{1}$	$\mathbb{1}$	0	0	0	$\mathbb{1}$	0	0

Continued on next page

Supplementary Table 7 – continued

SI	Invariants											
1100	110	00	0	$\mathbb{1}$	0	$\mathbb{1}$	0	0	0	0	$\mathbb{1}$	$\mathbb{1}$
1100	110	00	$\mathbb{1}$	0	$\mathbb{1}$	0	0	$\mathbb{1}$	$\mathbb{1}$	0	0	0
1100	110	20	0	$\mathbb{1}$	$\mathbb{1}$	0	1	0	$\mathbb{1}$	0	$\mathbb{1}$	0
1100	110	20	$\mathbb{1}$	0	0	$\mathbb{1}$	1	$\mathbb{1}$	0	0	0	$\mathbb{1}$
1102	110	00	0	$\mathbb{1}$	$\mathbb{1}$	0	1	$\mathbb{1}$	0	1	0	$\mathbb{1}$
1102	110	00	$\mathbb{1}$	0	0	$\mathbb{1}$	1	0	$\mathbb{1}$	1	$\mathbb{1}$	0
1102	110	20	0	$\mathbb{1}$	0	$\mathbb{1}$	0	$\mathbb{1}$	$\mathbb{1}$	1	0	0
1102	110	20	$\mathbb{1}$	0	$\mathbb{1}$	0	0	0	0	1	$\mathbb{1}$	$\mathbb{1}$

Space group #67 : *Cmme*

$\mathbb{Z}_{2,2,2,4}$	weak	$m^{010}_{(2)}$	$m^{100}_{(2)}$	$g^{001}_{0\frac{1}{2}0}$	$g^{010}_{\frac{1}{2}00}$	$g^{100}_{0\frac{1}{2}0}$	2^{001}	2^{010}	2^{100}	i	2^{010}_1	2^{100}_1
0000	000	0	0	0	0	0	0	0	0	0	0	0
0000	000	0	2	$\mathbb{1}$	0	$\mathbb{1}$	$\mathbb{1}$	0	$\mathbb{1}$	0	0	$\mathbb{1}$
0000	000	2	0	$\mathbb{1}$	$\mathbb{1}$	0	$\mathbb{1}$	$\mathbb{1}$	0	0	$\mathbb{1}$	0
0000	000	2	2	0	$\mathbb{1}$	$\mathbb{1}$	0	$\mathbb{1}$	$\mathbb{1}$	0	$\mathbb{1}$	$\mathbb{1}$
0002	000	0	0	$\mathbb{1}$	0	0	0	$\mathbb{1}$	$\mathbb{1}$	$\mathbb{1}$	$\mathbb{1}$	$\mathbb{1}$
0002	000	0	2	0	0	$\mathbb{1}$	$\mathbb{1}$	$\mathbb{1}$	0	$\mathbb{1}$	$\mathbb{1}$	0
0002	000	2	0	0	$\mathbb{1}$	0	$\mathbb{1}$	0	$\mathbb{1}$	$\mathbb{1}$	0	$\mathbb{1}$
0002	000	2	2	$\mathbb{1}$	$\mathbb{1}$	$\mathbb{1}$	0	0	0	$\mathbb{1}$	0	0
0010	001	0	0	0	0	0	0	0	0	0	0	0
0010	001	0	2	1	0	$\mathbb{1}$	$\mathbb{1}$	0	1	0	0	1
0010	001	2	0	1	$\mathbb{1}$	0	$\mathbb{1}$	1	0	0	1	0
0010	001	2	2	0	$\mathbb{1}$	$\mathbb{1}$	0	1	1	0	1	1
0012	001	0	0	1	0	0	0	1	1	1	1	1
0012	001	0	2	0	0	$\mathbb{1}$	$\mathbb{1}$	1	0	1	1	0
0012	001	2	0	0	$\mathbb{1}$	0	$\mathbb{1}$	0	1	1	0	1
0012	001	2	2	1	$\mathbb{1}$	$\mathbb{1}$	0	0	0	1	0	0
1100	110	0	0	0	$\mathbb{1}$	$\mathbb{1}$	0	$\mathbb{1}$	0	0	0	$\mathbb{1}$
1100	110	0	2	$\mathbb{1}$	$\mathbb{1}$	0	1	$\mathbb{1}$	$\mathbb{1}$	0	0	0
1100	110	2	0	$\mathbb{1}$	0	$\mathbb{1}$	1	0	0	0	$\mathbb{1}$	$\mathbb{1}$
1100	110	2	2	0	0	0	0	0	$\mathbb{1}$	0	$\mathbb{1}$	0
1102	110	0	0	$\mathbb{1}$	$\mathbb{1}$	$\mathbb{1}$	0	0	$\mathbb{1}$	1	$\mathbb{1}$	0
1102	110	0	2	0	$\mathbb{1}$	0	1	0	0	1	$\mathbb{1}$	$\mathbb{1}$
1102	110	2	0	0	0	$\mathbb{1}$	1	$\mathbb{1}$	$\mathbb{1}$	1	0	0
1102	110	2	2	$\mathbb{1}$	0	0	0	$\mathbb{1}$	0	1	0	$\mathbb{1}$
1110	111	0	0	0	$\mathbb{1}$	$\mathbb{1}$	0	1	0	0	0	1
1110	111	0	2	1	$\mathbb{1}$	0	1	1	1	0	0	0
1110	111	2	0	1	0	$\mathbb{1}$	1	0	0	0	1	1
1110	111	2	2	0	0	0	0	0	1	0	1	0
1112	111	0	0	1	$\mathbb{1}$	$\mathbb{1}$	0	0	1	1	1	0
1112	111	0	2	0	$\mathbb{1}$	0	1	0	0	1	1	1
1112	111	2	0	0	0	$\mathbb{1}$	1	1	1	1	0	0
1112	111	2	2	1	0	0	0	1	0	1	0	1

Space group #68 : *Ccce*

$\mathbb{Z}_{2,2,2,4}$	weak	$g^{001}_{0\frac{1}{2}0}$	$g^{010}_{00\frac{1}{2}}$	$g^{010}_{\frac{1}{2}0\frac{1}{2}}$	$g^{100}_{00\frac{1}{2}}$	$g^{100}_{0\frac{1}{2}\frac{1}{2}}$	2^{001}	2^{010}	2^{100}	i	2^{010}_1	2^{100}_1
0000	000	0	0	0	0	0	0	0	0	0	0	0
0000	000	0	$\mathbb{1}$	$\mathbb{1}$	$\mathbb{1}$	$\mathbb{1}$	0	$\mathbb{1}$	$\mathbb{1}$	0	$\mathbb{1}$	$\mathbb{1}$
0000	000	$\mathbb{1}$	0	0	$\mathbb{1}$	$\mathbb{1}$	$\mathbb{1}$	0	$\mathbb{1}$	0	0	$\mathbb{1}$
0000	000	$\mathbb{1}$	$\mathbb{1}$	$\mathbb{1}$	0	0	$\mathbb{1}$	$\mathbb{1}$	0	0	$\mathbb{1}$	0
0002	000	0	0	0	$\mathbb{1}$	$\mathbb{1}$	$\mathbb{1}$	$\mathbb{1}$	0	$\mathbb{1}$	$\mathbb{1}$	0
0002	000	0	$\mathbb{1}$	$\mathbb{1}$	0	0	$\mathbb{1}$	0	$\mathbb{1}$	$\mathbb{1}$	0	$\mathbb{1}$
0002	000	$\mathbb{1}$	0	0	0	0	0	$\mathbb{1}$	$\mathbb{1}$	$\mathbb{1}$	$\mathbb{1}$	$\mathbb{1}$
0002	000	$\mathbb{1}$	$\mathbb{1}$	$\mathbb{1}$	$\mathbb{1}$	$\mathbb{1}$	0	0	0	$\mathbb{1}$	0	0
1100	110	0	0	$\mathbb{1}$	$\mathbb{1}$	0	0	0	0	0	$\mathbb{1}$	$\mathbb{1}$
1100	110	0	$\mathbb{1}$	0	0	$\mathbb{1}$	0	$\mathbb{1}$	$\mathbb{1}$	0	0	0
1100	110	$\mathbb{1}$	0	$\mathbb{1}$	0	$\mathbb{1}$	1	0	$\mathbb{1}$	0	$\mathbb{1}$	0
1100	110	$\mathbb{1}$	$\mathbb{1}$	0	$\mathbb{1}$	0	1	$\mathbb{1}$	0	0	0	$\mathbb{1}$
1102	110	0	0	$\mathbb{1}$	0	$\mathbb{1}$	1	$\mathbb{1}$	0	1	0	$\mathbb{1}$
1102	110	0	$\mathbb{1}$	0	$\mathbb{1}$	0	1	0	$\mathbb{1}$	1	$\mathbb{1}$	0
1102	110	$\mathbb{1}$	0	$\mathbb{1}$	$\mathbb{1}$	0	0	$\mathbb{1}$	$\mathbb{1}$	1	0	0

Continued on next page

Supplementary Table 7 – continued

SI	Invariants												
1102	110	$\mathbb{1}$	$\mathbb{1}$	0	0	$\mathbb{1}$	0	0	0	1	$\mathbb{1}$	$\mathbb{1}$	

Space group #69 : $Fmmm$

$\mathbb{Z}_{2,2,2,4}$	weak	$m^{001}_{(2)}$	$m^{010}_{(2)}$	$m^{100}_{(2)}$	$g^{001}_{\frac{1}{2}00}$	$g^{010}_{\frac{1}{2}00}$	$g^{100}_{0\frac{1}{2}0}$	2^{001}	2^{010}	2^{100}	i	2^{001}_1	2^{010}_1	2^{100}_1
0000	000	0	0	0	0	0	0	0	0	0	0	0	0	0
0000	000	0	2	2	0	$\mathbb{1}$	$\mathbb{1}$	0	$\mathbb{1}$	$\mathbb{1}$	0	0	$\mathbb{1}$	$\mathbb{1}$
0000	000	2	0	2	$\mathbb{1}$	0	$\mathbb{1}$	$\mathbb{1}$	0	$\mathbb{1}$	0	$\mathbb{1}$	0	$\mathbb{1}$
0000	000	2	2	0	$\mathbb{1}$	$\mathbb{1}$	0	$\mathbb{1}$	$\mathbb{1}$	0	0	$\mathbb{1}$	$\mathbb{1}$	0
0002	000	0	0	2	0	0	$\mathbb{1}$	$\mathbb{1}$	$\mathbb{1}$	0	$\mathbb{1}$	$\mathbb{1}$	$\mathbb{1}$	0
0002	000	0	2	0	0	$\mathbb{1}$	0	$\mathbb{1}$	0	$\mathbb{1}$	$\mathbb{1}$	$\mathbb{1}$	0	$\mathbb{1}$
0002	000	2	0	0	$\mathbb{1}$	0	0	0	$\mathbb{1}$	$\mathbb{1}$	$\mathbb{1}$	0	$\mathbb{1}$	$\mathbb{1}$
0002	000	2	2	2	$\mathbb{1}$	$\mathbb{1}$	$\mathbb{1}$	0	0	0	$\mathbb{1}$	0	0	0
0110	011	0	0	0	$\mathbb{1}$	$\mathbb{1}$	$\mathbb{1}$	0	0	0	0	0	0	$\mathbb{1}$
0110	011	0	2	2	$\mathbb{1}$	0	0	0	1	$\mathbb{1}$	0	0	1	0
0110	011	2	0	2	0	$\mathbb{1}$	0	1	0	$\mathbb{1}$	0	1	0	0
0110	011	2	2	0	0	0	$\mathbb{1}$	1	1	0	0	1	1	$\mathbb{1}$
0112	011	0	0	2	$\mathbb{1}$	$\mathbb{1}$	0	1	1	0	1	1	1	$\mathbb{1}$
0112	011	0	2	0	$\mathbb{1}$	0	$\mathbb{1}$	1	0	$\mathbb{1}$	1	1	0	0
0112	011	2	0	0	0	$\mathbb{1}$	$\mathbb{1}$	0	1	$\mathbb{1}$	1	0	1	0
0112	011	2	2	2	0	0	0	0	0	0	1	0	0	$\mathbb{1}$
1010	101	0	0	0	0	$\mathbb{1}$	$\mathbb{1}$	0	0	0	0	1	$\mathbb{1}$	0
1010	101	0	2	2	0	0	0	0	$\mathbb{1}$	1	0	1	0	1
1010	101	2	0	2	$\mathbb{1}$	$\mathbb{1}$	0	1	0	1	0	0	$\mathbb{1}$	1
1010	101	2	2	0	$\mathbb{1}$	0	$\mathbb{1}$	1	$\mathbb{1}$	0	0	0	0	0
1012	101	0	0	2	0	$\mathbb{1}$	0	1	$\mathbb{1}$	0	1	0	0	0
1012	101	0	2	0	0	0	$\mathbb{1}$	1	0	1	1	0	$\mathbb{1}$	1
1012	101	2	0	0	$\mathbb{1}$	$\mathbb{1}$	$\mathbb{1}$	0	$\mathbb{1}$	1	1	1	0	1
1012	101	2	2	2	$\mathbb{1}$	0	0	0	0	0	1	1	$\mathbb{1}$	0
1100	110	0	0	0	$\mathbb{1}$	0	0	0	0	0	0	$\mathbb{1}$	1	1
1100	110	0	2	2	$\mathbb{1}$	$\mathbb{1}$	$\mathbb{1}$	0	1	1	0	$\mathbb{1}$	0	0
1100	110	2	0	2	0	0	$\mathbb{1}$	$\mathbb{1}$	0	1	0	0	1	0
1100	110	2	2	0	0	$\mathbb{1}$	0	$\mathbb{1}$	1	0	0	0	0	1
1102	110	0	0	2	$\mathbb{1}$	0	$\mathbb{1}$	$\mathbb{1}$	1	0	1	0	0	1
1102	110	0	2	0	$\mathbb{1}$	$\mathbb{1}$	0	$\mathbb{1}$	0	1	1	0	1	0
1102	110	2	0	0	0	0	0	0	1	1	1	$\mathbb{1}$	0	0
1102	110	2	2	2	0	$\mathbb{1}$	$\mathbb{1}$	0	0	0	1	$\mathbb{1}$	1	1

Space group #70 : $Fddd$

$\mathbb{Z}_{2,2,2,4}$	weak	$g^{001}_{\frac{1}{4}\frac{1}{4}0}$	$g^{001}_{\frac{1}{4}\bar{\frac{1}{4}}0}$	$g^{010}_{\frac{1}{4}0\bar{\frac{1}{4}}}$	$g^{010}_{\frac{1}{4}0\frac{1}{4}}$	$g^{100}_{0\frac{1}{4}\frac{1}{4}}$	$g^{100}_{0\frac{1}{4}\bar{\frac{1}{4}}}$	2^{001}	2^{010}	2^{100}	i	2^{001}_1	2^{010}_1	2^{100}_1
0000	000	0	0	0	0	0	0	0	0	0	0	0	0	0
0000	000	0	0	$\mathbb{1}$	$\mathbb{1}$	$\mathbb{1}$	$\mathbb{1}$	0	$\mathbb{1}$	$\mathbb{1}$	0	0	$\mathbb{1}$	$\mathbb{1}$
0000	000	$\mathbb{1}$	$\mathbb{1}$	0	0	$\mathbb{1}$	$\mathbb{1}$	$\mathbb{1}$	0	$\mathbb{1}$	0	$\mathbb{1}$	0	$\mathbb{1}$
0000	000	$\mathbb{1}$	$\mathbb{1}$	$\mathbb{1}$	$\mathbb{1}$	0	0	$\mathbb{1}$	$\mathbb{1}$	0	0	$\mathbb{1}$	$\mathbb{1}$	0
0002	000	0	0	0	0	$\mathbb{1}$	$\mathbb{1}$	$\mathbb{1}$	$\mathbb{1}$	0	$\mathbb{1}$	$\mathbb{1}$	$\mathbb{1}$	0
0002	000	0	0	$\mathbb{1}$	$\mathbb{1}$	0	0	$\mathbb{1}$	0	$\mathbb{1}$	$\mathbb{1}$	$\mathbb{1}$	0	$\mathbb{1}$
0002	000	$\mathbb{1}$	$\mathbb{1}$	0	0	0	0	0	$\mathbb{1}$	$\mathbb{1}$	$\mathbb{1}$	0	$\mathbb{1}$	$\mathbb{1}$
0002	000	$\mathbb{1}$	$\mathbb{1}$	$\mathbb{1}$	$\mathbb{1}$	$\mathbb{1}$	$\mathbb{1}$	0	0	0	$\mathbb{1}$	0	0	0

Space group #71 : $Immm$

$\mathbb{Z}_{2,2,2,4}$	weak	$m^{001}_{(2)}$	$m^{010}_{(2)}$	$m^{100}_{(2)}$	$g^{001}_{\frac{1}{2}\frac{1}{2}0}$	$g^{010}_{\frac{1}{2}0\frac{1}{2}}$	$g^{100}_{0\frac{1}{2}\frac{1}{2}}$	2^{001}	2^{010}	2^{100}	i	2^{001}_1	2^{010}_1	2^{100}_1
0000	000	0	0	0	0	0	0	0	0	0	0	0	0	0
0000	000	0	2	2	0	$\mathbb{1}$	$\mathbb{1}$	0	$\mathbb{1}$	$\mathbb{1}$	0	0	$\mathbb{1}$	$\mathbb{1}$
0000	000	2	0	2	$\mathbb{1}$	0	$\mathbb{1}$	$\mathbb{1}$	0	$\mathbb{1}$	0	$\mathbb{1}$	0	$\mathbb{1}$
0000	000	2	2	0	$\mathbb{1}$	$\mathbb{1}$	0	$\mathbb{1}$	$\mathbb{1}$	0	0	$\mathbb{1}$	$\mathbb{1}$	0
0002	000	0	0	2	0	0	$\mathbb{1}$	$\mathbb{1}$	$\mathbb{1}$	0	$\mathbb{1}$	$\mathbb{1}$	$\mathbb{1}$	0
0002	000	0	2	0	0	$\mathbb{1}$	0	$\mathbb{1}$	0	$\mathbb{1}$	$\mathbb{1}$	$\mathbb{1}$	0	$\mathbb{1}$
0002	000	2	0	0	$\mathbb{1}$	0	0	0	$\mathbb{1}$	$\mathbb{1}$	$\mathbb{1}$	0	$\mathbb{1}$	$\mathbb{1}$
0002	000	2	2	2	$\mathbb{1}$	$\mathbb{1}$	$\mathbb{1}$	0	0	0	$\mathbb{1}$	0	0	0
1110	111	0	0	0	$\mathbb{1}$	$\mathbb{1}$	$\mathbb{1}$	0	0	0	0	$\mathbb{1}$	$\mathbb{1}$	$\mathbb{1}$
1110	111	0	2	2	$\mathbb{1}$	0	0	0	$\mathbb{1}$	$\mathbb{1}$	0	$\mathbb{1}$	0	0
1110	111	2	0	2	0	$\mathbb{1}$	0	$\mathbb{1}$	0	$\mathbb{1}$	0	0	$\mathbb{1}$	0
1110	111	2	2	0	0	0	$\mathbb{1}$	$\mathbb{1}$	$\mathbb{1}$	0	0	0	0	$\mathbb{1}$

Continued on next page

Supplementary Table 7 – continued

SI	Invariants													
1112	111	0	0	2	𝟙	𝟙	0	𝟙	𝟙	0	1	0	0	𝟙
1112	111	0	2	0	𝟙	0	𝟙	𝟙	0	𝟙	1	0	𝟙	0
1112	111	2	0	0	0	𝟙	𝟙	0	𝟙	𝟙	1	𝟙	0	0
1112	111	2	2	2	0	0	0	0	0	0	1	𝟙	𝟙	𝟙

Space group #72 : *Ibam*

$\mathbb{Z}_{2,2,2,4}$	weak	$m^{001}_{(2)}$	$g^{001}_{\frac{1}{2}\frac{1}{2}0}$	$g^{010}_{00\frac{1}{2}}$	$g^{010}_{\frac{1}{2}00}$	$g^{100}_{00\frac{1}{2}}$	$g^{100}_{0\frac{1}{2}0}$	2^{001}	2^{010}	2^{100}	i	2^{001}_1	2^{010}_1	2^{100}_1
0000	000	0	0	0	0	0	0	0	0	0	0	0	0	0
0000	000	0	0	𝟙	𝟙	𝟙	𝟙	0	𝟙	𝟙	0	0	𝟙	𝟙
0000	000	2	𝟙	0	0	𝟙	𝟙	𝟙	0	𝟙	0	𝟙	0	𝟙
0000	000	2	𝟙	𝟙	𝟙	0	0	𝟙	𝟙	0	0	𝟙	𝟙	0
0002	000	0	0	0	0	𝟙	𝟙	𝟙	𝟙	0	𝟙	𝟙	𝟙	0
0002	000	0	0	𝟙	𝟙	0	0	𝟙	0	𝟙	𝟙	𝟙	0	𝟙
0002	000	2	𝟙	0	0	0	0	0	𝟙	𝟙	𝟙	0	𝟙	𝟙
0002	000	2	𝟙	𝟙	𝟙	𝟙	𝟙	0	0	0	𝟙	0	0	0
1110	111	0	𝟙	0	𝟙	0	𝟙	0	0	0	0	𝟙	𝟙	𝟙
1110	111	0	𝟙	𝟙	0	𝟙	0	0	𝟙	𝟙	0	𝟙	0	0
1110	111	2	0	0	𝟙	𝟙	0	𝟙	0	𝟙	0	0	𝟙	0
1110	111	2	0	𝟙	0	0	𝟙	𝟙	𝟙	0	0	0	0	𝟙
1112	111	0	𝟙	0	𝟙	𝟙	0	𝟙	𝟙	0	1	0	0	𝟙
1112	111	0	𝟙	𝟙	0	0	𝟙	𝟙	0	𝟙	1	0	𝟙	0
1112	111	2	0	0	𝟙	0	𝟙	0	𝟙	𝟙	1	𝟙	0	0
1112	111	2	0	𝟙	0	𝟙	0	0	0	0	1	𝟙	𝟙	𝟙

Space group #73 : *Ibca*

$\mathbb{Z}_{2,2,2,4}$	weak	$g^{001}_{0\frac{1}{2}0}$	$g^{001}_{\frac{1}{2}00}$	$g^{010}_{00\frac{1}{2}}$	$g^{010}_{\frac{1}{2}00}$	$g^{100}_{00\frac{1}{2}}$	$g^{100}_{0\frac{1}{2}0}$	2^{001}	2^{010}	2^{100}	i	2^{001}_1	2^{010}_1	2^{100}_1
0000	000	0	0	0	0	0	0	0	0	0	0	0	0	0
0000	000	0	0	𝟙	𝟙	𝟙	𝟙	0	𝟙	𝟙	0	0	𝟙	𝟙
0000	000	𝟙	𝟙	0	0	𝟙	𝟙	𝟙	0	𝟙	0	𝟙	0	𝟙
0000	000	𝟙	𝟙	𝟙	𝟙	0	0	𝟙	𝟙	0	0	𝟙	𝟙	0
0002	000	0	0	0	0	𝟙	𝟙	𝟙	𝟙	0	𝟙	𝟙	𝟙	0
0002	000	0	0	𝟙	𝟙	0	0	𝟙	0	𝟙	𝟙	𝟙	0	𝟙
0002	000	𝟙	𝟙	0	0	0	0	0	𝟙	𝟙	𝟙	0	𝟙	𝟙
0002	000	𝟙	𝟙	𝟙	𝟙	𝟙	𝟙	0	0	0	𝟙	0	0	0
1110	111	0	𝟙	0	𝟙	0	𝟙	0	𝟙	0	0	𝟙	0	𝟙
1110	111	0	𝟙	𝟙	0	𝟙	0	0	0	𝟙	0	𝟙	𝟙	0
1110	111	𝟙	0	0	𝟙	𝟙	0	𝟙	𝟙	𝟙	0	0	0	0
1110	111	𝟙	0	𝟙	0	0	𝟙	𝟙	0	0	0	0	𝟙	𝟙
1112	111	0	𝟙	0	𝟙	𝟙	0	𝟙	0	0	1	0	𝟙	𝟙
1112	111	0	𝟙	𝟙	0	0	𝟙	𝟙	𝟙	𝟙	1	0	0	0
1112	111	𝟙	0	0	𝟙	0	𝟙	0	0	𝟙	1	𝟙	𝟙	0
1112	111	𝟙	0	𝟙	0	𝟙	0	0	𝟙	0	1	𝟙	0	𝟙

Space group #74 : *Imma*

$\mathbb{Z}_{2,2,2,4}$	weak	$m^{010}_{(2)}$	$m^{100}_{(2)}$	$g^{001}_{0\frac{1}{2}0}$	$g^{001}_{\frac{1}{2}00}$	$g^{010}_{\frac{1}{2}0\frac{1}{2}}$	$g^{100}_{0\frac{1}{2}\frac{1}{2}}$	2^{001}	2^{010}	2^{100}	i	2^{001}_1	2^{010}_1	2^{100}_1
0000	000	0	0	0	0	0	0	0	0	0	0	0	0	0
0000	000	0	2	𝟙	𝟙	0	𝟙	𝟙	0	𝟙	0	𝟙	0	𝟙
0000	000	2	0	𝟙	𝟙	𝟙	0	𝟙	𝟙	0	0	𝟙	𝟙	0
0000	000	2	2	0	0	𝟙	𝟙	0	𝟙	𝟙	0	0	𝟙	𝟙
0002	000	0	0	𝟙	𝟙	0	0	0	𝟙	𝟙	𝟙	0	𝟙	𝟙
0002	000	0	2	0	0	0	𝟙	𝟙	𝟙	0	𝟙	𝟙	𝟙	0
0002	000	2	0	0	0	𝟙	0	𝟙	0	𝟙	𝟙	𝟙	0	𝟙
0002	000	2	2	𝟙	𝟙	𝟙	𝟙	0	0	0	𝟙	0	0	0
1110	111	0	0	0	𝟙	𝟙	𝟙	0	𝟙	0	0	𝟙	0	𝟙
1110	111	0	2	𝟙	0	𝟙	0	𝟙	𝟙	𝟙	0	0	0	0
1110	111	2	0	𝟙	0	0	𝟙	𝟙	0	0	0	0	𝟙	𝟙
1110	111	2	2	0	𝟙	0	0	0	0	𝟙	0	𝟙	𝟙	0
1112	111	0	0	𝟙	0	𝟙	𝟙	0	0	𝟙	1	𝟙	𝟙	0
1112	111	0	2	0	𝟙	𝟙	0	𝟙	0	0	1	0	𝟙	𝟙
1112	111	2	0	0	𝟙	0	𝟙	𝟙	𝟙	𝟙	1	0	0	0
1112	111	2	2	𝟙	0	0	0	0	𝟙	0	1	𝟙	0	𝟙

Space group #83 : *P4/m*

Continued on next page

Supplementary Table 7 – continued

SI	Invariants					
$\mathbb{Z}_{2,4,8}$	weak	$m^{001}_{(4)}$	2^{001}	4^{001}	i	$\bar{4}^{001}$
000	000	00	0	0	0	0
000	000	04	0	$\mathbb{1}$	0	$\mathbb{1}$
000	000	40	0	$\mathbb{1}$	0	$\mathbb{1}$
000	000	44	0	0	0	0
002	000	20	0	0	$\mathbb{1}$	$\mathbb{1}$
002	000	24	0	$\mathbb{1}$	$\mathbb{1}$	0
002	000	$\bar{2}0$	0	$\mathbb{1}$	$\mathbb{1}$	0
002	000	$\bar{2}4$	0	0	$\mathbb{1}$	$\mathbb{1}$
004	000	00	0	$\mathbb{1}$	0	$\mathbb{1}$
004	000	04	0	0	0	0
004	000	40	0	0	0	0
004	000	44	0	$\mathbb{1}$	0	$\mathbb{1}$
006	000	20	0	$\mathbb{1}$	$\mathbb{1}$	0
006	000	24	0	0	$\mathbb{1}$	$\mathbb{1}$
006	000	$\bar{2}0$	0	0	$\mathbb{1}$	$\mathbb{1}$
006	000	$\bar{2}4$	0	$\mathbb{1}$	$\mathbb{1}$	0
010	001	31	0	$\mathbb{1}$	0	1
010	001	$3\bar{3}$	0	0	0	0
010	001	$\bar{1}1$	0	0	0	0
010	001	$\bar{1}\bar{3}$	0	$\mathbb{1}$	0	1
012	001	11	0	0	1	1
012	001	$1\bar{3}$	0	$\mathbb{1}$	1	0
012	001	$\bar{3}1$	0	$\mathbb{1}$	1	0
012	001	$\bar{3}\bar{3}$	0	0	1	1
014	001	31	0	0	0	0
014	001	$3\bar{3}$	0	$\mathbb{1}$	0	1
014	001	$\bar{1}1$	0	$\mathbb{1}$	0	1
014	001	$\bar{1}\bar{3}$	0	0	0	0
016	001	11	0	$\mathbb{1}$	1	0
016	001	$1\bar{3}$	0	0	1	1
016	001	$\bar{3}1$	0	0	1	1
016	001	$\bar{3}\bar{3}$	0	$\mathbb{1}$	1	0
020	000	22	0	$\mathbb{1}$	0	$\mathbb{1}$
020	000	$2\bar{2}$	0	0	0	0
020	000	$\bar{2}2$	0	0	0	0
020	000	$\bar{2}\bar{2}$	0	$\mathbb{1}$	0	$\mathbb{1}$
022	000	02	0	0	$\mathbb{1}$	$\mathbb{1}$
022	000	$0\bar{2}$	0	$\mathbb{1}$	$\mathbb{1}$	0
022	000	42	0	$\mathbb{1}$	$\mathbb{1}$	0
022	000	$4\bar{2}$	0	0	$\mathbb{1}$	$\mathbb{1}$
024	000	22	0	0	0	0
024	000	$2\bar{2}$	0	$\mathbb{1}$	0	$\mathbb{1}$
024	000	$\bar{2}2$	0	$\mathbb{1}$	0	$\mathbb{1}$
024	000	$\bar{2}\bar{2}$	0	0	0	0
026	000	02	0	$\mathbb{1}$	$\mathbb{1}$	0
026	000	$0\bar{2}$	0	0	$\mathbb{1}$	$\mathbb{1}$
026	000	42	0	0	$\mathbb{1}$	$\mathbb{1}$
026	000	$4\bar{2}$	0	$\mathbb{1}$	$\mathbb{1}$	0
030	001	13	0	$\mathbb{1}$	0	1
030	001	$1\bar{1}$	0	0	0	0
030	001	$\bar{3}3$	0	0	0	0
030	001	$\bar{3}\bar{1}$	0	$\mathbb{1}$	0	1
032	001	33	0	$\mathbb{1}$	1	0
032	001	$3\bar{1}$	0	0	1	1
032	001	$\bar{1}3$	0	0	1	1
032	001	$\bar{1}\bar{1}$	0	$\mathbb{1}$	1	0
034	001	13	0	0	0	0
034	001	$1\bar{1}$	0	$\mathbb{1}$	0	1
034	001	$\bar{3}3$	0	$\mathbb{1}$	0	1

Continued on next page

Supplementary Table 7 – continued

SI	Invariants					
034	001	$\bar{3}\bar{1}$	0	0	0	0
036	001	33	0	0	1	1
036	001	$3\bar{1}$	0	1	1	0
036	001	$\bar{1}3$	0	1	1	0
036	001	$\bar{1}\bar{1}$	0	0	1	1
100	110	00	0	0	0	0
100	110	04	0	1	0	1
100	110	40	0	1	0	1
100	110	44	0	0	0	0
102	110	20	0	0	1	1
102	110	24	0	1	1	0
102	110	$\bar{2}0$	0	1	1	0
102	110	$\bar{2}4$	0	0	1	1
104	110	00	0	1	0	1
104	110	04	0	0	0	0
104	110	40	0	0	0	0
104	110	44	0	1	0	1
106	110	20	0	1	1	0
106	110	24	0	0	1	1
106	110	$\bar{2}0$	0	0	1	1
106	110	$\bar{2}4$	0	1	1	0
110	111	31	0	1	0	1
110	111	$3\bar{3}$	0	0	0	0
110	111	$\bar{1}1$	0	0	0	0
110	111	$\bar{1}\bar{3}$	0	1	0	1
112	111	11	0	0	1	1
112	111	$1\bar{3}$	0	1	1	0
112	111	$\bar{3}1$	0	1	1	0
112	111	$\bar{3}\bar{3}$	0	0	1	1
114	111	31	0	0	0	0
114	111	$3\bar{3}$	0	1	0	1
114	111	$\bar{1}1$	0	1	0	1
114	111	$\bar{1}\bar{3}$	0	0	0	0
116	111	11	0	1	1	0
116	111	$1\bar{3}$	0	0	1	1
116	111	$\bar{3}1$	0	0	1	1
116	111	$\bar{3}\bar{3}$	0	1	1	0
120	110	22	0	1	0	1
120	110	$2\bar{2}$	0	0	0	0
120	110	$\bar{2}2$	0	0	0	0
120	110	$\bar{2}\bar{2}$	0	1	0	1
122	110	02	0	0	1	1
122	110	$0\bar{2}$	0	1	1	0
122	110	42	0	1	1	0
122	110	$4\bar{2}$	0	0	1	1
124	110	22	0	0	0	0
124	110	$2\bar{2}$	0	1	0	1
124	110	$\bar{2}2$	0	1	0	1
124	110	$\bar{2}\bar{2}$	0	0	0	0
126	110	02	0	1	1	0
126	110	$0\bar{2}$	0	0	1	1
126	110	42	0	0	1	1
126	110	$4\bar{2}$	0	1	1	0
130	111	13	0	1	0	1
130	111	$1\bar{1}$	0	0	0	0
130	111	$\bar{3}3$	0	0	0	0
130	111	$\bar{3}\bar{1}$	0	1	0	1
132	111	33	0	1	1	0
132	111	$3\bar{1}$	0	0	1	1
132	111	$\bar{1}3$	0	0	1	1
132	111	$\bar{1}\bar{1}$	0	1	1	0

Continued on next page

Supplementary Table 7 – continued

SI	Invariants					
134	111	13	0	0	0	0
134	111	$1\bar{1}$	0	1	0	1
134	111	$\bar{3}3$	0	1	0	1
134	111	$\bar{3}\bar{1}$	0	0	0	0
136	111	33	0	0	1	1
136	111	$3\bar{1}$	0	1	1	0
136	111	$\bar{1}3$	0	1	1	0
136	111	$\bar{1}\bar{1}$	0	0	1	1

Space group #84 : $P4_2/m$

$\mathbb{Z}_{2,2,2,4}$	weak	$m^{001}_{(4)}$	2^{001}	i	4^{001}_2	$\bar{4}^{001}$
0000	000	00	0	0	0	0
0000	000	00	0	0	$\mathbb{1}$	$\mathbb{1}$
0000	000	40	0	0	0	0
0000	000	40	0	0	$\mathbb{1}$	$\mathbb{1}$
0002	000	20	0	$\mathbb{1}$	0	$\mathbb{1}$
0002	000	20	0	$\mathbb{1}$	$\mathbb{1}$	0
0002	000	$\bar{2}0$	0	$\mathbb{1}$	0	$\mathbb{1}$
0002	000	$\bar{2}0$	0	$\mathbb{1}$	$\mathbb{1}$	0
1100	110	00	0	0	0	0
1100	110	00	0	0	1	1
1100	110	40	0	0	0	0
1100	110	40	0	0	1	1
1102	110	20	0	1	0	1
1102	110	20	0	1	1	0
1102	110	$\bar{2}0$	0	1	0	1
1102	110	$\bar{2}0$	0	1	1	0

Space group #85 : $P4/n$

$\mathbb{Z}_{2,2,2,4}$	weak	$g^{001}_{\frac{1}{2}\frac{1}{2}0}$	2^{001}	4^{001}	i	$\bar{4}^{001}$
0000	000	0	0	0	0	0
0000	000	0	0	$\mathbb{1}$	0	$\mathbb{1}$
0002	000	$\mathbb{1}$	0	0	$\mathbb{1}$	$\mathbb{1}$
0002	000	$\mathbb{1}$	0	$\mathbb{1}$	$\mathbb{1}$	0
0010	001	0	0	0	0	0
0010	001	0	0	$\mathbb{1}$	0	1
0012	001	1	0	0	1	1
0012	001	1	0	$\mathbb{1}$	1	0

Space group #86 : $P4_2/n$

$\mathbb{Z}_{2,2,2,4}$	weak	$g^{001}_{\frac{1}{2}\frac{1}{2}0}$	2^{001}	i	4^{001}_2	$\bar{4}^{001}$
0000	000	0	0	0	0	0
0000	000	0	0	0	$\mathbb{1}$	$\mathbb{1}$
0002	000	$\mathbb{1}$	0	$\mathbb{1}$	0	$\mathbb{1}$
0002	000	$\mathbb{1}$	0	$\mathbb{1}$	$\mathbb{1}$	0

Space group #87 : $I4/m$

$\mathbb{Z}_{2,8}$	weak	$m^{001}_{(4)}$	$g^{001}_{\frac{1}{2}\frac{1}{2}0}$	2^{001}	4^{001}	i	2^{001}_1	4^{001}_2	$\bar{4}^{001}$
00	000	0	0	0	0	0	0	0	0
00	000	4	0	0	$\mathbb{1}$	0	0	$\mathbb{1}$	$\mathbb{1}$
02	000	2	$\mathbb{1}$	0	0	$\mathbb{1}$	0	0	$\mathbb{1}$
02	000	$\bar{2}$	$\mathbb{1}$	0	$\mathbb{1}$	$\mathbb{1}$	0	$\mathbb{1}$	0
04	000	0	0	0	$\mathbb{1}$	0	0	$\mathbb{1}$	$\mathbb{1}$
04	000	4	0	0	0	0	0	0	0
06	000	2	$\mathbb{1}$	0	$\mathbb{1}$	$\mathbb{1}$	0	$\mathbb{1}$	0
06	000	$\bar{2}$	$\mathbb{1}$	0	0	$\mathbb{1}$	0	0	$\mathbb{1}$
10	111	0	$\mathbb{1}$	0	0	0	$\mathbb{1}$	$\mathbb{1}$	0
10	111	4	$\mathbb{1}$	0	$\mathbb{1}$	0	$\mathbb{1}$	0	1
12	111	2	0	0	0	1	$\mathbb{1}$	$\mathbb{1}$	1
12	111	$\bar{2}$	0	0	$\mathbb{1}$	1	$\mathbb{1}$	0	0
14	111	0	$\mathbb{1}$	0	$\mathbb{1}$	0	$\mathbb{1}$	0	1
14	111	4	$\mathbb{1}$	0	0	0	$\mathbb{1}$	$\mathbb{1}$	0

Continued on next page

Supplementary Table 7 – continued

**SI**	**Invariants**											
16	111	2	0	0	𝟙	1	𝟙	0	0			
16	111	$\bar{2}$	0	0	0	1	𝟙	𝟙	1			
Space group #88 : $I4_1/a$												
$\mathbb{Z}_{2,2,2,4}$	weak	$g^{001}_{0\frac{1}{2}0}$	$g^{001}_{\frac{1}{2}00}$	2^{001}	i	2^{001}_1	4^{001}_1	4^{001}_3	$\bar{4}^{001}$			
0000	000	0	0	0	0	0	0	0	0			
0000	000	0	0	0	0	0	𝟙	𝟙	𝟙			
0002	000	𝟙	𝟙	0	𝟙	0	0	0	𝟙			
0002	000	𝟙	𝟙	0	𝟙	0	𝟙	𝟙	0			
Space group #123 : $P4/mmm$												
$\mathbb{Z}_{2,4,8}$	weak	$m^{001}_{(4)}$	$m^{1\bar{1}0}_{(2)}$	$m^{100}_{(2)}$	$g^{1\bar{1}0}_{\frac{1}{2}\frac{1}{2}0}$	2^{001}	2^{100}	2^{110}	4^{001}	i	2^{110}_1	$\bar{4}^{001}$
000	000	00	0	00	0	0	0	0	0	0	0	0
000	000	00	0	22	0	0	0	0	0	0	0	0
000	000	00	2	02	𝟙	0	𝟙	𝟙	0	0	𝟙	0
000	000	00	2	20	𝟙	0	𝟙	𝟙	0	0	𝟙	0
000	000	04	0	02	0	0	𝟙	0	𝟙	0	0	𝟙
000	000	04	0	20	0	0	𝟙	0	𝟙	0	0	𝟙
000	000	04	2	00	𝟙	0	0	𝟙	𝟙	0	𝟙	𝟙
000	000	04	2	22	𝟙	0	0	𝟙	𝟙	0	𝟙	𝟙
000	000	40	0	02	0	0	𝟙	0	𝟙	0	0	𝟙
000	000	40	0	20	0	0	𝟙	0	𝟙	0	0	𝟙
000	000	40	2	00	𝟙	0	0	𝟙	𝟙	0	𝟙	𝟙
000	000	40	2	22	𝟙	0	0	𝟙	𝟙	0	𝟙	𝟙
000	000	44	0	00	0	0	0	0	0	0	0	0
000	000	44	0	22	0	0	0	0	0	0	0	0
000	000	44	2	02	𝟙	0	𝟙	𝟙	0	0	𝟙	0
000	000	44	2	20	𝟙	0	𝟙	𝟙	0	0	𝟙	0
002	000	20	0	00	0	0	𝟙	𝟙	0	𝟙	𝟙	𝟙
002	000	20	0	22	0	0	𝟙	𝟙	0	𝟙	𝟙	𝟙
002	000	20	2	02	𝟙	0	0	0	0	𝟙	0	𝟙
002	000	20	2	20	𝟙	0	0	0	0	𝟙	0	𝟙
002	000	24	0	02	0	0	0	𝟙	𝟙	𝟙	𝟙	0
002	000	24	0	20	0	0	0	𝟙	𝟙	𝟙	𝟙	0
002	000	24	2	00	𝟙	0	𝟙	0	𝟙	𝟙	0	0
002	000	24	2	22	𝟙	0	𝟙	0	𝟙	𝟙	0	0
002	000	$\bar{2}0$	0	02	0	0	0	𝟙	𝟙	𝟙	𝟙	0
002	000	$\bar{2}0$	0	20	0	0	0	𝟙	𝟙	𝟙	𝟙	0
002	000	$\bar{2}0$	2	00	𝟙	0	𝟙	0	𝟙	𝟙	0	0
002	000	$\bar{2}0$	2	22	𝟙	0	𝟙	0	𝟙	𝟙	0	0
002	000	$\bar{2}4$	0	00	0	0	𝟙	𝟙	0	𝟙	𝟙	𝟙
002	000	$\bar{2}4$	0	22	0	0	𝟙	𝟙	0	𝟙	𝟙	𝟙
002	000	$\bar{2}4$	2	02	𝟙	0	0	0	0	𝟙	0	𝟙
002	000	$\bar{2}4$	2	20	𝟙	0	0	0	0	𝟙	0	𝟙
004	000	00	0	02	0	0	𝟙	0	𝟙	0	0	𝟙
004	000	00	0	20	0	0	𝟙	0	𝟙	0	0	𝟙
004	000	00	2	00	𝟙	0	0	𝟙	𝟙	0	𝟙	𝟙
004	000	00	2	22	𝟙	0	0	𝟙	𝟙	0	𝟙	𝟙
004	000	04	0	00	0	0	0	0	0	0	0	0
004	000	04	0	22	0	0	0	0	0	0	0	0
004	000	04	2	02	𝟙	0	𝟙	𝟙	0	0	𝟙	0
004	000	04	2	20	𝟙	0	𝟙	𝟙	0	0	𝟙	0
004	000	40	0	00	0	0	0	0	0	0	0	0
004	000	40	0	22	0	0	0	0	0	0	0	0
004	000	40	2	02	𝟙	0	𝟙	𝟙	0	0	𝟙	0
004	000	40	2	20	𝟙	0	𝟙	𝟙	0	0	𝟙	0
004	000	44	0	02	0	0	𝟙	0	𝟙	0	0	𝟙
004	000	44	0	20	0	0	𝟙	0	𝟙	0	0	𝟙
004	000	44	2	00	𝟙	0	0	𝟙	𝟙	0	𝟙	𝟙
004	000	44	2	22	𝟙	0	0	𝟙	𝟙	0	𝟙	𝟙
006	000	20	0	02	0	0	0	𝟙	𝟙	𝟙	𝟙	0

Continued on next page

Supplementary Table 7 – continued

SI	Invariants											
006	000	20	0	20	0	0	0	$\mathbb{1}$	$\mathbb{1}$	$\mathbb{1}$	$\mathbb{1}$	0
006	000	20	2	00	$\mathbb{1}$	0	$\mathbb{1}$	0	$\mathbb{1}$	$\mathbb{1}$	0	0
006	000	20	2	22	$\mathbb{1}$	0	$\mathbb{1}$	0	$\mathbb{1}$	$\mathbb{1}$	0	0
006	000	24	0	00	0	0	$\mathbb{1}$	$\mathbb{1}$	0	$\mathbb{1}$	$\mathbb{1}$	$\mathbb{1}$
006	000	24	0	22	0	0	$\mathbb{1}$	$\mathbb{1}$	0	$\mathbb{1}$	$\mathbb{1}$	$\mathbb{1}$
006	000	24	2	02	$\mathbb{1}$	0	0	0	0	$\mathbb{1}$	0	$\mathbb{1}$
006	000	24	2	20	$\mathbb{1}$	0	0	0	0	$\mathbb{1}$	0	$\mathbb{1}$
006	000	$\bar{2}0$	0	00	0	0	$\mathbb{1}$	$\mathbb{1}$	0	$\mathbb{1}$	$\mathbb{1}$	$\mathbb{1}$
006	000	$\bar{2}0$	0	22	0	0	$\mathbb{1}$	$\mathbb{1}$	0	$\mathbb{1}$	$\mathbb{1}$	$\mathbb{1}$
006	000	$\bar{2}0$	2	02	$\mathbb{1}$	0	0	0	0	$\mathbb{1}$	0	$\mathbb{1}$
006	000	$\bar{2}0$	2	20	$\mathbb{1}$	0	0	0	0	$\mathbb{1}$	0	$\mathbb{1}$
006	000	$\bar{2}4$	0	02	0	0	0	$\mathbb{1}$	$\mathbb{1}$	$\mathbb{1}$	$\mathbb{1}$	0
006	000	$\bar{2}4$	0	20	0	0	0	$\mathbb{1}$	$\mathbb{1}$	$\mathbb{1}$	$\mathbb{1}$	0
006	000	$\bar{2}4$	2	00	$\mathbb{1}$	0	$\mathbb{1}$	0	$\mathbb{1}$	$\mathbb{1}$	0	0
006	000	$\bar{2}4$	2	22	$\mathbb{1}$	0	$\mathbb{1}$	0	$\mathbb{1}$	$\mathbb{1}$	0	0
010	001	31	0	02	0	0	1	0	$\mathbb{1}$	0	1	1
010	001	31	0	20	0	0	1	0	$\mathbb{1}$	0	1	1
010	001	31	2	00	$\mathbb{1}$	0	0	1	$\mathbb{1}$	0	0	1
010	001	31	2	22	$\mathbb{1}$	0	0	1	$\mathbb{1}$	0	0	1
010	001	$3\bar{3}$	0	00	0	0	0	0	0	0	1	0
010	001	$3\bar{3}$	0	22	0	0	0	0	0	0	1	0
010	001	$3\bar{3}$	2	02	$\mathbb{1}$	0	1	1	0	0	0	0
010	001	$3\bar{3}$	2	20	$\mathbb{1}$	0	1	1	0	0	0	0
010	001	$\bar{1}1$	0	00	0	0	0	0	0	0	1	0
010	001	$\bar{1}1$	0	22	0	0	0	0	0	0	1	0
010	001	$\bar{1}1$	2	02	$\mathbb{1}$	0	1	1	0	0	0	0
010	001	$\bar{1}1$	2	20	$\mathbb{1}$	0	1	1	0	0	0	0
010	001	$\bar{1}\bar{3}$	0	02	0	0	1	0	$\mathbb{1}$	0	1	1
010	001	$\bar{1}\bar{3}$	0	20	0	0	1	0	$\mathbb{1}$	0	1	1
010	001	$\bar{1}\bar{3}$	2	00	$\mathbb{1}$	0	0	1	$\mathbb{1}$	0	0	1
010	001	$\bar{1}\bar{3}$	2	22	$\mathbb{1}$	0	0	1	$\mathbb{1}$	0	0	1
012	001	11	0	00	0	0	1	1	0	1	0	1
012	001	11	0	22	0	0	1	1	0	1	0	1
012	001	11	2	02	$\mathbb{1}$	0	0	0	0	1	1	1
012	001	11	2	20	$\mathbb{1}$	0	0	0	0	1	1	1
012	001	$1\bar{3}$	0	02	0	0	0	1	$\mathbb{1}$	1	0	0
012	001	$1\bar{3}$	0	20	0	0	0	1	$\mathbb{1}$	1	0	0
012	001	$1\bar{3}$	2	00	$\mathbb{1}$	0	1	0	$\mathbb{1}$	1	1	0
012	001	$1\bar{3}$	2	22	$\mathbb{1}$	0	1	0	$\mathbb{1}$	1	1	0
012	001	$\bar{3}1$	0	02	0	0	0	1	$\mathbb{1}$	1	0	0
012	001	$\bar{3}1$	0	20	0	0	0	1	$\mathbb{1}$	1	0	0
012	001	$\bar{3}1$	2	00	$\mathbb{1}$	0	1	0	$\mathbb{1}$	1	1	0
012	001	$\bar{3}1$	2	22	$\mathbb{1}$	0	1	0	$\mathbb{1}$	1	1	0
012	001	$\bar{3}\bar{3}$	0	00	0	0	1	1	0	1	0	1
012	001	$\bar{3}\bar{3}$	0	22	0	0	1	1	0	1	0	1
012	001	$\bar{3}\bar{3}$	2	02	$\mathbb{1}$	0	0	0	0	1	1	1
012	001	$\bar{3}\bar{3}$	2	20	$\mathbb{1}$	0	0	0	0	1	1	1
014	001	31	0	00	0	0	0	0	0	0	1	0
014	001	31	0	22	0	0	0	0	0	0	1	0
014	001	31	2	02	$\mathbb{1}$	0	1	1	0	0	0	0
014	001	31	2	20	$\mathbb{1}$	0	1	1	0	0	0	0
014	001	$3\bar{3}$	0	02	0	0	1	0	$\mathbb{1}$	0	1	1
014	001	$3\bar{3}$	0	20	0	0	1	0	$\mathbb{1}$	0	1	1
014	001	$3\bar{3}$	2	00	$\mathbb{1}$	0	0	1	$\mathbb{1}$	0	0	1
014	001	$3\bar{3}$	2	22	$\mathbb{1}$	0	0	1	$\mathbb{1}$	0	0	1
014	001	$\bar{1}1$	0	02	0	0	1	0	$\mathbb{1}$	0	1	1
014	001	$\bar{1}1$	0	20	0	0	1	0	$\mathbb{1}$	0	1	1
014	001	$\bar{1}1$	2	00	$\mathbb{1}$	0	0	1	$\mathbb{1}$	0	0	1
014	001	$\bar{1}1$	2	22	$\mathbb{1}$	0	0	1	$\mathbb{1}$	0	0	1
014	001	$\bar{1}\bar{3}$	0	00	0	0	0	0	0	0	1	0
014	001	$\bar{1}\bar{3}$	0	22	0	0	0	0	0	0	1	0

Continued on next page

Supplementary Table 7 – continued

SI	Invariants											
014	001	$\bar{1}\bar{3}$	2	02	$\mathbb{1}$	0	1	1	0	0	0	0
014	001	$\bar{1}\bar{3}$	2	20	$\mathbb{1}$	0	1	1	0	0	0	0
016	001	11	0	02	0	0	0	1	$\mathbb{1}$	1	0	0
016	001	11	0	20	0	0	0	1	$\mathbb{1}$	1	0	0
016	001	11	2	00	$\mathbb{1}$	0	1	0	$\mathbb{1}$	1	1	0
016	001	11	2	22	$\mathbb{1}$	0	1	0	$\mathbb{1}$	1	1	0
016	001	$1\bar{3}$	0	00	0	0	1	1	0	1	0	1
016	001	$1\bar{3}$	0	22	0	0	1	1	0	1	0	1
016	001	$1\bar{3}$	2	02	$\mathbb{1}$	0	0	0	0	1	1	1
016	001	$1\bar{3}$	2	20	$\mathbb{1}$	0	0	0	0	1	1	1
016	001	$\bar{3}1$	0	00	0	0	1	1	0	1	0	1
016	001	$\bar{3}1$	0	22	0	0	1	1	0	1	0	1
016	001	$\bar{3}1$	2	02	$\mathbb{1}$	0	0	0	0	1	1	1
016	001	$\bar{3}1$	2	20	$\mathbb{1}$	0	0	0	0	1	1	1
016	001	$\bar{3}\bar{3}$	0	02	0	0	0	1	$\mathbb{1}$	1	0	0
016	001	$\bar{3}\bar{3}$	0	20	0	0	0	1	$\mathbb{1}$	1	0	0
016	001	$\bar{3}\bar{3}$	2	00	$\mathbb{1}$	0	1	0	$\mathbb{1}$	1	1	0
016	001	$\bar{3}\bar{3}$	2	22	$\mathbb{1}$	0	1	0	$\mathbb{1}$	1	1	0
020	000	22	0	02	0	0	$\mathbb{1}$	0	$\mathbb{1}$	0	0	$\mathbb{1}$
020	000	22	0	20	0	0	$\mathbb{1}$	0	$\mathbb{1}$	0	0	$\mathbb{1}$
020	000	22	2	00	$\mathbb{1}$	0	0	$\mathbb{1}$	$\mathbb{1}$	0	$\mathbb{1}$	$\mathbb{1}$
020	000	22	2	22	$\mathbb{1}$	0	0	$\mathbb{1}$	$\mathbb{1}$	0	$\mathbb{1}$	$\mathbb{1}$
020	000	$2\bar{2}$	0	00	0	0	0	0	0	0	0	0
020	000	$2\bar{2}$	0	22	0	0	0	0	0	0	0	0
020	000	$2\bar{2}$	2	02	$\mathbb{1}$	0	$\mathbb{1}$	$\mathbb{1}$	0	0	$\mathbb{1}$	0
020	000	$2\bar{2}$	2	20	$\mathbb{1}$	0	$\mathbb{1}$	$\mathbb{1}$	0	0	$\mathbb{1}$	0
020	000	$\bar{2}2$	0	00	0	0	0	0	0	0	0	0
020	000	$\bar{2}2$	0	22	0	0	0	0	0	0	0	0
020	000	$\bar{2}2$	2	02	$\mathbb{1}$	0	$\mathbb{1}$	$\mathbb{1}$	0	0	$\mathbb{1}$	0
020	000	$\bar{2}2$	2	20	$\mathbb{1}$	0	$\mathbb{1}$	$\mathbb{1}$	0	0	$\mathbb{1}$	0
020	000	$\bar{2}\bar{2}$	0	02	0	0	$\mathbb{1}$	0	$\mathbb{1}$	0	0	$\mathbb{1}$
020	000	$\bar{2}\bar{2}$	0	20	0	0	$\mathbb{1}$	0	$\mathbb{1}$	0	0	$\mathbb{1}$
020	000	$\bar{2}\bar{2}$	2	00	$\mathbb{1}$	0	0	$\mathbb{1}$	$\mathbb{1}$	0	$\mathbb{1}$	$\mathbb{1}$
020	000	$\bar{2}\bar{2}$	2	22	$\mathbb{1}$	0	0	$\mathbb{1}$	$\mathbb{1}$	0	$\mathbb{1}$	$\mathbb{1}$
022	000	02	0	00	0	0	$\mathbb{1}$	$\mathbb{1}$	0	$\mathbb{1}$	$\mathbb{1}$	$\mathbb{1}$
022	000	02	0	22	0	0	$\mathbb{1}$	$\mathbb{1}$	0	$\mathbb{1}$	$\mathbb{1}$	$\mathbb{1}$
022	000	02	2	02	$\mathbb{1}$	0	0	0	0	$\mathbb{1}$	0	$\mathbb{1}$
022	000	02	2	20	$\mathbb{1}$	0	0	0	0	$\mathbb{1}$	0	$\mathbb{1}$
022	000	$0\bar{2}$	0	02	0	0	0	$\mathbb{1}$	$\mathbb{1}$	$\mathbb{1}$	$\mathbb{1}$	0
022	000	$0\bar{2}$	0	20	0	0	0	$\mathbb{1}$	$\mathbb{1}$	$\mathbb{1}$	$\mathbb{1}$	0
022	000	$0\bar{2}$	2	00	$\mathbb{1}$	0	$\mathbb{1}$	0	$\mathbb{1}$	$\mathbb{1}$	0	0
022	000	$0\bar{2}$	2	22	$\mathbb{1}$	0	$\mathbb{1}$	0	$\mathbb{1}$	$\mathbb{1}$	0	0
022	000	42	0	02	0	0	0	$\mathbb{1}$	$\mathbb{1}$	$\mathbb{1}$	$\mathbb{1}$	0
022	000	42	0	20	0	0	0	$\mathbb{1}$	$\mathbb{1}$	$\mathbb{1}$	$\mathbb{1}$	0
022	000	42	2	00	$\mathbb{1}$	0	$\mathbb{1}$	0	$\mathbb{1}$	$\mathbb{1}$	0	0
022	000	42	2	22	$\mathbb{1}$	0	$\mathbb{1}$	0	$\mathbb{1}$	$\mathbb{1}$	0	0
022	000	$4\bar{2}$	0	00	0	0	$\mathbb{1}$	$\mathbb{1}$	0	$\mathbb{1}$	$\mathbb{1}$	$\mathbb{1}$
022	000	$4\bar{2}$	0	22	0	0	$\mathbb{1}$	$\mathbb{1}$	0	$\mathbb{1}$	$\mathbb{1}$	$\mathbb{1}$
022	000	$4\bar{2}$	2	02	$\mathbb{1}$	0	0	0	0	$\mathbb{1}$	0	$\mathbb{1}$
022	000	$4\bar{2}$	2	20	$\mathbb{1}$	0	0	0	0	$\mathbb{1}$	0	$\mathbb{1}$
024	000	22	0	00	0	0	0	0	0	0	0	0
024	000	22	0	22	0	0	0	0	0	0	0	0
024	000	22	2	02	$\mathbb{1}$	0	$\mathbb{1}$	$\mathbb{1}$	0	0	$\mathbb{1}$	0
024	000	22	2	20	$\mathbb{1}$	0	$\mathbb{1}$	$\mathbb{1}$	0	0	$\mathbb{1}$	0
024	000	$2\bar{2}$	0	02	0	0	$\mathbb{1}$	0	$\mathbb{1}$	0	0	$\mathbb{1}$
024	000	$2\bar{2}$	0	20	0	0	$\mathbb{1}$	0	$\mathbb{1}$	0	0	$\mathbb{1}$
024	000	$2\bar{2}$	2	00	$\mathbb{1}$	0	0	$\mathbb{1}$	$\mathbb{1}$	0	$\mathbb{1}$	$\mathbb{1}$
024	000	$2\bar{2}$	2	22	$\mathbb{1}$	0	0	$\mathbb{1}$	$\mathbb{1}$	0	$\mathbb{1}$	$\mathbb{1}$
024	000	$\bar{2}2$	0	02	0	0	$\mathbb{1}$	0	$\mathbb{1}$	0	0	$\mathbb{1}$
024	000	$\bar{2}2$	0	20	0	0	$\mathbb{1}$	0	$\mathbb{1}$	0	0	$\mathbb{1}$
024	000	$\bar{2}2$	2	00	$\mathbb{1}$	0	0	$\mathbb{1}$	$\mathbb{1}$	0	$\mathbb{1}$	$\mathbb{1}$

Continued on next page

Supplementary Table 7 – continued

SI	Invariants											
024	000	$\bar{2}2$	2	22	𝟙	0	0	𝟙	𝟙	0	𝟙	𝟙
024	000	$\bar{2}\bar{2}$	0	00	0	0	0	0	0	0	0	0
024	000	$\bar{2}\bar{2}$	0	22	0	0	0	0	0	0	0	0
024	000	$\bar{2}\bar{2}$	2	02	𝟙	0	𝟙	𝟙	0	0	𝟙	0
024	000	$\bar{2}\bar{2}$	2	20	𝟙	0	𝟙	𝟙	0	0	𝟙	0
026	000	02	0	02	0	0	0	𝟙	𝟙	𝟙	𝟙	0
026	000	02	0	20	0	0	0	𝟙	𝟙	𝟙	𝟙	0
026	000	02	2	00	𝟙	0	𝟙	0	𝟙	𝟙	0	0
026	000	02	2	22	𝟙	0	𝟙	0	𝟙	𝟙	0	0
026	000	$0\bar{2}$	0	00	0	0	𝟙	𝟙	0	𝟙	𝟙	𝟙
026	000	$0\bar{2}$	0	22	0	0	𝟙	𝟙	0	𝟙	𝟙	𝟙
026	000	$0\bar{2}$	2	02	𝟙	0	0	0	0	𝟙	0	𝟙
026	000	$0\bar{2}$	2	20	𝟙	0	0	0	0	𝟙	0	𝟙
026	000	42	0	00	0	0	𝟙	𝟙	0	𝟙	𝟙	𝟙
026	000	42	0	22	0	0	𝟙	𝟙	0	𝟙	𝟙	𝟙
026	000	42	2	02	𝟙	0	0	0	0	𝟙	0	𝟙
026	000	42	2	20	𝟙	0	0	0	0	𝟙	0	𝟙
026	000	$4\bar{2}$	0	02	0	0	0	𝟙	𝟙	𝟙	𝟙	0
026	000	$4\bar{2}$	0	20	0	0	0	𝟙	𝟙	𝟙	𝟙	0
026	000	$4\bar{2}$	2	00	𝟙	0	𝟙	0	𝟙	𝟙	0	0
026	000	$4\bar{2}$	2	22	𝟙	0	𝟙	0	𝟙	𝟙	0	0
030	001	13	0	02	0	0	1	0	𝟙	0	1	1
030	001	13	0	20	0	0	1	0	𝟙	0	1	1
030	001	13	2	00	𝟙	0	0	1	𝟙	0	0	1
030	001	13	2	22	𝟙	0	0	1	𝟙	0	0	1
030	001	$1\bar{1}$	0	00	0	0	0	0	0	0	1	0
030	001	$1\bar{1}$	0	22	0	0	0	0	0	0	1	0
030	001	$1\bar{1}$	2	02	𝟙	0	1	1	0	0	0	0
030	001	$1\bar{1}$	2	20	𝟙	0	1	1	0	0	0	0
030	001	$\bar{3}3$	0	00	0	0	0	0	0	0	1	0
030	001	$\bar{3}3$	0	22	0	0	0	0	0	0	1	0
030	001	$\bar{3}3$	2	02	𝟙	0	1	1	0	0	0	0
030	001	$\bar{3}3$	2	20	𝟙	0	1	1	0	0	0	0
030	001	$\bar{3}\bar{1}$	0	02	0	0	1	0	𝟙	0	1	1
030	001	$\bar{3}\bar{1}$	0	20	0	0	1	0	𝟙	0	1	1
030	001	$\bar{3}\bar{1}$	2	00	𝟙	0	0	1	𝟙	0	0	1
030	001	$\bar{3}\bar{1}$	2	22	𝟙	0	0	1	𝟙	0	0	1
032	001	33	0	02	0	0	0	1	𝟙	1	0	0
032	001	33	0	20	0	0	0	1	𝟙	1	0	0
032	001	33	2	00	𝟙	0	1	0	𝟙	1	1	0
032	001	33	2	22	𝟙	0	1	0	𝟙	1	1	0
032	001	$3\bar{1}$	0	00	0	0	1	1	0	1	0	1
032	001	$3\bar{1}$	0	22	0	0	1	1	0	1	0	1
032	001	$3\bar{1}$	2	02	𝟙	0	0	0	0	1	1	1
032	001	$3\bar{1}$	2	20	𝟙	0	0	0	0	1	1	1
032	001	$\bar{1}3$	0	00	0	0	1	1	0	1	0	1
032	001	$\bar{1}3$	0	22	0	0	1	1	0	1	0	1
032	001	$\bar{1}3$	2	02	𝟙	0	0	0	0	1	1	1
032	001	$\bar{1}3$	2	20	𝟙	0	0	0	0	1	1	1
032	001	$\bar{1}\bar{1}$	0	02	0	0	0	1	𝟙	1	0	0
032	001	$\bar{1}\bar{1}$	0	20	0	0	0	1	𝟙	1	0	0
032	001	$\bar{1}\bar{1}$	2	00	𝟙	0	1	0	𝟙	1	1	0
032	001	$\bar{1}\bar{1}$	2	22	𝟙	0	1	0	𝟙	1	1	0
034	001	13	0	00	0	0	0	0	0	0	1	0
034	001	13	0	22	0	0	0	0	0	0	1	0
034	001	13	2	02	𝟙	0	1	1	0	0	0	0
034	001	13	2	20	𝟙	0	1	1	0	0	0	0
034	001	$1\bar{1}$	0	02	0	0	1	0	𝟙	0	1	1
034	001	$1\bar{1}$	0	20	0	0	1	0	𝟙	0	1	1
034	001	$1\bar{1}$	2	00	𝟙	0	0	1	𝟙	0	0	1
034	001	$1\bar{1}$	2	22	𝟙	0	0	1	𝟙	0	0	1

Continued on next page

Supplementary Table 7 – continued

SI	Invariants											
034	001	$\bar{3}3$	0	02	0	0	1	0	$\mathbb{1}$	0	1	1
034	001	$\bar{3}3$	0	20	0	0	1	0	$\mathbb{1}$	0	1	1
034	001	$\bar{3}3$	2	00	$\mathbb{1}$	0	0	1	$\mathbb{1}$	0	0	1
034	001	$\bar{3}3$	2	22	$\mathbb{1}$	0	0	1	$\mathbb{1}$	0	0	1
034	001	$\bar{3}\bar{1}$	0	00	0	0	0	0	0	0	1	0
034	001	$\bar{3}\bar{1}$	0	22	0	0	0	0	0	0	1	0
034	001	$\bar{3}\bar{1}$	2	02	$\mathbb{1}$	0	1	1	0	0	0	0
034	001	$\bar{3}\bar{1}$	2	20	$\mathbb{1}$	0	1	1	0	0	0	0
036	001	33	0	00	0	0	1	1	0	1	0	1
036	001	33	0	22	0	0	1	1	0	1	0	1
036	001	33	2	02	$\mathbb{1}$	0	0	0	0	1	1	1
036	001	33	2	20	$\mathbb{1}$	0	0	0	0	1	1	1
036	001	$3\bar{1}$	0	02	0	0	0	1	$\mathbb{1}$	1	0	0
036	001	$3\bar{1}$	0	20	0	0	0	1	$\mathbb{1}$	1	0	0
036	001	$3\bar{1}$	2	00	$\mathbb{1}$	0	1	0	$\mathbb{1}$	1	1	0
036	001	$3\bar{1}$	2	22	$\mathbb{1}$	0	1	0	$\mathbb{1}$	1	1	0
036	001	$\bar{1}3$	0	02	0	0	0	1	$\mathbb{1}$	1	0	0
036	001	$\bar{1}3$	0	20	0	0	0	1	$\mathbb{1}$	1	0	0
036	001	$\bar{1}3$	2	00	$\mathbb{1}$	0	1	0	$\mathbb{1}$	1	1	0
036	001	$\bar{1}3$	2	22	$\mathbb{1}$	0	1	0	$\mathbb{1}$	1	1	0
036	001	$\bar{1}\bar{1}$	0	00	0	0	1	1	0	1	0	1
036	001	$\bar{1}\bar{1}$	0	22	0	0	1	1	0	1	0	1
036	001	$\bar{1}\bar{1}$	2	02	$\mathbb{1}$	0	0	0	0	1	1	1
036	001	$\bar{1}\bar{1}$	2	20	$\mathbb{1}$	0	0	0	0	1	1	1
100	110	00	0	$1\bar{1}$	$\mathbb{1}$	0	0	0	0	0	$\mathbb{1}$	0
100	110	00	0	$\bar{1}1$	$\mathbb{1}$	0	0	0	0	0	$\mathbb{1}$	0
100	110	00	2	11	0	0	1	$\mathbb{1}$	0	0	0	0
100	110	00	2	$\bar{1}\bar{1}$	0	0	1	$\mathbb{1}$	0	0	0	0
100	110	04	0	11	$\mathbb{1}$	0	1	0	1	0	$\mathbb{1}$	1
100	110	04	0	$\bar{1}\bar{1}$	$\mathbb{1}$	0	1	0	1	0	$\mathbb{1}$	1
100	110	04	2	$1\bar{1}$	0	0	0	$\mathbb{1}$	1	0	0	1
100	110	04	2	$\bar{1}1$	0	0	0	$\mathbb{1}$	1	0	0	1
100	110	40	0	11	$\mathbb{1}$	0	1	0	1	0	$\mathbb{1}$	1
100	110	40	0	$\bar{1}\bar{1}$	$\mathbb{1}$	0	1	0	1	0	$\mathbb{1}$	1
100	110	40	2	$1\bar{1}$	0	0	0	$\mathbb{1}$	1	0	0	1
100	110	40	2	$\bar{1}1$	0	0	0	$\mathbb{1}$	1	0	0	1
100	110	44	0	$1\bar{1}$	$\mathbb{1}$	0	0	0	0	0	$\mathbb{1}$	0
100	110	44	0	$\bar{1}1$	$\mathbb{1}$	0	0	0	0	0	$\mathbb{1}$	0
100	110	44	2	11	0	0	1	$\mathbb{1}$	0	0	0	0
100	110	44	2	$\bar{1}\bar{1}$	0	0	1	$\mathbb{1}$	0	0	0	0
102	110	20	0	$1\bar{1}$	$\mathbb{1}$	0	1	$\mathbb{1}$	0	1	0	1
102	110	20	0	$\bar{1}1$	$\mathbb{1}$	0	1	$\mathbb{1}$	0	1	0	1
102	110	20	2	11	0	0	0	0	0	1	$\mathbb{1}$	1
102	110	20	2	$\bar{1}\bar{1}$	0	0	0	0	0	1	$\mathbb{1}$	1
102	110	24	0	11	$\mathbb{1}$	0	0	$\mathbb{1}$	1	1	0	0
102	110	24	0	$\bar{1}\bar{1}$	$\mathbb{1}$	0	0	$\mathbb{1}$	1	1	0	0
102	110	24	2	$1\bar{1}$	0	0	1	0	1	1	$\mathbb{1}$	0
102	110	24	2	$\bar{1}1$	0	0	1	0	1	1	$\mathbb{1}$	0
102	110	$\bar{2}0$	0	11	$\mathbb{1}$	0	0	$\mathbb{1}$	1	1	0	0
102	110	$\bar{2}0$	0	$\bar{1}\bar{1}$	$\mathbb{1}$	0	0	$\mathbb{1}$	1	1	0	0
102	110	$\bar{2}0$	2	$1\bar{1}$	0	0	1	0	1	1	$\mathbb{1}$	0
102	110	$\bar{2}0$	2	$\bar{1}1$	0	0	1	0	1	1	$\mathbb{1}$	0
102	110	$\bar{2}4$	0	$1\bar{1}$	$\mathbb{1}$	0	1	$\mathbb{1}$	0	1	0	1
102	110	$\bar{2}4$	0	$\bar{1}1$	$\mathbb{1}$	0	1	$\mathbb{1}$	0	1	0	1
102	110	$\bar{2}4$	2	11	0	0	0	0	0	1	$\mathbb{1}$	1
102	110	$\bar{2}4$	2	$\bar{1}\bar{1}$	0	0	0	0	0	1	$\mathbb{1}$	1
104	110	00	0	11	$\mathbb{1}$	0	1	0	1	0	$\mathbb{1}$	1
104	110	00	0	$\bar{1}\bar{1}$	$\mathbb{1}$	0	1	0	1	0	$\mathbb{1}$	1
104	110	00	2	$1\bar{1}$	0	0	0	$\mathbb{1}$	1	0	0	1
104	110	00	2	$\bar{1}1$	0	0	0	$\mathbb{1}$	1	0	0	1
104	110	04	0	$1\bar{1}$	$\mathbb{1}$	0	0	0	0	0	$\mathbb{1}$	0

Continued on next page

Supplementary Table 7 – continued

SI	Invariants											
104	110	04	0	$\bar{1}1$	$\mathbb{1}$	0	0	0	0	0	$\mathbb{1}$	0
104	110	04	2	11	0	0	1	$\mathbb{1}$	0	0	0	0
104	110	04	2	$\bar{1}\bar{1}$	0	0	1	$\mathbb{1}$	0	0	0	0
104	110	40	0	$1\bar{1}$	$\mathbb{1}$	0	0	0	0	0	$\mathbb{1}$	0
104	110	40	0	$\bar{1}1$	$\mathbb{1}$	0	0	0	0	0	$\mathbb{1}$	0
104	110	40	2	11	0	0	1	$\mathbb{1}$	0	0	0	0
104	110	40	2	$\bar{1}\bar{1}$	0	0	1	$\mathbb{1}$	0	0	0	0
104	110	44	0	11	$\mathbb{1}$	0	1	0	1	0	$\mathbb{1}$	1
104	110	44	0	$\bar{1}\bar{1}$	$\mathbb{1}$	0	1	0	1	0	$\mathbb{1}$	1
104	110	44	2	$1\bar{1}$	0	0	0	$\mathbb{1}$	1	0	0	1
104	110	44	2	$\bar{1}1$	0	0	0	$\mathbb{1}$	1	0	0	1
106	110	20	0	11	$\mathbb{1}$	0	0	$\mathbb{1}$	1	1	0	0
106	110	20	0	$\bar{1}\bar{1}$	$\mathbb{1}$	0	0	$\mathbb{1}$	1	1	0	0
106	110	20	2	$1\bar{1}$	0	0	1	0	1	1	$\mathbb{1}$	0
106	110	20	2	$\bar{1}1$	0	0	1	0	1	1	$\mathbb{1}$	0
106	110	24	0	$1\bar{1}$	$\mathbb{1}$	0	1	$\mathbb{1}$	0	1	0	1
106	110	24	0	$\bar{1}1$	$\mathbb{1}$	0	1	$\mathbb{1}$	0	1	0	1
106	110	24	2	11	0	0	0	0	0	1	$\mathbb{1}$	1
106	110	24	2	$\bar{1}\bar{1}$	0	0	0	0	0	1	$\mathbb{1}$	1
106	110	$\bar{2}0$	0	$1\bar{1}$	$\mathbb{1}$	0	1	$\mathbb{1}$	0	1	0	1
106	110	$\bar{2}0$	0	$\bar{1}1$	$\mathbb{1}$	0	1	$\mathbb{1}$	0	1	0	1
106	110	$\bar{2}0$	2	11	0	0	0	0	0	1	$\mathbb{1}$	1
106	110	$\bar{2}0$	2	$\bar{1}\bar{1}$	0	0	0	0	0	1	$\mathbb{1}$	1
106	110	$\bar{2}4$	0	11	$\mathbb{1}$	0	0	$\mathbb{1}$	1	1	0	0
106	110	$\bar{2}4$	0	$\bar{1}\bar{1}$	$\mathbb{1}$	0	0	$\mathbb{1}$	1	1	0	0
106	110	$\bar{2}4$	2	$1\bar{1}$	0	0	1	0	1	1	$\mathbb{1}$	0
106	110	$\bar{2}4$	2	$\bar{1}1$	0	0	1	0	1	1	$\mathbb{1}$	0
110	111	31	0	11	$\mathbb{1}$	0	1	0	1	0	0	1
110	111	31	0	$\bar{1}\bar{1}$	$\mathbb{1}$	0	1	0	1	0	0	1
110	111	31	2	$1\bar{1}$	0	0	0	1	1	0	1	1
110	111	31	2	$\bar{1}1$	0	0	0	1	1	0	1	1
110	111	$3\bar{3}$	0	$1\bar{1}$	$\mathbb{1}$	0	0	0	0	0	0	0
110	111	$3\bar{3}$	0	$\bar{1}1$	$\mathbb{1}$	0	0	0	0	0	0	0
110	111	$3\bar{3}$	2	11	0	0	1	1	0	0	1	0
110	111	$3\bar{3}$	2	$\bar{1}\bar{1}$	0	0	1	1	0	0	1	0
110	111	$\bar{1}1$	0	$1\bar{1}$	$\mathbb{1}$	0	0	0	0	0	0	0
110	111	$\bar{1}1$	0	$\bar{1}1$	$\mathbb{1}$	0	0	0	0	0	0	0
110	111	$\bar{1}1$	2	11	0	0	1	1	0	0	1	0
110	111	$\bar{1}1$	2	$\bar{1}\bar{1}$	0	0	1	1	0	0	1	0
110	111	$\bar{1}\bar{3}$	0	11	$\mathbb{1}$	0	1	0	1	0	0	1
110	111	$\bar{1}\bar{3}$	0	$\bar{1}\bar{1}$	$\mathbb{1}$	0	1	0	1	0	0	1
110	111	$\bar{1}\bar{3}$	2	$1\bar{1}$	0	0	0	1	1	0	1	1
110	111	$\bar{1}\bar{3}$	2	$\bar{1}1$	0	0	0	1	1	0	1	1
112	111	11	0	$1\bar{1}$	$\mathbb{1}$	0	1	1	0	1	1	1
112	111	11	0	$\bar{1}1$	$\mathbb{1}$	0	1	1	0	1	1	1
112	111	11	2	11	0	0	0	0	0	1	0	1
112	111	11	2	$\bar{1}\bar{1}$	0	0	0	0	0	1	0	1
112	111	$1\bar{3}$	0	11	$\mathbb{1}$	0	0	1	1	1	1	0
112	111	$1\bar{3}$	0	$\bar{1}\bar{1}$	$\mathbb{1}$	0	0	1	1	1	1	0
112	111	$1\bar{3}$	2	$1\bar{1}$	0	0	1	0	1	1	0	0
112	111	$1\bar{3}$	2	$\bar{1}1$	0	0	1	0	1	1	0	0
112	111	$\bar{3}1$	0	11	$\mathbb{1}$	0	0	1	1	1	1	0
112	111	$\bar{3}1$	0	$\bar{1}\bar{1}$	$\mathbb{1}$	0	0	1	1	1	1	0
112	111	$\bar{3}1$	2	$1\bar{1}$	0	0	1	0	1	1	0	0
112	111	$\bar{3}1$	2	$\bar{1}1$	0	0	1	0	1	1	0	0
112	111	$\bar{3}\bar{3}$	0	$1\bar{1}$	$\mathbb{1}$	0	1	1	0	1	1	1
112	111	$\bar{3}\bar{3}$	0	$\bar{1}1$	$\mathbb{1}$	0	1	1	0	1	1	1
112	111	$\bar{3}\bar{3}$	2	11	0	0	0	0	0	1	0	1
112	111	$\bar{3}\bar{3}$	2	$\bar{1}\bar{1}$	0	0	0	0	0	1	0	1
114	111	31	0	$1\bar{1}$	$\mathbb{1}$	0	0	0	0	0	0	0
114	111	31	0	$\bar{1}1$	$\mathbb{1}$	0	0	0	0	0	0	0

Continued on next page

Supplementary Table 7 – continued

SI	Invariants											
114	111	31	2	11	0	0	1	1	0	0	1	0
114	111	31	2	$\bar{1}\bar{1}$	0	0	1	1	0	0	1	0
114	111	$3\bar{3}$	0	11	$\mathbb{1}$	0	1	0	1	0	0	1
114	111	$3\bar{3}$	0	$\bar{1}\bar{1}$	$\mathbb{1}$	0	1	0	1	0	0	1
114	111	$3\bar{3}$	2	$1\bar{1}$	0	0	0	1	1	0	1	1
114	111	$3\bar{3}$	2	$\bar{1}1$	0	0	0	1	1	0	1	1
114	111	$\bar{1}1$	0	11	$\mathbb{1}$	0	1	0	1	0	0	1
114	111	$\bar{1}1$	0	$\bar{1}\bar{1}$	$\mathbb{1}$	0	1	0	1	0	0	1
114	111	$\bar{1}1$	2	$1\bar{1}$	0	0	0	1	1	0	1	1
114	111	$\bar{1}1$	2	$\bar{1}1$	0	0	0	1	1	0	1	1
114	111	$\bar{1}\bar{3}$	0	$1\bar{1}$	$\mathbb{1}$	0	0	0	0	0	0	0
114	111	$\bar{1}\bar{3}$	0	$\bar{1}1$	$\mathbb{1}$	0	0	0	0	0	0	0
114	111	$\bar{1}\bar{3}$	2	11	0	0	1	1	0	0	1	0
114	111	$\bar{1}\bar{3}$	2	$\bar{1}\bar{1}$	0	0	1	1	0	0	1	0
116	111	11	0	11	$\mathbb{1}$	0	0	1	1	1	1	0
116	111	11	0	$\bar{1}\bar{1}$	$\mathbb{1}$	0	0	1	1	1	1	0
116	111	11	2	$1\bar{1}$	0	0	1	0	1	1	0	0
116	111	11	2	$\bar{1}1$	0	0	1	0	1	1	0	0
116	111	$1\bar{3}$	0	$1\bar{1}$	$\mathbb{1}$	0	1	1	0	1	1	1
116	111	$1\bar{3}$	0	$\bar{1}1$	$\mathbb{1}$	0	1	1	0	1	1	1
116	111	$1\bar{3}$	2	11	0	0	0	0	0	1	0	1
116	111	$1\bar{3}$	2	$\bar{1}\bar{1}$	0	0	0	0	0	1	0	1
116	111	$\bar{3}1$	0	$1\bar{1}$	$\mathbb{1}$	0	1	1	0	1	1	1
116	111	$\bar{3}1$	0	$\bar{1}1$	$\mathbb{1}$	0	1	1	0	1	1	1
116	111	$\bar{3}1$	2	11	0	0	0	0	0	1	0	1
116	111	$\bar{3}1$	2	$\bar{1}\bar{1}$	0	0	0	0	0	1	0	1
116	111	$\bar{3}\bar{3}$	0	11	$\mathbb{1}$	0	0	1	1	1	1	0
116	111	$\bar{3}\bar{3}$	0	$\bar{1}\bar{1}$	$\mathbb{1}$	0	0	1	1	1	1	0
116	111	$\bar{3}\bar{3}$	2	$1\bar{1}$	0	0	1	0	1	1	0	0
116	111	$\bar{3}\bar{3}$	2	$\bar{1}1$	0	0	1	0	1	1	0	0
120	110	22	0	11	$\mathbb{1}$	0	1	0	1	0	$\mathbb{1}$	1
120	110	22	0	$\bar{1}\bar{1}$	$\mathbb{1}$	0	1	0	1	0	$\mathbb{1}$	1
120	110	22	2	$1\bar{1}$	0	0	0	$\mathbb{1}$	1	0	0	1
120	110	22	2	$\bar{1}1$	0	0	0	$\mathbb{1}$	1	0	0	1
120	110	$2\bar{2}$	0	$1\bar{1}$	$\mathbb{1}$	0	0	0	0	0	$\mathbb{1}$	0
120	110	$2\bar{2}$	0	$\bar{1}1$	$\mathbb{1}$	0	0	0	0	0	$\mathbb{1}$	0
120	110	$2\bar{2}$	2	11	0	0	1	$\mathbb{1}$	0	0	0	0
120	110	$2\bar{2}$	2	$\bar{1}\bar{1}$	0	0	1	$\mathbb{1}$	0	0	0	0
120	110	$\bar{2}2$	0	$1\bar{1}$	$\mathbb{1}$	0	0	0	0	0	$\mathbb{1}$	0
120	110	$\bar{2}2$	0	$\bar{1}1$	$\mathbb{1}$	0	0	0	0	0	$\mathbb{1}$	0
120	110	$\bar{2}2$	2	11	0	0	1	$\mathbb{1}$	0	0	0	0
120	110	$\bar{2}2$	2	$\bar{1}\bar{1}$	0	0	1	$\mathbb{1}$	0	0	0	0
120	110	$\bar{2}\bar{2}$	0	11	$\mathbb{1}$	0	1	0	1	0	$\mathbb{1}$	1
120	110	$\bar{2}\bar{2}$	0	$\bar{1}\bar{1}$	$\mathbb{1}$	0	1	0	1	0	$\mathbb{1}$	1
120	110	$\bar{2}\bar{2}$	2	$1\bar{1}$	0	0	0	$\mathbb{1}$	1	0	0	1
120	110	$\bar{2}\bar{2}$	2	$\bar{1}1$	0	0	0	$\mathbb{1}$	1	0	0	1
122	110	02	0	$1\bar{1}$	$\mathbb{1}$	0	1	$\mathbb{1}$	0	1	0	1
122	110	02	0	$\bar{1}1$	$\mathbb{1}$	0	1	$\mathbb{1}$	0	1	0	1
122	110	02	2	11	0	0	0	0	0	1	$\mathbb{1}$	1
122	110	02	2	$\bar{1}\bar{1}$	0	0	0	0	0	1	$\mathbb{1}$	1
122	110	$0\bar{2}$	0	11	$\mathbb{1}$	0	0	$\mathbb{1}$	1	1	0	0
122	110	$0\bar{2}$	0	$\bar{1}\bar{1}$	$\mathbb{1}$	0	0	$\mathbb{1}$	1	1	0	0
122	110	$0\bar{2}$	2	$1\bar{1}$	0	0	1	0	1	1	$\mathbb{1}$	0
122	110	$0\bar{2}$	2	$\bar{1}1$	0	0	1	0	1	1	$\mathbb{1}$	0
122	110	42	0	11	$\mathbb{1}$	0	0	$\mathbb{1}$	1	1	0	0
122	110	42	0	$\bar{1}\bar{1}$	$\mathbb{1}$	0	0	$\mathbb{1}$	1	1	0	0
122	110	42	2	$1\bar{1}$	0	0	1	0	1	1	$\mathbb{1}$	0
122	110	42	2	$\bar{1}1$	0	0	1	0	1	1	$\mathbb{1}$	0
122	110	$4\bar{2}$	0	$1\bar{1}$	$\mathbb{1}$	0	1	$\mathbb{1}$	0	1	0	1
122	110	$4\bar{2}$	0	$\bar{1}1$	$\mathbb{1}$	0	1	$\mathbb{1}$	0	1	0	1
122	110	$4\bar{2}$	2	11	0	0	0	0	0	1	$\mathbb{1}$	1

Continued on next page

Supplementary Table 7 – continued

SI	Invariants											
122	110	$4\bar{2}$	2	$1\bar{1}$	0	0	0	0	0	1	𝟙	1
124	110	22	0	$1\bar{1}$	𝟙	0	0	0	0	0	𝟙	0
124	110	22	0	$\bar{1}1$	𝟙	0	0	0	0	0	𝟙	0
124	110	22	2	11	0	0	1	𝟙	0	0	0	0
124	110	22	2	$\bar{1}\bar{1}$	0	0	1	𝟙	0	0	0	0
124	110	$2\bar{2}$	0	11	𝟙	0	1	0	1	0	𝟙	1
124	110	$2\bar{2}$	0	$\bar{1}\bar{1}$	𝟙	0	1	0	1	0	𝟙	1
124	110	$2\bar{2}$	2	$1\bar{1}$	0	0	0	𝟙	1	0	0	1
124	110	$2\bar{2}$	2	$\bar{1}1$	0	0	0	𝟙	1	0	0	1
124	110	$\bar{2}2$	0	11	𝟙	0	1	0	1	0	𝟙	1
124	110	$\bar{2}2$	0	$\bar{1}\bar{1}$	𝟙	0	1	0	1	0	𝟙	1
124	110	$\bar{2}2$	2	$1\bar{1}$	0	0	0	𝟙	1	0	0	1
124	110	$\bar{2}2$	2	$\bar{1}1$	0	0	0	𝟙	1	0	0	1
124	110	$\bar{2}\bar{2}$	0	$1\bar{1}$	𝟙	0	0	0	0	0	𝟙	0
124	110	$\bar{2}\bar{2}$	0	$\bar{1}1$	𝟙	0	0	0	0	0	𝟙	0
124	110	$\bar{2}\bar{2}$	2	11	0	0	1	𝟙	0	0	0	0
124	110	$\bar{2}\bar{2}$	2	$\bar{1}\bar{1}$	0	0	1	𝟙	0	0	0	0
126	110	02	0	11	𝟙	0	0	𝟙	1	1	0	0
126	110	02	0	$\bar{1}\bar{1}$	𝟙	0	0	𝟙	1	1	0	0
126	110	02	2	$1\bar{1}$	0	0	1	0	1	1	𝟙	0
126	110	02	2	$\bar{1}1$	0	0	1	0	1	1	𝟙	0
126	110	$0\bar{2}$	0	$1\bar{1}$	𝟙	0	1	𝟙	0	1	0	1
126	110	$0\bar{2}$	0	$\bar{1}1$	𝟙	0	1	𝟙	0	1	0	1
126	110	$0\bar{2}$	2	11	0	0	0	0	0	1	𝟙	1
126	110	$0\bar{2}$	2	$\bar{1}\bar{1}$	0	0	0	0	0	1	𝟙	1
126	110	42	0	$1\bar{1}$	𝟙	0	1	𝟙	0	1	0	1
126	110	42	0	$\bar{1}1$	𝟙	0	1	𝟙	0	1	0	1
126	110	42	2	11	0	0	0	0	0	1	𝟙	1
126	110	42	2	$\bar{1}\bar{1}$	0	0	0	0	0	1	𝟙	1
126	110	$4\bar{2}$	0	11	𝟙	0	0	𝟙	1	1	0	0
126	110	$4\bar{2}$	0	$\bar{1}\bar{1}$	𝟙	0	0	𝟙	1	1	0	0
126	110	$4\bar{2}$	2	$1\bar{1}$	0	0	1	0	1	1	𝟙	0
126	110	$4\bar{2}$	2	$\bar{1}1$	0	0	1	0	1	1	𝟙	0
130	111	13	0	11	𝟙	0	1	0	1	0	0	1
130	111	13	0	$\bar{1}\bar{1}$	𝟙	0	1	0	1	0	0	1
130	111	13	2	$1\bar{1}$	0	0	0	1	1	0	1	1
130	111	13	2	$\bar{1}1$	0	0	0	1	1	0	1	1
130	111	$1\bar{1}$	0	$1\bar{1}$	𝟙	0	0	0	0	0	0	0
130	111	$1\bar{1}$	0	$\bar{1}1$	𝟙	0	0	0	0	0	0	0
130	111	$1\bar{1}$	2	11	0	0	1	1	0	0	1	0
130	111	$1\bar{1}$	2	$\bar{1}\bar{1}$	0	0	1	1	0	0	1	0
130	111	$\bar{3}3$	0	$1\bar{1}$	𝟙	0	0	0	0	0	0	0
130	111	$\bar{3}3$	0	$\bar{1}1$	𝟙	0	0	0	0	0	0	0
130	111	$\bar{3}3$	2	11	0	0	1	1	0	0	1	0
130	111	$\bar{3}3$	2	$\bar{1}\bar{1}$	0	0	1	1	0	0	1	0
130	111	$\bar{3}\bar{1}$	0	11	𝟙	0	1	0	1	0	0	1
130	111	$\bar{3}\bar{1}$	0	$\bar{1}\bar{1}$	𝟙	0	1	0	1	0	0	1
130	111	$\bar{3}\bar{1}$	2	$1\bar{1}$	0	0	0	1	1	0	1	1
130	111	$\bar{3}\bar{1}$	2	$\bar{1}1$	0	0	0	1	1	0	1	1
132	111	33	0	11	𝟙	0	0	1	1	1	1	0
132	111	33	0	$\bar{1}\bar{1}$	𝟙	0	0	1	1	1	1	0
132	111	33	2	$1\bar{1}$	0	0	1	0	1	1	0	0
132	111	33	2	$\bar{1}1$	0	0	1	0	1	1	0	0
132	111	$3\bar{1}$	0	$1\bar{1}$	𝟙	0	1	1	0	1	1	1
132	111	$3\bar{1}$	0	$\bar{1}1$	𝟙	0	1	1	0	1	1	1
132	111	$3\bar{1}$	2	11	0	0	0	0	0	1	0	1
132	111	$3\bar{1}$	2	$\bar{1}\bar{1}$	0	0	0	0	0	1	0	1
132	111	$\bar{1}3$	0	$1\bar{1}$	𝟙	0	1	1	0	1	1	1
132	111	$\bar{1}3$	0	$\bar{1}1$	𝟙	0	1	1	0	1	1	1
132	111	$\bar{1}3$	2	11	0	0	0	0	0	1	0	1
132	111	$\bar{1}3$	2	$\bar{1}\bar{1}$	0	0	0	0	0	1	0	1

Continued on next page

Supplementary Table 7 – continued

SI	Invariants										
132	111	$\bar{1}\bar{1}$	0	11	$\mathbb{1}$	0	0	1	1	1	1	0
132	111	$\bar{1}\bar{1}$	0	$\bar{1}\bar{1}$	$\mathbb{1}$	0	0	1	1	1	1	0
132	111	$\bar{1}\bar{1}$	2	$1\bar{1}$	0	0	1	0	1	1	0	0
132	111	$\bar{1}\bar{1}$	2	$\bar{1}1$	0	0	1	0	1	1	0	0
134	111	13	0	$1\bar{1}$	$\mathbb{1}$	0	0	0	0	0	0	0
134	111	13	0	$\bar{1}1$	$\mathbb{1}$	0	0	0	0	0	0	0
134	111	13	2	11	0	0	1	1	0	0	1	0
134	111	13	2	$\bar{1}\bar{1}$	0	0	1	1	0	0	1	0
134	111	$1\bar{1}$	0	11	$\mathbb{1}$	0	1	0	1	0	0	1
134	111	$1\bar{1}$	0	$\bar{1}\bar{1}$	$\mathbb{1}$	0	1	0	1	0	0	1
134	111	$1\bar{1}$	2	$1\bar{1}$	0	0	0	1	1	0	1	1
134	111	$1\bar{1}$	2	$\bar{1}1$	0	0	0	1	1	0	1	1
134	111	$\bar{3}3$	0	11	$\mathbb{1}$	0	1	0	1	0	0	1
134	111	$\bar{3}3$	0	$\bar{1}\bar{1}$	$\mathbb{1}$	0	1	0	1	0	0	1
134	111	$\bar{3}3$	2	$1\bar{1}$	0	0	0	1	1	0	1	1
134	111	$\bar{3}3$	2	$\bar{1}1$	0	0	0	1	1	0	1	1
134	111	$\bar{3}\bar{1}$	0	$1\bar{1}$	$\mathbb{1}$	0	0	0	0	0	0	0
134	111	$\bar{3}\bar{1}$	0	$\bar{1}1$	$\mathbb{1}$	0	0	0	0	0	0	0
134	111	$\bar{3}\bar{1}$	2	11	0	0	1	1	0	0	1	0
134	111	$\bar{3}\bar{1}$	2	$\bar{1}\bar{1}$	0	0	1	1	0	0	1	0
136	111	33	0	$1\bar{1}$	$\mathbb{1}$	0	1	1	0	1	1	1
136	111	33	0	$\bar{1}1$	$\mathbb{1}$	0	1	1	0	1	1	1
136	111	33	2	11	0	0	0	0	0	1	0	1
136	111	33	2	$\bar{1}\bar{1}$	0	0	0	0	0	1	0	1
136	111	$3\bar{1}$	0	11	$\mathbb{1}$	0	0	1	1	1	1	0
136	111	$3\bar{1}$	0	$\bar{1}\bar{1}$	$\mathbb{1}$	0	0	1	1	1	1	0
136	111	$3\bar{1}$	2	$1\bar{1}$	0	0	1	0	1	1	0	0
136	111	$3\bar{1}$	2	$\bar{1}1$	0	0	1	0	1	1	0	0
136	111	$\bar{1}3$	0	11	$\mathbb{1}$	0	0	1	1	1	1	0
136	111	$\bar{1}3$	0	$\bar{1}\bar{1}$	$\mathbb{1}$	0	0	1	1	1	1	0
136	111	$\bar{1}3$	2	$1\bar{1}$	0	0	1	0	1	1	0	0
136	111	$\bar{1}3$	2	$\bar{1}1$	0	0	1	0	1	1	0	0
136	111	$\bar{1}\bar{1}$	0	$1\bar{1}$	$\mathbb{1}$	0	1	1	0	1	1	1
136	111	$\bar{1}\bar{1}$	0	$\bar{1}1$	$\mathbb{1}$	0	1	1	0	1	1	1
136	111	$\bar{1}\bar{1}$	2	11	0	0	0	0	0	1	0	1
136	111	$\bar{1}\bar{1}$	2	$\bar{1}\bar{1}$	0	0	0	0	0	1	0	1

Space group #124 : $P4/mcc$

$\mathbb{Z}_{2,8}$	weak	$m_{(4)}^{001}$	$g_{00\frac{1}{2}}^{1\bar{1}0}$	$g_{\frac{1}{2}\frac{1}{2}\frac{1}{2}}^{1\bar{1}0}$	$g_{00\frac{1}{2}}^{100}$	2^{001}	2^{100}	2^{110}	4^{001}	i	2_1^{110}	$\bar{4}^{001}$
00	000	00	0	0	0	0	0	0	0	0	0	0
00	000	00	$\mathbb{1}$	$\mathbb{1}$	$\mathbb{1}$	0	$\mathbb{1}$	$\mathbb{1}$	0	0	$\mathbb{1}$	0
00	000	40	0	0	$\mathbb{1}$	0	$\mathbb{1}$	0	$\mathbb{1}$	0	0	$\mathbb{1}$
00	000	40	$\mathbb{1}$	$\mathbb{1}$	0	0	0	$\mathbb{1}$	$\mathbb{1}$	0	$\mathbb{1}$	$\mathbb{1}$
02	000	20	0	0	0	0	$\mathbb{1}$	$\mathbb{1}$	0	$\mathbb{1}$	$\mathbb{1}$	$\mathbb{1}$
02	000	20	$\mathbb{1}$	$\mathbb{1}$	$\mathbb{1}$	0	0	0	0	$\mathbb{1}$	0	$\mathbb{1}$
02	000	$\bar{2}0$	0	0	$\mathbb{1}$	0	0	$\mathbb{1}$	$\mathbb{1}$	$\mathbb{1}$	$\mathbb{1}$	0
02	000	$\bar{2}0$	$\mathbb{1}$	$\mathbb{1}$	0	0	$\mathbb{1}$	0	$\mathbb{1}$	$\mathbb{1}$	0	0
04	000	00	0	0	$\mathbb{1}$	0	$\mathbb{1}$	0	$\mathbb{1}$	0	0	$\mathbb{1}$
04	000	00	$\mathbb{1}$	$\mathbb{1}$	0	0	0	$\mathbb{1}$	$\mathbb{1}$	0	$\mathbb{1}$	$\mathbb{1}$
04	000	40	0	0	0	0	0	0	0	0	0	0
04	000	40	$\mathbb{1}$	$\mathbb{1}$	$\mathbb{1}$	0	$\mathbb{1}$	$\mathbb{1}$	0	0	$\mathbb{1}$	0
06	000	20	0	0	$\mathbb{1}$	0	0	$\mathbb{1}$	$\mathbb{1}$	$\mathbb{1}$	$\mathbb{1}$	0
06	000	20	$\mathbb{1}$	$\mathbb{1}$	0	0	$\mathbb{1}$	0	$\mathbb{1}$	$\mathbb{1}$	0	0
06	000	$\bar{2}0$	0	0	0	0	$\mathbb{1}$	$\mathbb{1}$	0	$\mathbb{1}$	$\mathbb{1}$	$\mathbb{1}$
06	000	$\bar{2}0$	$\mathbb{1}$	$\mathbb{1}$	$\mathbb{1}$	0	0	0	0	$\mathbb{1}$	0	$\mathbb{1}$
10	110	00	0	$\mathbb{1}$	0	0	0	0	0	0	$\mathbb{1}$	0
10	110	00	$\mathbb{1}$	0	1	0	1	$\mathbb{1}$	0	0	0	0
10	110	40	0	$\mathbb{1}$	1	0	1	0	1	0	$\mathbb{1}$	1
10	110	40	$\mathbb{1}$	0	0	0	0	$\mathbb{1}$	1	0	0	1
12	110	20	0	$\mathbb{1}$	0	0	1	$\mathbb{1}$	0	1	0	1
12	110	20	$\mathbb{1}$	0	1	0	0	0	0	1	$\mathbb{1}$	1

Continued on next page

Supplementary Table 7 – continued

SI	Invariants										
12	110	$\bar{2}0$	0	$\mathbb{1}$	1	0	0	$\mathbb{1}$	1	1	0	0
12	110	$\bar{2}0$	$\mathbb{1}$	0	0	0	1	0	1	1	$\mathbb{1}$	0
14	110	00	0	$\mathbb{1}$	1	0	1	0	1	0	$\mathbb{1}$	1
14	110	00	$\mathbb{1}$	0	0	0	0	$\mathbb{1}$	1	0	0	1
14	110	40	0	$\mathbb{1}$	0	0	0	0	0	0	$\mathbb{1}$	0
14	110	40	$\mathbb{1}$	0	1	0	1	$\mathbb{1}$	0	0	0	0
16	110	20	0	$\mathbb{1}$	1	0	0	$\mathbb{1}$	1	1	0	0
16	110	20	$\mathbb{1}$	0	0	0	1	0	1	1	$\mathbb{1}$	0
16	110	$\bar{2}0$	0	$\mathbb{1}$	0	0	1	$\mathbb{1}$	0	1	0	1
16	110	$\bar{2}0$	$\mathbb{1}$	0	1	0	0	0	0	1	$\mathbb{1}$	1

Space group #125 : $P4/nbm$

$\mathbb{Z}_{2,2,2,4}$	weak	$m^{1\bar{1}0}_{(2)}$	$g^{001}_{\frac{1}{2}\frac{1}{2}0}$	$g^{1\bar{1}0}_{\frac{1}{2}\frac{1}{2}0}$	$g^{100}_{0\frac{1}{2}0}$	2^{001}	2^{100}	2^{110}	4^{001}	i	2^{110}_1	$\bar{4}^{001}$
0000	000	0	0	0	0	0	0	0	0	0	0	0
0000	000	0	0	0	$\mathbb{1}$	0	$\mathbb{1}$	0	$\mathbb{1}$	0	0	$\mathbb{1}$
0000	000	2	0	$\mathbb{1}$	0	0	0	$\mathbb{1}$	$\mathbb{1}$	0	$\mathbb{1}$	$\mathbb{1}$
0000	000	2	0	$\mathbb{1}$	$\mathbb{1}$	0	$\mathbb{1}$	$\mathbb{1}$	0	0	$\mathbb{1}$	0
0002	000	0	$\mathbb{1}$	0	0	0	$\mathbb{1}$	$\mathbb{1}$	0	$\mathbb{1}$	$\mathbb{1}$	$\mathbb{1}$
0002	000	0	$\mathbb{1}$	0	$\mathbb{1}$	0	0	$\mathbb{1}$	$\mathbb{1}$	$\mathbb{1}$	$\mathbb{1}$	0
0002	000	2	$\mathbb{1}$	$\mathbb{1}$	0	0	$\mathbb{1}$	0	$\mathbb{1}$	$\mathbb{1}$	0	0
0002	000	2	$\mathbb{1}$	$\mathbb{1}$	$\mathbb{1}$	0	0	0	0	$\mathbb{1}$	0	$\mathbb{1}$
0010	001	0	0	0	0	0	0	0	0	0	1	0
0010	001	0	0	0	$\mathbb{1}$	0	1	0	$\mathbb{1}$	0	1	1
0010	001	2	0	$\mathbb{1}$	0	0	0	1	$\mathbb{1}$	0	0	1
0010	001	2	0	$\mathbb{1}$	$\mathbb{1}$	0	1	1	0	0	0	0
0012	001	0	1	0	0	0	1	1	0	1	0	1
0012	001	0	1	0	$\mathbb{1}$	0	0	1	$\mathbb{1}$	1	0	0
0012	001	2	1	$\mathbb{1}$	0	0	1	0	$\mathbb{1}$	1	1	0
0012	001	2	1	$\mathbb{1}$	$\mathbb{1}$	0	0	0	0	1	1	1

Space group #126 : $P4/nnc$

$\mathbb{Z}_{2,2,2,4}$	weak	$g^{001}_{\frac{1}{2}\frac{1}{2}0}$	$g^{1\bar{1}0}_{00\frac{1}{2}}$	$g^{1\bar{1}0}_{\frac{1}{2}\frac{1}{2}\frac{1}{2}}$	$g^{100}_{0\frac{1}{2}\frac{1}{2}}$	2^{001}	2^{100}	2^{110}	4^{001}	i	2^{110}_1	$\bar{4}^{001}$
0000	000	0	0	0	0	0	0	0	0	0	0	0
0000	000	0	0	0	$\mathbb{1}$	0	$\mathbb{1}$	0	$\mathbb{1}$	0	0	$\mathbb{1}$
0000	000	0	$\mathbb{1}$	$\mathbb{1}$	0	0	0	$\mathbb{1}$	$\mathbb{1}$	0	$\mathbb{1}$	$\mathbb{1}$
0000	000	0	$\mathbb{1}$	$\mathbb{1}$	$\mathbb{1}$	0	$\mathbb{1}$	$\mathbb{1}$	0	0	$\mathbb{1}$	0
0002	000	$\mathbb{1}$	0	0	0	0	$\mathbb{1}$	$\mathbb{1}$	0	$\mathbb{1}$	$\mathbb{1}$	$\mathbb{1}$
0002	000	$\mathbb{1}$	0	0	$\mathbb{1}$	0	0	$\mathbb{1}$	$\mathbb{1}$	$\mathbb{1}$	$\mathbb{1}$	0
0002	000	$\mathbb{1}$	$\mathbb{1}$	$\mathbb{1}$	0	0	$\mathbb{1}$	0	$\mathbb{1}$	$\mathbb{1}$	0	0
0002	000	$\mathbb{1}$	$\mathbb{1}$	$\mathbb{1}$	$\mathbb{1}$	0	0	0	0	$\mathbb{1}$	0	$\mathbb{1}$

Space group #127 : $P4/mbm$

$\mathbb{Z}_{4,8}$	weak	$m^{001}_{(4)}$	$m^{1\bar{1}0}_{(2)}$	$g^{1\bar{1}0}_{\frac{1}{2}\frac{1}{2}0}$	$g^{100}_{0\frac{1}{2}0}$	2^{001}	2^{110}	4^{001}	i	2^{100}_1	2^{110}_1	$\bar{4}^{001}$
00	000	00	0	0	0	0	0	0	0	0	0	0
00	000	00	2	$\mathbb{1}$	$\mathbb{1}$	0	$\mathbb{1}$	0	0	$\mathbb{1}$	$\mathbb{1}$	0
00	000	04	0	0	$\mathbb{1}$	0	0	$\mathbb{1}$	0	$\mathbb{1}$	0	$\mathbb{1}$
00	000	04	2	$\mathbb{1}$	0	0	$\mathbb{1}$	$\mathbb{1}$	0	0	$\mathbb{1}$	$\mathbb{1}$
00	000	40	0	0	$\mathbb{1}$	0	0	$\mathbb{1}$	0	$\mathbb{1}$	0	$\mathbb{1}$
00	000	40	2	$\mathbb{1}$	0	0	$\mathbb{1}$	$\mathbb{1}$	0	0	$\mathbb{1}$	$\mathbb{1}$
00	000	44	0	0	0	0	0	0	0	0	0	0
00	000	44	2	$\mathbb{1}$	$\mathbb{1}$	0	$\mathbb{1}$	0	0	$\mathbb{1}$	$\mathbb{1}$	0
02	000	20	0	0	0	0	$\mathbb{1}$	0	$\mathbb{1}$	$\mathbb{1}$	$\mathbb{1}$	$\mathbb{1}$
02	000	20	2	$\mathbb{1}$	$\mathbb{1}$	0	0	0	$\mathbb{1}$	0	0	$\mathbb{1}$
02	000	24	0	0	$\mathbb{1}$	0	$\mathbb{1}$	$\mathbb{1}$	$\mathbb{1}$	0	$\mathbb{1}$	0
02	000	24	2	$\mathbb{1}$	0	0	0	$\mathbb{1}$	$\mathbb{1}$	$\mathbb{1}$	0	0
02	000	$\bar{2}0$	0	0	$\mathbb{1}$	0	$\mathbb{1}$	$\mathbb{1}$	$\mathbb{1}$	0	$\mathbb{1}$	0
02	000	$\bar{2}0$	2	$\mathbb{1}$	0	0	0	$\mathbb{1}$	$\mathbb{1}$	$\mathbb{1}$	0	0
02	000	$\bar{2}4$	0	0	0	0	$\mathbb{1}$	0	$\mathbb{1}$	$\mathbb{1}$	$\mathbb{1}$	$\mathbb{1}$
02	000	$\bar{2}4$	2	$\mathbb{1}$	$\mathbb{1}$	0	0	0	$\mathbb{1}$	0	0	$\mathbb{1}$
04	000	00	0	0	$\mathbb{1}$	0	0	$\mathbb{1}$	0	$\mathbb{1}$	0	$\mathbb{1}$
04	000	00	2	$\mathbb{1}$	0	0	$\mathbb{1}$	$\mathbb{1}$	0	0	$\mathbb{1}$	$\mathbb{1}$

Continued on next page

Supplementary Table 7 – continued

SI	Invariants											
04	000	04	0	0	0	0	0	0	0	0	0	0
04	000	04	2	1	1	0	1	0	0	1	1	0
04	000	40	0	0	0	0	0	0	0	0	0	0
04	000	40	2	1	1	0	1	0	0	1	1	0
04	000	44	0	0	1	0	0	1	0	1	0	1
04	000	44	2	1	0	0	1	1	0	0	1	1
06	000	20	0	0	1	0	1	1	1	0	1	0
06	000	20	2	1	0	0	0	1	1	1	0	0
06	000	24	0	0	0	0	1	0	1	1	1	1
06	000	24	2	1	1	0	0	0	1	0	0	1
06	000	$\bar{2}0$	0	0	0	0	1	0	1	1	1	1
06	000	$\bar{2}0$	2	1	1	0	0	0	1	0	0	1
06	000	$\bar{2}4$	0	0	1	0	1	1	1	0	1	0
06	000	$\bar{2}4$	2	1	0	0	0	1	1	1	0	0
10	001	31	0	0	1	0	1	1	0	1	0	1
10	001	31	2	1	0	0	0	1	0	0	1	1
10	001	$3\bar{3}$	0	0	0	0	1	0	0	0	0	0
10	001	$3\bar{3}$	2	1	1	0	0	0	0	1	1	0
10	001	$\bar{1}1$	0	0	0	0	1	0	0	0	0	0
10	001	$\bar{1}1$	2	1	1	0	0	0	0	1	1	0
10	001	$\bar{1}\bar{3}$	0	0	1	0	1	1	0	1	0	1
10	001	$\bar{1}\bar{3}$	2	1	0	0	0	1	0	0	1	1
12	001	11	0	0	0	0	0	0	1	1	1	1
12	001	11	2	1	1	0	1	0	1	0	0	1
12	001	$1\bar{3}$	0	0	1	0	0	1	1	0	1	0
12	001	$1\bar{3}$	2	1	0	0	1	1	1	1	0	0
12	001	$\bar{3}1$	0	0	1	0	0	1	1	0	1	0
12	001	$\bar{3}1$	2	1	0	0	1	1	1	1	0	0
12	001	$\bar{3}\bar{3}$	0	0	0	0	0	0	1	1	1	1
12	001	$\bar{3}\bar{3}$	2	1	1	0	1	0	1	0	0	1
14	001	31	0	0	0	0	1	0	0	0	0	0
14	001	31	2	1	1	0	0	0	0	1	1	0
14	001	$3\bar{3}$	0	0	1	0	1	1	0	1	0	1
14	001	$3\bar{3}$	2	1	0	0	0	1	0	0	1	1
14	001	$\bar{1}1$	0	0	1	0	1	1	0	1	0	1
14	001	$\bar{1}1$	2	1	0	0	0	1	0	0	1	1
14	001	$\bar{1}\bar{3}$	0	0	0	0	1	0	0	0	0	0
14	001	$\bar{1}\bar{3}$	2	1	1	0	0	0	0	1	1	0
16	001	11	0	0	1	0	0	1	1	0	1	0
16	001	11	2	1	0	0	1	1	1	1	0	0
16	001	$1\bar{3}$	0	0	0	0	0	0	1	1	1	1
16	001	$1\bar{3}$	2	1	1	0	1	0	1	0	0	1
16	001	$\bar{3}1$	0	0	0	0	0	0	1	1	1	1
16	001	$\bar{3}1$	2	1	1	0	1	0	1	0	0	1
16	001	$\bar{3}\bar{3}$	0	0	1	0	0	1	1	0	1	0
16	001	$\bar{3}\bar{3}$	2	1	0	0	1	1	1	1	0	0
20	000	22	0	0	1	0	0	1	0	1	0	1
20	000	22	2	1	0	0	1	1	0	0	1	1
20	000	$2\bar{2}$	0	0	0	0	0	0	0	0	0	0
20	000	$2\bar{2}$	2	1	1	0	1	0	0	1	1	0
20	000	$\bar{2}2$	0	0	0	0	0	0	0	0	0	0
20	000	$\bar{2}2$	2	1	1	0	1	0	0	1	1	0
20	000	$\bar{2}\bar{2}$	0	0	1	0	0	1	0	1	0	1
20	000	$\bar{2}\bar{2}$	2	1	0	0	1	1	0	0	1	1
22	000	02	0	0	0	0	1	0	1	1	1	1
22	000	02	2	1	1	0	0	0	1	0	0	1
22	000	$0\bar{2}$	0	0	1	0	1	1	1	0	1	0
22	000	$0\bar{2}$	2	1	0	0	0	1	1	1	0	0
22	000	42	0	0	1	0	1	1	1	0	1	0
22	000	42	2	1	0	0	0	1	1	1	0	0
22	000	$4\bar{2}$	0	0	0	0	1	0	1	1	1	1

Continued on next page

Supplementary Table 7 – continued

SI	Invariants											
22	000	$4\bar{2}$	2	$\mathbb{1}$	$\mathbb{1}$	0	0	0	$\mathbb{1}$	0	0	$\mathbb{1}$
24	000	22	0	0	0	0	0	0	0	0	0	0
24	000	22	2	$\mathbb{1}$	$\mathbb{1}$	0	$\mathbb{1}$	0	0	$\mathbb{1}$	$\mathbb{1}$	0
24	000	$2\bar{2}$	0	0	$\mathbb{1}$	0	0	$\mathbb{1}$	0	$\mathbb{1}$	0	$\mathbb{1}$
24	000	$2\bar{2}$	2	$\mathbb{1}$	0	0	$\mathbb{1}$	$\mathbb{1}$	0	0	$\mathbb{1}$	$\mathbb{1}$
24	000	$\bar{2}2$	0	0	$\mathbb{1}$	0	0	$\mathbb{1}$	0	$\mathbb{1}$	0	$\mathbb{1}$
24	000	$\bar{2}2$	2	$\mathbb{1}$	0	0	$\mathbb{1}$	$\mathbb{1}$	0	0	$\mathbb{1}$	$\mathbb{1}$
24	000	$\bar{2}\bar{2}$	0	0	0	0	0	0	0	0	0	0
24	000	$\bar{2}\bar{2}$	2	$\mathbb{1}$	$\mathbb{1}$	0	$\mathbb{1}$	0	0	$\mathbb{1}$	$\mathbb{1}$	0
26	000	02	0	0	$\mathbb{1}$	0	$\mathbb{1}$	$\mathbb{1}$	$\mathbb{1}$	0	$\mathbb{1}$	0
26	000	02	2	$\mathbb{1}$	0	0	0	$\mathbb{1}$	$\mathbb{1}$	$\mathbb{1}$	0	0
26	000	$0\bar{2}$	0	0	0	0	$\mathbb{1}$	0	$\mathbb{1}$	$\mathbb{1}$	$\mathbb{1}$	$\mathbb{1}$
26	000	$0\bar{2}$	2	$\mathbb{1}$	$\mathbb{1}$	0	0	0	$\mathbb{1}$	0	0	$\mathbb{1}$
26	000	42	0	0	0	0	$\mathbb{1}$	0	$\mathbb{1}$	$\mathbb{1}$	$\mathbb{1}$	$\mathbb{1}$
26	000	42	2	$\mathbb{1}$	$\mathbb{1}$	0	0	0	$\mathbb{1}$	0	0	$\mathbb{1}$
26	000	$4\bar{2}$	0	0	$\mathbb{1}$	0	$\mathbb{1}$	$\mathbb{1}$	$\mathbb{1}$	0	$\mathbb{1}$	0
26	000	$4\bar{2}$	2	$\mathbb{1}$	0	0	0	$\mathbb{1}$	$\mathbb{1}$	$\mathbb{1}$	0	0
30	001	13	0	0	$\mathbb{1}$	0	1	$\mathbb{1}$	0	1	0	1
30	001	13	2	$\mathbb{1}$	0	0	0	$\mathbb{1}$	0	0	1	1
30	001	$1\bar{1}$	0	0	0	0	1	0	0	0	0	0
30	001	$1\bar{1}$	2	$\mathbb{1}$	$\mathbb{1}$	0	0	0	0	1	1	0
30	001	$\bar{3}3$	0	0	0	0	1	0	0	0	0	0
30	001	$\bar{3}3$	2	$\mathbb{1}$	$\mathbb{1}$	0	0	0	0	1	1	0
30	001	$\bar{3}\bar{1}$	0	0	$\mathbb{1}$	0	1	$\mathbb{1}$	0	1	0	1
30	001	$\bar{3}\bar{1}$	2	$\mathbb{1}$	0	0	0	$\mathbb{1}$	0	0	1	1
32	001	33	0	0	$\mathbb{1}$	0	0	$\mathbb{1}$	1	0	1	0
32	001	33	2	$\mathbb{1}$	0	0	1	$\mathbb{1}$	1	1	0	0
32	001	$3\bar{1}$	0	0	0	0	0	0	1	1	1	1
32	001	$3\bar{1}$	2	$\mathbb{1}$	$\mathbb{1}$	0	1	0	1	0	0	1
32	001	$\bar{1}3$	0	0	0	0	0	0	1	1	1	1
32	001	$\bar{1}3$	2	$\mathbb{1}$	$\mathbb{1}$	0	1	0	1	0	0	1
32	001	$\bar{1}\bar{1}$	0	0	$\mathbb{1}$	0	0	$\mathbb{1}$	1	0	1	0
32	001	$\bar{1}\bar{1}$	2	$\mathbb{1}$	0	0	1	$\mathbb{1}$	1	1	0	0
34	001	13	0	0	0	0	1	0	0	0	0	0
34	001	13	2	$\mathbb{1}$	$\mathbb{1}$	0	0	0	0	1	1	0
34	001	$1\bar{1}$	0	0	$\mathbb{1}$	0	1	$\mathbb{1}$	0	1	0	1
34	001	$1\bar{1}$	2	$\mathbb{1}$	0	0	0	$\mathbb{1}$	0	0	1	1
34	001	$\bar{3}3$	0	0	$\mathbb{1}$	0	1	$\mathbb{1}$	0	1	0	1
34	001	$\bar{3}3$	2	$\mathbb{1}$	0	0	0	$\mathbb{1}$	0	0	1	1
34	001	$\bar{3}\bar{1}$	0	0	0	0	1	0	0	0	0	0
34	001	$\bar{3}\bar{1}$	2	$\mathbb{1}$	$\mathbb{1}$	0	0	0	0	1	1	0
36	001	33	0	0	0	0	0	0	1	1	1	1
36	001	33	2	$\mathbb{1}$	$\mathbb{1}$	0	1	0	1	0	0	1
36	001	$3\bar{1}$	0	0	$\mathbb{1}$	0	0	$\mathbb{1}$	1	0	1	0
36	001	$3\bar{1}$	2	$\mathbb{1}$	0	0	1	$\mathbb{1}$	1	1	0	0
36	001	$\bar{1}3$	0	0	$\mathbb{1}$	0	0	$\mathbb{1}$	1	0	1	0
36	001	$\bar{1}3$	2	$\mathbb{1}$	0	0	1	$\mathbb{1}$	1	1	0	0
36	001	$\bar{1}\bar{1}$	0	0	0	0	0	0	1	1	1	1
36	001	$\bar{1}\bar{1}$	2	$\mathbb{1}$	$\mathbb{1}$	0	1	0	1	0	0	1

Space group #128 : $P4/mnc$

$\mathbb{Z}_8$	weak	$m^{001}_{(4)}$	$g^{1\bar{1}0}_{00\frac{1}{2}}$	$g^{1\bar{1}0}_{\frac{1}{2}\frac{1}{2}\frac{1}{2}}$	$g^{100}_{0\frac{1}{2}\frac{1}{2}}$	2^{001}	2^{110}	4^{001}	i	2^{100}_{1}	2^{110}_{1}	$\bar{4}^{001}$
0	000	00	0	0	0	0	0	0	0	0	0	0
0	000	00	$\mathbb{1}$	$\mathbb{1}$	$\mathbb{1}$	0	$\mathbb{1}$	0	0	$\mathbb{1}$	$\mathbb{1}$	0
0	000	40	0	0	$\mathbb{1}$	0	0	$\mathbb{1}$	0	$\mathbb{1}$	0	$\mathbb{1}$
0	000	40	$\mathbb{1}$	$\mathbb{1}$	0	0	$\mathbb{1}$	$\mathbb{1}$	0	0	$\mathbb{1}$	$\mathbb{1}$
2	000	20	0	0	0	0	$\mathbb{1}$	0	$\mathbb{1}$	$\mathbb{1}$	$\mathbb{1}$	$\mathbb{1}$
2	000	20	$\mathbb{1}$	$\mathbb{1}$	$\mathbb{1}$	0	0	0	$\mathbb{1}$	0	0	$\mathbb{1}$
2	000	$\bar{2}0$	0	0	$\mathbb{1}$	0	$\mathbb{1}$	$\mathbb{1}$	$\mathbb{1}$	0	$\mathbb{1}$	0
2	000	$\bar{2}0$	$\mathbb{1}$	$\mathbb{1}$	0	0	0	$\mathbb{1}$	$\mathbb{1}$	$\mathbb{1}$	0	0
4	000	00	0	0	$\mathbb{1}$	0	0	$\mathbb{1}$	0	$\mathbb{1}$	0	$\mathbb{1}$

Continued on next page

Supplementary Table 7 – continued

SI	Invariants											
4	000	00	$\mathbb{1}$	$\mathbb{1}$	0	0	$\mathbb{1}$	$\mathbb{1}$	0	0	$\mathbb{1}$	$\mathbb{1}$
4	000	40	0	0	0	0	0	0	0	0	0	0
4	000	40	$\mathbb{1}$	$\mathbb{1}$	$\mathbb{1}$	0	$\mathbb{1}$	0	0	$\mathbb{1}$	$\mathbb{1}$	0
6	000	20	0	0	$\mathbb{1}$	0	$\mathbb{1}$	$\mathbb{1}$	$\mathbb{1}$	0	$\mathbb{1}$	0
6	000	20	$\mathbb{1}$	$\mathbb{1}$	0	0	0	$\mathbb{1}$	$\mathbb{1}$	$\mathbb{1}$	0	0
6	000	$\bar{2}0$	0	0	0	0	$\mathbb{1}$	0	$\mathbb{1}$	$\mathbb{1}$	$\mathbb{1}$	$\mathbb{1}$
6	000	$\bar{2}0$	$\mathbb{1}$	$\mathbb{1}$	$\mathbb{1}$	0	0	0	$\mathbb{1}$	0	0	$\mathbb{1}$

Space group #129 : $P4/nmm$

$\mathbb{Z}_{2,2,2,4}$	weak	$m^{1\bar{1}0}_{(2)}$	$m^{100}_{(2)}$	$g^{001}_{\frac{1}{2}\frac{1}{2}0}$	$g^{1\bar{1}0}_{\frac{1}{2}\frac{1}{2}0}$	2^{001}	2^{110}	4^{001}	i	2^{100}_1	2^{110}_1	$\bar{4}^{001}$
0000	000	0	00	0	0	0	0	0	0	0	0	0
0000	000	0	20	0	0	0	0	$\mathbb{1}$	0	$\mathbb{1}$	0	$\mathbb{1}$
0000	000	2	00	0	$\mathbb{1}$	0	$\mathbb{1}$	$\mathbb{1}$	0	0	$\mathbb{1}$	$\mathbb{1}$
0000	000	2	20	0	$\mathbb{1}$	0	$\mathbb{1}$	0	0	$\mathbb{1}$	$\mathbb{1}$	0
0002	000	0	00	$\mathbb{1}$	0	0	$\mathbb{1}$	0	$\mathbb{1}$	$\mathbb{1}$	$\mathbb{1}$	$\mathbb{1}$
0002	000	0	20	$\mathbb{1}$	0	0	$\mathbb{1}$	$\mathbb{1}$	$\mathbb{1}$	0	$\mathbb{1}$	0
0002	000	2	00	$\mathbb{1}$	$\mathbb{1}$	0	0	$\mathbb{1}$	$\mathbb{1}$	$\mathbb{1}$	0	0
0002	000	2	20	$\mathbb{1}$	$\mathbb{1}$	0	0	0	$\mathbb{1}$	0	0	$\mathbb{1}$
0010	001	0	00	0	0	0	1	0	0	0	0	0
0010	001	0	20	0	0	0	1	$\mathbb{1}$	0	1	0	1
0010	001	2	00	0	$\mathbb{1}$	0	0	$\mathbb{1}$	0	0	1	1
0010	001	2	20	0	$\mathbb{1}$	0	0	0	0	1	1	0
0012	001	0	00	1	0	0	0	0	1	1	1	1
0012	001	0	20	1	0	0	0	$\mathbb{1}$	1	0	1	0
0012	001	2	00	1	$\mathbb{1}$	0	1	$\mathbb{1}$	1	1	0	0
0012	001	2	20	1	$\mathbb{1}$	0	1	0	1	0	0	1

Space group #130 : $P4/ncc$

$\mathbb{Z}_{2,2,2,4}$	weak	$g^{001}_{\frac{1}{2}\frac{1}{2}0}$	$g^{1\bar{1}0}_{00\frac{1}{2}}$	$g^{1\bar{1}0}_{\frac{1}{2}\frac{1}{2}\frac{1}{2}}$	$g^{100}_{00\frac{1}{2}}$	2^{001}	2^{110}	4^{001}	i	2^{100}_1	2^{110}_1	$\bar{4}^{001}$
0000	000	0	0	0	0	0	0	0	0	0	0	0
0000	000	0	0	0	$\mathbb{1}$	0	0	$\mathbb{1}$	0	$\mathbb{1}$	0	$\mathbb{1}$
0000	000	0	$\mathbb{1}$	$\mathbb{1}$	0	0	$\mathbb{1}$	$\mathbb{1}$	0	0	$\mathbb{1}$	$\mathbb{1}$
0000	000	0	$\mathbb{1}$	$\mathbb{1}$	$\mathbb{1}$	0	$\mathbb{1}$	0	0	$\mathbb{1}$	$\mathbb{1}$	0
0002	000	$\mathbb{1}$	0	0	0	0	$\mathbb{1}$	0	$\mathbb{1}$	$\mathbb{1}$	$\mathbb{1}$	$\mathbb{1}$
0002	000	$\mathbb{1}$	0	0	$\mathbb{1}$	0	$\mathbb{1}$	$\mathbb{1}$	$\mathbb{1}$	0	$\mathbb{1}$	0
0002	000	$\mathbb{1}$	$\mathbb{1}$	$\mathbb{1}$	0	0	0	$\mathbb{1}$	$\mathbb{1}$	$\mathbb{1}$	0	0
0002	000	$\mathbb{1}$	$\mathbb{1}$	$\mathbb{1}$	$\mathbb{1}$	0	0	0	$\mathbb{1}$	0	0	$\mathbb{1}$

Space group #131 : $P4_2/mmc$

$\mathbb{Z}_{2,2,2,4}$	weak	$m^{001}_{(4)}$	$m^{100}_{(2)}$	$g^{1\bar{1}0}_{00\frac{1}{2}}$	$g^{1\bar{1}0}_{\frac{1}{2}\frac{1}{2}\frac{1}{2}}$	2^{001}	2^{100}	2^{110}	i	2^{110}_1	4^{001}_2	$\bar{4}^{001}$
0000	000	00	00	0	0	0	0	0	0	0	0	0
0000	000	00	00	$\mathbb{1}$	$\mathbb{1}$	0	0	$\mathbb{1}$	0	$\mathbb{1}$	$\mathbb{1}$	$\mathbb{1}$
0000	000	00	02	0	0	0	$\mathbb{1}$	0	0	0	$\mathbb{1}$	$\mathbb{1}$
0000	000	00	02	$\mathbb{1}$	$\mathbb{1}$	0	$\mathbb{1}$	$\mathbb{1}$	0	$\mathbb{1}$	0	0
0000	000	00	20	0	0	0	$\mathbb{1}$	0	0	0	$\mathbb{1}$	$\mathbb{1}$
0000	000	00	20	$\mathbb{1}$	$\mathbb{1}$	0	$\mathbb{1}$	$\mathbb{1}$	0	$\mathbb{1}$	0	0
0000	000	00	22	0	0	0	0	0	0	0	0	0
0000	000	00	22	$\mathbb{1}$	$\mathbb{1}$	0	0	$\mathbb{1}$	0	$\mathbb{1}$	$\mathbb{1}$	$\mathbb{1}$
0000	000	40	00	0	0	0	0	0	0	0	0	0
0000	000	40	00	$\mathbb{1}$	$\mathbb{1}$	0	0	$\mathbb{1}$	0	$\mathbb{1}$	$\mathbb{1}$	$\mathbb{1}$
0000	000	40	02	0	0	0	$\mathbb{1}$	0	0	0	$\mathbb{1}$	$\mathbb{1}$
0000	000	40	02	$\mathbb{1}$	$\mathbb{1}$	0	$\mathbb{1}$	$\mathbb{1}$	0	$\mathbb{1}$	0	0
0000	000	40	20	0	0	0	$\mathbb{1}$	0	0	0	$\mathbb{1}$	$\mathbb{1}$
0000	000	40	20	$\mathbb{1}$	$\mathbb{1}$	0	$\mathbb{1}$	$\mathbb{1}$	0	$\mathbb{1}$	0	0
0000	000	40	22	0	0	0	0	0	0	0	0	0
0000	000	40	22	$\mathbb{1}$	$\mathbb{1}$	0	0	$\mathbb{1}$	0	$\mathbb{1}$	$\mathbb{1}$	$\mathbb{1}$
0002	000	20	00	0	0	0	$\mathbb{1}$	$\mathbb{1}$	$\mathbb{1}$	$\mathbb{1}$	0	$\mathbb{1}$
0002	000	20	00	$\mathbb{1}$	$\mathbb{1}$	0	$\mathbb{1}$	0	$\mathbb{1}$	0	$\mathbb{1}$	0
0002	000	20	02	0	0	0	0	$\mathbb{1}$	$\mathbb{1}$	$\mathbb{1}$	$\mathbb{1}$	0
0002	000	20	02	$\mathbb{1}$	$\mathbb{1}$	0	0	0	$\mathbb{1}$	0	0	$\mathbb{1}$
0002	000	20	20	0	0	0	0	$\mathbb{1}$	$\mathbb{1}$	$\mathbb{1}$	$\mathbb{1}$	0

Continued on next page

Supplementary Table 7 – continued

**SI**	**Invariants**											
0002	000	20	20	𝟙	𝟙	0	0	0	𝟙	0	0	𝟙
0002	000	20	22	0	0	0	𝟙	𝟙	𝟙	𝟙	0	𝟙
0002	000	20	22	𝟙	𝟙	0	𝟙	0	𝟙	0	𝟙	0
0002	000	$\bar{2}0$	00	0	0	0	𝟙	𝟙	𝟙	𝟙	0	𝟙
0002	000	$\bar{2}0$	00	𝟙	𝟙	0	𝟙	0	𝟙	0	𝟙	0
0002	000	$\bar{2}0$	02	0	0	0	0	𝟙	𝟙	𝟙	𝟙	0
0002	000	$\bar{2}0$	02	𝟙	𝟙	0	0	0	𝟙	0	0	𝟙
0002	000	$\bar{2}0$	20	0	0	0	0	𝟙	𝟙	𝟙	𝟙	0
0002	000	$\bar{2}0$	20	𝟙	𝟙	0	0	0	𝟙	0	0	𝟙
0002	000	$\bar{2}0$	22	0	0	0	𝟙	𝟙	𝟙	𝟙	0	𝟙
0002	000	$\bar{2}0$	22	𝟙	𝟙	0	𝟙	0	𝟙	0	𝟙	0
1100	110	00	11	0	𝟙	0	1	0	0	𝟙	1	1
1100	110	00	11	𝟙	0	0	1	𝟙	0	0	0	0
1100	110	00	$1\bar{1}$	0	𝟙	0	0	0	0	𝟙	0	0
1100	110	00	$1\bar{1}$	𝟙	0	0	0	𝟙	0	0	1	1
1100	110	00	$\bar{1}1$	0	𝟙	0	0	0	0	𝟙	0	0
1100	110	00	$\bar{1}1$	𝟙	0	0	0	𝟙	0	0	1	1
1100	110	00	$\bar{1}\bar{1}$	0	𝟙	0	1	0	0	𝟙	1	1
1100	110	00	$\bar{1}\bar{1}$	𝟙	0	0	1	𝟙	0	0	0	0
1100	110	40	11	0	𝟙	0	1	0	0	𝟙	1	1
1100	110	40	11	𝟙	0	0	1	𝟙	0	0	0	0
1100	110	40	$1\bar{1}$	0	𝟙	0	0	0	0	𝟙	0	0
1100	110	40	$1\bar{1}$	𝟙	0	0	0	𝟙	0	0	1	1
1100	110	40	$\bar{1}1$	0	𝟙	0	0	0	0	𝟙	0	0
1100	110	40	$\bar{1}1$	𝟙	0	0	0	𝟙	0	0	1	1
1100	110	40	$\bar{1}\bar{1}$	0	𝟙	0	1	0	0	𝟙	1	1
1100	110	40	$\bar{1}\bar{1}$	𝟙	0	0	1	𝟙	0	0	0	0
1102	110	20	11	0	𝟙	0	0	𝟙	1	0	1	0
1102	110	20	11	𝟙	0	0	0	0	1	𝟙	0	1
1102	110	20	$1\bar{1}$	0	𝟙	0	1	𝟙	1	0	0	1
1102	110	20	$1\bar{1}$	𝟙	0	0	1	0	1	𝟙	1	0
1102	110	20	$\bar{1}1$	0	𝟙	0	1	𝟙	1	0	0	1
1102	110	20	$\bar{1}1$	𝟙	0	0	1	0	1	𝟙	1	0
1102	110	20	$\bar{1}\bar{1}$	0	𝟙	0	0	𝟙	1	0	1	0
1102	110	20	$\bar{1}\bar{1}$	𝟙	0	0	0	0	1	𝟙	0	1
1102	110	$\bar{2}0$	11	0	𝟙	0	0	𝟙	1	0	1	0
1102	110	$\bar{2}0$	11	𝟙	0	0	0	0	1	𝟙	0	1
1102	110	$\bar{2}0$	$1\bar{1}$	0	𝟙	0	1	𝟙	1	0	0	1
1102	110	$\bar{2}0$	$1\bar{1}$	𝟙	0	0	1	0	1	𝟙	1	0
1102	110	$\bar{2}0$	$\bar{1}1$	0	𝟙	0	1	𝟙	1	0	0	1
1102	110	$\bar{2}0$	$\bar{1}1$	𝟙	0	0	1	0	1	𝟙	1	0
1102	110	$\bar{2}0$	$\bar{1}\bar{1}$	0	𝟙	0	0	𝟙	1	0	1	0
1102	110	$\bar{2}0$	$\bar{1}\bar{1}$	𝟙	0	0	0	0	1	𝟙	0	1

Space group #132 : $P4_2/mcm$

$\mathbb{Z}_{2,2,2,4}$	weak	$m^{001}_{(4)}$	$m^{1\bar{1}0}_{(2)}$	$g^{1\bar{1}0}_{\frac{1}{2}\frac{1}{2}0}$	$g^{100}_{00\frac{1}{2}}$	2^{001}	2^{100}	2^{110}	i	2^{110}_{1}	4^{001}_{2}	$\bar{4}^{001}$
0000	000	00	0	0	0	0	0	0	0	0	0	0
0000	000	00	0	0	𝟙	0	𝟙	0	0	0	𝟙	𝟙
0000	000	00	2	𝟙	0	0	0	𝟙	0	𝟙	𝟙	𝟙
0000	000	00	2	𝟙	𝟙	0	𝟙	𝟙	0	𝟙	0	0
0000	000	40	0	0	0	0	0	0	0	0	0	0
0000	000	40	0	0	𝟙	0	𝟙	0	0	0	𝟙	𝟙
0000	000	40	2	𝟙	0	0	0	𝟙	0	𝟙	𝟙	𝟙
0000	000	40	2	𝟙	𝟙	0	𝟙	𝟙	0	𝟙	0	0
0002	000	20	0	0	0	0	𝟙	𝟙	𝟙	𝟙	0	𝟙
0002	000	20	0	0	𝟙	0	0	𝟙	𝟙	𝟙	𝟙	0
0002	000	20	2	𝟙	0	0	𝟙	0	𝟙	0	𝟙	0
0002	000	20	2	𝟙	𝟙	0	0	0	𝟙	0	0	𝟙
0002	000	$\bar{2}0$	0	0	0	0	𝟙	𝟙	𝟙	𝟙	0	𝟙
0002	000	$\bar{2}0$	0	0	𝟙	0	0	𝟙	𝟙	𝟙	𝟙	0
0002	000	$\bar{2}0$	2	𝟙	0	0	𝟙	0	𝟙	0	𝟙	0

Continued on next page

Supplementary Table 7 – continued

SI	Invariants											
0002	000	$\bar{2}0$	2	$\mathbb{1}$	$\mathbb{1}$	0	0	0	$\mathbb{1}$	0	0	$\mathbb{1}$
1100	110	00	0	$\mathbb{1}$	0	0	0	0	0	$\mathbb{1}$	0	0
1100	110	00	0	$\mathbb{1}$	1	0	1	0	0	$\mathbb{1}$	1	1
1100	110	00	2	0	0	0	0	$\mathbb{1}$	0	0	1	1
1100	110	00	2	0	1	0	1	$\mathbb{1}$	0	0	0	0
1100	110	40	0	$\mathbb{1}$	0	0	0	0	0	$\mathbb{1}$	0	0
1100	110	40	0	$\mathbb{1}$	1	0	1	0	0	$\mathbb{1}$	1	1
1100	110	40	2	0	0	0	0	$\mathbb{1}$	0	0	1	1
1100	110	40	2	0	1	0	1	$\mathbb{1}$	0	0	0	0
1102	110	20	0	$\mathbb{1}$	0	0	1	$\mathbb{1}$	1	0	0	1
1102	110	20	0	$\mathbb{1}$	1	0	0	$\mathbb{1}$	1	0	1	0
1102	110	20	2	0	0	0	1	0	1	$\mathbb{1}$	1	0
1102	110	20	2	0	1	0	0	0	1	$\mathbb{1}$	0	1
1102	110	$\bar{2}0$	0	$\mathbb{1}$	0	0	1	$\mathbb{1}$	1	0	0	1
1102	110	$\bar{2}0$	0	$\mathbb{1}$	1	0	0	$\mathbb{1}$	1	0	1	0
1102	110	$\bar{2}0$	2	0	0	0	1	0	1	$\mathbb{1}$	1	0
1102	110	$\bar{2}0$	2	0	1	0	0	0	1	$\mathbb{1}$	0	1

Space group #133 : $P4_2/nbc$

$\mathbb{Z}_{2,2,2,4}$	weak	$g^{001}_{\frac12\frac12 0}$	$g^{1\bar10}_{00\frac12}$	$g^{1\bar10}_{\frac12\frac12\frac12}$	$g^{100}_{0\frac12 0}$	2^{001}	2^{100}	2^{110}	i	2_1^{110}	4_2^{001}	$\bar4^{001}$
0000	000	0	0	0	0	0	0	0	0	0	0	0
0000	000	0	0	0	$\mathbb{1}$	0	$\mathbb{1}$	0	0	0	$\mathbb{1}$	$\mathbb{1}$
0000	000	0	$\mathbb{1}$	$\mathbb{1}$	0	0	0	$\mathbb{1}$	0	$\mathbb{1}$	$\mathbb{1}$	$\mathbb{1}$
0000	000	0	$\mathbb{1}$	$\mathbb{1}$	$\mathbb{1}$	0	$\mathbb{1}$	$\mathbb{1}$	0	$\mathbb{1}$	0	0
0002	000	$\mathbb{1}$	0	0	0	0	$\mathbb{1}$	$\mathbb{1}$	$\mathbb{1}$	$\mathbb{1}$	0	$\mathbb{1}$
0002	000	$\mathbb{1}$	0	0	$\mathbb{1}$	0	0	$\mathbb{1}$	$\mathbb{1}$	$\mathbb{1}$	$\mathbb{1}$	0
0002	000	$\mathbb{1}$	$\mathbb{1}$	$\mathbb{1}$	0	0	$\mathbb{1}$	0	$\mathbb{1}$	0	$\mathbb{1}$	0
0002	000	$\mathbb{1}$	$\mathbb{1}$	$\mathbb{1}$	$\mathbb{1}$	0	0	0	$\mathbb{1}$	0	0	$\mathbb{1}$

Space group #134 : $P4_2/nnm$

$\mathbb{Z}_{2,2,2,4}$	weak	$m^{1\bar10}_{(2)}$	$g^{001}_{\frac12\frac12 0}$	$g^{1\bar10}_{\frac12\frac12 0}$	$g^{100}_{0\frac12\frac12}$	2^{001}	2^{100}	2^{110}	i	2_1^{110}	4_2^{001}	$\bar4^{001}$
0000	000	0	0	0	0	0	0	0	0	0	0	0
0000	000	0	0	0	$\mathbb{1}$	0	$\mathbb{1}$	0	0	0	$\mathbb{1}$	$\mathbb{1}$
0000	000	2	0	$\mathbb{1}$	0	0	0	$\mathbb{1}$	0	$\mathbb{1}$	$\mathbb{1}$	$\mathbb{1}$
0000	000	2	0	$\mathbb{1}$	$\mathbb{1}$	0	$\mathbb{1}$	$\mathbb{1}$	0	$\mathbb{1}$	0	0
0002	000	0	$\mathbb{1}$	0	0	0	$\mathbb{1}$	$\mathbb{1}$	$\mathbb{1}$	$\mathbb{1}$	0	$\mathbb{1}$
0002	000	0	$\mathbb{1}$	0	$\mathbb{1}$	0	0	$\mathbb{1}$	$\mathbb{1}$	$\mathbb{1}$	$\mathbb{1}$	0
0002	000	2	$\mathbb{1}$	$\mathbb{1}$	0	0	$\mathbb{1}$	0	$\mathbb{1}$	0	$\mathbb{1}$	0
0002	000	2	$\mathbb{1}$	$\mathbb{1}$	$\mathbb{1}$	0	0	0	$\mathbb{1}$	0	0	$\mathbb{1}$

Space group #135 : $P4_2/mbc$

$\mathbb{Z}_{2,2,2,4}$	weak	$m^{001}_{(4)}$	$g^{1\bar10}_{00\frac12}$	$g^{1\bar10}_{\frac12\frac12\frac12}$	$g^{100}_{0\frac12 0}$	2^{001}	2^{110}	i	2_1^{100}	2_1^{110}	4_2^{001}	$\bar4^{001}$
0000	000	00	0	0	0	0	0	0	0	0	0	0
0000	000	00	0	0	$\mathbb{1}$	0	0	0	$\mathbb{1}$	0	$\mathbb{1}$	$\mathbb{1}$
0000	000	00	$\mathbb{1}$	$\mathbb{1}$	0	0	$\mathbb{1}$	0	0	$\mathbb{1}$	$\mathbb{1}$	$\mathbb{1}$
0000	000	00	$\mathbb{1}$	$\mathbb{1}$	$\mathbb{1}$	0	$\mathbb{1}$	0	$\mathbb{1}$	$\mathbb{1}$	0	0
0000	000	40	0	0	0	0	0	0	0	0	0	0
0000	000	40	0	0	$\mathbb{1}$	0	0	0	$\mathbb{1}$	0	$\mathbb{1}$	$\mathbb{1}$
0000	000	40	$\mathbb{1}$	$\mathbb{1}$	0	0	$\mathbb{1}$	0	0	$\mathbb{1}$	$\mathbb{1}$	$\mathbb{1}$
0000	000	40	$\mathbb{1}$	$\mathbb{1}$	$\mathbb{1}$	0	$\mathbb{1}$	0	$\mathbb{1}$	$\mathbb{1}$	0	0
0002	000	20	0	0	0	0	$\mathbb{1}$	$\mathbb{1}$	$\mathbb{1}$	$\mathbb{1}$	0	$\mathbb{1}$
0002	000	20	0	0	$\mathbb{1}$	0	$\mathbb{1}$	$\mathbb{1}$	0	$\mathbb{1}$	$\mathbb{1}$	0
0002	000	20	$\mathbb{1}$	$\mathbb{1}$	0	0	0	$\mathbb{1}$	$\mathbb{1}$	0	$\mathbb{1}$	0
0002	000	20	$\mathbb{1}$	$\mathbb{1}$	$\mathbb{1}$	0	0	$\mathbb{1}$	0	0	0	$\mathbb{1}$
0002	000	$\bar{2}0$	0	0	0	0	$\mathbb{1}$	$\mathbb{1}$	$\mathbb{1}$	$\mathbb{1}$	0	$\mathbb{1}$
0002	000	$\bar{2}0$	0	0	$\mathbb{1}$	0	$\mathbb{1}$	$\mathbb{1}$	0	$\mathbb{1}$	$\mathbb{1}$	0
0002	000	$\bar{2}0$	$\mathbb{1}$	$\mathbb{1}$	0	0	0	$\mathbb{1}$	$\mathbb{1}$	0	$\mathbb{1}$	0
0002	000	$\bar{2}0$	$\mathbb{1}$	$\mathbb{1}$	$\mathbb{1}$	0	0	$\mathbb{1}$	0	0	0	$\mathbb{1}$

Space group #136 : $P4_2/mnm$

$\mathbb{Z}_{2,2,2,4}$	weak	$m^{001}_{(4)}$	$m^{1\bar10}_{(2)}$	$g^{1\bar10}_{\frac12\frac12 0}$	$g^{100}_{0\frac12\frac12}$	2^{001}	2^{110}	i	2_1^{100}	2_1^{110}	4_2^{001}	$\bar4^{001}$
0000	000	00	0	0	0	0	0	0	0	0	0	0

Continued on next page

Supplementary Table 7 – continued

SI	Invariants											
0000	000	00	0	0	𝟙	0	0	0	𝟙	0	𝟙	𝟙
0000	000	00	2	𝟙	0	0	𝟙	0	0	𝟙	𝟙	𝟙
0000	000	00	2	𝟙	𝟙	0	𝟙	0	𝟙	𝟙	0	0
0000	000	40	0	0	0	0	0	0	0	0	0	0
0000	000	40	0	0	𝟙	0	0	0	𝟙	0	𝟙	𝟙
0000	000	40	2	𝟙	0	0	𝟙	0	0	𝟙	𝟙	𝟙
0000	000	40	2	𝟙	𝟙	0	𝟙	0	𝟙	𝟙	0	0
0002	000	20	0	0	0	0	𝟙	𝟙	𝟙	𝟙	0	𝟙
0002	000	20	0	0	𝟙	0	𝟙	𝟙	0	𝟙	𝟙	0
0002	000	20	2	𝟙	0	0	0	𝟙	𝟙	0	𝟙	0
0002	000	20	2	𝟙	𝟙	0	0	𝟙	0	0	0	𝟙
0002	000	$\bar{2}0$	0	0	0	0	𝟙	𝟙	𝟙	𝟙	0	𝟙
0002	000	$\bar{2}0$	0	0	𝟙	0	𝟙	𝟙	0	𝟙	𝟙	0
0002	000	$\bar{2}0$	2	𝟙	0	0	0	𝟙	𝟙	0	𝟙	0
0002	000	$\bar{2}0$	2	𝟙	𝟙	0	0	𝟙	0	0	0	𝟙

Space group #137 : $P4_2/nmc$

$\mathbb{Z}_{2,2,2,4}$	weak	$m_{(2)}^{100}$	$g_{\frac{1}{2}\frac{1}{2}0}^{001}$	$g_{00\frac{1}{2}}^{1\bar{1}0}$	$g_{\frac{1}{2}\frac{1}{2}\frac{1}{2}}^{1\bar{1}0}$	2^{001}	2^{110}	i	2_1^{100}	2_1^{110}	4_2^{001}	$\bar{4}^{001}$
0000	000	00	0	0	0	0	0	0	0	0	0	0
0000	000	00	0	𝟙	𝟙	0	𝟙	0	0	𝟙	𝟙	𝟙
0000	000	20	0	0	0	0	0	0	𝟙	0	𝟙	𝟙
0000	000	20	0	𝟙	𝟙	0	𝟙	0	𝟙	𝟙	0	0
0002	000	00	𝟙	0	0	0	𝟙	𝟙	𝟙	𝟙	0	𝟙
0002	000	00	𝟙	𝟙	𝟙	0	0	𝟙	𝟙	0	𝟙	0
0002	000	20	𝟙	0	0	0	𝟙	𝟙	0	𝟙	𝟙	0
0002	000	20	𝟙	𝟙	𝟙	0	0	𝟙	0	0	0	𝟙

Space group #138 : $P4_2/ncm$

$\mathbb{Z}_{2,2,2,4}$	weak	$m_{(2)}^{1\bar{1}0}$	$g_{\frac{1}{2}\frac{1}{2}0}^{001}$	$g_{\frac{1}{2}\frac{1}{2}0}^{1\bar{1}0}$	$g_{00\frac{1}{2}}^{100}$	2^{001}	2^{110}	i	2_1^{100}	2_1^{110}	4_2^{001}	$\bar{4}^{001}$
0000	000	0	0	0	0	0	0	0	0	0	0	0
0000	000	0	0	0	𝟙	0	0	0	𝟙	0	𝟙	𝟙
0000	000	2	0	𝟙	0	0	𝟙	0	0	𝟙	𝟙	𝟙
0000	000	2	0	𝟙	𝟙	0	𝟙	0	𝟙	𝟙	0	0
0002	000	0	𝟙	0	0	0	𝟙	𝟙	𝟙	𝟙	0	𝟙
0002	000	0	𝟙	0	𝟙	0	𝟙	𝟙	0	𝟙	𝟙	0
0002	000	2	𝟙	𝟙	0	0	0	𝟙	𝟙	0	𝟙	0
0002	000	2	𝟙	𝟙	𝟙	0	0	𝟙	0	0	0	𝟙

Space group #139 : $I4/mmm$

$\mathbb{Z}_{2,8}$	weak	$m_{(4)}^{001}$	$m_{(2)}^{1\bar{1}0}$	$m_{(2)}^{100}$	$g_{\frac{1}{2}\frac{1}{2}0}^{001}$	$g_{\frac{1}{2}\frac{1}{2}0}^{1\bar{1}0}$	$g_{0\frac{1}{2}\frac{1}{2}}^{100}$	2^{001}	2^{100}	2^{110}	4^{001}	i	2_1^{001}	2_1^{100}	2_1^{110}	4_2^{001}	$\bar{4}^{001}$
00	000	0	0	0	0	0	0	0	0	0	0	0	0	0	0	0	0
00	000	0	2	2	0	𝟙	𝟙	0	𝟙	𝟙	0	0	0	𝟙	𝟙	0	0
00	000	4	0	2	0	0	𝟙	0	𝟙	0	𝟙	0	0	𝟙	0	𝟙	𝟙
00	000	4	2	0	0	𝟙	0	0	0	𝟙	𝟙	0	0	0	𝟙	𝟙	𝟙
02	000	2	0	0	𝟙	0	0	0	𝟙	𝟙	0	𝟙	0	𝟙	𝟙	0	𝟙
02	000	2	2	2	𝟙	𝟙	𝟙	0	0	0	0	𝟙	0	0	0	0	𝟙
02	000	$\bar{2}$	0	2	𝟙	0	𝟙	0	0	𝟙	𝟙	𝟙	0	0	𝟙	𝟙	0
02	000	$\bar{2}$	2	0	𝟙	𝟙	0	0	𝟙	0	𝟙	𝟙	0	𝟙	0	𝟙	0
04	000	0	0	2	0	0	𝟙	0	𝟙	0	𝟙	0	0	𝟙	0	𝟙	𝟙
04	000	0	2	0	0	𝟙	0	0	0	𝟙	𝟙	0	0	0	𝟙	𝟙	𝟙
04	000	4	0	0	0	0	0	0	0	0	0	0	0	0	0	0	0
04	000	4	2	2	0	𝟙	𝟙	0	𝟙	𝟙	0	0	0	𝟙	𝟙	0	0
06	000	2	0	2	𝟙	0	𝟙	0	0	𝟙	𝟙	𝟙	0	0	𝟙	𝟙	0
06	000	2	2	0	𝟙	𝟙	0	0	𝟙	0	𝟙	𝟙	0	𝟙	0	𝟙	0
06	000	$\bar{2}$	0	0	𝟙	0	0	0	𝟙	𝟙	0	𝟙	0	𝟙	𝟙	0	𝟙
06	000	$\bar{2}$	2	2	𝟙	𝟙	𝟙	0	0	0	0	𝟙	0	0	0	0	𝟙
10	111	0	0	0	𝟙	0	𝟙	0	0	0	0	0	𝟙	𝟙	1	𝟙	0
10	111	0	2	2	𝟙	𝟙	0	0	𝟙	1	0	0	𝟙	0	0	𝟙	0
10	111	4	0	2	𝟙	0	0	0	𝟙	0	𝟙	0	𝟙	0	1	0	1
10	111	4	2	0	𝟙	𝟙	𝟙	0	0	1	𝟙	0	𝟙	𝟙	0	0	1
12	111	2	0	0	0	0	𝟙	0	𝟙	1	0	1	𝟙	0	0	𝟙	1

Continued on next page

Supplementary Table 7 – continued

SI	Invariants																
12	111	2	2	2	0	𝟙	0	0	0	0	0	1	𝟙	𝟙	1	𝟙	1
12	111	$\bar{2}$	0	2	0	0	0	0	0	1	𝟙	1	𝟙	𝟙	0	0	0
12	111	$\bar{2}$	2	0	0	𝟙	𝟙	0	𝟙	0	𝟙	1	𝟙	0	1	0	0
14	111	0	0	2	𝟙	0	0	0	𝟙	0	𝟙	0	𝟙	0	1	0	1
14	111	0	2	0	𝟙	𝟙	𝟙	0	0	1	𝟙	0	𝟙	𝟙	0	0	1
14	111	4	0	0	𝟙	0	𝟙	0	0	0	0	0	𝟙	𝟙	1	𝟙	0
14	111	4	2	2	𝟙	𝟙	0	0	𝟙	1	0	0	𝟙	0	0	𝟙	0
16	111	2	0	2	0	0	0	0	0	1	𝟙	1	𝟙	𝟙	0	0	0
16	111	2	2	0	0	𝟙	𝟙	0	𝟙	0	𝟙	1	𝟙	0	1	0	0
16	111	$\bar{2}$	0	0	0	0	𝟙	0	𝟙	1	0	1	𝟙	0	0	𝟙	1
16	111	$\bar{2}$	2	2	0	𝟙	0	0	0	0	0	1	𝟙	𝟙	1	𝟙	1
Space group #140 : $I4/mcm$																	
$\mathbb{Z}_{2,8}$	weak	$m^{001}_{(4)}$	$m^{1\bar{1}0}_{(2)}$	$g^{001}_{\frac{1}{2}\frac{1}{2}0}$	$g^{1\bar{1}0}_{\frac{1}{2}\frac{1}{2}0}$	$g^{100}_{00\frac{1}{2}}$	$g^{100}_{0\frac{1}{2}0}$	2^{001}	2^{100}	2^{110}	4^{001}	i	2^{001}_1	2^{100}_1	2^{110}_1	4^{001}_2	$\bar{4}^{001}$
00	000	0	0	0	0	0	0	0	0	0	0	0	0	0	0	0	0
00	000	0	2	0	𝟙	𝟙	𝟙	0	𝟙	𝟙	0	0	0	𝟙	𝟙	0	0
00	000	4	0	0	0	𝟙	𝟙	0	𝟙	0	𝟙	0	0	𝟙	0	𝟙	𝟙
00	000	4	2	0	𝟙	0	0	0	0	𝟙	𝟙	0	0	0	𝟙	𝟙	𝟙
02	000	2	0	𝟙	0	0	0	0	𝟙	𝟙	0	𝟙	0	𝟙	𝟙	0	𝟙
02	000	2	2	𝟙	𝟙	𝟙	𝟙	0	0	0	0	𝟙	0	0	0	0	𝟙
02	000	$\bar{2}$	0	𝟙	0	𝟙	𝟙	0	0	𝟙	𝟙	𝟙	0	0	𝟙	𝟙	0
02	000	$\bar{2}$	2	𝟙	𝟙	0	0	0	𝟙	0	𝟙	𝟙	0	𝟙	0	𝟙	0
04	000	0	0	0	0	𝟙	𝟙	0	𝟙	0	𝟙	0	0	𝟙	0	𝟙	𝟙
04	000	0	2	0	𝟙	0	0	0	0	𝟙	𝟙	0	0	0	𝟙	𝟙	𝟙
04	000	4	0	0	0	0	0	0	0	0	0	0	0	0	0	0	0
04	000	4	2	0	𝟙	𝟙	𝟙	0	𝟙	𝟙	0	0	0	𝟙	𝟙	0	0
06	000	2	0	𝟙	0	𝟙	𝟙	0	0	𝟙	𝟙	𝟙	0	0	𝟙	𝟙	0
06	000	2	2	𝟙	𝟙	0	0	0	𝟙	0	𝟙	𝟙	0	𝟙	0	𝟙	0
06	000	$\bar{2}$	0	𝟙	0	0	0	0	𝟙	𝟙	0	𝟙	0	𝟙	𝟙	0	𝟙
06	000	$\bar{2}$	2	𝟙	𝟙	𝟙	𝟙	0	0	0	0	𝟙	0	0	0	0	𝟙
10	111	0	0	𝟙	0	𝟙	0	0	𝟙	1	0	0	𝟙	0	0	𝟙	0
10	111	0	2	𝟙	𝟙	0	𝟙	0	0	0	0	0	𝟙	𝟙	1	𝟙	0
10	111	4	0	𝟙	0	0	𝟙	0	0	1	𝟙	0	𝟙	𝟙	0	0	1
10	111	4	2	𝟙	𝟙	𝟙	0	0	𝟙	0	𝟙	0	𝟙	0	1	0	1
12	111	2	0	0	0	𝟙	0	0	0	0	0	1	𝟙	𝟙	1	𝟙	1
12	111	2	2	0	𝟙	0	𝟙	0	𝟙	1	0	1	𝟙	0	0	𝟙	1
12	111	$\bar{2}$	0	0	0	0	𝟙	0	𝟙	0	𝟙	1	𝟙	0	1	0	0
12	111	$\bar{2}$	2	0	𝟙	𝟙	0	0	0	1	𝟙	1	𝟙	𝟙	0	0	0
14	111	0	0	𝟙	0	0	𝟙	0	0	1	𝟙	0	𝟙	𝟙	0	0	1
14	111	0	2	𝟙	𝟙	𝟙	0	0	𝟙	0	𝟙	0	𝟙	0	1	0	1
14	111	4	0	𝟙	0	𝟙	0	0	𝟙	1	0	0	𝟙	0	0	𝟙	0
14	111	4	2	𝟙	𝟙	0	𝟙	0	0	0	0	0	𝟙	𝟙	1	𝟙	0
16	111	2	0	0	0	0	𝟙	0	𝟙	0	𝟙	1	𝟙	0	1	0	0
16	111	2	2	0	𝟙	𝟙	0	0	0	1	𝟙	1	𝟙	𝟙	0	0	0
16	111	$\bar{2}$	0	0	0	𝟙	0	0	0	0	0	1	𝟙	𝟙	1	𝟙	1
16	111	$\bar{2}$	2	0	𝟙	0	𝟙	0	𝟙	1	0	1	𝟙	0	0	𝟙	1
Space group #141 : $I4_1/amd$																	
$\mathbb{Z}_{2,2,2,4}$	weak	$m^{100}_{(2)}$	$g^{001}_{0\frac{1}{2}0}$	$g^{001}_{\frac{1}{2}00}$	$g^{1\bar{1}0}_{\frac{1}{4}\frac{1}{4}\bar{\frac{1}{4}}}$	$g^{1\bar{1}0}_{\frac{1}{4}\frac{1}{4}\frac{1}{4}}$	$g^{100}_{0\frac{1}{2}\frac{1}{2}}$	2^{001}	2^{100}	2^{110}	i	2^{001}_1	2^{100}_1	2^{110}_1	4^{001}_1	4^{001}_3	$\bar{4}^{001}$
0000	000	0	0	0	0	0	0	0	0	0	0	0	0	0	0	0	0
0000	000	0	0	0	𝟙	𝟙	0	0	0	𝟙	0	0	0	𝟙	𝟙	𝟙	𝟙
0000	000	2	0	0	0	0	𝟙	0	𝟙	0	0	0	𝟙	0	𝟙	𝟙	𝟙
0000	000	2	0	0	𝟙	𝟙	𝟙	0	𝟙	𝟙	0	0	𝟙	𝟙	0	0	0
0002	000	0	𝟙	𝟙	0	0	0	0	𝟙	𝟙	𝟙	0	𝟙	𝟙	0	0	𝟙
0002	000	0	𝟙	𝟙	𝟙	𝟙	0	0	𝟙	0	𝟙	0	𝟙	0	𝟙	𝟙	0
0002	000	2	𝟙	𝟙	0	0	𝟙	0	0	𝟙	𝟙	0	0	𝟙	𝟙	𝟙	0
0002	000	2	𝟙	𝟙	𝟙	𝟙	𝟙	0	0	0	𝟙	0	0	0	0	0	𝟙
Space group #142 : $I4_1/acd$																	
$\mathbb{Z}_{2,2,2,4}$	weak	$g^{001}_{0\frac{1}{2}0}$	$g^{001}_{\frac{1}{2}00}$	$g^{1\bar{1}0}_{\frac{1}{4}\frac{1}{4}\bar{\frac{1}{4}}}$	$g^{1\bar{1}0}_{\frac{1}{4}\frac{1}{4}\frac{1}{4}}$	$g^{100}_{00\frac{1}{2}}$	$g^{100}_{0\frac{1}{2}0}$	2^{001}	2^{100}	2^{110}	i	2^{001}_1	2^{100}_1	2^{110}_1	4^{001}_1	4^{001}_3	$\bar{4}^{001}$
0000	000	0	0	0	0	0	0	0	0	0	0	0	0	0	0	0	0

Continued on next page

Supplementary Table 7 – continued

SI	Invariants																
0000	000	0	0	0	0	𝟙	𝟙	0	𝟙	0	0	0	𝟙	0	𝟙	𝟙	𝟙
0000	000	0	0	𝟙	𝟙	0	0	0	0	𝟙	0	0	0	𝟙	𝟙	𝟙	𝟙
0000	000	0	0	𝟙	𝟙	𝟙	𝟙	0	𝟙	𝟙	0	0	𝟙	𝟙	0	0	0
0002	000	𝟙	𝟙	0	0	0	0	0	𝟙	𝟙	𝟙	0	𝟙	𝟙	0	0	𝟙
0002	000	𝟙	𝟙	0	0	𝟙	𝟙	0	0	𝟙	𝟙	0	0	𝟙	𝟙	𝟙	0
0002	000	𝟙	𝟙	𝟙	𝟙	0	0	0	𝟙	0	𝟙	0	𝟙	0	𝟙	𝟙	0
0002	000	𝟙	𝟙	𝟙	𝟙	𝟙	𝟙	0	0	0	𝟙	0	0	0	0	0	𝟙

Space group #147 : $P\bar{3}$

$\mathbb{Z}_{2,2,2,4}$	weak	i
0000	000	0
0002	000	𝟙
0010	001	0
0012	001	1

Space group #148 : $R\bar{3}$

$\mathbb{Z}_{2,2,2,4}$	weak	i
0000	000	0
0002	000	𝟙
1110	111	0
1112	111	1

Space group #162 : $P\bar{3}1m$

$\mathbb{Z}_{2,2,2,4}$	weak	$m^{010}_{(2)}$	$g^{010}_{\frac{1}{2}00}$	2^{120}	i	2^{120}_{1}
0000	000	0	0	0	0	0
0000	000	2	𝟙	𝟙	0	𝟙
0002	000	0	0	𝟙	𝟙	𝟙
0002	000	2	𝟙	0	𝟙	0
0010	001	0	0	0	0	0
0010	001	2	𝟙	1	0	1
0012	001	0	0	1	1	1
0012	001	2	𝟙	0	1	0

Space group #163 : $P\bar{3}1c$

$\mathbb{Z}_{2,2,2,4}$	weak	$g^{010}_{00\frac{1}{2}}$	$g^{010}_{\frac{1}{2}0\frac{1}{2}}$	2^{120}	i	2^{120}_{1}
0000	000	0	0	0	0	0
0000	000	𝟙	𝟙	𝟙	0	𝟙
0002	000	0	0	𝟙	𝟙	𝟙
0002	000	𝟙	𝟙	0	𝟙	0

Space group #164 : $P\bar{3}m1$

$\mathbb{Z}_{2,2,2,4}$	weak	$m^{\bar{2}10}_{(2)}$	$g^{\bar{2}10}_{\frac{1}{2}10}$	2^{100}	i	2^{100}_{1}
0000	000	0	0	0	0	0
0000	000	2	𝟙	𝟙	0	𝟙
0002	000	0	0	𝟙	𝟙	𝟙
0002	000	2	𝟙	0	𝟙	0
0010	001	0	0	0	0	0
0010	001	2	𝟙	1	0	1
0012	001	0	0	1	1	1
0012	001	2	𝟙	0	1	0

Space group #165 : $P\bar{3}c1$

$\mathbb{Z}_{2,2,2,4}$	weak	$g^{\bar{2}10}_{00\frac{1}{2}}$	$g^{\bar{2}10}_{\frac{1}{2}1\frac{1}{2}}$	2^{100}	i	2^{100}_{1}
0000	000	0	0	0	0	0
0000	000	𝟙	𝟙	𝟙	0	𝟙
0002	000	0	0	𝟙	𝟙	𝟙
0002	000	𝟙	𝟙	0	𝟙	0

Space group #166 : $R\bar{3}m$

$\mathbb{Z}_{2,2,2,4}$	weak	$m^{\bar{2}10}_{(2)}$	$g^{\bar{2}10}_{\frac{1}{6}\frac{1}{3}\frac{1}{3}}$	2^{100}	i	2^{100}_{1}
0000	000	0	0	0	0	0
0000	000	2	𝟙	𝟙	0	𝟙
0002	000	0	0	𝟙	𝟙	𝟙

Continued on next page

Supplementary Table 7 – continued

SI	Invariants					
0002	000	2	$\mathbb{1}$	0	$\mathbb{1}$	0
1110	111	0	$\mathbb{1}$	0	0	0
1110	111	2	0	1	0	1
1112	111	0	$\mathbb{1}$	1	1	1
1112	111	2	0	0	1	0

Space group #167 : $R\bar{3}c$

$\mathbb{Z}_{2,2,2,4}$	weak	$g^{\bar{2}10}_{\frac{\bar{1}}{6}\frac{\bar{1}}{3}\frac{1}{6}}$	$g^{\bar{2}10}_{00\frac{1}{2}}$	2^{100}	i	2^{100}_{1}
0000	000	0	0	0	0	0
0000	000	$\mathbb{1}$	$\mathbb{1}$	$\mathbb{1}$	0	$\mathbb{1}$
0002	000	0	0	$\mathbb{1}$	$\mathbb{1}$	$\mathbb{1}$
0002	000	$\mathbb{1}$	$\mathbb{1}$	0	$\mathbb{1}$	0

Space group #174 : $P\bar{6}$

$\mathbb{Z}_{3,3}$	weak	$m^{001}_{(3)}$
00	000	00
00	001	33
01	000	$0\bar{2}$
01	001	31
02	000	02
02	001	$3\bar{1}$
10	000	$\bar{2}0$
10	001	13
11	000	$\bar{2}\bar{2}$
11	001	11
12	000	$\bar{2}2$
12	001	$1\bar{1}$
20	000	20
20	001	$\bar{1}3$
21	000	$2\bar{2}$
21	001	$\bar{1}1$
22	000	22
22	001	$\bar{1}\bar{1}$

Space group #175 : $P6/m$

$\mathbb{Z}_{6,12}$	weak	$m^{001}_{(6)}$	2^{001}	6^{001}	i
0, 0	000	00	0	0	0
0, 0	000	06	$\mathbb{1}$	$\mathbb{1}$	0
0, 0	000	60	$\mathbb{1}$	$\mathbb{1}$	0
0, 0	000	66	0	0	0
0, 2	000	20	0	0	$\mathbb{1}$
0, 2	000	26	$\mathbb{1}$	$\mathbb{1}$	$\mathbb{1}$
0, 2	000	$\bar{4}0$	$\mathbb{1}$	$\mathbb{1}$	$\mathbb{1}$
0, 2	000	$\bar{4}6$	0	0	$\mathbb{1}$
0, 4	000	40	0	0	0
0, 4	000	46	$\mathbb{1}$	$\mathbb{1}$	0
0, 4	000	$\bar{2}0$	$\mathbb{1}$	$\mathbb{1}$	0
0, 4	000	$\bar{2}6$	0	0	0
0, 6	000	00	$\mathbb{1}$	$\mathbb{1}$	$\mathbb{1}$
0, 6	000	06	0	0	$\mathbb{1}$
0, 6	000	60	0	0	$\mathbb{1}$
0, 6	000	66	$\mathbb{1}$	$\mathbb{1}$	$\mathbb{1}$
0, 8	000	20	$\mathbb{1}$	$\mathbb{1}$	0
0, 8	000	26	0	0	0
0, 8	000	$\bar{4}0$	0	0	0
0, 8	000	$\bar{4}6$	$\mathbb{1}$	$\mathbb{1}$	0
0,10	000	40	$\mathbb{1}$	$\mathbb{1}$	$\mathbb{1}$
0,10	000	46	0	0	$\mathbb{1}$
0,10	000	$\bar{2}0$	0	0	$\mathbb{1}$
0,10	000	$\bar{2}6$	$\mathbb{1}$	$\mathbb{1}$	$\mathbb{1}$
1, 0	001	51	$\mathbb{1}$	$\mathbb{1}$	0
1, 0	001	$5\bar{5}$	0	0	0

Continued on next page

Supplementary Table 7 – continued

SI	Invariants				
1, 0	001	$\bar{1}1$	0	0	0
1, 0	001	$\bar{1}\bar{5}$	$\mathbb{1}$	$\mathbb{1}$	0
1, 2	001	11	0	0	1
1, 2	001	$1\bar{5}$	$\mathbb{1}$	$\mathbb{1}$	1
1, 2	001	$\bar{5}1$	$\mathbb{1}$	$\mathbb{1}$	1
1, 2	001	$\bar{5}\bar{5}$	0	0	1
1, 4	001	31	0	0	0
1, 4	001	$3\bar{5}$	$\mathbb{1}$	$\mathbb{1}$	0
1, 4	001	$\bar{3}1$	$\mathbb{1}$	$\mathbb{1}$	0
1, 4	001	$\bar{3}\bar{5}$	0	0	0
1, 6	001	51	0	0	1
1, 6	001	$5\bar{5}$	$\mathbb{1}$	$\mathbb{1}$	1
1, 6	001	$\bar{1}1$	$\mathbb{1}$	$\mathbb{1}$	1
1, 6	001	$\bar{1}\bar{5}$	0	0	1
1, 8	001	11	$\mathbb{1}$	$\mathbb{1}$	0
1, 8	001	$1\bar{5}$	0	0	0
1, 8	001	$\bar{5}1$	0	0	0
1, 8	001	$\bar{5}\bar{5}$	$\mathbb{1}$	$\mathbb{1}$	0
1,10	001	31	$\mathbb{1}$	$\mathbb{1}$	1
1,10	001	$3\bar{5}$	0	0	1
1,10	001	$\bar{3}1$	0	0	1
1,10	001	$\bar{3}\bar{5}$	$\mathbb{1}$	$\mathbb{1}$	1
2, 0	000	42	$\mathbb{1}$	$\mathbb{1}$	0
2, 0	000	$4\bar{4}$	0	0	0
2, 0	000	$\bar{2}2$	0	0	0
2, 0	000	$\bar{2}\bar{4}$	$\mathbb{1}$	$\mathbb{1}$	0
2, 2	000	02	0	0	$\mathbb{1}$
2, 2	000	$0\bar{4}$	$\mathbb{1}$	$\mathbb{1}$	$\mathbb{1}$
2, 2	000	62	$\mathbb{1}$	$\mathbb{1}$	$\mathbb{1}$
2, 2	000	$6\bar{4}$	0	0	$\mathbb{1}$
2, 4	000	22	0	0	0
2, 4	000	$2\bar{4}$	$\mathbb{1}$	$\mathbb{1}$	0
2, 4	000	$\bar{4}2$	$\mathbb{1}$	$\mathbb{1}$	0
2, 4	000	$\bar{4}\bar{4}$	0	0	0
2, 6	000	42	0	0	$\mathbb{1}$
2, 6	000	$4\bar{4}$	$\mathbb{1}$	$\mathbb{1}$	$\mathbb{1}$
2, 6	000	$\bar{2}2$	$\mathbb{1}$	$\mathbb{1}$	$\mathbb{1}$
2, 6	000	$\bar{2}\bar{4}$	0	0	$\mathbb{1}$
2, 8	000	02	$\mathbb{1}$	$\mathbb{1}$	0
2, 8	000	$0\bar{4}$	0	0	0
2, 8	000	62	0	0	0
2, 8	000	$6\bar{4}$	$\mathbb{1}$	$\mathbb{1}$	0
2,10	000	22	$\mathbb{1}$	$\mathbb{1}$	$\mathbb{1}$
2,10	000	$2\bar{4}$	0	0	$\mathbb{1}$
2,10	000	$\bar{4}2$	0	0	$\mathbb{1}$
2,10	000	$\bar{4}\bar{4}$	$\mathbb{1}$	$\mathbb{1}$	$\mathbb{1}$
3, 0	001	33	$\mathbb{1}$	$\mathbb{1}$	0
3, 0	001	$3\bar{3}$	0	0	0
3, 0	001	$\bar{3}3$	0	0	0
3, 0	001	$\bar{3}\bar{3}$	$\mathbb{1}$	$\mathbb{1}$	0
3, 2	001	53	$\mathbb{1}$	$\mathbb{1}$	1
3, 2	001	$5\bar{3}$	0	0	1
3, 2	001	$\bar{1}3$	0	0	1
3, 2	001	$\bar{1}\bar{3}$	$\mathbb{1}$	$\mathbb{1}$	1
3, 4	001	13	0	0	0
3, 4	001	$1\bar{3}$	$\mathbb{1}$	$\mathbb{1}$	0
3, 4	001	$\bar{5}3$	$\mathbb{1}$	$\mathbb{1}$	0
3, 4	001	$\bar{5}\bar{3}$	0	0	0
3, 6	001	33	0	0	1
3, 6	001	$3\bar{3}$	$\mathbb{1}$	$\mathbb{1}$	1
3, 6	001	$\bar{3}3$	$\mathbb{1}$	$\mathbb{1}$	1

Continued on next page

Supplementary Table 7 – continued

SI	Invariants				
3, 6	001	$3\bar{3}$	0	0	1
3, 8	001	53	0	0	0
3, 8	001	$5\bar{3}$	$\mathbb{1}$	$\mathbb{1}$	0
3, 8	001	$\bar{1}3$	$\mathbb{1}$	$\mathbb{1}$	0
3, 8	001	$\bar{1}\bar{3}$	0	0	0
3,10	001	13	$\mathbb{1}$	$\mathbb{1}$	1
3,10	001	$1\bar{3}$	0	0	1
3,10	001	$\bar{5}3$	0	0	1
3,10	001	$\bar{5}\bar{3}$	$\mathbb{1}$	$\mathbb{1}$	1
4, 0	000	24	$\mathbb{1}$	$\mathbb{1}$	0
4, 0	000	$2\bar{2}$	0	0	0
4, 0	000	$\bar{4}4$	0	0	0
4, 0	000	$\bar{4}\bar{2}$	$\mathbb{1}$	$\mathbb{1}$	0
4, 2	000	44	$\mathbb{1}$	$\mathbb{1}$	$\mathbb{1}$
4, 2	000	$4\bar{2}$	0	0	$\mathbb{1}$
4, 2	000	$\bar{2}4$	0	0	$\mathbb{1}$
4, 2	000	$\bar{2}\bar{2}$	$\mathbb{1}$	$\mathbb{1}$	$\mathbb{1}$
4, 4	000	04	0	0	0
4, 4	000	$0\bar{2}$	$\mathbb{1}$	$\mathbb{1}$	0
4, 4	000	64	$\mathbb{1}$	$\mathbb{1}$	0
4, 4	000	$6\bar{2}$	0	0	0
4, 6	000	24	0	0	$\mathbb{1}$
4, 6	000	$2\bar{2}$	$\mathbb{1}$	$\mathbb{1}$	$\mathbb{1}$
4, 6	000	$\bar{4}4$	$\mathbb{1}$	$\mathbb{1}$	$\mathbb{1}$
4, 6	000	$\bar{4}\bar{2}$	0	0	$\mathbb{1}$
4, 8	000	44	0	0	0
4, 8	000	$4\bar{2}$	$\mathbb{1}$	$\mathbb{1}$	0
4, 8	000	$\bar{2}4$	$\mathbb{1}$	$\mathbb{1}$	0
4, 8	000	$\bar{2}\bar{2}$	0	0	0
4,10	000	04	$\mathbb{1}$	$\mathbb{1}$	$\mathbb{1}$
4,10	000	$0\bar{2}$	0	0	$\mathbb{1}$
4,10	000	64	0	0	$\mathbb{1}$
4,10	000	$6\bar{2}$	$\mathbb{1}$	$\mathbb{1}$	$\mathbb{1}$
5, 0	001	15	$\mathbb{1}$	$\mathbb{1}$	0
5, 0	001	$1\bar{1}$	0	0	0
5, 0	001	$\bar{5}5$	0	0	0
5, 0	001	$\bar{5}\bar{1}$	$\mathbb{1}$	$\mathbb{1}$	0
5, 2	001	35	$\mathbb{1}$	$\mathbb{1}$	1
5, 2	001	$3\bar{1}$	0	0	1
5, 2	001	$\bar{3}5$	0	0	1
5, 2	001	$\bar{3}\bar{1}$	$\mathbb{1}$	$\mathbb{1}$	1
5, 4	001	55	$\mathbb{1}$	$\mathbb{1}$	0
5, 4	001	$5\bar{1}$	0	0	0
5, 4	001	$\bar{1}5$	0	0	0
5, 4	001	$\bar{1}\bar{1}$	$\mathbb{1}$	$\mathbb{1}$	0
5, 6	001	15	0	0	1
5, 6	001	$1\bar{1}$	$\mathbb{1}$	$\mathbb{1}$	1
5, 6	001	$\bar{5}5$	$\mathbb{1}$	$\mathbb{1}$	1
5, 6	001	$\bar{5}\bar{1}$	0	0	1
5, 8	001	35	0	0	0
5, 8	001	$3\bar{1}$	$\mathbb{1}$	$\mathbb{1}$	0
5, 8	001	$\bar{3}5$	$\mathbb{1}$	$\mathbb{1}$	0
5, 8	001	$\bar{3}\bar{1}$	0	0	0
5,10	001	55	0	0	1
5,10	001	$5\bar{1}$	$\mathbb{1}$	$\mathbb{1}$	1
5,10	001	$\bar{1}5$	$\mathbb{1}$	$\mathbb{1}$	1
5,10	001	$\bar{1}\bar{1}$	0	0	1
Space group #176 : $P6_3/m$					
$\mathbb{Z}_{12}$	weak	$m^{001}_{(6)}$	i	2^{001}_1	6^{001}_3
0	000	00	0	0	0

Continued on next page

Supplementary Table 7 – continued

SI	Invariants				
0	000	60	0	$\mathbb{1}$	$\mathbb{1}$
2	000	20	$\mathbb{1}$	0	0
2	000	$\bar{4}0$	$\mathbb{1}$	$\mathbb{1}$	$\mathbb{1}$
4	000	40	0	0	0
4	000	$\bar{2}0$	0	$\mathbb{1}$	$\mathbb{1}$
6	000	00	$\mathbb{1}$	$\mathbb{1}$	$\mathbb{1}$
6	000	60	$\mathbb{1}$	0	0
8	000	20	0	$\mathbb{1}$	$\mathbb{1}$
8	000	$\bar{4}0$	0	0	0
10	000	40	$\mathbb{1}$	$\mathbb{1}$	$\mathbb{1}$
10	000	$\bar{2}0$	$\mathbb{1}$	0	0

Space group #187 : $P\bar{6}m2$

$\mathbb{Z}_{3,3}$	weak	$m^{\bar{1}20}_{(2)}$	$m^{001}_{(3)}$	$g^{\bar{1}20}_{1\frac{1}{2}0}$	$2^{1\bar{1}0}$	$2_1^{1\bar{1}0}$
00	000	0	00	0	0	0
00	000	0	00	0	$\mathbb{1}$	$\mathbb{1}$
00	000	2	00	$\mathbb{1}$	0	0
00	000	2	00	$\mathbb{1}$	$\mathbb{1}$	$\mathbb{1}$
00	001	0	33	0	0	1
00	001	0	33	0	1	0
00	001	2	33	$\mathbb{1}$	0	1
00	001	2	33	$\mathbb{1}$	1	0
01	000	0	$0\bar{2}$	0	0	0
01	000	0	$0\bar{2}$	0	$\mathbb{1}$	$\mathbb{1}$
01	000	2	$0\bar{2}$	$\mathbb{1}$	0	0
01	000	2	$0\bar{2}$	$\mathbb{1}$	$\mathbb{1}$	$\mathbb{1}$
01	001	0	31	0	0	1
01	001	0	31	0	1	0
01	001	2	31	$\mathbb{1}$	0	1
01	001	2	31	$\mathbb{1}$	1	0
02	000	0	02	0	0	0
02	000	0	02	0	$\mathbb{1}$	$\mathbb{1}$
02	000	2	02	$\mathbb{1}$	0	0
02	000	2	02	$\mathbb{1}$	$\mathbb{1}$	$\mathbb{1}$
02	001	0	$3\bar{1}$	0	0	1
02	001	0	$3\bar{1}$	0	1	0
02	001	2	$3\bar{1}$	$\mathbb{1}$	0	1
02	001	2	$3\bar{1}$	$\mathbb{1}$	1	0
10	000	0	$\bar{2}0$	0	0	0
10	000	0	$\bar{2}0$	0	$\mathbb{1}$	$\mathbb{1}$
10	000	2	$\bar{2}0$	$\mathbb{1}$	0	0
10	000	2	$\bar{2}0$	$\mathbb{1}$	$\mathbb{1}$	$\mathbb{1}$
10	001	0	13	0	0	1
10	001	0	13	0	1	0
10	001	2	13	$\mathbb{1}$	0	1
10	001	2	13	$\mathbb{1}$	1	0
11	000	0	$\bar{2}\bar{2}$	0	0	0
11	000	0	$\bar{2}\bar{2}$	0	$\mathbb{1}$	$\mathbb{1}$
11	000	2	$\bar{2}\bar{2}$	$\mathbb{1}$	0	0
11	000	2	$\bar{2}\bar{2}$	$\mathbb{1}$	$\mathbb{1}$	$\mathbb{1}$
11	001	0	11	0	0	1
11	001	0	11	0	1	0
11	001	2	11	$\mathbb{1}$	0	1
11	001	2	11	$\mathbb{1}$	1	0
12	000	0	$\bar{2}2$	0	0	0
12	000	0	$\bar{2}2$	0	$\mathbb{1}$	$\mathbb{1}$
12	000	2	$\bar{2}2$	$\mathbb{1}$	0	0
12	000	2	$\bar{2}2$	$\mathbb{1}$	$\mathbb{1}$	$\mathbb{1}$
12	001	0	$1\bar{1}$	0	0	1
12	001	0	$1\bar{1}$	0	1	0
12	001	2	$1\bar{1}$	$\mathbb{1}$	0	1

Continued on next page

Supplementary Table 7 – continued

**SI**	**Invariants**					
12	001	2	$1\bar{1}$	$\mathbb{1}$	1	0
20	000	0	20	0	0	0
20	000	0	20	0	$\mathbb{1}$	$\mathbb{1}$
20	000	2	20	$\mathbb{1}$	0	0
20	000	2	20	$\mathbb{1}$	$\mathbb{1}$	$\mathbb{1}$
20	001	0	$\bar{1}3$	0	0	1
20	001	0	$\bar{1}3$	0	1	0
20	001	2	$\bar{1}3$	$\mathbb{1}$	0	1
20	001	2	$\bar{1}3$	$\mathbb{1}$	1	0
21	000	0	$2\bar{2}$	0	0	0
21	000	0	$2\bar{2}$	0	$\mathbb{1}$	$\mathbb{1}$
21	000	2	$2\bar{2}$	$\mathbb{1}$	0	0
21	000	2	$2\bar{2}$	$\mathbb{1}$	$\mathbb{1}$	$\mathbb{1}$
21	001	0	$\bar{1}1$	0	0	1
21	001	0	$\bar{1}1$	0	1	0
21	001	2	$\bar{1}1$	$\mathbb{1}$	0	1
21	001	2	$\bar{1}1$	$\mathbb{1}$	1	0
22	000	0	22	0	0	0
22	000	0	22	0	$\mathbb{1}$	$\mathbb{1}$
22	000	2	22	$\mathbb{1}$	0	0
22	000	2	22	$\mathbb{1}$	$\mathbb{1}$	$\mathbb{1}$
22	001	0	$\bar{1}\bar{1}$	0	0	1
22	001	0	$\bar{1}\bar{1}$	0	1	0
22	001	2	$\bar{1}\bar{1}$	$\mathbb{1}$	0	1
22	001	2	$\bar{1}\bar{1}$	$\mathbb{1}$	1	0
Space group #188 : $P\bar{6}c2$						
$\mathbb{Z}_3$	weak	$m^{001}_{(3)}$	$g^{\bar{1}20}_{00\frac{1}{2}}$	$g^{\bar{1}20}_{1\frac{1}{2}\frac{1}{2}}$	$2^{1\bar{1}0}$	$2^{1\bar{1}0}_{1}$
0	000	00	0	0	0	0
0	000	00	0	0	$\mathbb{1}$	$\mathbb{1}$
0	000	00	$\mathbb{1}$	$\mathbb{1}$	0	0
0	000	00	$\mathbb{1}$	$\mathbb{1}$	$\mathbb{1}$	$\mathbb{1}$
1	000	$\bar{2}0$	0	0	0	0
1	000	$\bar{2}0$	0	0	$\mathbb{1}$	$\mathbb{1}$
1	000	$\bar{2}0$	$\mathbb{1}$	$\mathbb{1}$	0	0
1	000	$\bar{2}0$	$\mathbb{1}$	$\mathbb{1}$	$\mathbb{1}$	$\mathbb{1}$
2	000	20	0	0	0	0
2	000	20	0	0	$\mathbb{1}$	$\mathbb{1}$
2	000	20	$\mathbb{1}$	$\mathbb{1}$	0	0
2	000	20	$\mathbb{1}$	$\mathbb{1}$	$\mathbb{1}$	$\mathbb{1}$
Space group #189 : $P\bar{6}2m$						
$\mathbb{Z}_{3,3}$	weak	$m^{001}_{(3)}$	$m^{1\bar{1}0}_{(2)}$	$g^{1\bar{1}0}_{\frac{1}{2}\frac{1}{2}0}$	2^{010}	2^{010}_{1}
00	000	00	0	0	0	0
00	000	00	0	0	$\mathbb{1}$	$\mathbb{1}$
00	000	00	2	$\mathbb{1}$	0	0
00	000	00	2	$\mathbb{1}$	$\mathbb{1}$	$\mathbb{1}$
00	001	33	0	0	0	1
00	001	33	0	0	1	0
00	001	33	2	$\mathbb{1}$	0	1
00	001	33	2	$\mathbb{1}$	1	0
01	000	$0\bar{2}$	0	0	0	0
01	000	$0\bar{2}$	0	0	$\mathbb{1}$	$\mathbb{1}$
01	000	$0\bar{2}$	2	$\mathbb{1}$	0	0
01	000	$0\bar{2}$	2	$\mathbb{1}$	$\mathbb{1}$	$\mathbb{1}$
01	001	31	0	0	0	1
01	001	31	0	0	1	0
01	001	31	2	$\mathbb{1}$	0	1
01	001	31	2	$\mathbb{1}$	1	0
02	000	02	0	0	0	0
02	000	02	0	0	$\mathbb{1}$	$\mathbb{1}$

Continued on next page

Supplementary Table 7 – continued

SI	Invariants					
02	000	02	2	$\mathbb{1}$	0	0
02	000	02	2	$\mathbb{1}$	$\mathbb{1}$	$\mathbb{1}$
02	001	$3\bar{1}$	0	0	0	1
02	001	$3\bar{1}$	0	0	1	0
02	001	$3\bar{1}$	2	$\mathbb{1}$	0	1
02	001	$3\bar{1}$	2	$\mathbb{1}$	1	0
10	000	$\bar{2}0$	0	0	0	0
10	000	$\bar{2}0$	0	0	$\mathbb{1}$	$\mathbb{1}$
10	000	$\bar{2}0$	2	$\mathbb{1}$	0	0
10	000	$\bar{2}0$	2	$\mathbb{1}$	$\mathbb{1}$	$\mathbb{1}$
10	001	13	0	0	0	1
10	001	13	0	0	1	0
10	001	13	2	$\mathbb{1}$	0	1
10	001	13	2	$\mathbb{1}$	1	0
11	000	$\bar{2}\bar{2}$	0	0	0	0
11	000	$\bar{2}\bar{2}$	0	0	$\mathbb{1}$	$\mathbb{1}$
11	000	$\bar{2}\bar{2}$	2	$\mathbb{1}$	0	0
11	000	$\bar{2}\bar{2}$	2	$\mathbb{1}$	$\mathbb{1}$	$\mathbb{1}$
11	001	11	0	0	0	1
11	001	11	0	0	1	0
11	001	11	2	$\mathbb{1}$	0	1
11	001	11	2	$\mathbb{1}$	1	0
12	000	$\bar{2}2$	0	0	0	0
12	000	$\bar{2}2$	0	0	$\mathbb{1}$	$\mathbb{1}$
12	000	$\bar{2}2$	2	$\mathbb{1}$	0	0
12	000	$\bar{2}2$	2	$\mathbb{1}$	$\mathbb{1}$	$\mathbb{1}$
12	001	$1\bar{1}$	0	0	0	1
12	001	$1\bar{1}$	0	0	1	0
12	001	$1\bar{1}$	2	$\mathbb{1}$	0	1
12	001	$1\bar{1}$	2	$\mathbb{1}$	1	0
20	000	20	0	0	0	0
20	000	20	0	0	$\mathbb{1}$	$\mathbb{1}$
20	000	20	2	$\mathbb{1}$	0	0
20	000	20	2	$\mathbb{1}$	$\mathbb{1}$	$\mathbb{1}$
20	001	$\bar{1}3$	0	0	0	1
20	001	$\bar{1}3$	0	0	1	0
20	001	$\bar{1}3$	2	$\mathbb{1}$	0	1
20	001	$\bar{1}3$	2	$\mathbb{1}$	1	0
21	000	$2\bar{2}$	0	0	0	0
21	000	$2\bar{2}$	0	0	$\mathbb{1}$	$\mathbb{1}$
21	000	$2\bar{2}$	2	$\mathbb{1}$	0	0
21	000	$2\bar{2}$	2	$\mathbb{1}$	$\mathbb{1}$	$\mathbb{1}$
21	001	$\bar{1}1$	0	0	0	1
21	001	$\bar{1}1$	0	0	1	0
21	001	$\bar{1}1$	2	$\mathbb{1}$	0	1
21	001	$\bar{1}1$	2	$\mathbb{1}$	1	0
22	000	22	0	0	0	0
22	000	22	0	0	$\mathbb{1}$	$\mathbb{1}$
22	000	22	2	$\mathbb{1}$	0	0
22	000	22	2	$\mathbb{1}$	$\mathbb{1}$	$\mathbb{1}$
22	001	$\bar{1}\bar{1}$	0	0	0	1
22	001	$\bar{1}\bar{1}$	0	0	1	0
22	001	$\bar{1}\bar{1}$	2	$\mathbb{1}$	0	1
22	001	$\bar{1}\bar{1}$	2	$\mathbb{1}$	1	0
Space group #190 : $P\bar{6}2c$						
$\mathbb{Z}_3$	weak	$m^{001}_{(3)}$	$g^{1\bar{1}0}_{00\frac{1}{2}}$	$g^{1\bar{1}0}_{\frac{1}{2}\frac{1}{2}\frac{1}{2}}$	2^{010}	2^{010}_1
0	000	00	0	0	0	0
0	000	00	0	0	$\mathbb{1}$	$\mathbb{1}$
0	000	00	$\mathbb{1}$	$\mathbb{1}$	0	0
0	000	00	$\mathbb{1}$	$\mathbb{1}$	$\mathbb{1}$	$\mathbb{1}$

Continued on next page

Supplementary Table 7 – continued

**SI**	**Invariants**					
1	000	$\bar{2}0$	0	0	0	0
1	000	$\bar{2}0$	0	0	$\mathbb{1}$	$\mathbb{1}$
1	000	$\bar{2}0$	$\mathbb{1}$	$\mathbb{1}$	0	0
1	000	$\bar{2}0$	$\mathbb{1}$	$\mathbb{1}$	$\mathbb{1}$	$\mathbb{1}$
2	000	20	0	0	0	0
2	000	20	0	0	$\mathbb{1}$	$\mathbb{1}$
2	000	20	$\mathbb{1}$	$\mathbb{1}$	0	0
2	000	20	$\mathbb{1}$	$\mathbb{1}$	$\mathbb{1}$	$\mathbb{1}$

Space group #191 : $P6/mmm$

$\mathbb{Z}_{6,12}$	weak	$m^{\bar{1}20}_{(2)}$	$m^{001}_{(6)}$	$m^{010}_{(2)}$	$g^{\bar{1}20}_{1\frac{1}{2}0}$	$g^{010}_{\frac{1}{2}00}$	2^{001}	2^{010}	$2^{1\bar{1}0}$	6^{001}	i	2^{010}_{1}	$2^{1\bar{1}0}_{1}$
0, 0	000	0	00	0	0	0	0	0	0	0	0	0	0
0, 0	000	0	06	2	0	$\mathbb{1}$	$\mathbb{1}$	0	$\mathbb{1}$	$\mathbb{1}$	0	0	$\mathbb{1}$
0, 0	000	0	60	2	0	$\mathbb{1}$	$\mathbb{1}$	0	$\mathbb{1}$	$\mathbb{1}$	0	0	$\mathbb{1}$
0, 0	000	0	66	0	0	0	0	0	0	0	0	0	0
0, 0	000	2	00	2	$\mathbb{1}$	$\mathbb{1}$	0	$\mathbb{1}$	$\mathbb{1}$	0	0	$\mathbb{1}$	$\mathbb{1}$
0, 0	000	2	06	0	$\mathbb{1}$	0	$\mathbb{1}$	$\mathbb{1}$	0	$\mathbb{1}$	0	$\mathbb{1}$	0
0, 0	000	2	60	0	$\mathbb{1}$	0	$\mathbb{1}$	$\mathbb{1}$	0	$\mathbb{1}$	0	$\mathbb{1}$	0
0, 0	000	2	66	2	$\mathbb{1}$	$\mathbb{1}$	0	$\mathbb{1}$	$\mathbb{1}$	0	0	$\mathbb{1}$	$\mathbb{1}$
0, 2	000	0	20	0	0	0	0	$\mathbb{1}$	$\mathbb{1}$	0	$\mathbb{1}$	$\mathbb{1}$	$\mathbb{1}$
0, 2	000	0	26	2	0	$\mathbb{1}$	$\mathbb{1}$	$\mathbb{1}$	0	$\mathbb{1}$	$\mathbb{1}$	$\mathbb{1}$	0
0, 2	000	0	$\bar{4}0$	2	0	$\mathbb{1}$	$\mathbb{1}$	$\mathbb{1}$	0	$\mathbb{1}$	$\mathbb{1}$	$\mathbb{1}$	0
0, 2	000	0	$\bar{4}6$	0	0	0	0	$\mathbb{1}$	$\mathbb{1}$	0	$\mathbb{1}$	$\mathbb{1}$	$\mathbb{1}$
0, 2	000	2	20	2	$\mathbb{1}$	$\mathbb{1}$	0	0	0	0	$\mathbb{1}$	0	0
0, 2	000	2	26	0	$\mathbb{1}$	0	$\mathbb{1}$	0	$\mathbb{1}$	$\mathbb{1}$	$\mathbb{1}$	0	$\mathbb{1}$
0, 2	000	2	$\bar{4}0$	0	$\mathbb{1}$	0	$\mathbb{1}$	0	$\mathbb{1}$	$\mathbb{1}$	$\mathbb{1}$	0	$\mathbb{1}$
0, 2	000	2	$\bar{4}6$	2	$\mathbb{1}$	$\mathbb{1}$	0	0	0	0	$\mathbb{1}$	0	0
0, 4	000	0	40	0	0	0	0	0	0	0	0	0	0
0, 4	000	0	46	2	0	$\mathbb{1}$	$\mathbb{1}$	0	$\mathbb{1}$	$\mathbb{1}$	0	0	$\mathbb{1}$
0, 4	000	0	$\bar{2}0$	2	0	$\mathbb{1}$	$\mathbb{1}$	0	$\mathbb{1}$	$\mathbb{1}$	0	0	$\mathbb{1}$
0, 4	000	0	$\bar{2}6$	0	0	0	0	0	0	0	0	0	0
0, 4	000	2	40	2	$\mathbb{1}$	$\mathbb{1}$	0	$\mathbb{1}$	$\mathbb{1}$	0	0	$\mathbb{1}$	$\mathbb{1}$
0, 4	000	2	46	0	$\mathbb{1}$	0	$\mathbb{1}$	$\mathbb{1}$	0	$\mathbb{1}$	0	$\mathbb{1}$	0
0, 4	000	2	$\bar{2}0$	0	$\mathbb{1}$	0	$\mathbb{1}$	$\mathbb{1}$	0	$\mathbb{1}$	0	$\mathbb{1}$	0
0, 4	000	2	$\bar{2}6$	2	$\mathbb{1}$	$\mathbb{1}$	0	$\mathbb{1}$	$\mathbb{1}$	0	0	$\mathbb{1}$	$\mathbb{1}$
0, 6	000	0	00	2	0	$\mathbb{1}$	$\mathbb{1}$	$\mathbb{1}$	0	$\mathbb{1}$	$\mathbb{1}$	$\mathbb{1}$	0
0, 6	000	0	06	0	0	0	0	$\mathbb{1}$	$\mathbb{1}$	0	$\mathbb{1}$	$\mathbb{1}$	$\mathbb{1}$
0, 6	000	0	60	0	0	0	0	$\mathbb{1}$	$\mathbb{1}$	0	$\mathbb{1}$	$\mathbb{1}$	$\mathbb{1}$
0, 6	000	0	66	2	0	$\mathbb{1}$	$\mathbb{1}$	$\mathbb{1}$	0	$\mathbb{1}$	$\mathbb{1}$	$\mathbb{1}$	0
0, 6	000	2	00	0	$\mathbb{1}$	0	$\mathbb{1}$	0	$\mathbb{1}$	$\mathbb{1}$	$\mathbb{1}$	0	$\mathbb{1}$
0, 6	000	2	06	2	$\mathbb{1}$	$\mathbb{1}$	0	0	0	0	$\mathbb{1}$	0	0
0, 6	000	2	60	2	$\mathbb{1}$	$\mathbb{1}$	0	0	0	0	$\mathbb{1}$	0	0
0, 6	000	2	66	0	$\mathbb{1}$	0	$\mathbb{1}$	0	$\mathbb{1}$	$\mathbb{1}$	$\mathbb{1}$	0	$\mathbb{1}$
0, 8	000	0	20	2	0	$\mathbb{1}$	$\mathbb{1}$	0	$\mathbb{1}$	$\mathbb{1}$	0	0	$\mathbb{1}$
0, 8	000	0	26	0	0	0	0	0	0	0	0	0	0
0, 8	000	0	$\bar{4}0$	0	0	0	0	0	0	0	0	0	0
0, 8	000	0	$\bar{4}6$	2	0	$\mathbb{1}$	$\mathbb{1}$	0	$\mathbb{1}$	$\mathbb{1}$	0	0	$\mathbb{1}$
0, 8	000	2	20	0	$\mathbb{1}$	0	$\mathbb{1}$	$\mathbb{1}$	0	$\mathbb{1}$	0	$\mathbb{1}$	0
0, 8	000	2	26	2	$\mathbb{1}$	$\mathbb{1}$	0	$\mathbb{1}$	$\mathbb{1}$	0	0	$\mathbb{1}$	$\mathbb{1}$
0, 8	000	2	$\bar{4}0$	2	$\mathbb{1}$	$\mathbb{1}$	0	$\mathbb{1}$	$\mathbb{1}$	0	0	$\mathbb{1}$	$\mathbb{1}$
0, 8	000	2	$\bar{4}6$	0	$\mathbb{1}$	0	$\mathbb{1}$	$\mathbb{1}$	0	$\mathbb{1}$	0	$\mathbb{1}$	0
0,10	000	0	40	2	0	$\mathbb{1}$	$\mathbb{1}$	$\mathbb{1}$	0	$\mathbb{1}$	$\mathbb{1}$	$\mathbb{1}$	0
0,10	000	0	46	0	0	0	0	$\mathbb{1}$	$\mathbb{1}$	0	$\mathbb{1}$	$\mathbb{1}$	$\mathbb{1}$
0,10	000	0	$\bar{2}0$	0	0	0	0	$\mathbb{1}$	$\mathbb{1}$	0	$\mathbb{1}$	$\mathbb{1}$	$\mathbb{1}$
0,10	000	0	$\bar{2}6$	2	0	$\mathbb{1}$	$\mathbb{1}$	$\mathbb{1}$	0	$\mathbb{1}$	$\mathbb{1}$	$\mathbb{1}$	0
0,10	000	2	40	0	$\mathbb{1}$	0	$\mathbb{1}$	0	$\mathbb{1}$	$\mathbb{1}$	$\mathbb{1}$	0	$\mathbb{1}$
0,10	000	2	46	2	$\mathbb{1}$	$\mathbb{1}$	0	0	0	0	$\mathbb{1}$	0	0
0,10	000	2	$\bar{2}0$	2	$\mathbb{1}$	$\mathbb{1}$	0	0	0	0	$\mathbb{1}$	0	0
0,10	000	2	$\bar{2}6$	0	$\mathbb{1}$	0	$\mathbb{1}$	0	$\mathbb{1}$	$\mathbb{1}$	$\mathbb{1}$	0	$\mathbb{1}$
1, 0	001	0	51	2	0	$\mathbb{1}$	$\mathbb{1}$	0	1	$\mathbb{1}$	0	1	0
1, 0	001	0	$5\bar{5}$	0	0	0	0	0	0	0	0	1	1

Continued on next page

Supplementary Table 7 – continued

SI	Invariants												
1, 0	001	0	$\bar{1}1$	0	0	0	0	0	0	0	0	1	1
1, 0	001	0	$\bar{1}\bar{5}$	2	0	$\mathbb{1}$	$\mathbb{1}$	0	1	$\mathbb{1}$	0	1	0
1, 0	001	2	51	0	$\mathbb{1}$	0	$\mathbb{1}$	1	0	$\mathbb{1}$	0	0	1
1, 0	001	2	$5\bar{5}$	2	$\mathbb{1}$	$\mathbb{1}$	0	1	1	0	0	0	0
1, 0	001	2	$\bar{1}1$	2	$\mathbb{1}$	$\mathbb{1}$	0	1	1	0	0	0	0
1, 0	001	2	$\bar{1}\bar{5}$	0	$\mathbb{1}$	0	$\mathbb{1}$	1	0	$\mathbb{1}$	0	0	1
1, 2	001	0	11	0	0	0	0	1	1	0	1	0	0
1, 2	001	0	$1\bar{5}$	2	0	$\mathbb{1}$	$\mathbb{1}$	1	0	$\mathbb{1}$	1	0	1
1, 2	001	0	$\bar{5}1$	2	0	$\mathbb{1}$	$\mathbb{1}$	1	0	$\mathbb{1}$	1	0	1
1, 2	001	0	$\bar{5}\bar{5}$	0	0	0	0	1	1	0	1	0	0
1, 2	001	2	11	2	$\mathbb{1}$	$\mathbb{1}$	0	0	0	0	1	1	1
1, 2	001	2	$1\bar{5}$	0	$\mathbb{1}$	0	$\mathbb{1}$	0	1	$\mathbb{1}$	1	1	0
1, 2	001	2	$\bar{5}1$	0	$\mathbb{1}$	0	$\mathbb{1}$	0	1	$\mathbb{1}$	1	1	0
1, 2	001	2	$\bar{5}\bar{5}$	2	$\mathbb{1}$	$\mathbb{1}$	0	0	0	0	1	1	1
1, 4	001	0	31	0	0	0	0	0	0	0	0	1	1
1, 4	001	0	$3\bar{5}$	2	0	$\mathbb{1}$	$\mathbb{1}$	0	1	$\mathbb{1}$	0	1	0
1, 4	001	0	$\bar{3}1$	2	0	$\mathbb{1}$	$\mathbb{1}$	0	1	$\mathbb{1}$	0	1	0
1, 4	001	0	$\bar{3}\bar{5}$	0	0	0	0	0	0	0	0	1	1
1, 4	001	2	31	2	$\mathbb{1}$	$\mathbb{1}$	0	1	1	0	0	0	0
1, 4	001	2	$3\bar{5}$	0	$\mathbb{1}$	0	$\mathbb{1}$	1	0	$\mathbb{1}$	0	0	1
1, 4	001	2	$\bar{3}1$	0	$\mathbb{1}$	0	$\mathbb{1}$	1	0	$\mathbb{1}$	0	0	1
1, 4	001	2	$\bar{3}\bar{5}$	2	$\mathbb{1}$	$\mathbb{1}$	0	1	1	0	0	0	0
1, 6	001	0	51	0	0	0	0	1	1	0	1	0	0
1, 6	001	0	$5\bar{5}$	2	0	$\mathbb{1}$	$\mathbb{1}$	1	0	$\mathbb{1}$	1	0	1
1, 6	001	0	$\bar{1}1$	2	0	$\mathbb{1}$	$\mathbb{1}$	1	0	$\mathbb{1}$	1	0	1
1, 6	001	0	$\bar{1}\bar{5}$	0	0	0	0	1	1	0	1	0	0
1, 6	001	2	51	2	$\mathbb{1}$	$\mathbb{1}$	0	0	0	0	1	1	1
1, 6	001	2	$5\bar{5}$	0	$\mathbb{1}$	0	$\mathbb{1}$	0	1	$\mathbb{1}$	1	1	0
1, 6	001	2	$\bar{1}1$	0	$\mathbb{1}$	0	$\mathbb{1}$	0	1	$\mathbb{1}$	1	1	0
1, 6	001	2	$\bar{1}\bar{5}$	2	$\mathbb{1}$	$\mathbb{1}$	0	0	0	0	1	1	1
1, 8	001	0	11	2	0	$\mathbb{1}$	$\mathbb{1}$	0	1	$\mathbb{1}$	0	1	0
1, 8	001	0	$1\bar{5}$	0	0	0	0	0	0	0	0	1	1
1, 8	001	0	$\bar{5}1$	0	0	0	0	0	0	0	0	1	1
1, 8	001	0	$\bar{5}\bar{5}$	2	0	$\mathbb{1}$	$\mathbb{1}$	0	1	$\mathbb{1}$	0	1	0
1, 8	001	2	11	0	$\mathbb{1}$	0	$\mathbb{1}$	1	0	$\mathbb{1}$	0	0	1
1, 8	001	2	$1\bar{5}$	2	$\mathbb{1}$	$\mathbb{1}$	0	1	1	0	0	0	0
1, 8	001	2	$\bar{5}1$	2	$\mathbb{1}$	$\mathbb{1}$	0	1	1	0	0	0	0
1, 8	001	2	$\bar{5}\bar{5}$	0	$\mathbb{1}$	0	$\mathbb{1}$	1	0	$\mathbb{1}$	0	0	1
1,10	001	0	31	2	0	$\mathbb{1}$	$\mathbb{1}$	1	0	$\mathbb{1}$	1	0	1
1,10	001	0	$3\bar{5}$	0	0	0	0	1	1	0	1	0	0
1,10	001	0	$\bar{3}1$	0	0	0	0	1	1	0	1	0	0
1,10	001	0	$\bar{3}\bar{5}$	2	0	$\mathbb{1}$	$\mathbb{1}$	1	0	$\mathbb{1}$	1	0	1
1,10	001	2	31	0	$\mathbb{1}$	0	$\mathbb{1}$	0	1	$\mathbb{1}$	1	1	0
1,10	001	2	$3\bar{5}$	2	$\mathbb{1}$	$\mathbb{1}$	0	0	0	0	1	1	1
1,10	001	2	$\bar{3}1$	2	$\mathbb{1}$	$\mathbb{1}$	0	0	0	0	1	1	1
1,10	001	2	$\bar{3}\bar{5}$	0	$\mathbb{1}$	0	$\mathbb{1}$	0	1	$\mathbb{1}$	1	1	0
2, 0	000	0	42	2	0	$\mathbb{1}$	$\mathbb{1}$	0	$\mathbb{1}$	$\mathbb{1}$	0	0	$\mathbb{1}$
2, 0	000	0	$4\bar{4}$	0	0	0	0	0	0	0	0	0	0
2, 0	000	0	$\bar{2}2$	0	0	0	0	0	0	0	0	0	0
2, 0	000	0	$\bar{2}\bar{4}$	2	0	$\mathbb{1}$	$\mathbb{1}$	0	$\mathbb{1}$	$\mathbb{1}$	0	0	$\mathbb{1}$
2, 0	000	2	42	0	$\mathbb{1}$	0	$\mathbb{1}$	$\mathbb{1}$	0	$\mathbb{1}$	0	$\mathbb{1}$	0
2, 0	000	2	$4\bar{4}$	2	$\mathbb{1}$	$\mathbb{1}$	0	$\mathbb{1}$	$\mathbb{1}$	0	0	$\mathbb{1}$	$\mathbb{1}$
2, 0	000	2	$\bar{2}2$	2	$\mathbb{1}$	$\mathbb{1}$	0	$\mathbb{1}$	$\mathbb{1}$	0	0	$\mathbb{1}$	$\mathbb{1}$
2, 0	000	2	$\bar{2}\bar{4}$	0	$\mathbb{1}$	0	$\mathbb{1}$	$\mathbb{1}$	0	$\mathbb{1}$	0	$\mathbb{1}$	0
2, 2	000	0	02	0	0	0	0	$\mathbb{1}$	$\mathbb{1}$	0	$\mathbb{1}$	$\mathbb{1}$	$\mathbb{1}$
2, 2	000	0	$0\bar{4}$	2	0	$\mathbb{1}$	$\mathbb{1}$	$\mathbb{1}$	0	$\mathbb{1}$	$\mathbb{1}$	$\mathbb{1}$	0
2, 2	000	0	62	2	0	$\mathbb{1}$	$\mathbb{1}$	$\mathbb{1}$	0	$\mathbb{1}$	$\mathbb{1}$	$\mathbb{1}$	0
2, 2	000	0	$6\bar{4}$	0	0	0	0	$\mathbb{1}$	$\mathbb{1}$	0	$\mathbb{1}$	$\mathbb{1}$	$\mathbb{1}$
2, 2	000	2	02	2	$\mathbb{1}$	$\mathbb{1}$	0	0	0	0	$\mathbb{1}$	0	0
2, 2	000	2	$0\bar{4}$	0	$\mathbb{1}$	0	$\mathbb{1}$	0	$\mathbb{1}$	$\mathbb{1}$	$\mathbb{1}$	0	$\mathbb{1}$
2, 2	000	2	62	0	$\mathbb{1}$	0	$\mathbb{1}$	0	$\mathbb{1}$	$\mathbb{1}$	$\mathbb{1}$	0	$\mathbb{1}$

Continued on next page

Supplementary Table 7 – continued

SI	Invariants												
2, 2	000	2	$6\bar{4}$	2	$\mathbb{1}$	$\mathbb{1}$	0	0	0	0	$\mathbb{1}$	0	0
2, 4	000	0	22	0	0	0	0	0	0	0	0	0	0
2, 4	000	0	$2\bar{4}$	2	0	$\mathbb{1}$	$\mathbb{1}$	0	$\mathbb{1}$	$\mathbb{1}$	0	0	$\mathbb{1}$
2, 4	000	0	$\bar{4}2$	2	0	$\mathbb{1}$	$\mathbb{1}$	0	$\mathbb{1}$	$\mathbb{1}$	0	0	$\mathbb{1}$
2, 4	000	0	$\bar{4}\bar{4}$	0	0	0	0	0	0	0	0	0	0
2, 4	000	2	22	2	$\mathbb{1}$	$\mathbb{1}$	0	$\mathbb{1}$	$\mathbb{1}$	0	0	$\mathbb{1}$	$\mathbb{1}$
2, 4	000	2	$2\bar{4}$	0	$\mathbb{1}$	0	$\mathbb{1}$	$\mathbb{1}$	0	$\mathbb{1}$	0	$\mathbb{1}$	0
2, 4	000	2	$\bar{4}2$	0	$\mathbb{1}$	0	$\mathbb{1}$	$\mathbb{1}$	0	$\mathbb{1}$	0	$\mathbb{1}$	0
2, 4	000	2	$\bar{4}\bar{4}$	2	$\mathbb{1}$	$\mathbb{1}$	0	$\mathbb{1}$	$\mathbb{1}$	0	0	$\mathbb{1}$	$\mathbb{1}$
2, 6	000	0	42	0	0	0	0	$\mathbb{1}$	$\mathbb{1}$	0	$\mathbb{1}$	$\mathbb{1}$	$\mathbb{1}$
2, 6	000	0	$4\bar{4}$	2	0	$\mathbb{1}$	$\mathbb{1}$	$\mathbb{1}$	0	$\mathbb{1}$	$\mathbb{1}$	$\mathbb{1}$	0
2, 6	000	0	$\bar{2}2$	2	0	$\mathbb{1}$	$\mathbb{1}$	$\mathbb{1}$	0	$\mathbb{1}$	$\mathbb{1}$	$\mathbb{1}$	0
2, 6	000	0	$\bar{2}\bar{4}$	0	0	0	0	$\mathbb{1}$	$\mathbb{1}$	0	$\mathbb{1}$	$\mathbb{1}$	$\mathbb{1}$
2, 6	000	2	42	2	$\mathbb{1}$	$\mathbb{1}$	0	0	0	0	$\mathbb{1}$	0	0
2, 6	000	2	$4\bar{4}$	0	$\mathbb{1}$	0	$\mathbb{1}$	0	$\mathbb{1}$	$\mathbb{1}$	$\mathbb{1}$	0	$\mathbb{1}$
2, 6	000	2	$\bar{2}2$	0	$\mathbb{1}$	0	$\mathbb{1}$	0	$\mathbb{1}$	$\mathbb{1}$	$\mathbb{1}$	0	$\mathbb{1}$
2, 6	000	2	$\bar{2}\bar{4}$	2	$\mathbb{1}$	$\mathbb{1}$	0	0	0	0	$\mathbb{1}$	0	0
2, 8	000	0	02	2	0	$\mathbb{1}$	$\mathbb{1}$	0	$\mathbb{1}$	$\mathbb{1}$	0	0	$\mathbb{1}$
2, 8	000	0	$0\bar{4}$	0	0	0	0	0	0	0	0	0	0
2, 8	000	0	62	0	0	0	0	0	0	0	0	0	0
2, 8	000	0	$6\bar{4}$	2	0	$\mathbb{1}$	$\mathbb{1}$	0	$\mathbb{1}$	$\mathbb{1}$	0	0	$\mathbb{1}$
2, 8	000	2	02	0	$\mathbb{1}$	0	$\mathbb{1}$	$\mathbb{1}$	0	$\mathbb{1}$	0	$\mathbb{1}$	0
2, 8	000	2	$0\bar{4}$	2	$\mathbb{1}$	$\mathbb{1}$	0	$\mathbb{1}$	$\mathbb{1}$	0	0	$\mathbb{1}$	$\mathbb{1}$
2, 8	000	2	62	2	$\mathbb{1}$	$\mathbb{1}$	0	$\mathbb{1}$	$\mathbb{1}$	0	0	$\mathbb{1}$	$\mathbb{1}$
2, 8	000	2	$6\bar{4}$	0	$\mathbb{1}$	0	$\mathbb{1}$	$\mathbb{1}$	0	$\mathbb{1}$	0	$\mathbb{1}$	0
2,10	000	0	22	2	0	$\mathbb{1}$	$\mathbb{1}$	$\mathbb{1}$	0	$\mathbb{1}$	$\mathbb{1}$	$\mathbb{1}$	0
2,10	000	0	$2\bar{4}$	0	0	0	0	$\mathbb{1}$	$\mathbb{1}$	0	$\mathbb{1}$	$\mathbb{1}$	$\mathbb{1}$
2,10	000	0	$\bar{4}2$	0	0	0	0	$\mathbb{1}$	$\mathbb{1}$	0	$\mathbb{1}$	$\mathbb{1}$	$\mathbb{1}$
2,10	000	0	$\bar{4}\bar{4}$	2	0	$\mathbb{1}$	$\mathbb{1}$	$\mathbb{1}$	0	$\mathbb{1}$	$\mathbb{1}$	$\mathbb{1}$	0
2,10	000	2	22	0	$\mathbb{1}$	0	$\mathbb{1}$	0	$\mathbb{1}$	$\mathbb{1}$	$\mathbb{1}$	0	$\mathbb{1}$
2,10	000	2	$2\bar{4}$	2	$\mathbb{1}$	$\mathbb{1}$	0	0	0	0	$\mathbb{1}$	0	0
2,10	000	2	$\bar{4}2$	2	$\mathbb{1}$	$\mathbb{1}$	0	0	0	0	$\mathbb{1}$	0	0
2,10	000	2	$\bar{4}\bar{4}$	0	$\mathbb{1}$	0	$\mathbb{1}$	0	$\mathbb{1}$	$\mathbb{1}$	$\mathbb{1}$	0	$\mathbb{1}$
3, 0	001	0	33	2	0	$\mathbb{1}$	$\mathbb{1}$	0	1	$\mathbb{1}$	0	1	0
3, 0	001	0	$3\bar{3}$	0	0	0	0	0	0	0	0	1	1
3, 0	001	0	$\bar{3}3$	0	0	0	0	0	0	0	0	1	1
3, 0	001	0	$\bar{3}\bar{3}$	2	0	$\mathbb{1}$	$\mathbb{1}$	0	1	$\mathbb{1}$	0	1	0
3, 0	001	2	33	0	$\mathbb{1}$	0	$\mathbb{1}$	1	0	$\mathbb{1}$	0	0	1
3, 0	001	2	$3\bar{3}$	2	$\mathbb{1}$	$\mathbb{1}$	0	1	1	0	0	0	0
3, 0	001	2	$\bar{3}3$	2	$\mathbb{1}$	$\mathbb{1}$	0	1	1	0	0	0	0
3, 0	001	2	$\bar{3}\bar{3}$	0	$\mathbb{1}$	0	$\mathbb{1}$	1	0	$\mathbb{1}$	0	0	1
3, 2	001	0	53	2	0	$\mathbb{1}$	$\mathbb{1}$	1	0	$\mathbb{1}$	1	0	1
3, 2	001	0	$5\bar{3}$	0	0	0	0	1	1	0	1	0	0
3, 2	001	0	$\bar{1}3$	0	0	0	0	1	1	0	1	0	0
3, 2	001	0	$\bar{1}\bar{3}$	2	0	$\mathbb{1}$	$\mathbb{1}$	1	0	$\mathbb{1}$	1	0	1
3, 2	001	2	53	0	$\mathbb{1}$	0	$\mathbb{1}$	0	1	$\mathbb{1}$	1	1	0
3, 2	001	2	$5\bar{3}$	2	$\mathbb{1}$	$\mathbb{1}$	0	0	0	0	1	1	1
3, 2	001	2	$\bar{1}3$	2	$\mathbb{1}$	$\mathbb{1}$	0	0	0	0	1	1	1
3, 2	001	2	$\bar{1}\bar{3}$	0	$\mathbb{1}$	0	$\mathbb{1}$	0	1	$\mathbb{1}$	1	1	0
3, 4	001	0	13	0	0	0	0	0	0	0	0	1	1
3, 4	001	0	$1\bar{3}$	2	0	$\mathbb{1}$	$\mathbb{1}$	0	1	$\mathbb{1}$	0	1	0
3, 4	001	0	$\bar{5}3$	2	0	$\mathbb{1}$	$\mathbb{1}$	0	1	$\mathbb{1}$	0	1	0
3, 4	001	0	$\bar{5}\bar{3}$	0	0	0	0	0	0	0	0	1	1
3, 4	001	2	13	2	$\mathbb{1}$	$\mathbb{1}$	0	1	1	0	0	0	0
3, 4	001	2	$1\bar{3}$	0	$\mathbb{1}$	0	$\mathbb{1}$	1	0	$\mathbb{1}$	0	0	1
3, 4	001	2	$\bar{5}3$	0	$\mathbb{1}$	0	$\mathbb{1}$	1	0	$\mathbb{1}$	0	0	1
3, 4	001	2	$\bar{5}\bar{3}$	2	$\mathbb{1}$	$\mathbb{1}$	0	1	1	0	0	0	0
3, 6	001	0	33	0	0	0	0	1	1	0	1	0	0
3, 6	001	0	$3\bar{3}$	2	0	$\mathbb{1}$	$\mathbb{1}$	1	0	$\mathbb{1}$	1	0	1
3, 6	001	0	$\bar{3}3$	2	0	$\mathbb{1}$	$\mathbb{1}$	1	0	$\mathbb{1}$	1	0	1
3, 6	001	0	$\bar{3}\bar{3}$	0	0	0	0	1	1	0	1	0	0

Continued on next page

Supplementary Table 7 – continued

SI	Invariants												
3, 6	001	2	33	2	$\mathbb{1}$	$\mathbb{1}$	0	0	0	0	1	1	1
3, 6	001	2	$3\bar{3}$	0	$\mathbb{1}$	0	$\mathbb{1}$	0	1	$\mathbb{1}$	1	1	0
3, 6	001	2	$\bar{3}3$	0	$\mathbb{1}$	0	$\mathbb{1}$	0	1	$\mathbb{1}$	1	1	0
3, 6	001	2	$\bar{3}\bar{3}$	2	$\mathbb{1}$	$\mathbb{1}$	0	0	0	0	1	1	1
3, 8	001	0	53	0	0	0	0	0	0	0	0	1	1
3, 8	001	0	$5\bar{3}$	2	0	$\mathbb{1}$	$\mathbb{1}$	0	1	$\mathbb{1}$	0	1	0
3, 8	001	0	$\bar{1}3$	2	0	$\mathbb{1}$	$\mathbb{1}$	0	1	$\mathbb{1}$	0	1	0
3, 8	001	0	$\bar{1}\bar{3}$	0	0	0	0	0	0	0	0	1	1
3, 8	001	2	53	2	$\mathbb{1}$	$\mathbb{1}$	0	1	1	0	0	0	0
3, 8	001	2	$5\bar{3}$	0	$\mathbb{1}$	0	$\mathbb{1}$	1	0	$\mathbb{1}$	0	0	1
3, 8	001	2	$\bar{1}3$	0	$\mathbb{1}$	0	$\mathbb{1}$	1	0	$\mathbb{1}$	0	0	1
3, 8	001	2	$\bar{1}\bar{3}$	2	$\mathbb{1}$	$\mathbb{1}$	0	1	1	0	0	0	0
3,10	001	0	13	2	0	$\mathbb{1}$	$\mathbb{1}$	1	0	$\mathbb{1}$	1	0	1
3,10	001	0	$1\bar{3}$	0	0	0	0	1	1	0	1	0	0
3,10	001	0	$\bar{5}3$	0	0	0	0	1	1	0	1	0	0
3,10	001	0	$\bar{5}\bar{3}$	2	0	$\mathbb{1}$	$\mathbb{1}$	1	0	$\mathbb{1}$	1	0	1
3,10	001	2	13	0	$\mathbb{1}$	0	$\mathbb{1}$	0	1	$\mathbb{1}$	1	1	0
3,10	001	2	$1\bar{3}$	2	$\mathbb{1}$	$\mathbb{1}$	0	0	0	0	1	1	1
3,10	001	2	$\bar{5}3$	2	$\mathbb{1}$	$\mathbb{1}$	0	0	0	0	1	1	1
3,10	001	2	$\bar{5}\bar{3}$	0	$\mathbb{1}$	0	$\mathbb{1}$	0	1	$\mathbb{1}$	1	1	0
4, 0	000	0	24	2	0	$\mathbb{1}$	$\mathbb{1}$	0	$\mathbb{1}$	$\mathbb{1}$	0	0	$\mathbb{1}$
4, 0	000	0	$2\bar{2}$	0	0	0	0	0	0	0	0	0	0
4, 0	000	0	$\bar{4}4$	0	0	0	0	0	0	0	0	0	0
4, 0	000	0	$\bar{4}\bar{2}$	2	0	$\mathbb{1}$	$\mathbb{1}$	0	$\mathbb{1}$	$\mathbb{1}$	0	0	$\mathbb{1}$
4, 0	000	2	24	0	$\mathbb{1}$	0	$\mathbb{1}$	$\mathbb{1}$	0	$\mathbb{1}$	0	$\mathbb{1}$	0
4, 0	000	2	$2\bar{2}$	2	$\mathbb{1}$	$\mathbb{1}$	0	$\mathbb{1}$	$\mathbb{1}$	0	0	$\mathbb{1}$	$\mathbb{1}$
4, 0	000	2	$\bar{4}4$	2	$\mathbb{1}$	$\mathbb{1}$	0	$\mathbb{1}$	$\mathbb{1}$	0	0	$\mathbb{1}$	$\mathbb{1}$
4, 0	000	2	$\bar{4}\bar{2}$	0	$\mathbb{1}$	0	$\mathbb{1}$	$\mathbb{1}$	0	$\mathbb{1}$	0	$\mathbb{1}$	0
4, 2	000	0	44	2	0	$\mathbb{1}$	$\mathbb{1}$	$\mathbb{1}$	0	$\mathbb{1}$	$\mathbb{1}$	$\mathbb{1}$	0
4, 2	000	0	$4\bar{2}$	0	0	0	0	$\mathbb{1}$	$\mathbb{1}$	0	$\mathbb{1}$	$\mathbb{1}$	$\mathbb{1}$
4, 2	000	0	$\bar{2}4$	0	0	0	0	$\mathbb{1}$	$\mathbb{1}$	0	$\mathbb{1}$	$\mathbb{1}$	$\mathbb{1}$
4, 2	000	0	$\bar{2}\bar{2}$	2	0	$\mathbb{1}$	$\mathbb{1}$	$\mathbb{1}$	0	$\mathbb{1}$	$\mathbb{1}$	$\mathbb{1}$	0
4, 2	000	2	44	0	$\mathbb{1}$	0	$\mathbb{1}$	0	$\mathbb{1}$	$\mathbb{1}$	$\mathbb{1}$	0	$\mathbb{1}$
4, 2	000	2	$4\bar{2}$	2	$\mathbb{1}$	$\mathbb{1}$	0	0	0	0	$\mathbb{1}$	0	0
4, 2	000	2	$\bar{2}4$	2	$\mathbb{1}$	$\mathbb{1}$	0	0	0	0	$\mathbb{1}$	0	0
4, 2	000	2	$\bar{2}\bar{2}$	0	$\mathbb{1}$	0	$\mathbb{1}$	0	$\mathbb{1}$	$\mathbb{1}$	$\mathbb{1}$	0	$\mathbb{1}$
4, 4	000	0	04	0	0	0	0	0	0	0	0	0	0
4, 4	000	0	$0\bar{2}$	2	0	$\mathbb{1}$	$\mathbb{1}$	0	$\mathbb{1}$	$\mathbb{1}$	0	0	$\mathbb{1}$
4, 4	000	0	64	2	0	$\mathbb{1}$	$\mathbb{1}$	0	$\mathbb{1}$	$\mathbb{1}$	0	0	$\mathbb{1}$
4, 4	000	0	$6\bar{2}$	0	0	0	0	0	0	0	0	0	0
4, 4	000	2	04	2	$\mathbb{1}$	$\mathbb{1}$	0	$\mathbb{1}$	$\mathbb{1}$	0	0	$\mathbb{1}$	$\mathbb{1}$
4, 4	000	2	$0\bar{2}$	0	$\mathbb{1}$	0	$\mathbb{1}$	$\mathbb{1}$	0	$\mathbb{1}$	0	$\mathbb{1}$	0
4, 4	000	2	64	0	$\mathbb{1}$	0	$\mathbb{1}$	$\mathbb{1}$	0	$\mathbb{1}$	0	$\mathbb{1}$	0
4, 4	000	2	$6\bar{2}$	2	$\mathbb{1}$	$\mathbb{1}$	0	$\mathbb{1}$	$\mathbb{1}$	0	0	$\mathbb{1}$	$\mathbb{1}$
4, 6	000	0	24	0	0	0	0	$\mathbb{1}$	$\mathbb{1}$	0	$\mathbb{1}$	$\mathbb{1}$	$\mathbb{1}$
4, 6	000	0	$2\bar{2}$	2	0	$\mathbb{1}$	$\mathbb{1}$	$\mathbb{1}$	0	$\mathbb{1}$	$\mathbb{1}$	$\mathbb{1}$	0
4, 6	000	0	$\bar{4}4$	2	0	$\mathbb{1}$	$\mathbb{1}$	$\mathbb{1}$	0	$\mathbb{1}$	$\mathbb{1}$	$\mathbb{1}$	0
4, 6	000	0	$\bar{4}\bar{2}$	0	0	0	0	$\mathbb{1}$	$\mathbb{1}$	0	$\mathbb{1}$	$\mathbb{1}$	$\mathbb{1}$
4, 6	000	2	24	2	$\mathbb{1}$	$\mathbb{1}$	0	0	0	0	$\mathbb{1}$	0	0
4, 6	000	2	$2\bar{2}$	0	$\mathbb{1}$	0	$\mathbb{1}$	0	$\mathbb{1}$	$\mathbb{1}$	$\mathbb{1}$	0	$\mathbb{1}$
4, 6	000	2	$\bar{4}4$	0	$\mathbb{1}$	0	$\mathbb{1}$	0	$\mathbb{1}$	$\mathbb{1}$	$\mathbb{1}$	0	$\mathbb{1}$
4, 6	000	2	$\bar{4}\bar{2}$	2	$\mathbb{1}$	$\mathbb{1}$	0	0	0	0	$\mathbb{1}$	0	0
4, 8	000	0	44	0	0	0	0	0	0	0	0	0	0
4, 8	000	0	$4\bar{2}$	2	0	$\mathbb{1}$	$\mathbb{1}$	0	$\mathbb{1}$	$\mathbb{1}$	0	0	$\mathbb{1}$
4, 8	000	0	$\bar{2}4$	2	0	$\mathbb{1}$	$\mathbb{1}$	0	$\mathbb{1}$	$\mathbb{1}$	0	0	$\mathbb{1}$
4, 8	000	0	$\bar{2}\bar{2}$	0	0	0	0	0	0	0	0	0	0
4, 8	000	2	44	2	$\mathbb{1}$	$\mathbb{1}$	0	$\mathbb{1}$	$\mathbb{1}$	0	0	$\mathbb{1}$	$\mathbb{1}$
4, 8	000	2	$4\bar{2}$	0	$\mathbb{1}$	0	$\mathbb{1}$	$\mathbb{1}$	0	$\mathbb{1}$	0	$\mathbb{1}$	0
4, 8	000	2	$\bar{2}4$	0	$\mathbb{1}$	0	$\mathbb{1}$	$\mathbb{1}$	0	$\mathbb{1}$	0	$\mathbb{1}$	0
4, 8	000	2	$\bar{2}\bar{2}$	2	$\mathbb{1}$	$\mathbb{1}$	0	$\mathbb{1}$	$\mathbb{1}$	0	0	$\mathbb{1}$	$\mathbb{1}$
4,10	000	0	04	2	0	$\mathbb{1}$	$\mathbb{1}$	$\mathbb{1}$	0	$\mathbb{1}$	$\mathbb{1}$	$\mathbb{1}$	0

Continued on next page

Supplementary Table 7 – continued

SI	Invariants												
4,10	000	0	$0\bar{2}$	0	0	0	0	𝟙	𝟙	0	𝟙	𝟙	𝟙
4,10	000	0	64	0	0	0	0	𝟙	𝟙	0	𝟙	𝟙	𝟙
4,10	000	0	$6\bar{2}$	2	0	𝟙	𝟙	𝟙	0	𝟙	𝟙	𝟙	0
4,10	000	2	04	0	𝟙	0	𝟙	0	𝟙	𝟙	𝟙	0	𝟙
4,10	000	2	$0\bar{2}$	2	𝟙	𝟙	0	0	0	0	𝟙	0	0
4,10	000	2	64	2	𝟙	𝟙	0	0	0	0	𝟙	0	0
4,10	000	2	$6\bar{2}$	0	𝟙	0	𝟙	0	𝟙	𝟙	𝟙	0	𝟙
5, 0	001	0	15	2	0	𝟙	𝟙	0	1	𝟙	0	1	0
5, 0	001	0	$1\bar{1}$	0	0	0	0	0	0	0	0	1	1
5, 0	001	0	$\bar{5}5$	0	0	0	0	0	0	0	0	1	1
5, 0	001	0	$\bar{5}\bar{1}$	2	0	𝟙	𝟙	0	1	𝟙	0	1	0
5, 0	001	2	15	0	𝟙	0	𝟙	1	0	𝟙	0	0	1
5, 0	001	2	$1\bar{1}$	2	𝟙	𝟙	0	1	1	0	0	0	0
5, 0	001	2	$\bar{5}5$	2	𝟙	𝟙	0	1	1	0	0	0	0
5, 0	001	2	$\bar{5}\bar{1}$	0	𝟙	0	𝟙	1	0	𝟙	0	0	1
5, 2	001	0	35	2	0	𝟙	𝟙	1	0	𝟙	1	0	1
5, 2	001	0	$3\bar{1}$	0	0	0	0	1	1	0	1	0	0
5, 2	001	0	$\bar{3}5$	0	0	0	0	1	1	0	1	0	0
5, 2	001	0	$\bar{3}\bar{1}$	2	0	𝟙	𝟙	1	0	𝟙	1	0	1
5, 2	001	2	35	0	𝟙	0	𝟙	0	1	𝟙	1	1	0
5, 2	001	2	$3\bar{1}$	2	𝟙	𝟙	0	0	0	0	1	1	1
5, 2	001	2	$\bar{3}5$	2	𝟙	𝟙	0	0	0	0	1	1	1
5, 2	001	2	$\bar{3}\bar{1}$	0	𝟙	0	𝟙	0	1	𝟙	1	1	0
5, 4	001	0	55	2	0	𝟙	𝟙	0	1	𝟙	0	1	0
5, 4	001	0	$5\bar{1}$	0	0	0	0	0	0	0	0	1	1
5, 4	001	0	$\bar{1}5$	0	0	0	0	0	0	0	0	1	1
5, 4	001	0	$\bar{1}\bar{1}$	2	0	𝟙	𝟙	0	1	𝟙	0	1	0
5, 4	001	2	55	0	𝟙	0	𝟙	1	0	𝟙	0	0	1
5, 4	001	2	$5\bar{1}$	2	𝟙	𝟙	0	1	1	0	0	0	0
5, 4	001	2	$\bar{1}5$	2	𝟙	𝟙	0	1	1	0	0	0	0
5, 4	001	2	$\bar{1}\bar{1}$	0	𝟙	0	𝟙	1	0	𝟙	0	0	1
5, 6	001	0	15	0	0	0	0	1	1	0	1	0	0
5, 6	001	0	$1\bar{1}$	2	0	𝟙	𝟙	1	0	𝟙	1	0	1
5, 6	001	0	$\bar{5}5$	2	0	𝟙	𝟙	1	0	𝟙	1	0	1
5, 6	001	0	$\bar{5}\bar{1}$	0	0	0	0	1	1	0	1	0	0
5, 6	001	2	15	2	𝟙	𝟙	0	0	0	0	1	1	1
5, 6	001	2	$1\bar{1}$	0	𝟙	0	𝟙	0	1	𝟙	1	1	0
5, 6	001	2	$\bar{5}5$	0	𝟙	0	𝟙	0	1	𝟙	1	1	0
5, 6	001	2	$\bar{5}\bar{1}$	2	𝟙	𝟙	0	0	0	0	1	1	1
5, 8	001	0	35	0	0	0	0	0	0	0	0	1	1
5, 8	001	0	$3\bar{1}$	2	0	𝟙	𝟙	0	1	𝟙	0	1	0
5, 8	001	0	$\bar{3}5$	2	0	𝟙	𝟙	0	1	𝟙	0	1	0
5, 8	001	0	$\bar{3}\bar{1}$	0	0	0	0	0	0	0	0	1	1
5, 8	001	2	35	2	𝟙	𝟙	0	1	1	0	0	0	0
5, 8	001	2	$3\bar{1}$	0	𝟙	0	𝟙	1	0	𝟙	0	0	1
5, 8	001	2	$\bar{3}5$	0	𝟙	0	𝟙	1	0	𝟙	0	0	1
5, 8	001	2	$\bar{3}\bar{1}$	2	𝟙	𝟙	0	1	1	0	0	0	0
5,10	001	0	55	0	0	0	0	1	1	0	1	0	0
5,10	001	0	$5\bar{1}$	2	0	𝟙	𝟙	1	0	𝟙	1	0	1
5,10	001	0	$\bar{1}5$	2	0	𝟙	𝟙	1	0	𝟙	1	0	1
5,10	001	0	$\bar{1}\bar{1}$	0	0	0	0	1	1	0	1	0	0
5,10	001	2	55	2	𝟙	𝟙	0	0	0	0	1	1	1
5,10	001	2	$5\bar{1}$	0	𝟙	0	𝟙	0	1	𝟙	1	1	0
5,10	001	2	$\bar{1}5$	0	𝟙	0	𝟙	0	1	𝟙	1	1	0
5,10	001	2	$\bar{1}\bar{1}$	2	𝟙	𝟙	0	0	0	0	1	1	1

Space group #192 : $P6/mcc$

$\mathbb{Z}_{12}$	weak	$m^{001}_{(6)}$	$g^{\bar{1}20}_{00\frac{1}{2}}$	$g^{\bar{1}20}_{1\frac{1}{2}\frac{1}{2}}$	$g^{010}_{00\frac{1}{2}}$	$g^{010}_{\frac{1}{2}0\frac{1}{2}}$	2^{001}	2^{010}	$2^{1\bar{1}0}$	6^{001}	i	2^{010}_1	$2^{1\bar{1}0}_1$
0	000	00	0	0	0	0	0	0	0	0	0	0	0
0	000	00	𝟙	𝟙	𝟙	𝟙	0	𝟙	𝟙	0	0	𝟙	𝟙
0	000	60	0	0	𝟙	𝟙	𝟙	0	𝟙	𝟙	0	0	𝟙

Continued on next page

Supplementary Table 7 – continued

SI	Invariants												
0	000	60	𝟙	𝟙	0	0	𝟙	𝟙	0	𝟙	0	𝟙	0
2	000	20	0	0	0	0	0	𝟙	𝟙	0	𝟙	𝟙	𝟙
2	000	20	𝟙	𝟙	𝟙	𝟙	0	0	0	0	𝟙	0	0
2	000	$\bar{4}0$	0	0	𝟙	𝟙	𝟙	𝟙	0	𝟙	𝟙	𝟙	0
2	000	$\bar{4}0$	𝟙	𝟙	0	0	𝟙	0	𝟙	𝟙	𝟙	0	𝟙
4	000	40	0	0	0	0	0	0	0	0	0	0	0
4	000	40	𝟙	𝟙	𝟙	𝟙	0	𝟙	𝟙	0	0	𝟙	𝟙
4	000	$\bar{2}0$	0	0	𝟙	𝟙	𝟙	0	𝟙	𝟙	0	0	𝟙
4	000	$\bar{2}0$	𝟙	𝟙	0	0	𝟙	𝟙	0	𝟙	0	𝟙	0
6	000	00	0	0	𝟙	𝟙	𝟙	𝟙	0	𝟙	𝟙	𝟙	0
6	000	00	𝟙	𝟙	0	0	𝟙	0	𝟙	𝟙	𝟙	0	𝟙
6	000	60	0	0	0	0	0	𝟙	𝟙	0	𝟙	𝟙	𝟙
6	000	60	𝟙	𝟙	𝟙	𝟙	0	0	0	0	𝟙	0	0
8	000	20	0	0	𝟙	𝟙	𝟙	0	𝟙	𝟙	0	0	𝟙
8	000	20	𝟙	𝟙	0	0	𝟙	𝟙	0	𝟙	0	𝟙	0
8	000	$\bar{4}0$	0	0	0	0	0	0	0	0	0	0	0
8	000	$\bar{4}0$	𝟙	𝟙	𝟙	𝟙	0	𝟙	𝟙	0	0	𝟙	𝟙
10	000	40	0	0	𝟙	𝟙	𝟙	𝟙	0	𝟙	𝟙	𝟙	0
10	000	40	𝟙	𝟙	0	0	𝟙	0	𝟙	𝟙	𝟙	0	𝟙
10	000	$\bar{2}0$	0	0	0	0	0	𝟙	𝟙	0	𝟙	𝟙	𝟙
10	000	$\bar{2}0$	𝟙	𝟙	𝟙	𝟙	0	0	0	0	𝟙	0	0

Space group #193 : $P6_3/mcm$

$\mathbb{Z}_{12}$	weak	$m_{(6)}^{001}$	$m_{(2)}^{010}$	$g_{00\frac{1}{2}}^{\bar{1}20}$	$g_{1\frac{1}{2}\frac{1}{2}}^{\bar{1}20}$	$g_{\frac{1}{2}00}^{010}$	2^{010}	$2^{1\bar{1}0}$	i	2_1^{001}	2_1^{010}	$2_1^{1\bar{1}0}$	6_3^{001}
0	000	00	0	0	0	0	0	0	0	0	0	0	0
0	000	00	2	𝟙	𝟙	𝟙	𝟙	𝟙	0	0	𝟙	𝟙	0
0	000	60	0	𝟙	𝟙	0	𝟙	0	0	𝟙	𝟙	0	𝟙
0	000	60	2	0	0	𝟙	0	𝟙	0	𝟙	0	𝟙	𝟙
2	000	20	0	0	0	0	𝟙	𝟙	𝟙	0	𝟙	𝟙	0
2	000	20	2	𝟙	𝟙	𝟙	0	0	𝟙	0	0	0	0
2	000	$\bar{4}0$	0	𝟙	𝟙	0	0	𝟙	𝟙	𝟙	0	𝟙	𝟙
2	000	$\bar{4}0$	2	0	0	𝟙	𝟙	0	𝟙	𝟙	𝟙	0	𝟙
4	000	40	0	0	0	0	0	0	0	0	0	0	0
4	000	40	2	𝟙	𝟙	𝟙	𝟙	𝟙	0	0	𝟙	𝟙	0
4	000	$\bar{2}0$	0	𝟙	𝟙	0	𝟙	0	0	𝟙	𝟙	0	𝟙
4	000	$\bar{2}0$	2	0	0	𝟙	0	𝟙	0	𝟙	0	𝟙	𝟙
6	000	00	0	𝟙	𝟙	0	0	𝟙	𝟙	𝟙	0	𝟙	𝟙
6	000	00	2	0	0	𝟙	𝟙	0	𝟙	𝟙	𝟙	0	𝟙
6	000	60	0	0	0	0	𝟙	𝟙	𝟙	0	𝟙	𝟙	0
6	000	60	2	𝟙	𝟙	𝟙	0	0	𝟙	0	0	0	0
8	000	20	0	𝟙	𝟙	0	𝟙	0	0	𝟙	𝟙	0	𝟙
8	000	20	2	0	0	𝟙	0	𝟙	0	𝟙	0	𝟙	𝟙
8	000	$\bar{4}0$	0	0	0	0	0	0	0	0	0	0	0
8	000	$\bar{4}0$	2	𝟙	𝟙	𝟙	𝟙	𝟙	0	0	𝟙	𝟙	0
10	000	40	0	𝟙	𝟙	0	0	𝟙	𝟙	𝟙	0	𝟙	𝟙
10	000	40	2	0	0	𝟙	𝟙	0	𝟙	𝟙	𝟙	0	𝟙
10	000	$\bar{2}0$	0	0	0	0	𝟙	𝟙	𝟙	0	𝟙	𝟙	0
10	000	$\bar{2}0$	2	𝟙	𝟙	𝟙	0	0	𝟙	0	0	0	0

Space group #194 : $P6_3/mmc$

$\mathbb{Z}_{12}$	weak	$m_{(2)}^{\bar{1}20}$	$m_{(6)}^{001}$	$g_{1\frac{1}{2}0}^{\bar{1}20}$	$g_{00\frac{1}{2}}^{010}$	$g_{\frac{1}{2}0\frac{1}{2}}^{010}$	2^{010}	$2^{1\bar{1}0}$	i	2_1^{001}	2_1^{010}	$2_1^{1\bar{1}0}$	6_3^{001}
0	000	0	00	0	0	0	0	0	0	0	0	0	0
0	000	0	60	0	𝟙	𝟙	0	𝟙	0	𝟙	0	𝟙	𝟙
0	000	2	00	𝟙	𝟙	𝟙	𝟙	𝟙	0	0	𝟙	𝟙	0
0	000	2	60	𝟙	0	0	𝟙	0	0	𝟙	𝟙	0	𝟙
2	000	0	20	0	0	0	𝟙	𝟙	𝟙	0	𝟙	𝟙	0
2	000	0	$\bar{4}0$	0	𝟙	𝟙	𝟙	0	𝟙	𝟙	𝟙	0	𝟙
2	000	2	20	𝟙	𝟙	𝟙	0	0	𝟙	0	0	0	0
2	000	2	$\bar{4}0$	𝟙	0	0	0	𝟙	𝟙	𝟙	0	𝟙	𝟙
4	000	0	40	0	0	0	0	0	0	0	0	0	0
4	000	0	$\bar{2}0$	0	𝟙	𝟙	0	𝟙	0	𝟙	0	𝟙	𝟙

Continued on next page

Supplementary Table 7 – continued

SI	Invariants												
4	000	2	40	𝟙	𝟙	𝟙	𝟙	𝟙	0	0	𝟙	𝟙	0
4	000	2	$\bar{2}0$	𝟙	0	0	𝟙	0	0	𝟙	𝟙	0	𝟙
6	000	0	00	0	𝟙	𝟙	𝟙	0	𝟙	𝟙	𝟙	0	𝟙
6	000	0	60	0	0	0	𝟙	𝟙	𝟙	0	𝟙	𝟙	0
6	000	2	00	𝟙	0	0	0	𝟙	𝟙	𝟙	0	𝟙	𝟙
6	000	2	60	𝟙	𝟙	𝟙	0	0	𝟙	0	0	0	0
8	000	0	20	0	𝟙	𝟙	0	𝟙	0	𝟙	0	𝟙	𝟙
8	000	0	$\bar{4}0$	0	0	0	0	0	0	0	0	0	0
8	000	2	20	𝟙	0	0	𝟙	0	0	𝟙	𝟙	0	𝟙
8	000	2	$\bar{4}0$	𝟙	𝟙	𝟙	𝟙	𝟙	0	0	𝟙	𝟙	0
10	000	0	40	0	𝟙	𝟙	𝟙	0	𝟙	𝟙	𝟙	0	𝟙
10	000	0	$\bar{2}0$	0	0	0	𝟙	𝟙	𝟙	0	𝟙	𝟙	0
10	000	2	40	𝟙	0	0	0	𝟙	𝟙	𝟙	0	𝟙	𝟙
10	000	2	$\bar{2}0$	𝟙	𝟙	𝟙	0	0	𝟙	0	0	0	0

Space group #200 : $Pm\bar{3}$

$\mathbb{Z}_{2,2,2,4}$	weak	$m^{001}_{(2)}$	2^{001}	i
0000	000	00	0	0
0000	000	22	0	0
0002	000	02	0	𝟙
0002	000	20	0	𝟙
1110	111	$1\bar{1}$	0	0
1110	111	$\bar{1}1$	0	0
1112	111	11	0	1
1112	111	$\bar{1}\bar{1}$	0	1

Space group #201 : $Pn\bar{3}$

$\mathbb{Z}_{2,2,2,4}$	weak	$g^{001}_{\frac{1}{2}\frac{1}{2}0}$	2^{001}	i
0000	000	0	0	0
0002	000	𝟙	0	𝟙

Space group #202 : $Fm\bar{3}$

$\mathbb{Z}_{2,2,2,4}$	weak	$m^{001}_{(2)}$	$g^{001}_{\frac{1}{2}00}$	2^{001}	i	2^{001}_{1}
0000	000	0	0	0	0	0
0002	000	2	𝟙	0	𝟙	0

Space group #203 : $Fd\bar{3}$

$\mathbb{Z}_{2,2,2,4}$	weak	$g^{001}_{\frac{1}{4}\frac{1}{4}0}$	$g^{001}_{\frac{1}{4}\bar{\frac{1}{4}}0}$	2^{001}	i	2^{001}_{1}
0000	000	0	0	0	0	0
0002	000	𝟙	𝟙	0	𝟙	0

Space group #204 : $Im\bar{3}$

$\mathbb{Z}_{2,2,2,4}$	weak	$m^{001}_{(2)}$	$g^{001}_{\frac{1}{2}\frac{1}{2}0}$	2^{001}	i	2^{001}_{1}
0000	000	0	0	0	0	0
0002	000	2	𝟙	0	𝟙	0
1110	111	0	𝟙	0	0	𝟙
1112	111	2	0	0	1	𝟙

Space group #205 : $Pa\bar{3}$

$\mathbb{Z}_{2,2,2,4}$	weak	$g^{001}_{\frac{1}{2}00}$	i	2^{001}_{1}
0000	000	0	0	0
0002	000	𝟙	𝟙	0

Space group #206 : $Ia\bar{3}$

$\mathbb{Z}_{2,2,2,4}$	weak	$g^{001}_{0\frac{1}{2}0}$	$g^{001}_{\frac{1}{2}00}$	2^{001}	i	2^{001}_{1}
0000	000	0	0	0	0	0
0002	000	𝟙	𝟙	0	𝟙	0
1110	111	𝟙	0	𝟙	0	0
1112	111	0	𝟙	𝟙	1	0

Space group #221 : $Pm\bar{3}m$

$\mathbb{Z}_{4,8}$	weak	$m^{001}_{(4)}$	$m^{101}_{(2)}$	$g^{101}_{\frac{1}{2}0\frac{1}{2}}$	2^{001}	2^{011}	4^{001}	i	2^{011}_{1}	$\bar{4}^{001}$
00	000	00	0	0	0	0	0	0	0	0
00	000	04	2	𝟙	0	𝟙	𝟙	0	𝟙	𝟙

Continued on next page

Supplementary Table 7 – continued

SI	Invariants									
00	000	40	2	𝟙	0	𝟙	𝟙	0	𝟙	𝟙
00	000	44	0	0	0	0	0	0	0	0
02	000	20	2	𝟙	0	0	0	𝟙	0	𝟙
02	000	24	0	0	0	𝟙	𝟙	𝟙	𝟙	0
02	000	$\bar{2}0$	0	0	0	𝟙	𝟙	𝟙	𝟙	0
02	000	$\bar{2}4$	2	𝟙	0	0	0	𝟙	0	𝟙
04	000	00	2	𝟙	0	𝟙	𝟙	0	𝟙	𝟙
04	000	04	0	0	0	0	0	0	0	0
04	000	40	0	0	0	0	0	0	0	0
04	000	44	2	𝟙	0	𝟙	𝟙	0	𝟙	𝟙
06	000	20	0	0	0	𝟙	𝟙	𝟙	𝟙	0
06	000	24	2	𝟙	0	0	0	𝟙	0	𝟙
06	000	$\bar{2}0$	2	𝟙	0	0	0	𝟙	0	𝟙
06	000	$\bar{2}4$	0	0	0	𝟙	𝟙	𝟙	𝟙	0
10	111	31	2	0	0	1	1	0	0	1
10	111	$3\bar{3}$	0	𝟙	0	0	0	0	1	0
10	111	$\bar{1}1$	0	𝟙	0	0	0	0	1	0
10	111	$\bar{1}\bar{3}$	2	0	0	1	1	0	0	1
12	111	11	2	0	0	0	0	1	1	1
12	111	$1\bar{3}$	0	𝟙	0	1	1	1	0	0
12	111	$\bar{3}1$	0	𝟙	0	1	1	1	0	0
12	111	$\bar{3}\bar{3}$	2	0	0	0	0	1	1	1
14	111	31	0	𝟙	0	0	0	0	1	0
14	111	$3\bar{3}$	2	0	0	1	1	0	0	1
14	111	$\bar{1}1$	2	0	0	1	1	0	0	1
14	111	$\bar{1}\bar{3}$	0	𝟙	0	0	0	0	1	0
16	111	11	0	𝟙	0	1	1	1	0	0
16	111	$1\bar{3}$	2	0	0	0	0	1	1	1
16	111	$\bar{3}1$	2	0	0	0	0	1	1	1
16	111	$\bar{3}\bar{3}$	0	𝟙	0	1	1	1	0	0
20	000	22	2	𝟙	0	𝟙	𝟙	0	𝟙	𝟙
20	000	$2\bar{2}$	0	0	0	0	0	0	0	0
20	000	$\bar{2}2$	0	0	0	0	0	0	0	0
20	000	$\bar{2}\bar{2}$	2	𝟙	0	𝟙	𝟙	0	𝟙	𝟙
22	000	02	2	𝟙	0	0	0	𝟙	0	𝟙
22	000	$0\bar{2}$	0	0	0	𝟙	𝟙	𝟙	𝟙	0
22	000	42	0	0	0	𝟙	𝟙	𝟙	𝟙	0
22	000	$4\bar{2}$	2	𝟙	0	0	0	𝟙	0	𝟙
24	000	22	0	0	0	0	0	0	0	0
24	000	$2\bar{2}$	2	𝟙	0	𝟙	𝟙	0	𝟙	𝟙
24	000	$\bar{2}2$	2	𝟙	0	𝟙	𝟙	0	𝟙	𝟙
24	000	$\bar{2}\bar{2}$	0	0	0	0	0	0	0	0
26	000	02	0	0	0	𝟙	𝟙	𝟙	𝟙	0
26	000	$0\bar{2}$	2	𝟙	0	0	0	𝟙	0	𝟙
26	000	42	2	𝟙	0	0	0	𝟙	0	𝟙
26	000	$4\bar{2}$	0	0	0	𝟙	𝟙	𝟙	𝟙	0
30	111	13	2	0	0	1	1	0	0	1
30	111	$1\bar{1}$	0	𝟙	0	0	0	0	1	0
30	111	$\bar{3}3$	0	𝟙	0	0	0	0	1	0
30	111	$\bar{3}\bar{1}$	2	0	0	1	1	0	0	1
32	111	33	0	𝟙	0	1	1	1	0	0
32	111	$3\bar{1}$	2	0	0	0	0	1	1	1
32	111	$\bar{1}3$	2	0	0	0	0	1	1	1
32	111	$\bar{1}\bar{1}$	0	𝟙	0	1	1	1	0	0
34	111	13	0	𝟙	0	0	0	0	1	0
34	111	$1\bar{1}$	2	0	0	1	1	0	0	1
34	111	$\bar{3}3$	2	0	0	1	1	0	0	1
34	111	$\bar{3}\bar{1}$	0	𝟙	0	0	0	0	1	0
36	111	33	2	0	0	0	0	1	1	1
36	111	$3\bar{1}$	0	𝟙	0	1	1	1	0	0
36	111	$\bar{1}3$	0	𝟙	0	1	1	1	0	0

Continued on next page

Supplementary Table 7 – continued

**SI**	**Invariants**									
36	111	$\bar{1}\bar{1}$	2	0	0	0	0	1	1	1

Space group #222 : $Pn\bar{3}n$

$\mathbb{Z}_{2,2,2,4}$	weak	$g^{001}_{\frac{1}{2}\frac{1}{2}0}$	$g^{101}_{0\frac{1}{2}0}$	$g^{101}_{\frac{\bar{1}}{2}\frac{1}{2}\frac{1}{2}}$	2^{001}	2^{011}	4^{001}	i	2_1^{011}	$\bar{4}^{001}$
0000	000	0	0	0	0	0	0	0	0	0
0000	000	0	$\mathbb{1}$	$\mathbb{1}$	0	$\mathbb{1}$	$\mathbb{1}$	0	$\mathbb{1}$	$\mathbb{1}$
0002	000	$\mathbb{1}$	0	0	0	$\mathbb{1}$	$\mathbb{1}$	$\mathbb{1}$	$\mathbb{1}$	0
0002	000	$\mathbb{1}$	$\mathbb{1}$	$\mathbb{1}$	0	0	0	$\mathbb{1}$	0	$\mathbb{1}$

Space group #223 : $Pm\bar{3}n$

$\mathbb{Z}_{2,2,2,4}$	weak	$m^{001}_{(4)}$	$g^{101}_{0\frac{1}{2}0}$	$g^{101}_{\frac{\bar{1}}{2}\frac{1}{2}\frac{1}{2}}$	2^{001}	2^{011}	i	2_1^{011}	4_2^{001}	$\bar{4}^{001}$
0000	000	00	0	0	0	0	0	0	0	0
0000	000	00	$\mathbb{1}$	$\mathbb{1}$	0	$\mathbb{1}$	0	$\mathbb{1}$	$\mathbb{1}$	$\mathbb{1}$
0000	000	40	0	0	0	0	0	0	0	0
0000	000	40	$\mathbb{1}$	$\mathbb{1}$	0	$\mathbb{1}$	0	$\mathbb{1}$	$\mathbb{1}$	$\mathbb{1}$
0002	000	20	0	0	0	$\mathbb{1}$	$\mathbb{1}$	$\mathbb{1}$	$\mathbb{1}$	0
0002	000	20	$\mathbb{1}$	$\mathbb{1}$	0	0	$\mathbb{1}$	0	0	$\mathbb{1}$
0002	000	$\bar{2}0$	0	0	0	$\mathbb{1}$	$\mathbb{1}$	$\mathbb{1}$	$\mathbb{1}$	0
0002	000	$\bar{2}0$	$\mathbb{1}$	$\mathbb{1}$	0	0	$\mathbb{1}$	0	0	$\mathbb{1}$

Space group #224 : $Pn\bar{3}m$

$\mathbb{Z}_{2,2,2,4}$	weak	$m^{101}_{(2)}$	$g^{001}_{\frac{1}{2}\frac{1}{2}0}$	$g^{101}_{\frac{\bar{1}}{2}0\frac{1}{2}}$	2^{001}	2^{011}	i	2_1^{011}	4_2^{001}	$\bar{4}^{001}$
0000	000	0	0	0	0	0	0	0	0	0
0000	000	2	0	$\mathbb{1}$	0	$\mathbb{1}$	0	$\mathbb{1}$	$\mathbb{1}$	$\mathbb{1}$
0002	000	0	$\mathbb{1}$	0	0	$\mathbb{1}$	$\mathbb{1}$	$\mathbb{1}$	$\mathbb{1}$	0
0002	000	2	$\mathbb{1}$	$\mathbb{1}$	0	0	$\mathbb{1}$	0	0	$\mathbb{1}$

Space group #225 : $Fm\bar{3}m$

$\mathbb{Z}_8$	weak	$m^{001}_{(4)}$	$m^{101}_{(2)}$	$g^{001}_{\frac{1}{2}00}$	$g^{101}_{\frac{1}{4}\frac{1}{2}\frac{\bar{1}}{4}}$	2^{001}	2^{011}	4^{001}	i	2_1^{001}	2_1^{011}	4_2^{001}	$\bar{4}^{001}$
0	000	0	0	0	0	0	0	0	0	0	0	0	0
0	000	4	2	0	$\mathbb{1}$	0	$\mathbb{1}$	$\mathbb{1}$	0	0	$\mathbb{1}$	$\mathbb{1}$	$\mathbb{1}$
2	000	2	2	$\mathbb{1}$	$\mathbb{1}$	0	0	0	$\mathbb{1}$	0	0	0	$\mathbb{1}$
2	000	$\bar{2}$	0	$\mathbb{1}$	0	0	$\mathbb{1}$	$\mathbb{1}$	$\mathbb{1}$	0	$\mathbb{1}$	$\mathbb{1}$	0
4	000	0	2	0	$\mathbb{1}$	0	$\mathbb{1}$	$\mathbb{1}$	0	0	$\mathbb{1}$	$\mathbb{1}$	$\mathbb{1}$
4	000	4	0	0	0	0	0	0	0	0	0	0	0
6	000	2	0	$\mathbb{1}$	0	0	$\mathbb{1}$	$\mathbb{1}$	$\mathbb{1}$	0	$\mathbb{1}$	$\mathbb{1}$	0
6	000	$\bar{2}$	2	$\mathbb{1}$	$\mathbb{1}$	0	0	0	$\mathbb{1}$	0	0	0	$\mathbb{1}$

Space group #226 : $Fm\bar{3}c$

$\mathbb{Z}_8$	weak	$m^{001}_{(4)}$	$g^{001}_{\frac{1}{2}00}$	$g^{101}_{0\frac{1}{2}0}$	$g^{101}_{\frac{1}{4}0\frac{\bar{1}}{4}}$	2^{001}	2^{011}	4^{001}	i	2_1^{001}	2_1^{011}	4_2^{001}	$\bar{4}^{001}$
0	000	0	0	0	0	0	0	0	0	0	0	0	0
0	000	4	0	$\mathbb{1}$	$\mathbb{1}$	0	$\mathbb{1}$	$\mathbb{1}$	0	0	$\mathbb{1}$	$\mathbb{1}$	$\mathbb{1}$
2	000	2	$\mathbb{1}$	$\mathbb{1}$	$\mathbb{1}$	0	0	0	$\mathbb{1}$	0	0	0	$\mathbb{1}$
2	000	$\bar{2}$	$\mathbb{1}$	0	0	0	$\mathbb{1}$	$\mathbb{1}$	$\mathbb{1}$	0	$\mathbb{1}$	$\mathbb{1}$	0
4	000	0	0	$\mathbb{1}$	$\mathbb{1}$	0	$\mathbb{1}$	$\mathbb{1}$	0	0	$\mathbb{1}$	$\mathbb{1}$	$\mathbb{1}$
4	000	4	0	0	0	0	0	0	0	0	0	0	0
6	000	2	$\mathbb{1}$	0	0	0	$\mathbb{1}$	$\mathbb{1}$	$\mathbb{1}$	0	$\mathbb{1}$	$\mathbb{1}$	0
6	000	$\bar{2}$	$\mathbb{1}$	$\mathbb{1}$	$\mathbb{1}$	0	0	0	$\mathbb{1}$	0	0	0	$\mathbb{1}$

Space group #227 : $Fd\bar{3}m$

$\mathbb{Z}_{2,2,2,4}$	weak	$m^{101}_{(2)}$	$g^{001}_{\frac{1}{4}\frac{1}{4}0}$	$g^{001}_{\frac{1}{4}\frac{\bar{1}}{4}0}$	$g^{101}_{\frac{1}{4}\frac{1}{2}\frac{\bar{1}}{4}}$	2^{001}	2^{011}	i	2_1^{001}	2_1^{011}	4_1^{001}	4_3^{001}	$\bar{4}^{001}$
0000	000	0	0	0	0	0	0	0	0	0	0	0	0
0000	000	2	0	0	$\mathbb{1}$	0	$\mathbb{1}$	0	0	$\mathbb{1}$	$\mathbb{1}$	$\mathbb{1}$	$\mathbb{1}$
0002	000	0	$\mathbb{1}$	$\mathbb{1}$	0	0	$\mathbb{1}$	$\mathbb{1}$	0	$\mathbb{1}$	$\mathbb{1}$	$\mathbb{1}$	0
0002	000	2	$\mathbb{1}$	$\mathbb{1}$	$\mathbb{1}$	0	0	$\mathbb{1}$	0	0	0	0	$\mathbb{1}$

Space group #228 : $Fd\bar{3}c$

$\mathbb{Z}_{2,2,2,4}$	weak	$g^{001}_{\frac{1}{4}\frac{1}{4}0}$	$g^{001}_{\frac{1}{4}\frac{\bar{1}}{4}0}$	$g^{101}_{0\frac{1}{2}0}$	$g^{101}_{\frac{1}{4}0\frac{\bar{1}}{4}}$	2^{001}	2^{011}	i	2_1^{001}	2_1^{011}	4_1^{001}	4_3^{001}	$\bar{4}^{001}$
0000	000	0	0	0	0	0	0	0	0	0	0	0	0
0000	000	0	0	$\mathbb{1}$	$\mathbb{1}$	0	$\mathbb{1}$	0	0	$\mathbb{1}$	$\mathbb{1}$	$\mathbb{1}$	$\mathbb{1}$
0002	000	$\mathbb{1}$	$\mathbb{1}$	0	0	0	$\mathbb{1}$	$\mathbb{1}$	0	$\mathbb{1}$	$\mathbb{1}$	$\mathbb{1}$	0
0002	000	$\mathbb{1}$	$\mathbb{1}$	$\mathbb{1}$	$\mathbb{1}$	0	0	$\mathbb{1}$	0	0	0	0	$\mathbb{1}$

Space group #229 : $Im\bar{3}m$

Continued on next page

Supplementary Table 7 – continued

**SI**	**Invariants**												
$\mathbb{Z}_{2,8}$	weak	$m_{(4)}^{001}$	$m_{(2)}^{101}$	$g_{\frac{1}{2}\frac{1}{2}0}^{001}$	$g_{0\frac{1}{2}0}^{101}$	2^{001}	2^{011}	4^{001}	i	2_1^{001}	2_1^{011}	4_2^{001}	$\bar{4}^{001}$
00	000	0	0	0	0	0	0	0	0	0	0	0	0
00	000	4	2	0	$\mathbb{1}$	0	$\mathbb{1}$	$\mathbb{1}$	0	0	$\mathbb{1}$	$\mathbb{1}$	$\mathbb{1}$
02	000	2	2	$\mathbb{1}$	$\mathbb{1}$	0	0	0	$\mathbb{1}$	0	0	0	$\mathbb{1}$
02	000	$\bar{2}$	0	$\mathbb{1}$	0	0	$\mathbb{1}$	$\mathbb{1}$	$\mathbb{1}$	0	$\mathbb{1}$	$\mathbb{1}$	0
04	000	0	2	0	$\mathbb{1}$	0	$\mathbb{1}$	$\mathbb{1}$	0	0	$\mathbb{1}$	$\mathbb{1}$	$\mathbb{1}$
04	000	4	0	0	0	0	0	0	0	0	0	0	0
06	000	2	0	$\mathbb{1}$	0	0	$\mathbb{1}$	$\mathbb{1}$	$\mathbb{1}$	0	$\mathbb{1}$	$\mathbb{1}$	0
06	000	$\bar{2}$	2	$\mathbb{1}$	$\mathbb{1}$	0	0	0	$\mathbb{1}$	0	0	0	$\mathbb{1}$
10	111	0	0	$\mathbb{1}$	$\mathbb{1}$	0	0	0	0	$\mathbb{1}$	1	$\mathbb{1}$	0
10	111	4	2	$\mathbb{1}$	0	0	1	$\mathbb{1}$	0	$\mathbb{1}$	0	0	1
12	111	2	2	0	0	0	0	0	1	$\mathbb{1}$	1	$\mathbb{1}$	1
12	111	$\bar{2}$	0	0	$\mathbb{1}$	0	1	$\mathbb{1}$	1	$\mathbb{1}$	0	0	0
14	111	0	2	$\mathbb{1}$	0	0	1	$\mathbb{1}$	0	$\mathbb{1}$	0	0	1
14	111	4	0	$\mathbb{1}$	$\mathbb{1}$	0	0	0	0	$\mathbb{1}$	1	$\mathbb{1}$	0
16	111	2	0	0	$\mathbb{1}$	0	1	$\mathbb{1}$	1	$\mathbb{1}$	0	0	0
16	111	$\bar{2}$	2	0	0	0	0	0	1	$\mathbb{1}$	1	$\mathbb{1}$	1
Space group #230 : $Ia\bar{3}d$													
$\mathbb{Z}_{2,2,2,4}$	weak	$g_{0\frac{1}{2}0}^{001}$	$g_{\frac{1}{2}00}^{001}$	$g_{\frac{\bar{1}}{4}\frac{1}{4}\frac{1}{4}}^{101}$	$g_{\frac{1}{4}\frac{1}{4}\frac{\bar{1}}{4}}^{101}$	2^{001}	2^{011}	i	2_1^{001}	2_1^{011}	4_1^{001}	4_3^{001}	$\bar{4}^{001}$
0000	000	0	0	0	0	0	0	0	0	0	0	0	0
0000	000	0	0	$\mathbb{1}$	$\mathbb{1}$	0	$\mathbb{1}$	0	0	$\mathbb{1}$	$\mathbb{1}$	$\mathbb{1}$	$\mathbb{1}$
0002	000	$\mathbb{1}$	$\mathbb{1}$	0	0	0	$\mathbb{1}$	$\mathbb{1}$	0	$\mathbb{1}$	$\mathbb{1}$	$\mathbb{1}$	0
0002	000	$\mathbb{1}$	$\mathbb{1}$	$\mathbb{1}$	$\mathbb{1}$	0	0	$\mathbb{1}$	0	0	0	0	$\mathbb{1}$

Supplementary Table 8: TCI invariant combinations in all SGs with trivial SI groups.

**Invariants**	
Space group #1 : $P1$	
weak	
000	
001	
010	
011	
100	
101	
110	
111	
Space group #3 : $P2$	
weak	2^{010}
000	0
000	$\mathbb{1}$
001	0
001	1
010	0
010	$\mathbb{1}$
011	0
011	1
100	0
100	1
101	0
101	1
110	0
110	1
111	0
111	1
Space group #4 : $P2_1$	
weak	2_1^{010}

Continued on next column

Supplementary Table 8 – continued

**Invariants**		
000	0	
000	$\mathbb{1}$	
001	0	
001	1	
100	0	
100	1	
101	0	
101	1	
Space group #5 : $C2$		
weak	2^{010}	2_1^{010}
000	0	0
000	$\mathbb{1}$	$\mathbb{1}$
001	0	0
001	1	1
110	0	$\mathbb{1}$
110	$\mathbb{1}$	0
111	0	1
111	1	0
Space group #6 : Pm		
weak	$m_{(2)}^{010}$	
000	00	
000	02	
000	20	
000	22	
001	00	
001	02	
001	20	
001	22	
010	11	
010	$1\bar{1}$	
010	$\bar{1}1$	
010	$\bar{1}\bar{1}$	

Continued on next column

Supplementary Table 8 – continued

Invariants	
011	11
011	$1\bar{1}$
011	$\bar{1}1$
011	$\bar{1}\bar{1}$
100	00
100	02
100	20
100	22
101	00
101	02
101	20
101	22
110	11
110	$1\bar{1}$
110	$\bar{1}1$
110	$\bar{1}\bar{1}$
111	11
111	$1\bar{1}$
111	$\bar{1}1$
111	$\bar{1}\bar{1}$

Space group #7 : Pc

weak	$g^{010}_{00\frac{1}{2}}$
000	0
000	$\mathbb{1}$
010	0
010	1
100	0
100	$\mathbb{1}$
110	0
110	1

Space group #8 : Cm

weak	$m^{010}_{(2)}$	$g^{010}_{\frac{1}{2}00}$
000	0	0
000	2	$\mathbb{1}$
001	0	0
001	2	$\mathbb{1}$
110	0	$\mathbb{1}$
110	2	0
111	0	$\mathbb{1}$
111	2	0

Space group #9 : Cc

weak	$g^{010}_{00\frac{1}{2}}$	$g^{010}_{\frac{1}{2}0\frac{1}{2}}$
000	0	0
000	$\mathbb{1}$	$\mathbb{1}$
110	0	$\mathbb{1}$
110	$\mathbb{1}$	0

Space group #16 : $P222$

weak	2^{001}	2^{010}	2^{100}
000	0	0	0
000	0	$\mathbb{1}$	$\mathbb{1}$
000	$\mathbb{1}$	0	$\mathbb{1}$
000	$\mathbb{1}$	$\mathbb{1}$	0
001	0	0	0
001	0	1	1
001	$\mathbb{1}$	0	1
001	$\mathbb{1}$	1	0
010	0	0	0
010	0	$\mathbb{1}$	1

Continued on next column

Supplementary Table 8 – continued

Invariants			
010	1	0	1
010	1	$\mathbb{1}$	0
011	0	0	0
011	0	1	1
011	1	0	1
011	1	1	0
100	0	0	0
100	0	1	$\mathbb{1}$
100	1	0	$\mathbb{1}$
100	1	1	0
101	0	0	0
101	0	1	1
101	1	0	1
101	1	1	0
110	0	0	0
110	0	1	1
110	1	0	1
110	1	1	0
111	0	0	0
111	0	1	1
111	1	0	1
111	1	1	0

Space group #17 : $P222_1$

weak	2^{010}	2^{100}	2_1^{001}
000	0	0	0
000	0	$\mathbb{1}$	$\mathbb{1}$
000	$\mathbb{1}$	0	$\mathbb{1}$
000	$\mathbb{1}$	$\mathbb{1}$	0
010	0	0	0
010	0	1	1
010	$\mathbb{1}$	0	1
010	$\mathbb{1}$	1	0
100	0	0	0
100	0	$\mathbb{1}$	1
100	1	0	1
100	1	$\mathbb{1}$	0
110	0	0	0
110	0	1	1
110	1	0	1
110	1	1	0

Space group #18 : $P2_12_12$

weak	2^{001}	2_1^{010}	2_1^{100}
000	0	0	0
000	0	$\mathbb{1}$	$\mathbb{1}$
000	$\mathbb{1}$	0	$\mathbb{1}$
000	$\mathbb{1}$	$\mathbb{1}$	0
001	0	0	0
001	0	1	1
001	$\mathbb{1}$	0	1
001	$\mathbb{1}$	1	0

Space group #19 : $P2_12_12_1$

weak	2_1^{001}	2_1^{010}	2_1^{100}
000	0	0	0
000	0	$\mathbb{1}$	$\mathbb{1}$
000	$\mathbb{1}$	0	$\mathbb{1}$
000	$\mathbb{1}$	$\mathbb{1}$	0

Space group #20 : $C222_1$

weak	2^{010}	2^{100}	2_1^{001}	2_1^{010}	2_1^{100}
000	0	0	0	0	0

Continued on next column

Supplementary Table 8 – continued

Invariants					
000	0	$\mathbb{1}$	$\mathbb{1}$	0	$\mathbb{1}$
000	$\mathbb{1}$	0	$\mathbb{1}$	$\mathbb{1}$	0
000	$\mathbb{1}$	$\mathbb{1}$	0	$\mathbb{1}$	$\mathbb{1}$
110	0	0	0	$\mathbb{1}$	$\mathbb{1}$
110	0	$\mathbb{1}$	1	$\mathbb{1}$	0
110	$\mathbb{1}$	0	1	0	$\mathbb{1}$
110	$\mathbb{1}$	$\mathbb{1}$	0	0	0

Space group #21 : $C222$

weak	2^{001}	2^{010}	2^{100}	2_1^{010}	2_1^{100}
000	0	0	0	0	0
000	0	$\mathbb{1}$	$\mathbb{1}$	$\mathbb{1}$	$\mathbb{1}$
000	$\mathbb{1}$	0	$\mathbb{1}$	0	$\mathbb{1}$
000	$\mathbb{1}$	$\mathbb{1}$	0	$\mathbb{1}$	0
001	0	0	0	0	0
001	0	1	1	1	1
001	$\mathbb{1}$	0	1	0	1
001	$\mathbb{1}$	1	0	1	0
110	0	0	0	$\mathbb{1}$	$\mathbb{1}$
110	0	$\mathbb{1}$	$\mathbb{1}$	0	0
110	1	0	$\mathbb{1}$	$\mathbb{1}$	0
110	1	$\mathbb{1}$	0	0	$\mathbb{1}$
111	0	0	0	1	1
111	0	1	1	0	0
111	1	0	1	1	0
111	1	1	0	0	1

Space group #22 : $F222$

weak	2^{001}	2^{010}	2^{100}	2_1^{001}	2_1^{010}	2_1^{100}
000	0	0	0	0	0	0
000	0	$\mathbb{1}$	$\mathbb{1}$	0	$\mathbb{1}$	$\mathbb{1}$
000	$\mathbb{1}$	0	$\mathbb{1}$	$\mathbb{1}$	0	$\mathbb{1}$
000	$\mathbb{1}$	$\mathbb{1}$	0	$\mathbb{1}$	$\mathbb{1}$	0
011	0	0	0	0	0	$\mathbb{1}$
011	0	1	$\mathbb{1}$	0	1	0
011	1	0	$\mathbb{1}$	1	0	0
011	1	1	0	1	1	$\mathbb{1}$
101	0	0	0	1	$\mathbb{1}$	0
101	0	$\mathbb{1}$	1	1	0	1
101	1	0	1	0	$\mathbb{1}$	1
101	1	$\mathbb{1}$	0	0	0	0
110	0	0	0	$\mathbb{1}$	1	1
110	0	1	1	$\mathbb{1}$	0	0
110	$\mathbb{1}$	0	1	0	1	0
110	$\mathbb{1}$	1	0	0	0	1

Space group #23 : $I222$

weak	2^{001}	2^{010}	2^{100}	2_1^{001}	2_1^{010}	2_1^{100}
000	0	0	0	0	0	0
000	0	$\mathbb{1}$	$\mathbb{1}$	0	$\mathbb{1}$	$\mathbb{1}$
000	$\mathbb{1}$	0	$\mathbb{1}$	$\mathbb{1}$	0	$\mathbb{1}$
000	$\mathbb{1}$	$\mathbb{1}$	0	$\mathbb{1}$	$\mathbb{1}$	0
111	0	0	0	$\mathbb{1}$	$\mathbb{1}$	$\mathbb{1}$
111	0	$\mathbb{1}$	$\mathbb{1}$	$\mathbb{1}$	0	0
111	$\mathbb{1}$	0	$\mathbb{1}$	0	$\mathbb{1}$	0
111	$\mathbb{1}$	$\mathbb{1}$	0	0	0	$\mathbb{1}$

Space group #24 : $I2_12_12_1$

weak	2^{001}	2^{010}	2^{100}	2_1^{001}	2_1^{010}	2_1^{100}
000	0	0	0	0	0	0
000	0	$\mathbb{1}$	$\mathbb{1}$	0	$\mathbb{1}$	$\mathbb{1}$
000	$\mathbb{1}$	0	$\mathbb{1}$	$\mathbb{1}$	0	$\mathbb{1}$
000	$\mathbb{1}$	$\mathbb{1}$	0	$\mathbb{1}$	$\mathbb{1}$	0

Continued on next column

Supplementary Table 8 – continued

Invariants						
111	0	0	$\mathbb{1}$	$\mathbb{1}$	$\mathbb{1}$	0
111	0	$\mathbb{1}$	0	$\mathbb{1}$	0	$\mathbb{1}$
111	$\mathbb{1}$	0	0	0	$\mathbb{1}$	$\mathbb{1}$
111	$\mathbb{1}$	$\mathbb{1}$	$\mathbb{1}$	0	0	0

Space group #25 : $Pmm2$

weak	$m_{(2)}^{010}$	$m_{(2)}^{100}$	2^{001}
000	00	00	0
000	00	02	$\mathbb{1}$
000	00	20	$\mathbb{1}$
000	00	22	0
000	02	00	$\mathbb{1}$
000	02	02	0
000	02	20	0
000	02	22	$\mathbb{1}$
000	20	00	$\mathbb{1}$
000	20	02	0
000	20	20	0
000	20	22	$\mathbb{1}$
000	22	00	0
000	22	02	$\mathbb{1}$
000	22	20	$\mathbb{1}$
000	22	22	0
001	00	00	0
001	00	02	$\mathbb{1}$
001	00	20	$\mathbb{1}$
001	00	22	0
001	02	00	$\mathbb{1}$
001	02	02	0
001	02	20	0
001	02	22	$\mathbb{1}$
001	20	00	$\mathbb{1}$
001	20	02	0
001	20	20	0
001	20	22	$\mathbb{1}$
001	22	00	0
001	22	02	$\mathbb{1}$
001	22	20	$\mathbb{1}$
001	22	22	0
010	11	00	1
010	11	02	0
010	11	20	0
010	11	22	1
010	$1\bar{1}$	00	0
010	$1\bar{1}$	02	1
010	$1\bar{1}$	20	1
010	$1\bar{1}$	22	0
010	$\bar{1}1$	00	0
010	$\bar{1}1$	02	1
010	$\bar{1}1$	20	1
010	$\bar{1}1$	22	0
010	$\bar{1}\bar{1}$	00	1
010	$\bar{1}\bar{1}$	02	0
010	$\bar{1}\bar{1}$	20	0
010	$\bar{1}\bar{1}$	22	1
011	11	00	1
011	11	02	0
011	11	20	0
011	11	22	1
011	$1\bar{1}$	00	0
011	$1\bar{1}$	02	1

Continued on next column

Supplementary Table 8 – continued

Invariants			
011	$1\bar{1}$	20	1
011	$1\bar{1}$	22	0
011	$\bar{1}1$	00	0
011	$\bar{1}1$	02	1
011	$\bar{1}1$	20	1
011	$\bar{1}1$	22	0
011	$\bar{1}\bar{1}$	00	1
011	$\bar{1}\bar{1}$	02	0
011	$\bar{1}\bar{1}$	20	0
011	$\bar{1}\bar{1}$	22	1
100	00	11	1
100	00	$1\bar{1}$	0
100	00	$\bar{1}1$	0
100	00	$\bar{1}\bar{1}$	1
100	02	11	0
100	02	$1\bar{1}$	1
100	02	$\bar{1}1$	1
100	02	$\bar{1}\bar{1}$	0
100	20	11	0
100	20	$1\bar{1}$	1
100	20	$\bar{1}1$	1
100	20	$\bar{1}\bar{1}$	0
100	22	11	1
100	22	$1\bar{1}$	0
100	22	$\bar{1}1$	0
100	22	$\bar{1}\bar{1}$	1
101	00	11	1
101	00	$1\bar{1}$	0
101	00	$\bar{1}1$	0
101	00	$\bar{1}\bar{1}$	1
101	02	11	0
101	02	$1\bar{1}$	1
101	02	$\bar{1}1$	1
101	02	$\bar{1}\bar{1}$	0
101	20	11	0
101	20	$1\bar{1}$	1
101	20	$\bar{1}1$	1
101	20	$\bar{1}\bar{1}$	0
101	22	11	1
101	22	$1\bar{1}$	0
101	22	$\bar{1}1$	0
101	22	$\bar{1}\bar{1}$	1
110	11	11	0
110	11	$1\bar{1}$	1
110	11	$\bar{1}1$	1
110	11	$\bar{1}\bar{1}$	0
110	$1\bar{1}$	11	1
110	$1\bar{1}$	$1\bar{1}$	0
110	$1\bar{1}$	$\bar{1}1$	0
110	$1\bar{1}$	$\bar{1}\bar{1}$	1
110	$\bar{1}1$	11	1
110	$\bar{1}1$	$1\bar{1}$	0
110	$\bar{1}1$	$\bar{1}1$	0
110	$\bar{1}1$	$\bar{1}\bar{1}$	1
110	$\bar{1}\bar{1}$	11	0
110	$\bar{1}\bar{1}$	$1\bar{1}$	1
110	$\bar{1}\bar{1}$	$\bar{1}1$	1
110	$\bar{1}\bar{1}$	$\bar{1}\bar{1}$	0
111	11	11	0
111	11	$1\bar{1}$	1
111	11	$\bar{1}1$	1

Continued on next column

Supplementary Table 8 – continued

Invariants			
111	11	$\bar{1}\bar{1}$	0
111	$1\bar{1}$	11	1
111	$1\bar{1}$	$1\bar{1}$	0
111	$1\bar{1}$	$\bar{1}1$	0
111	$1\bar{1}$	$\bar{1}\bar{1}$	1
111	$\bar{1}1$	11	1
111	$\bar{1}1$	$1\bar{1}$	0
111	$\bar{1}1$	$\bar{1}1$	0
111	$\bar{1}1$	$\bar{1}\bar{1}$	1
111	$\bar{1}\bar{1}$	11	0
111	$\bar{1}\bar{1}$	$1\bar{1}$	1
111	$\bar{1}\bar{1}$	$\bar{1}1$	1
111	$\bar{1}\bar{1}$	$\bar{1}\bar{1}$	0

Space group #26 : $Pmc2_1$

weak	$m^{100}_{(2)}$	$g^{010}_{00\frac{1}{2}}$	2^{001}_1
000	00	0	0
000	00	$\mathbb{1}$	$\mathbb{1}$
000	02	0	$\mathbb{1}$
000	02	$\mathbb{1}$	0
000	20	0	$\mathbb{1}$
000	20	$\mathbb{1}$	0
000	22	0	0
000	22	$\mathbb{1}$	$\mathbb{1}$
010	00	0	0
010	00	1	1
010	02	0	1
010	02	1	0
010	20	0	1
010	20	1	0
010	22	0	0
010	22	1	1
100	11	0	1
100	11	$\mathbb{1}$	0
100	$1\bar{1}$	0	0
100	$1\bar{1}$	$\mathbb{1}$	1
100	$\bar{1}1$	0	0
100	$\bar{1}1$	$\mathbb{1}$	1
100	$\bar{1}\bar{1}$	0	1
100	$\bar{1}\bar{1}$	$\mathbb{1}$	0
110	11	0	1
110	11	1	0
110	$1\bar{1}$	0	0
110	$1\bar{1}$	1	1
110	$\bar{1}1$	0	0
110	$\bar{1}1$	1	1
110	$\bar{1}\bar{1}$	0	1
110	$\bar{1}\bar{1}$	1	0

Space group #27 : $Pcc2$

weak	$g^{010}_{00\frac{1}{2}}$	$g^{100}_{00\frac{1}{2}}$	2^{001}
000	0	0	0
000	0	$\mathbb{1}$	$\mathbb{1}$
000	$\mathbb{1}$	0	$\mathbb{1}$
000	$\mathbb{1}$	$\mathbb{1}$	0
010	0	0	0
010	0	$\mathbb{1}$	1
010	1	0	1
010	1	$\mathbb{1}$	0
100	0	0	0
100	0	1	1

Continued on next column

Supplementary Table 8 – continued

Invariants			
100	$\mathbb{1}$	0	1
100	$\mathbb{1}$	1	0
110	0	0	0
110	0	1	1
110	1	0	1
110	1	1	0

Space group #28 : $Pma2$

weak	$m^{100}_{(2)}$	$g^{010}_{\frac{1}{2}00}$	2^{001}
000	00	0	0
000	00	$\mathbb{1}$	$\mathbb{1}$
000	20	0	$\mathbb{1}$
000	20	$\mathbb{1}$	0
001	00	0	0
001	00	$\mathbb{1}$	$\mathbb{1}$
001	20	0	$\mathbb{1}$
001	20	$\mathbb{1}$	0
010	00	0	0
010	00	1	1
010	20	0	1
010	20	1	0
011	00	0	0
011	00	1	1
011	20	0	1
011	20	1	0

Space group #29 : $Pca2_1$

weak	$g^{010}_{\frac{1}{2}00}$	$g^{100}_{00\frac{1}{2}}$	2^{001}_1
000	0	0	0
000	0	$\mathbb{1}$	$\mathbb{1}$
000	$\mathbb{1}$	0	$\mathbb{1}$
000	$\mathbb{1}$	$\mathbb{1}$	0
010	0	0	0
010	0	$\mathbb{1}$	1
010	1	0	1
010	1	$\mathbb{1}$	0

Space group #30 : $Pnc2$

weak	$g^{010}_{00\frac{1}{2}}$	$g^{100}_{0\frac{1}{2}\frac{1}{2}}$	2^{001}
000	0	0	0
000	0	$\mathbb{1}$	$\mathbb{1}$
000	$\mathbb{1}$	0	$\mathbb{1}$
000	$\mathbb{1}$	$\mathbb{1}$	0
100	0	0	0
100	0	1	1
100	$\mathbb{1}$	0	1
100	$\mathbb{1}$	1	0

Space group #31 : $Pmn2_1$

weak	$m^{100}_{(2)}$	$g^{010}_{\frac{1}{2}0\frac{1}{2}}$	2^{001}_1
000	00	0	0
000	00	$\mathbb{1}$	$\mathbb{1}$
000	20	0	$\mathbb{1}$
000	20	$\mathbb{1}$	0
010	00	0	0
010	00	1	1
010	20	0	1
010	20	1	0

Space group #32 : $Pba2$

weak	$g^{010}_{\frac{1}{2}00}$	$g^{100}_{0\frac{1}{2}0}$	2^{001}
000	0	0	0

Continued on next column

Supplementary Table 8 – continued

Invariants			
000	0	$\mathbb{1}$	$\mathbb{1}$
000	$\mathbb{1}$	0	$\mathbb{1}$
000	$\mathbb{1}$	$\mathbb{1}$	0
001	0	0	0
001	0	$\mathbb{1}$	$\mathbb{1}$
001	$\mathbb{1}$	0	$\mathbb{1}$
001	$\mathbb{1}$	$\mathbb{1}$	0

Space group #33 : $Pna2_1$

weak	$g^{010}_{\frac{1}{2}00}$	$g^{100}_{0\frac{1}{2}\frac{1}{2}}$	2^{001}_1
000	0	0	0
000	0	$\mathbb{1}$	$\mathbb{1}$
000	$\mathbb{1}$	0	$\mathbb{1}$
000	$\mathbb{1}$	$\mathbb{1}$	0

Space group #34 : $Pnn2$

weak	$g^{010}_{\frac{1}{2}0\frac{1}{2}}$	$g^{100}_{0\frac{1}{2}\frac{1}{2}}$	2^{001}
000	0	0	0
000	0	$\mathbb{1}$	$\mathbb{1}$
000	$\mathbb{1}$	0	$\mathbb{1}$
000	$\mathbb{1}$	$\mathbb{1}$	0

Space group #35 : $Cmm2$

weak	$m^{010}_{(2)}$	$m^{100}_{(2)}$	$g^{010}_{\frac{1}{2}00}$	$g^{100}_{0\frac{1}{2}0}$	2^{001}
000	0	0	0	0	0
000	0	2	0	$\mathbb{1}$	$\mathbb{1}$
000	2	0	$\mathbb{1}$	0	$\mathbb{1}$
000	2	2	$\mathbb{1}$	$\mathbb{1}$	0
001	0	0	0	0	0
001	0	2	0	$\mathbb{1}$	$\mathbb{1}$
001	2	0	$\mathbb{1}$	0	$\mathbb{1}$
001	2	2	$\mathbb{1}$	$\mathbb{1}$	0
110	0	0	$\mathbb{1}$	$\mathbb{1}$	0
110	0	2	$\mathbb{1}$	0	1
110	2	0	0	$\mathbb{1}$	1
110	2	2	0	0	0
111	0	0	$\mathbb{1}$	$\mathbb{1}$	0
111	0	2	$\mathbb{1}$	0	1
111	2	0	0	$\mathbb{1}$	1
111	2	2	0	0	0

Space group #36 : $Cmc2_1$

weak	$m^{100}_{(2)}$	$g^{010}_{00\frac{1}{2}}$	$g^{010}_{\frac{1}{2}0\frac{1}{2}}$	$g^{100}_{0\frac{1}{2}0}$	2^{001}_1
000	0	0	0	0	0
000	0	$\mathbb{1}$	$\mathbb{1}$	0	$\mathbb{1}$
000	2	0	0	$\mathbb{1}$	$\mathbb{1}$
000	2	$\mathbb{1}$	$\mathbb{1}$	$\mathbb{1}$	0
110	0	0	$\mathbb{1}$	$\mathbb{1}$	0
110	0	$\mathbb{1}$	0	$\mathbb{1}$	1
110	2	0	$\mathbb{1}$	0	1
110	2	$\mathbb{1}$	0	0	0

Space group #37 : $Ccc2$

weak	$g^{010}_{00\frac{1}{2}}$	$g^{010}_{\frac{1}{2}0\frac{1}{2}}$	$g^{100}_{00\frac{1}{2}}$	$g^{100}_{0\frac{1}{2}\frac{1}{2}}$	2^{001}
000	0	0	0	0	0
000	0	0	$\mathbb{1}$	$\mathbb{1}$	$\mathbb{1}$
000	$\mathbb{1}$	$\mathbb{1}$	0	0	$\mathbb{1}$
000	$\mathbb{1}$	$\mathbb{1}$	$\mathbb{1}$	$\mathbb{1}$	0
110	0	$\mathbb{1}$	0	$\mathbb{1}$	0
110	0	$\mathbb{1}$	$\mathbb{1}$	0	1
110	$\mathbb{1}$	0	0	$\mathbb{1}$	1
110	$\mathbb{1}$	0	$\mathbb{1}$	0	0

Continued on next column

Supplementary Table 8 – continued

Invariants

Space group #38 : $Amm2$

weak	$m^{010}_{(2)}$	$m^{100}_{(2)}$	$g^{010}_{00\frac{1}{2}}$	2^{001}	2^{001}_1
000	0	00	0	0	0
000	0	02	0	$\mathbb{1}$	$\mathbb{1}$
000	0	20	0	$\mathbb{1}$	$\mathbb{1}$
000	0	22	0	0	0
000	2	00	$\mathbb{1}$	$\mathbb{1}$	$\mathbb{1}$
000	2	02	$\mathbb{1}$	0	0
000	2	20	$\mathbb{1}$	0	0
000	2	22	$\mathbb{1}$	$\mathbb{1}$	$\mathbb{1}$
011	0	00	$\mathbb{1}$	0	$\mathbb{1}$
011	0	02	$\mathbb{1}$	$\mathbb{1}$	0
011	0	20	$\mathbb{1}$	$\mathbb{1}$	0
011	0	22	$\mathbb{1}$	0	$\mathbb{1}$
011	2	00	0	$\mathbb{1}$	0
011	2	02	0	0	$\mathbb{1}$
011	2	20	0	0	$\mathbb{1}$
011	2	22	0	$\mathbb{1}$	0
100	0	11	0	1	1
100	0	$1\bar{1}$	0	0	0
100	0	$\bar{1}1$	0	0	0
100	0	$\bar{1}\bar{1}$	0	1	1
100	2	11	$\mathbb{1}$	0	0
100	2	$1\bar{1}$	$\mathbb{1}$	1	1
100	2	$\bar{1}1$	$\mathbb{1}$	1	1
100	2	$\bar{1}\bar{1}$	$\mathbb{1}$	0	0
111	0	11	$\mathbb{1}$	1	0
111	0	$1\bar{1}$	$\mathbb{1}$	0	1
111	0	$\bar{1}1$	$\mathbb{1}$	0	1
111	0	$\bar{1}\bar{1}$	$\mathbb{1}$	1	0
111	2	11	0	0	1
111	2	$1\bar{1}$	0	1	0
111	2	$\bar{1}1$	0	1	0
111	2	$\bar{1}\bar{1}$	0	0	1

Space group #39 : $Aem2$

weak	$m^{010}_{(2)}$	$g^{010}_{00\frac{1}{2}}$	$g^{100}_{00\frac{1}{2}}$	2^{001}	2^{001}_1
000	0	0	0	0	0
000	0	0	$\mathbb{1}$	$\mathbb{1}$	$\mathbb{1}$
000	2	$\mathbb{1}$	0	$\mathbb{1}$	$\mathbb{1}$
000	2	$\mathbb{1}$	$\mathbb{1}$	0	0
011	0	$\mathbb{1}$	0	$\mathbb{1}$	0
011	0	$\mathbb{1}$	$\mathbb{1}$	0	$\mathbb{1}$
011	2	0	0	0	$\mathbb{1}$
011	2	0	$\mathbb{1}$	$\mathbb{1}$	0
100	0	0	0	0	0
100	0	0	1	1	1
100	2	$\mathbb{1}$	0	1	1
100	2	$\mathbb{1}$	1	0	0
111	0	$\mathbb{1}$	0	1	0
111	0	$\mathbb{1}$	1	0	1
111	2	0	0	0	1
111	2	0	1	1	0

Space group #40 : $Ama2$

weak	$m^{100}_{(2)}$	$g^{010}_{\frac{1}{2}00}$	$g^{010}_{\frac{1}{2}0\frac{1}{2}}$	2^{001}	2^{001}_1
000	00	0	0	0	0
000	00	$\mathbb{1}$	$\mathbb{1}$	$\mathbb{1}$	$\mathbb{1}$
000	20	0	0	$\mathbb{1}$	$\mathbb{1}$
000	20	$\mathbb{1}$	$\mathbb{1}$	0	0
011	00	0	$\mathbb{1}$	0	$\mathbb{1}$
011	00	$\mathbb{1}$	0	$\mathbb{1}$	0
011	20	0	$\mathbb{1}$	$\mathbb{1}$	0
011	20	$\mathbb{1}$	0	0	$\mathbb{1}$

Continued on next column

Supplementary Table 8 – continued

Invariants

Space group #41 : $Aea2$

weak	$g^{010}_{\frac{1}{2}00}$	$g^{010}_{\frac{1}{2}0\frac{1}{2}}$	$g^{100}_{00\frac{1}{2}}$	2^{001}	2^{001}_1
000	0	0	0	0	0
000	0	0	$\mathbb{1}$	$\mathbb{1}$	$\mathbb{1}$
000	$\mathbb{1}$	$\mathbb{1}$	0	$\mathbb{1}$	$\mathbb{1}$
000	$\mathbb{1}$	$\mathbb{1}$	$\mathbb{1}$	0	0
011	0	$\mathbb{1}$	0	$\mathbb{1}$	0
011	0	$\mathbb{1}$	$\mathbb{1}$	0	$\mathbb{1}$
011	$\mathbb{1}$	0	0	0	$\mathbb{1}$
011	$\mathbb{1}$	0	$\mathbb{1}$	$\mathbb{1}$	0

Space group #42 : $Fmm2$

weak	$m^{010}_{(2)}$	$m^{100}_{(2)}$	$g^{010}_{\frac{1}{2}00}$	$g^{100}_{0\frac{1}{2}0}$	2^{001}	2^{001}_1
000	0	0	0	0	0	0
000	0	2	0	$\mathbb{1}$	$\mathbb{1}$	$\mathbb{1}$
000	2	0	$\mathbb{1}$	0	$\mathbb{1}$	$\mathbb{1}$
000	2	2	$\mathbb{1}$	$\mathbb{1}$	0	0
011	0	0	$\mathbb{1}$	$\mathbb{1}$	0	0
011	0	2	$\mathbb{1}$	0	1	1
011	2	0	0	$\mathbb{1}$	1	1
011	2	2	0	0	0	0
101	0	0	$\mathbb{1}$	$\mathbb{1}$	0	1
101	0	2	$\mathbb{1}$	0	1	0
101	2	0	0	$\mathbb{1}$	1	0
101	2	2	0	0	0	1
110	0	0	0	0	0	$\mathbb{1}$
110	0	2	0	$\mathbb{1}$	$\mathbb{1}$	0
110	2	0	$\mathbb{1}$	0	$\mathbb{1}$	0
110	2	2	$\mathbb{1}$	$\mathbb{1}$	0	$\mathbb{1}$

Space group #43 : $Fdd2$

weak	$g^{010}_{\frac{1}{4}0\frac{\bar{1}}{4}}$	$g^{010}_{\frac{1}{4}0\frac{1}{4}}$	$g^{100}_{0\frac{1}{4}\frac{1}{4}}$	$g^{100}_{0\frac{1}{4}\frac{\bar{1}}{4}}$	2^{001}	2^{001}_1
000	0	0	0	0	0	0
000	0	0	$\mathbb{1}$	$\mathbb{1}$	$\mathbb{1}$	$\mathbb{1}$
000	$\mathbb{1}$	$\mathbb{1}$	0	0	$\mathbb{1}$	$\mathbb{1}$
000	$\mathbb{1}$	$\mathbb{1}$	$\mathbb{1}$	$\mathbb{1}$	0	0

Space group #44 : $Imm2$

weak	$m^{010}_{(2)}$	$m^{100}_{(2)}$	$g^{010}_{\frac{1}{2}0\frac{1}{2}}$	$g^{100}_{0\frac{1}{2}\frac{1}{2}}$	2^{001}	2^{001}_1
000	0	0	0	0	0	0
000	0	2	0	$\mathbb{1}$	$\mathbb{1}$	$\mathbb{1}$
000	2	0	$\mathbb{1}$	0	$\mathbb{1}$	$\mathbb{1}$
000	2	2	$\mathbb{1}$	$\mathbb{1}$	0	0
111	0	0	$\mathbb{1}$	$\mathbb{1}$	0	$\mathbb{1}$
111	0	2	$\mathbb{1}$	0	$\mathbb{1}$	0
111	2	0	0	$\mathbb{1}$	$\mathbb{1}$	0
111	2	2	0	0	0	$\mathbb{1}$

Space group #45 : $Iba2$

weak	$g^{010}_{00\frac{1}{2}}$	$g^{010}_{\frac{1}{2}00}$	$g^{100}_{00\frac{1}{2}}$	$g^{100}_{0\frac{1}{2}0}$	2^{001}	2^{001}_1
000	0	0	0	0	0	0
000	0	0	$\mathbb{1}$	$\mathbb{1}$	$\mathbb{1}$	$\mathbb{1}$
000	$\mathbb{1}$	$\mathbb{1}$	0	0	$\mathbb{1}$	$\mathbb{1}$
000	$\mathbb{1}$	$\mathbb{1}$	$\mathbb{1}$	$\mathbb{1}$	0	0
111	0	$\mathbb{1}$	0	$\mathbb{1}$	0	$\mathbb{1}$
111	0	$\mathbb{1}$	$\mathbb{1}$	0	$\mathbb{1}$	0
111	$\mathbb{1}$	0	0	$\mathbb{1}$	$\mathbb{1}$	0
111	$\mathbb{1}$	0	$\mathbb{1}$	0	0	$\mathbb{1}$

Continued on next column

Supplementary Table 8 – continued

Invariants

Space group #46 : $Ima2$

weak	$m^{100}_{(2)}$	$g^{010}_{00\frac{1}{2}}$	$g^{010}_{\frac{1}{2}00}$	$g^{100}_{0\frac{1}{2}\frac{1}{2}}$	2^{001}	2^{001}_1
000	0	0	0	0	0	0
000	0	$\mathbb{1}$	$\mathbb{1}$	0	$\mathbb{1}$	$\mathbb{1}$
000	2	0	0	$\mathbb{1}$	$\mathbb{1}$	$\mathbb{1}$
000	2	$\mathbb{1}$	$\mathbb{1}$	$\mathbb{1}$	0	0
111	0	0	$\mathbb{1}$	$\mathbb{1}$	$\mathbb{1}$	0
111	0	$\mathbb{1}$	0	$\mathbb{1}$	0	$\mathbb{1}$
111	2	0	$\mathbb{1}$	0	0	$\mathbb{1}$
111	2	$\mathbb{1}$	0	0	$\mathbb{1}$	0

Space group #75 : $P4$

weak	2^{001}	4^{001}
000	0	0
000	0	$\mathbb{1}$
001	0	0
001	0	$\mathbb{1}$
110	0	0
110	0	1
111	0	0
111	0	1

Space group #76 : $P4_1$

weak	2^{001}_1	4^{001}_1
000	0	0
000	0	$\mathbb{1}$
110	0	0
110	0	1

Space group #77 : $P4_2$

weak	2^{001}	4^{001}_2
000	0	0
000	0	$\mathbb{1}$
110	0	0
110	0	1

Space group #78 : $P4_3$

weak	2^{001}_1	4^{001}_3
000	0	0
000	0	$\mathbb{1}$
110	0	0
110	0	1

Space group #79 : $I4$

weak	2^{001}	4^{001}	2^{001}_1	4^{001}_2
000	0	0	0	0
000	0	$\mathbb{1}$	0	$\mathbb{1}$
111	0	0	$\mathbb{1}$	$\mathbb{1}$
111	0	$\mathbb{1}$	$\mathbb{1}$	0

Space group #80 : $I4_1$

weak	2^{001}	2^{001}_1	4^{001}_1	4^{001}_3
000	0	0	0	0
000	0	0	$\mathbb{1}$	$\mathbb{1}$
111	$\mathbb{1}$	0	0	$\mathbb{1}$
111	$\mathbb{1}$	0	$\mathbb{1}$	0

Space group #81 : $P\bar{4}$

weak	2^{001}	$\bar{4}^{001}$
000	0	0
000	0	$\mathbb{1}$
001	0	0
001	0	1
110	0	0
110	0	1
111	0	0
111	0	1

Space group #82 : $I\bar{4}$

weak	2^{001}	2^{001}_1	$\bar{4}^{001}$
000	0	0	0
000	0	0	$\mathbb{1}$
111	0	$\mathbb{1}$	0
111	0	$\mathbb{1}$	1

Space group #89 : $P422$

weak	2^{001}	2^{100}	2^{110}	4^{001}	2^{110}_1
000	0	0	0	0	0
000	0	0	$\mathbb{1}$	$\mathbb{1}$	$\mathbb{1}$
000	0	$\mathbb{1}$	0	$\mathbb{1}$	0
000	0	$\mathbb{1}$	$\mathbb{1}$	0	$\mathbb{1}$
001	0	0	0	0	1
001	0	0	1	$\mathbb{1}$	0
001	0	1	0	$\mathbb{1}$	1
001	0	1	1	0	0
110	0	0	0	0	$\mathbb{1}$
110	0	0	$\mathbb{1}$	1	0
110	0	1	0	1	$\mathbb{1}$
110	0	1	$\mathbb{1}$	0	0
111	0	0	0	0	0
111	0	0	1	1	1
111	0	1	0	1	0
111	0	1	1	0	1

Space group #90 : $P42_12$

weak	2^{001}	2^{110}	4^{001}	2^{100}_1	2^{110}_1
000	0	0	0	0	0
000	0	0	$\mathbb{1}$	$\mathbb{1}$	0
000	0	$\mathbb{1}$	0	$\mathbb{1}$	$\mathbb{1}$
000	0	$\mathbb{1}$	$\mathbb{1}$	0	$\mathbb{1}$
001	0	0	0	0	1
001	0	0	$\mathbb{1}$	1	1
001	0	1	0	1	0
001	0	1	$\mathbb{1}$	0	0

Space group #91 : $P4_122$

weak	2^{100}	2^{110}	2^{001}_1	2^{110}_1	4^{001}_1
000	0	0	0	0	0
000	0	$\mathbb{1}$	0	$\mathbb{1}$	$\mathbb{1}$
000	$\mathbb{1}$	0	0	0	$\mathbb{1}$
000	$\mathbb{1}$	$\mathbb{1}$	0	$\mathbb{1}$	0
110	0	0	0	$\mathbb{1}$	0
110	0	$\mathbb{1}$	0	0	1
110	1	0	0	$\mathbb{1}$	1
110	1	$\mathbb{1}$	0	0	0

Space group #92 : $P4_12_12$

weak	2^{110}	2^{001}_1	2^{100}_1	2^{110}_1	4^{001}_1
000	0	0	0	0	0
000	0	0	$\mathbb{1}$	0	$\mathbb{1}$
000	$\mathbb{1}$	0	0	$\mathbb{1}$	$\mathbb{1}$
000	$\mathbb{1}$	0	$\mathbb{1}$	$\mathbb{1}$	0

Space group #93 : $P4_222$

weak	2^{001}	2^{100}	2^{110}	2^{110}_1	4^{001}_2
000	0	0	0	0	0
000	0	0	$\mathbb{1}$	$\mathbb{1}$	$\mathbb{1}$
000	0	$\mathbb{1}$	0	0	$\mathbb{1}$

Continued on next column

Supplementary Table 8 – continued

Invariants					
000	0	𝟙	𝟙	𝟙	0
110	0	0	0	𝟙	0
110	0	0	𝟙	0	1
110	0	1	0	𝟙	1
110	0	1	𝟙	0	0

Space group #94 : $P4_22_12$

weak	2^{001}	2^{110}	2_1^{100}	2_1^{110}	4_2^{001}
000	0	0	0	0	0
000	0	0	𝟙	0	𝟙
000	0	𝟙	0	𝟙	𝟙
000	0	𝟙	𝟙	𝟙	0

Space group #95 : $P4_322$

weak	2^{100}	2^{110}	2_1^{001}	2_1^{110}	4_3^{001}
000	0	0	0	0	0
000	0	𝟙	0	𝟙	𝟙
000	𝟙	0	0	0	𝟙
000	𝟙	𝟙	0	𝟙	0
110	0	0	0	𝟙	0
110	0	𝟙	0	0	1
110	1	0	0	𝟙	1
110	1	𝟙	0	0	0

Space group #96 : $P4_32_12$

weak	2^{110}	2_1^{001}	2_1^{100}	2_1^{110}	4_3^{001}
000	0	0	0	0	0
000	0	0	𝟙	0	𝟙
000	𝟙	0	0	𝟙	𝟙
000	𝟙	0	𝟙	𝟙	0

Space group #97 : $I422$

weak	2^{001}	2^{100}	2^{110}	4^{001}	2_1^{001}	2_1^{100}	2_1^{110}	4_2^{001}
000	0	0	0	0	0	0	0	0
000	0	0	𝟙	𝟙	0	0	𝟙	𝟙
000	0	𝟙	0	𝟙	0	𝟙	0	𝟙
000	0	𝟙	𝟙	0	0	𝟙	𝟙	0
111	0	0	0	0	𝟙	𝟙	1	𝟙
111	0	0	1	𝟙	𝟙	𝟙	0	0
111	0	𝟙	0	𝟙	𝟙	0	1	0
111	0	𝟙	1	0	𝟙	0	0	𝟙

Space group #98 : $I4_122$

weak	2^{001}	2^{100}	2^{110}	2_1^{001}	2_1^{100}	2_1^{110}	4_1^{001}	4_3^{001}
000	0	0	0	0	0	0	0	0
000	0	0	𝟙	0	0	𝟙	𝟙	𝟙
000	0	𝟙	0	0	𝟙	0	𝟙	𝟙
000	0	𝟙	𝟙	0	𝟙	𝟙	0	0
111	𝟙	0	0	0	𝟙	1	𝟙	0
111	𝟙	0	1	0	𝟙	0	0	𝟙
111	𝟙	𝟙	0	0	0	1	0	𝟙
111	𝟙	𝟙	1	0	0	0	𝟙	0

Space group #99 : $P4mm$

weak	$m_{(2)}^{100}$	$m_{(2)}^{110}$	$g^{110}_{\frac12\frac12 0}$	2^{001}	4^{001}
000	00	0	0	0	0
000	00	2	𝟙	0	𝟙
000	02	0	0	0	𝟙
000	02	2	𝟙	0	0
000	20	0	0	0	𝟙
000	20	2	𝟙	0	0
000	22	0	0	0	0
000	22	2	𝟙	0	𝟙
001	00	0	0	0	0

Continued on next column

Supplementary Table 8 – continued

Invariants					
001	00	2	𝟙	0	𝟙
001	02	0	0	0	𝟙
001	02	2	𝟙	0	0
001	20	0	0	0	𝟙
001	20	2	𝟙	0	0
001	22	0	0	0	0
001	22	2	𝟙	0	𝟙
110	11	0	𝟙	0	1
110	11	2	0	0	0
110	$1\bar1$	0	𝟙	0	0
110	$1\bar1$	2	0	0	1
110	$\bar11$	0	𝟙	0	0
110	$\bar11$	2	0	0	1
110	$\bar1\bar1$	0	𝟙	0	1
110	$\bar1\bar1$	2	0	0	0
111	11	0	𝟙	0	1
111	11	2	0	0	0
111	$1\bar1$	0	𝟙	0	0
111	$1\bar1$	2	0	0	1
111	$\bar11$	0	𝟙	0	0
111	$\bar11$	2	0	0	1
111	$\bar1\bar1$	0	𝟙	0	1
111	$\bar1\bar1$	2	0	0	0

Space group #100 : $P4bm$

weak	$m_{(2)}^{110}$	$g^{100}_{0\frac12 0}$	$g^{110}_{\frac12\frac12 0}$	2^{001}	4^{001}
000	0	0	0	0	0
000	0	𝟙	0	0	𝟙
000	2	0	𝟙	0	𝟙
000	2	𝟙	𝟙	0	0
001	0	0	0	0	0
001	0	𝟙	0	0	𝟙
001	2	0	𝟙	0	𝟙
001	2	𝟙	𝟙	0	0

Space group #101 : $P4_2cm$

weak	$m_{(2)}^{110}$	$g^{100}_{00\frac12}$	$g^{110}_{\frac12\frac12 0}$	2^{001}	4_2^{001}
000	0	0	0	0	0
000	0	𝟙	0	0	𝟙
000	2	0	𝟙	0	𝟙
000	2	𝟙	𝟙	0	0
110	0	0	𝟙	0	0
110	0	1	𝟙	0	1
110	2	0	0	0	1
110	2	1	0	0	0

Space group #102 : $P4_2cm$

weak	$m_{(2)}^{110}$	$g^{100}_{0\frac12\frac12}$	$g^{110}_{\frac12\frac12 0}$	2^{001}	4_2^{001}
000	0	0	0	0	0
000	0	𝟙	0	0	𝟙
000	2	0	𝟙	0	𝟙
000	2	𝟙	𝟙	0	0

Space group #103 : $P4cc$

weak	$g^{100}_{00\frac12}$	$g^{110}_{00\frac12}$	$g^{110}_{\frac12\frac12\frac12}$	2^{001}	4^{001}
000	0	0	0	0	0
000	0	𝟙	𝟙	0	𝟙
000	𝟙	0	0	0	𝟙
000	𝟙	𝟙	𝟙	0	0
110	0	0	𝟙	0	0
110	0	𝟙	0	0	1
110	1	0	𝟙	0	1

Continued on next column

Supplementary Table 8 – continued

Invariants

110	1	$\mathbb{1}$	0	0	0

Space group #104 : $P4nc$

weak	$g^{100}_{0\frac{1}{2}\frac{1}{2}}$	$g^{110}_{00\frac{1}{2}}$	$g^{110}_{\frac{\bar{1}}{2}\frac{1}{2}\frac{1}{2}}$	2^{001}	4^{001}
000	0	0	0	0	0
000	0	$\mathbb{1}$	$\mathbb{1}$	0	$\mathbb{1}$
000	$\mathbb{1}$	0	0	0	$\mathbb{1}$
000	$\mathbb{1}$	$\mathbb{1}$	$\mathbb{1}$	0	0

Space group #105 : $P4_2mc$

weak	$m^{100}_{(2)}$	$g^{110}_{00\frac{1}{2}}$	$g^{110}_{\frac{\bar{1}}{2}\frac{1}{2}\frac{1}{2}}$	2^{001}	4_2^{001}
000	00	0	0	0	0
000	00	$\mathbb{1}$	$\mathbb{1}$	0	$\mathbb{1}$
000	02	0	0	0	$\mathbb{1}$
000	02	$\mathbb{1}$	$\mathbb{1}$	0	0
000	20	0	0	0	$\mathbb{1}$
000	20	$\mathbb{1}$	$\mathbb{1}$	0	0
000	22	0	0	0	0
000	22	$\mathbb{1}$	$\mathbb{1}$	0	$\mathbb{1}$
110	11	0	$\mathbb{1}$	0	1
110	11	$\mathbb{1}$	0	0	0
110	$1\bar{1}$	0	$\mathbb{1}$	0	0
110	$1\bar{1}$	$\mathbb{1}$	0	0	1
110	$\bar{1}1$	0	$\mathbb{1}$	0	0
110	$\bar{1}1$	$\mathbb{1}$	0	0	1
110	$\bar{1}\bar{1}$	0	$\mathbb{1}$	0	1
110	$\bar{1}\bar{1}$	$\mathbb{1}$	0	0	0

Space group #106 : $P4_2bc$

weak	$g^{100}_{0\frac{1}{2}0}$	$g^{110}_{00\frac{1}{2}}$	$g^{110}_{\frac{\bar{1}}{2}\frac{1}{2}\frac{1}{2}}$	2^{001}	4_2^{001}
000	0	0	0	0	0
000	0	$\mathbb{1}$	$\mathbb{1}$	0	$\mathbb{1}$
000	$\mathbb{1}$	0	0	0	$\mathbb{1}$
000	$\mathbb{1}$	$\mathbb{1}$	$\mathbb{1}$	0	0

Space group #107 : $I4mm$

weak	$m^{100}_{(2)}$	$m^{110}_{(2)}$	$g^{100}_{0\frac{1}{2}\frac{1}{2}}$	$g^{110}_{00\frac{1}{2}}$	2^{001}	4^{001}	2_1^{001}	4_2^{001}
000	0	0	0	0	0	0	0	0
000	0	2	0	$\mathbb{1}$	0	$\mathbb{1}$	0	$\mathbb{1}$
000	2	0	$\mathbb{1}$	0	0	$\mathbb{1}$	0	$\mathbb{1}$
000	2	2	$\mathbb{1}$	$\mathbb{1}$	0	0	0	0
111	0	0	$\mathbb{1}$	$\mathbb{1}$	0	0	$\mathbb{1}$	$\mathbb{1}$
111	0	2	$\mathbb{1}$	0	0	$\mathbb{1}$	$\mathbb{1}$	0
111	2	0	0	$\mathbb{1}$	0	$\mathbb{1}$	$\mathbb{1}$	0
111	2	2	0	0	0	0	$\mathbb{1}$	$\mathbb{1}$

Space group #108 : $I4cm$

weak	$m^{110}_{(2)}$	$g^{100}_{00\frac{1}{2}}$	$g^{100}_{0\frac{1}{2}0}$	$g^{110}_{00\frac{1}{2}}$	2^{001}	4^{001}	2_1^{001}	4_2^{001}
000	0	0	0	0	0	0	0	0
000	0	$\mathbb{1}$	$\mathbb{1}$	0	0	$\mathbb{1}$	0	$\mathbb{1}$
000	2	0	0	$\mathbb{1}$	0	$\mathbb{1}$	0	$\mathbb{1}$
000	2	$\mathbb{1}$	$\mathbb{1}$	$\mathbb{1}$	0	0	0	0
111	0	0	$\mathbb{1}$	$\mathbb{1}$	0	$\mathbb{1}$	$\mathbb{1}$	0
111	0	$\mathbb{1}$	0	$\mathbb{1}$	0	0	$\mathbb{1}$	$\mathbb{1}$
111	2	0	$\mathbb{1}$	0	0	0	$\mathbb{1}$	$\mathbb{1}$
111	2	$\mathbb{1}$	0	0	0	$\mathbb{1}$	$\mathbb{1}$	0

Space group #109 : $I4_1md$

weak	$m^{100}_{(2)}$	$g^{100}_{0\frac{1}{2}\frac{1}{2}}$	$g^{110}_{\frac{1}{4}\frac{\bar{1}}{4}\frac{1}{4}}$	$g^{110}_{\frac{\bar{1}}{4}\frac{1}{4}\frac{1}{4}}$	2^{001}	2_1^{001}	4_1^{001}	4_3^{001}
000	0	0	0	0	0	0	0	0
000	0	0	$\mathbb{1}$	$\mathbb{1}$	0	0	$\mathbb{1}$	$\mathbb{1}$
000	2	$\mathbb{1}$	0	0	0	0	$\mathbb{1}$	$\mathbb{1}$
000	2	$\mathbb{1}$	$\mathbb{1}$	$\mathbb{1}$	0	0	0	0

Continued on next column

Supplementary Table 8 – continued

Invariants

Space group #110 : $I4_1cd$

weak	$g^{100}_{00\frac{1}{2}}$	$g^{100}_{0\frac{1}{2}0}$	$g^{110}_{\frac{1}{4}\frac{\bar{1}}{4}\frac{1}{4}}$	$g^{110}_{\frac{\bar{1}}{4}\frac{1}{4}\frac{1}{4}}$	2^{001}	2_1^{001}	4_1^{001}	4_3^{001}
000	0	0	0	0	0	0	0	0
000	0	0	$\mathbb{1}$	$\mathbb{1}$	0	0	$\mathbb{1}$	$\mathbb{1}$
000	$\mathbb{1}$	$\mathbb{1}$	0	0	0	0	$\mathbb{1}$	$\mathbb{1}$
000	$\mathbb{1}$	$\mathbb{1}$	$\mathbb{1}$	$\mathbb{1}$	0	0	0	0

Space group #111 : $P\bar{4}2m$

weak	$m^{110}_{(2)}$	$g^{110}_{\frac{\bar{1}}{2}\frac{1}{2}0}$	2^{001}	2^{100}	$\bar{4}^{001}$
000	0	0	0	0	0
000	0	0	0	$\mathbb{1}$	$\mathbb{1}$
000	2	$\mathbb{1}$	0	0	$\mathbb{1}$
000	2	$\mathbb{1}$	0	$\mathbb{1}$	0
001	0	0	0	0	0
001	0	0	0	1	1
001	2	$\mathbb{1}$	0	0	1
001	2	$\mathbb{1}$	0	1	0
110	0	$\mathbb{1}$	0	0	0
110	0	$\mathbb{1}$	0	1	1
110	2	0	0	0	1
110	2	0	0	1	0
111	0	$\mathbb{1}$	0	0	0
111	0	$\mathbb{1}$	0	1	1
111	2	0	0	0	1
111	2	0	0	1	0

Space group #112 : $P\bar{4}2c$

weak	$g^{110}_{00\frac{1}{2}}$	$g^{110}_{\frac{\bar{1}}{2}\frac{1}{2}\frac{1}{2}}$	2^{001}	2^{100}	$\bar{4}^{001}$
000	0	0	0	0	0
000	0	0	0	$\mathbb{1}$	$\mathbb{1}$
000	$\mathbb{1}$	$\mathbb{1}$	0	0	$\mathbb{1}$
000	$\mathbb{1}$	$\mathbb{1}$	0	$\mathbb{1}$	0
110	0	$\mathbb{1}$	0	0	0
110	0	$\mathbb{1}$	0	1	1
110	$\mathbb{1}$	0	0	0	1
110	$\mathbb{1}$	0	0	1	0

Space group #113 : $P\bar{4}2_1m$

weak	$m^{110}_{(2)}$	$g^{110}_{\frac{\bar{1}}{2}\frac{1}{2}0}$	2^{001}	2_1^{100}	$\bar{4}^{001}$
000	0	0	0	0	0
000	0	0	0	$\mathbb{1}$	$\mathbb{1}$
000	2	$\mathbb{1}$	0	0	$\mathbb{1}$
000	2	$\mathbb{1}$	0	$\mathbb{1}$	0
001	0	0	0	0	0
001	0	0	0	1	1
001	2	$\mathbb{1}$	0	0	1
001	2	$\mathbb{1}$	0	1	0

Space group #114 : $P\bar{4}2_1c$

weak	$g^{110}_{00\frac{1}{2}}$	$g^{110}_{\frac{\bar{1}}{2}\frac{1}{2}\frac{1}{2}}$	2^{001}	2_1^{100}	$\bar{4}^{001}$
000	0	0	0	0	0
000	0	0	0	$\mathbb{1}$	$\mathbb{1}$
000	$\mathbb{1}$	$\mathbb{1}$	0	0	$\mathbb{1}$
000	$\mathbb{1}$	$\mathbb{1}$	0	$\mathbb{1}$	0

Space group #115 : $P\bar{4}m2$

weak	$m^{100}_{(2)}$	2^{001}	2^{110}	2_1^{110}	$\bar{4}^{001}$
000	00	0	0	0	0
000	00	0	$\mathbb{1}$	$\mathbb{1}$	$\mathbb{1}$
000	02	0	0	0	$\mathbb{1}$
000	02	0	$\mathbb{1}$	$\mathbb{1}$	0
000	20	0	0	0	$\mathbb{1}$

Continued on next column

Supplementary Table 8 – continued

Invariants					
000	20	0	$\mathbb{1}$	$\mathbb{1}$	0
000	22	0	0	0	0
000	22	0	$\mathbb{1}$	$\mathbb{1}$	$\mathbb{1}$
001	00	0	0	1	0
001	00	0	1	0	1
001	02	0	0	1	1
001	02	0	1	0	0
001	20	0	0	1	1
001	20	0	1	0	0
001	22	0	0	1	0
001	22	0	1	0	1
110	11	0	0	$\mathbb{1}$	1
110	11	0	$\mathbb{1}$	0	0
110	$1\bar{1}$	0	0	$\mathbb{1}$	0
110	$1\bar{1}$	0	$\mathbb{1}$	0	1
110	$\bar{1}1$	0	0	$\mathbb{1}$	0
110	$\bar{1}1$	0	$\mathbb{1}$	0	1
110	$\bar{1}\bar{1}$	0	0	$\mathbb{1}$	1
110	$\bar{1}\bar{1}$	0	$\mathbb{1}$	0	0
111	11	0	0	0	1
111	11	0	1	1	0
111	$1\bar{1}$	0	0	0	0
111	$1\bar{1}$	0	1	1	1
111	$\bar{1}1$	0	0	0	0
111	$\bar{1}1$	0	1	1	1
111	$\bar{1}\bar{1}$	0	0	0	1
111	$\bar{1}\bar{1}$	0	1	1	0

Space group #116 : $P\bar{4}c2$

weak	$g^{100}_{00\frac{1}{2}}$	2^{001}	2^{110}	2^{110}_1	$\bar{4}^{001}$
000	0	0	0	0	0
000	0	0	$\mathbb{1}$	$\mathbb{1}$	$\mathbb{1}$
000	$\mathbb{1}$	0	0	0	$\mathbb{1}$
000	$\mathbb{1}$	0	$\mathbb{1}$	$\mathbb{1}$	0
110	0	0	0	$\mathbb{1}$	0
110	0	0	$\mathbb{1}$	0	1
110	1	0	0	$\mathbb{1}$	1
110	1	0	$\mathbb{1}$	0	0

Space group #117 : $P\bar{4}b2$

weak	$g^{100}_{0\frac{1}{2}0}$	2^{001}	2^{110}	2^{110}_1	$\bar{4}^{001}$
000	0	0	0	0	0
000	0	0	$\mathbb{1}$	$\mathbb{1}$	$\mathbb{1}$
000	$\mathbb{1}$	0	0	0	$\mathbb{1}$
000	$\mathbb{1}$	0	$\mathbb{1}$	$\mathbb{1}$	0
001	0	0	0	1	1
001	0	0	1	0	0
001	$\mathbb{1}$	0	0	1	0
001	$\mathbb{1}$	0	1	0	1

Space group #118 : $P\bar{4}n2$

weak	$g^{100}_{0\frac{1}{2}\frac{1}{2}}$	2^{001}	2^{110}	2^{110}_1	$\bar{4}^{001}$
000	0	0	0	0	0
000	0	0	$\mathbb{1}$	$\mathbb{1}$	$\mathbb{1}$
000	$\mathbb{1}$	0	0	0	$\mathbb{1}$
000	$\mathbb{1}$	0	$\mathbb{1}$	$\mathbb{1}$	0

Space group #119 : $I\bar{4}m2$

weak	$m^{100}_{(2)}$	$g^{100}_{0\frac{1}{2}\frac{1}{2}}$	2^{001}	2^{110}	2^{001}_1	2^{110}_1	$\bar{4}^{001}$
000	0	0	0	0	0	0	0
000	0	0	0	$\mathbb{1}$	0	$\mathbb{1}$	$\mathbb{1}$
000	2	$\mathbb{1}$	0	0	0	0	$\mathbb{1}$

Continued on next column

Supplementary Table 8 – continued

Invariants							
000	2	$\mathbb{1}$	0	$\mathbb{1}$	0	$\mathbb{1}$	0
111	0	$\mathbb{1}$	0	0	$\mathbb{1}$	1	0
111	0	$\mathbb{1}$	0	1	$\mathbb{1}$	0	1
111	2	0	0	0	$\mathbb{1}$	1	1
111	2	0	0	1	$\mathbb{1}$	0	0

Space group #120 : $I\bar{4}c2$

weak	$g^{100}_{00\frac{1}{2}}$	$g^{100}_{0\frac{1}{2}0}$	2^{001}	2^{110}	2^{001}_1	2^{110}_1	$\bar{4}^{001}$
000	0	0	0	0	0	0	0
000	0	0	0	$\mathbb{1}$	0	$\mathbb{1}$	$\mathbb{1}$
000	$\mathbb{1}$	$\mathbb{1}$	0	0	0	0	$\mathbb{1}$
000	$\mathbb{1}$	$\mathbb{1}$	0	$\mathbb{1}$	0	$\mathbb{1}$	0
111	0	$\mathbb{1}$	0	0	$\mathbb{1}$	1	0
111	0	$\mathbb{1}$	0	1	$\mathbb{1}$	0	1
111	$\mathbb{1}$	0	0	0	$\mathbb{1}$	1	1
111	$\mathbb{1}$	0	0	1	$\mathbb{1}$	0	0

Space group #121 : $I\bar{4}2m$

weak	$m^{110}_{(2)}$	$g^{110}_{00\frac{1}{2}}$	2^{001}	2^{100}	2^{001}_1	2^{100}_1	$\bar{4}^{001}$
000	0	0	0	0	0	0	0
000	0	0	0	$\mathbb{1}$	0	$\mathbb{1}$	$\mathbb{1}$
000	2	$\mathbb{1}$	0	0	0	0	$\mathbb{1}$
000	2	$\mathbb{1}$	0	$\mathbb{1}$	0	$\mathbb{1}$	0
111	0	$\mathbb{1}$	0	0	$\mathbb{1}$	$\mathbb{1}$	0
111	0	$\mathbb{1}$	0	$\mathbb{1}$	$\mathbb{1}$	0	1
111	2	0	0	0	$\mathbb{1}$	$\mathbb{1}$	1
111	2	0	0	$\mathbb{1}$	$\mathbb{1}$	0	0

Space group #122 : $I\bar{4}2d$

weak	$g^{110}_{\frac{1}{4}\frac{\bar{1}}{4}\frac{1}{4}}$	$g^{110}_{\frac{\bar{1}}{4}\frac{1}{4}\frac{1}{4}}$	2^{001}	2^{100}	2^{001}_1	2^{100}_1	$\bar{4}^{001}$
000	0	0	0	0	0	0	0
000	0	0	0	$\mathbb{1}$	0	$\mathbb{1}$	$\mathbb{1}$
000	$\mathbb{1}$	$\mathbb{1}$	0	0	0	0	$\mathbb{1}$
000	$\mathbb{1}$	$\mathbb{1}$	0	$\mathbb{1}$	0	$\mathbb{1}$	0

Space group #143 : $P3$

weak
000
001

Space group #144 : $P3_1$

weak
000
001

Space group #145 : $P3_2$

weak
000
001

Space group #146 : $R3$

weak
000
111

Space group #149 : $P312$

weak	2^{120}	2^{120}_1
000	0	0
000	$\mathbb{1}$	$\mathbb{1}$
001	0	0
001	1	1

Space group #150 : $P321$

weak	2^{100}	2^{100}_1
000	0	0

Continued on next column

Supplementary Table 8 – continued

Invariants

000	$\mathbb{1}$	$\mathbb{1}$
001	0	0
001	1	1

Space group #151 : $P3_112$

weak	2^{120}	2_1^{120}
000	0	0
000	$\mathbb{1}$	$\mathbb{1}$
001	0	0
001	1	1

Space group #152 : $P3_121$

weak	2^{100}	2_1^{100}
000	0	0
000	$\mathbb{1}$	$\mathbb{1}$
001	0	0
001	1	1

Space group #153 : $P3_212$

weak	2^{120}	2_1^{120}
000	0	0
000	$\mathbb{1}$	$\mathbb{1}$
001	0	0
001	1	1

Space group #154 : $P3_221$

weak	2^{100}	2_1^{100}
000	0	0
000	$\mathbb{1}$	$\mathbb{1}$
001	0	1
001	1	0

Space group #155 : $R32$

weak	2^{100}	2_1^{100}
000	0	0
000	$\mathbb{1}$	$\mathbb{1}$
111	0	0
111	1	1

Space group #156 : $P3m1$

weak	$m_{(2)}^{\bar{2}10}$	$g_{\frac{1}{2}10}^{\bar{2}10}$
000	0	0
000	2	$\mathbb{1}$
001	0	0
001	2	$\mathbb{1}$

Space group #157 : $P31m$

weak	$m_{(2)}^{010}$	$g_{\frac{1}{2}00}^{010}$
000	0	0
000	2	$\mathbb{1}$
001	0	0
001	2	$\mathbb{1}$

Space group #158 : $P3c1$

weak	$g_{00\frac{1}{2}}^{\bar{2}10}$	$g_{\frac{1}{2}1\frac{1}{2}}^{\bar{2}10}$
000	0	0
000	$\mathbb{1}$	$\mathbb{1}$

Space group #159 : $P31c$

weak	$g_{00\frac{1}{2}}^{010}$	$g_{\frac{1}{2}0\frac{1}{2}}^{010}$
000	0	0
000	$\mathbb{1}$	$\mathbb{1}$

Space group #160 : $R3m$

weak	$m_{(2)}^{\bar{2}10}$	$g_{\frac{1}{6}\frac{1}{3}\frac{1}{3}}^{\bar{2}10}$

Continued on next column

Supplementary Table 8 – continued

Invariants

000	0	0
000	2	$\mathbb{1}$
111	0	$\mathbb{1}$
111	2	0

Space group #161 : $R3c$

weak	$g_{\frac{\bar{1}}{6}\frac{\bar{1}}{3}\frac{1}{6}}^{\bar{2}10}$	$g_{00\frac{1}{2}}^{\bar{2}10}$
000	0	0
000	$\mathbb{1}$	$\mathbb{1}$

Space group #168 : $P6$

weak	2^{001}	6^{001}
000	0	0
000	$\mathbb{1}$	$\mathbb{1}$
001	0	0
001	$\mathbb{1}$	$\mathbb{1}$

Space group #169 : $P6_1$

weak	2_1^{001}	6_1^{001}
000	0	0
000	$\mathbb{1}$	$\mathbb{1}$

Space group #170 : $P6_5$

weak	2_1^{001}	6_5^{001}
000	0	0
000	$\mathbb{1}$	$\mathbb{1}$

Space group #171 : $P6_2$

weak	2^{001}	6_2^{001}
000	0	0
000	$\mathbb{1}$	$\mathbb{1}$
001	0	$\mathbb{1}$
001	$\mathbb{1}$	0

Space group #172 : $P6_4$

weak	2^{001}	6_4^{001}
000	0	0
000	$\mathbb{1}$	$\mathbb{1}$
001	0	0
001	$\mathbb{1}$	$\mathbb{1}$

Space group #173 : $P6_3$

weak	2_1^{001}	6_3^{001}
000	0	0
000	$\mathbb{1}$	$\mathbb{1}$

Space group #177 : $P622$

weak	2^{001}	2^{010}	$2^{1\bar{1}0}$	6^{001}	2_1^{010}	$2_1^{1\bar{1}0}$
000	0	0	0	0	0	0
000	0	$\mathbb{1}$	$\mathbb{1}$	0	$\mathbb{1}$	$\mathbb{1}$
000	$\mathbb{1}$	0	$\mathbb{1}$	$\mathbb{1}$	0	$\mathbb{1}$
000	$\mathbb{1}$	$\mathbb{1}$	0	$\mathbb{1}$	$\mathbb{1}$	0
001	0	0	0	0	1	1
001	0	1	1	0	0	0
001	$\mathbb{1}$	0	1	$\mathbb{1}$	1	0
001	$\mathbb{1}$	1	0	$\mathbb{1}$	0	1

Space group #178 : $P6_122$

weak	2^{010}	$2^{1\bar{1}0}$	2_1^{001}	2_1^{010}	$2_1^{1\bar{1}0}$	6_1^{001}
000	0	0	0	0	0	0
000	0	$\mathbb{1}$	$\mathbb{1}$	0	$\mathbb{1}$	$\mathbb{1}$
000	$\mathbb{1}$	0	$\mathbb{1}$	$\mathbb{1}$	0	$\mathbb{1}$
000	$\mathbb{1}$	$\mathbb{1}$	0	$\mathbb{1}$	$\mathbb{1}$	0

Space group #179 : $P6_522$

weak	2^{010}	$2^{1\bar{1}0}$	2_1^{001}	2_1^{010}	$2_1^{1\bar{1}0}$	6_5^{001}

Continued on next column

Supplementary Table 8 – continued

Invariants

000	0	0	0	0	0	0
000	0	𝟙	𝟙	0	𝟙	𝟙
000	𝟙	0	𝟙	𝟙	0	𝟙
000	𝟙	𝟙	0	𝟙	𝟙	0

Space group #180 : $P6_222$

weak	2^{001}	2^{010}	$2^{1\bar{1}0}$	2_1^{010}	$2_1^{1\bar{1}0}$	6_2^{001}
000	0	0	0	0	0	0
000	0	𝟙	𝟙	𝟙	𝟙	0
000	𝟙	0	𝟙	0	𝟙	𝟙
000	𝟙	𝟙	0	𝟙	0	𝟙
001	0	0	1	1	0	𝟙
001	0	1	0	0	1	𝟙
001	𝟙	0	0	1	1	0
001	𝟙	1	1	0	0	0

Space group #181 : $P6_422$

weak	2^{001}	2^{010}	$2^{1\bar{1}0}$	2_1^{010}	$2_1^{1\bar{1}0}$	6_4^{001}
000	0	0	0	0	0	0
000	0	𝟙	𝟙	𝟙	𝟙	0
000	𝟙	0	𝟙	0	𝟙	𝟙
000	𝟙	𝟙	0	𝟙	0	𝟙
001	0	0	1	1	0	0
001	0	1	0	0	1	0
001	𝟙	0	0	1	1	𝟙
001	𝟙	1	1	0	0	𝟙

Space group #182 : $P6_322$

weak	2^{010}	$2^{1\bar{1}0}$	2_1^{001}	2_1^{010}	$2_1^{1\bar{1}0}$	6_3^{001}
000	0	0	0	0	0	0
000	0	𝟙	𝟙	0	𝟙	𝟙
000	𝟙	0	𝟙	𝟙	0	𝟙
000	𝟙	𝟙	0	𝟙	𝟙	0

Space group #183 : $P6mm$

weak	$m_{(2)}^{\bar{1}20}$	$m_{(2)}^{1\bar{1}0}$	$g_{1\frac{1}{2}0}^{\bar{1}20}$	$g_{\frac{1}{2}\frac{1}{2}0}^{1\bar{1}0}$	2^{001}	6^{001}
000	0	0	0	0	0	0
000	0	2	0	𝟙	𝟙	𝟙
000	2	0	𝟙	0	𝟙	𝟙
000	2	2	𝟙	𝟙	0	0
001	0	0	0	0	0	0
001	0	2	0	𝟙	𝟙	𝟙
001	2	0	𝟙	0	𝟙	𝟙
001	2	2	𝟙	𝟙	0	0

Space group #184 : $P6cc$

weak	$g_{00\frac{1}{2}}^{\bar{1}20}$	$g_{1\frac{1}{2}\frac{1}{2}}^{\bar{1}20}$	$g_{00\frac{1}{2}}^{1\bar{1}0}$	$g_{\frac{1}{2}\frac{1}{2}\frac{1}{2}}^{1\bar{1}0}$	2^{001}	6^{001}
000	0	0	0	0	0	0
000	0	0	𝟙	𝟙	𝟙	𝟙
000	𝟙	𝟙	0	0	𝟙	𝟙
000	𝟙	𝟙	𝟙	𝟙	0	0

Space group #185 : $P6_3cm$

weak	$m_{(2)}^{1\bar{1}0}$	$g_{00\frac{1}{2}}^{\bar{1}20}$	$g_{1\frac{1}{2}\frac{1}{2}}^{\bar{1}20}$	$g_{\frac{1}{2}\frac{1}{2}0}^{1\bar{1}0}$	2_1^{001}	6_3^{001}
000	0	0	0	0	0	0
000	0	𝟙	𝟙	0	𝟙	𝟙
000	2	0	0	𝟙	𝟙	𝟙
000	2	𝟙	𝟙	𝟙	0	0

Space group #186 : $P6_3mc$

weak	$m_{(2)}^{\bar{1}20}$	$g_{1\frac{1}{2}0}^{\bar{1}20}$	$g_{00\frac{1}{2}}^{1\bar{1}0}$	$g_{\frac{1}{2}\frac{1}{2}\frac{1}{2}}^{1\bar{1}0}$	2_1^{001}	6_3^{001}
000	0	0	0	0	0	0
000	0	0	𝟙	𝟙	𝟙	𝟙

Continued on next column

Supplementary Table 8 – continued

Invariants

000	2	𝟙	0	0	𝟙	𝟙
000	2	𝟙	𝟙	𝟙	0	0

Space group #195 : $P23$

weak	2^{001}
000	0
111	0

Space group #196 : $F23$

weak	2^{001}	2_1^{001}

Space group #197 : $I23$

weak	2^{001}	2_1^{001}
000	0	0
111	0	𝟙

Space group #198 : $P2_13$

weak	2_1^{001}

Space group #199 : $I2_13$

weak	2^{001}	2_1^{001}
000	0	0
111	𝟙	0

Space group #207 : $P432$

weak	2^{001}	2^{011}	4^{001}	2_1^{011}
000	0	0	0	0
000	0	𝟙	𝟙	𝟙
111	0	0	0	1
111	0	1	1	0

Space group #208 : $P4_232$

weak	2^{001}	2^{011}	2_1^{011}	4_2^{001}
000	0	0	0	0
000	0	𝟙	𝟙	𝟙

Space group #209 : $F432$

weak	2^{001}	2^{011}	4^{001}	2_1^{001}	2_1^{011}	4_2^{001}
000	0	0	0	0	0	0
000	0	𝟙	𝟙	0	𝟙	𝟙

Space group #210 : $F4_132$

weak	2^{001}	2^{011}	2_1^{001}	2_1^{011}	4_1^{001}	4_3^{001}
000	0	0	0	0	0	0
000	0	𝟙	0	𝟙	𝟙	𝟙

Space group #211 : $I432$

weak	2^{001}	2^{011}	4^{001}	2_1^{001}	2_1^{011}	4_2^{001}
000	0	0	0	0	0	0
000	0	𝟙	𝟙	0	𝟙	𝟙
111	0	0	0	𝟙	1	𝟙
111	0	1	𝟙	𝟙	0	0

Space group #212 : $P4_332$

weak	2^{011}	2_1^{001}	2_1^{011}	4_3^{001}
000	0	0	0	0
000	𝟙	0	𝟙	𝟙

Space group #213 : $P4_132$

weak	2^{011}	2_1^{001}	2_1^{011}	4_1^{001}
000	0	0	0	0
000	𝟙	0	𝟙	𝟙

Space group #214 : $I4_132$

weak	2^{001}	2^{011}	2_1^{001}	2_1^{011}	4_1^{001}	4_3^{001}
000	0	0	0	0	0	0
000	0	𝟙	0	𝟙	𝟙	𝟙
111	𝟙	0	0	1	0	𝟙
111	𝟙	1	0	0	𝟙	0

Continued on next column

Supplementary Table 8 – continued

Invariants

Space group #215 : $P\bar{4}3m$

weak	$m^{011}_{(2)}$	$g^{011}_{0\frac{\bar{1}}{2}\frac{1}{2}}$	2^{001}	$\bar{4}^{001}$
000	0	0	0	0
000	2	$\mathbb{1}$	0	$\mathbb{1}$
111	0	$\mathbb{1}$	0	0
111	2	0	0	1

Space group #216 : $F\bar{4}3m$

weak	$m^{011}_{(2)}$	$g^{011}_{\frac{1}{2}\frac{\bar{1}}{4}\frac{1}{4}}$	2^{001}	2^{001}_{1}	$\bar{4}^{001}$
000	0	0	0	0	0
000	2	$\mathbb{1}$	0	0	$\mathbb{1}$

Space group #217 : $I\bar{4}3m$

weak	$m^{011}_{(2)}$	$g^{011}_{\frac{1}{2}00}$	2^{001}	2^{001}_{1}	$\bar{4}^{001}$
000	0	0	0	0	0
000	2	$\mathbb{1}$	0	0	$\mathbb{1}$
111	0	$\mathbb{1}$	0	$\mathbb{1}$	0
111	2	0	0	$\mathbb{1}$	1

Space group #218 : $P\bar{4}3n$

weak	$g^{011}_{\frac{1}{2}00}$	$g^{011}_{\frac{1}{2}\frac{\bar{1}}{2}\frac{1}{2}}$	2^{001}	$\bar{4}^{001}$
000	0	0	0	0
000	$\mathbb{1}$	$\mathbb{1}$	0	$\mathbb{1}$

Space group #219 : $F\bar{4}3c$

weak	$g^{011}_{\frac{1}{2}00}$	$g^{011}_{0\frac{\bar{1}}{4}\frac{1}{4}}$	2^{001}	2^{001}_{1}	$\bar{4}^{001}$
000	0	0	0	0	0
000	$\mathbb{1}$	$\mathbb{1}$	0	0	$\mathbb{1}$

Space group #220 : $I\bar{4}3d$

weak	$g^{011}_{\frac{1}{4}\frac{1}{4}\frac{\bar{1}}{4}}$	$g^{011}_{\frac{1}{4}\frac{\bar{1}}{4}\frac{1}{4}}$	2^{001}	2^{001}_{1}	$\bar{4}^{001}$
000	0	0	0	0	0
000	$\mathbb{1}$	$\mathbb{1}$	0	0	$\mathbb{1}$

Supplementary References

[1] Wang, Z., Alexandradinata, A., Cava, R. J. & Bernevig, B. A. Hourglass fermions. *Nature* **532**, 189 EP – (2016).

[2] Alexandradinata, A., Wang, Z. & Bernevig, B. A. Topological Insulators from Group Cohomology. *Physical Review X* **6** (2016). ArXiv: 1604.03952.

[3] Ezawa, M. Hourglass fermion surface states in stacked topological insulators with nonsymmorphic symmetry. *Phys. Rev. B* **94**, 155148 (2016).

[4] Fang, C. & Fu, L. Rotation Anomaly and Topological Crystalline Insulators. *ArXiv e-prints* (2017). 1709.01929.

[5] Song, Z., Fang, Z. & Fang, C. $(d-2)$-dimensional edge states of rotation symmetry protected topological states. *Phys. Rev. Lett.* **119**, 246402 (2017).

[6] Hsieh, T. H. *et al.* Topological crystalline insulators in the snte material class. *Nature Communications* **3**, 982 EP – (2012).

[7] Fulga, I. C., Avraham, N., Beidenkopf, H. & Stern, A. Coupled-layer description of topological crystalline insulators. *Phys. Rev. B* **94**, 125405 (2016).

[8] Fu, L. & Kane, C. L. Topological insulators with inversion symmetry. *Phys. Rev. B* **76**, 045302 (2007).

[9] Hahn, T. *International tables for crystallography* (Dordrecht; London : Published for the International Union of Crystallography by Kluwer Academic Publishers, 2002., 2002).

[10] Po, H. C., Vishwanath, A. & Watanabe, H. Symmetry-based indicators of band topology in the 230 space groups. *Nature Communications* **8**, 50 (2017).

[11] Bradlyn, B. *et al.* Topological quantum chemistry. *Nature* **547**, 298 EP – (2017).

[12] Altmann, S. L. & Herzig, P. *Point-group theory tables* (Clarendon Press, 1994).

[13] Aroyo, M. I. *et al.* Brillouin-zone database on the Bilbao Crystallographic Server. *Acta Crystallographica Section A: Foundations and Advances* **70**, 126–137 (2014).

[14] Fang, C., Gilbert, M. J. & Bernevig, B. A. Bulk topological invariants in noninteracting point group symmetric insulators. *Phys. Rev. B* **86**, 115112 (2012).

[15] Shen, S.-Q. *Topological Insulators: Dirac Equation in Condensed Matters* (Springer, Heidelberg ; New York, 2013), 2013 edn.

[16] Chiu, C.-K., Teo, J. C., Schnyder, A. P. & Ryu, S. Classification of topological quantum matter with symmetries. *Reviews of Modern Physics* **88**, 035005 (2016).

[17] Aroyo, M. I. *et al.* Bilbao Crystallographic Server: I. Databases and crystallographic computing programs. *Zeitschrift füer Kristallographie - Crystalline Materials* **221**, 15–27 (2009).

[18] Vergniory, M. G. *et al.* Graph theory data for topological quantum chemistry. *Phys. Rev. E* **96**, 023310 (2017).

[19] Elcoro, L. *et al.* Double crystallographic groups and their representations on the Bilbao Crystallographic Server. *Journal of Applied Crystallography* **50**, 1457–1477 (2017).
